# Temporal dynamics of early inflammatory markers after professional dental cleaning: a meta-analysis and spline-based meta-regression of TNF-α, IL-1β, IL-6, and (hs)CRP

**DOI:** 10.3389/fimmu.2025.1634622

**Published:** 2025-08-28

**Authors:** Martina Cardisciani, Sara Di Nicolantonio, Serena Altamura, Eleonora Ortu, Rita Del Pinto, Davide Pietropaoli

**Affiliations:** ^1^ Department of Life, Health & Environmental Sciences, University of L’Aquila, L’Aquila, Italy; ^2^ Center of Oral Diseases, Prevention and Translational Research - Dental Clinic, L’Aquila, Italy; ^3^ Department of Medicine, Case Western Reserve University, School of Medicine, Cleveland, OH, United States; ^4^ Unit of Internal Medicine and Nephrology, San Salvatore Hospital, Center for Hypertension and Cardiovascular Prevention, L’Aquila, Italy; ^5^ Department of Physical and Chemical Science, University of L’Aquila, L’Aquila, Italy

**Keywords:** periodontitis, inflammation, tumor necrosis factor-α, interleukin-6, interleukin-1β, C-reactive protein, cytokines, meta-analysis

## Abstract

**Introduction:**

Chronic periodontitis is linked to systemic inflammation and cardiovascular risk, yet the temporal trajectory and magnitude of systemic cytokine reduction following nonsurgical-periodontal-therapy (NSPT) remain underexplored. We conducted a meta-analysis and spline-based meta-regression to assess whether intensive NSPT, compared to standard therapy, produces sustained reductions in circulating TNF-α, IL-1β, IL-6 and high-sensitivity C-reactive protein (hs-CRP).

**Methods:**

We systematically searched major databases for interventional studies published until January 2024 comparing intensive vs. standard NSPT on inflammatory markers. Using a custom R pipeline, standardized mean differences (SMDs), 95% confidence intervals (Cis) heterogeneity (I²) and stratified analyses by phenotype (e.g., smoking, diabetes) and time were performed. A spline-based mixed-effects meta-regression explored temporal dynamics of inflammatory reduction in the intensive group.

**Results:**

From 216 observations (14,374 paired values), intensive NSPT led to significantly greater reductions in TNF-α (SMD –0.59, 95% CI –1.02 to –0.16; *P*=0.008), IL-6 (SMD –0.20, 95% CI –0.39 to –0.00; *P*=0.046) and hs-CRP (SMD –1.17, 95% CI –2.18 to –0.16; *P*=0.024) compared to standard therapy. IL-1β showed a near-significant reduction (SMD –4.14, *P*=0.052). Standard therapy was paradoxically associated with greater CRP reduction (SMD –0.30, *P*=0.001). Age, tooth count and year of publication moderated effects. Early benefits emerged within 3 months for TNF-α and 6 months for IL-1β. Although no strong nonlinear time-response was confirmed (QM = 4.23, *P*=0.2372),a potential rebound in cytokine suppression was suggested. The overall anti-inflammatory effect remained significant.

**Discussion:**

Intensive NSPT reduces systemic inflammation particularly in younger, non-smoking individuals. The potential rebound in inflammatory markers underscores the need for longitudinal studies but, supports the systemic immunoregulatory effect of periodontal therapy.

**Systematic Review Registration:**

https://www.crd.york.ac.uk/PROSPERO, identifier CDR42024503063.

## Graphical Abstract

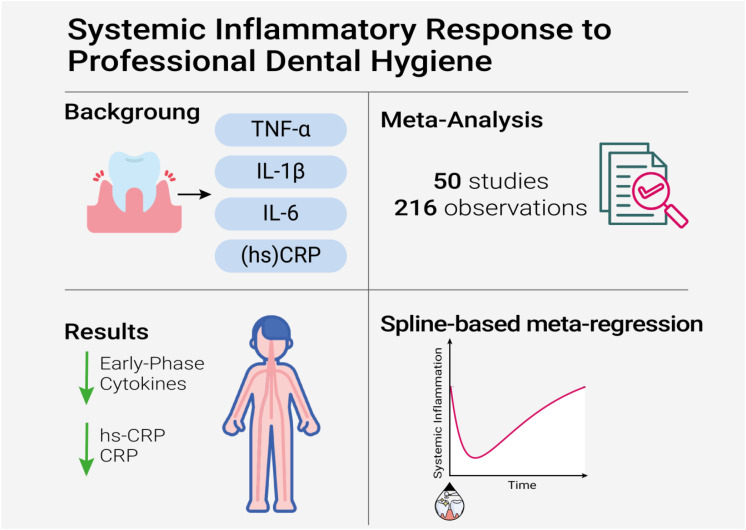

## Introduction

One of the most significant medical breakthroughs of recent years is the recognition that the immune system and inflammation are key players not only in self-directed and communicable disorders, or in defense against injury, but also in a broad range of non-communicable diseases that disproportionately affect global morbidity and mortality (1). Periodontal disease (PD) is a chronic inflammatory condition that, if not adequately treated, can progress to destruction of the supporting tissues of the teeth and alveolar bone. With more than 790 million people affected, periodontitis is the sixth most prevalent noncommunicable disease worldwide (2, 3) and a global public health problem with a huge economic impact, estimated at nearly $300 billion annually in the United States alone (4). PD is considered a multifactorial chronic inflammatory condition in which dysbiosis of the oral microbiome plays a key role (5, 6). Dysregulation of the immune system and increased levels of circulating pro-inflammatory cytokines, such as tumor necrosis factor alpha (TNF-α), interleukin (IL)-1, IL-1β, IL-6, and C-reactive protein (CRP), are detectable even in the very early stages of the disease (7) and may contribute to the establishment of a chronic, systemic, low-grade inflammatory state.

The role of periodontitis in the development of many other systemic conditions and its independent association with arterial hypertension (8), cardiovascular diseases, diabetes (9), dyslipidemia and obesity are well known, so much so that it has recently been included among the modifiable risk factors for such diseases. The pathogenetic mechanisms underlying this association are mainly related to low-grade bacteremia/endotoxemia and its inflammatory sequelae (10), the dissemination of oxidative stress-induced metabolites, and perivascular inflammation (9).

Although PD is considered a chronic and irreversible condition, meta-analytical data suggest a positive effect of nonsurgical periodontal therapy (NSPT) on disease progression, with improvement of local signs of inflammation. However, the effectiveness of NSPT in mitigating systemic inflammation, expressed in terms of serum cytokines concentrations and (high-sensitivity, hs) CRP levels, remains controversial. Furthermore, any concomitant local or systemic pharmacological therapy aimed at controlling inflammation could confound the true efficacy of NSPT alone on inflammation. A 2016 meta-analysis by Akram and colleagues reported inconsistent findings regarding the efficacy of NSPT in reducing serum cytokines (11), while other authors found a benefit of NSPT only on serum IL-6 levels in the short term, and only among obese individuals (12).

Of note, NSPT can have a different impact on systemic markers of inflammation depending on whether the standard (supragingival) or the intensive (supra- and subgingival) treatment is performed. Therefore, we performed an updated meta-analysis and systematic review to evaluate the impact of intensive versus standard NSPT on circulating markers of inflammation in patients with periodontitis.

## Methods

### Protocol and registration

The protocol for this systematic review and meta-analysis is registered with PROSPERO (CRD42024503063). The study followed the Preferred Reporting Items for Systematic Reviews and Meta-analysis (PRISMA) reporting guidelines (**Supplementary Table 1**).

### Search strategy

The MEDLINE database was queried through January 16th, 2024 for studies that published the effects of intensive and standard NSPT on serum cytokines in adult patients with periodontitis. The following search algorithm was used in the R library “rentrez”: (“periodontal diseases”[MeSH Terms] AND (“cytokines”[MeSH Terms] OR “c reactive protein”[MeSH Terms])) AND ((fha[Filter]) AND (clinicalstudy[Filter] OR clinicaltrial[Filter] OR clinicaltrialprotocol[Filter] OR clinicaltrialphasei[Filter] OR clinicaltrialphaseii[Filter] OR clinicaltrialphaseiii[Filter] OR clinicaltrialphaseiv[Filter] OR comparativestudy[Filter] OR controlledclinicaltrial[Filter] OR dataset[Filter] OR meta-analysis[Filter] OR observationalstudy[Filter] OR pragmaticclinicaltrial[Filter] OR randomizedcontrolledtrial[Filter])). Additional manual search was performed on Embase, Scopus and Cochrane. No date or language restrictions were applied.

### Eligibility criteria

Studies were selected based on the following inclusion criteria: randomized clinical trials, randomized double-blind clinical trials, comparative studies, prospective exploratory monocentric trials, randomized controlled trials, interventional controlled non-randomized clinical trials, controlled clinical trials, single-center interventional studies, randomized controlled parallel clinical trials, observational studies, prospective randomized clinical studies, and cohort studies. Eligible studies involved adult participants aged 18 years or older who had been diagnosed with periodontitis. Additionally, included studies investigated the effects of non-surgical periodontal therapy (NSPT) on serum cytokines - specifically TNF-α, IL-1β, IL-6 - and high-sensitivity C-reactive protein (hs-CRP) or CRP, in accordance with the following PICOS question: “*In adults diagnosed with periodontitis (P), is intensive NSPT (I) more effective than standard treatment (C) in reducing serum cytokines and (hs)CRP (O), as assessed in randomized controlled trials and prospective observational studies (S)?”*

### Outcome variables

The primary outcomes were changes in serum cytokines levels, namely TNF-α, IL-1β, and IL-6 (expressed in mg/L). Secondary outcomes were changes in CRP and hs-CRP (expressed in mg/L). All data reported in different units of measurement in the original studies were converted to mg/L. Outcome variables were analyzed as the change from pre-treatment to each available post-treatment follow-up.

### Study selection and data extraction

Study selection and data extraction were performed using a standardized data extraction Excel form.

Two reviewers (S.D.N. and M.C.) independently reviewed titles and abstracts to assess eligibility. Inter-reviewer reliability in the study selection process was determined by the Cohen κ test, assuming an acceptable threshold value of 0.81 (13, 14). Full-text articles of the potentially eligible studies were then analyzed to assess their inclusion. Disagreements regarding inclusion between reviewers were resolved by discussion and consensus.

Data were extracted by the two reviewers (S.D.N. and M.C.) independently, and inconsistencies were resolved through consensus discussions and consultation with a senior author (D.P). Extracted data included (see **Supplementary Methods 2** for detailed information): 1) study identifiers and methodology; 2) participants characteristics; 3) type of treatment; 4) cytokines serum levels before and after treatments; 5) when available, serum high-sensitivity C-reactive protein (hs-CRP) and CRP levels before and after treatments. If a study included multiple groups, only pertinent groups were extracted. When primary outcome data were incomplete or inconsistently reported and clarification was necessary to determine study eligibility, authors were contacted for clarification, and the study was excluded if no response was received within 4 weeks. Other reasons for exclusion, including lack of data of interest and population characteristics/study methodologies different than required, are detailed in **Supplementary Table 3**.

### Risk of bias and quality assessment

The bias assessment was conducted by two reviewers (S.D.N. and M.C.) independently using specific tools for observational or interventional studies, and inconsistencies were resolved through consensus discussions and consultation with a senior author (D.P). Specifically, the Newcastle–Ottawa quality assessment Scale (NOS) (15) was used to assess the risk of bias for case-control and cohort studies, while the Cochrane risk of bias template (Rob2.0) was used for randomized clinical trials (16). In order to perform meta-regression, both scales were converted into continuous values. After determining the score of each study, an overall estimation of plausible risk of bias was performed for each selected study. For randomized clinical trials, a low risk of bias was estimated when all of the criteria were met, a moderate risk was estimated when ≥1 criteria were partially met, and a high risk of bias was estimated when ≥1 criteria were not met. For case-control and cohort studies, the maximum score was 9, the minimum was 0. It was decided *a priori* that a score of 7 reflected high methodology quality (i.e., low risk of bias), a score of 5 or 6 indicated moderate quality (i.e. some concerns), and a score of 4 or less indicated a low quality (i.e., high risk of bias).

### Sex as a biological variable

Participants sex was reported as assessed in the original studies, and the variable was used for stratified analyses. On this basis, sex-specific assessments of post-treatment changes in selected cytokines could be performed.

### Statistical analysis

Study data were pooled for subsequent analyses in the software R. The meta-effect on serum inflammation of intensive versus standard NSPT was calculated from standardized mean differences (SMDs) and 95% confidence intervals (CI) using both common and random effect models. Due to the expected interstudy heterogeneity, the random effect model was preferred for the interpretation of findings.

We conducted stratified meta-analyses based on the type of inflammatory marker assessed (TNF-α, IL-1β, IL-6, CRP and hs-CRP) within and between treatment types. For each marker and treatment type, further stratification was performed based on the elapsed time to assessment of change, participants sex, smoking habits, and diabetes status, provided that at least two studies were available within each stratification. For studies reporting on intensive NSPT, stratification based on additional local treatments (full-mouth disinfection, FMD; laser treatment; curcumin gel) was performed. The elapsed time to change was assessed: 1) as reported in the original studies; 2) according to predefined time spans (<4 to 6 weeks; <3 months; <6 months; ≥6 months) based on biological plausibility. The meta-effect was considered significant for P<0.05. Forest plots for each meta-analysis, generated with specific R libraries, namely “meta” (17) and “forestploter” (18), present the raw data (sample sizes, means, SDs), point estimates (displayed as blocks) and CIs (displayed as lines) for the chosen effect, heterogeneity statistic (I^2^), overall average effect with related statistics, and percent weight given to each study.

We also performed random-effects meta-regression with different moderators (elapsed time to assessment of change, study year, mean age, female sex ratio, smokers, number of teeth before treatment, diabetes ratio).

Heterogeneity between studies was assessed by the I^2^ statistic as defined by the Cochrane Handbook for Systematic Reviews, and an I^2^ value of 50% or greater was considered to represent a substantial heterogeneity.

Publication bias was investigated by visual detection (funnel plot assessment) and quantitative analysis (regression asymmetry test, trim-and-fill method)

A custom in-house R pipeline was developed to perform common and random-effects meta-analyses for TNF-α, IL-1β, IL-6, CRP, and hs-CRP. Subsequently, stratified analyses and meta-regressions were conducted for each marker.

### Meta-regression analysis

To explore the temporal dynamics of inflammatory marker modulation following intensive NSPT, we conducted a meta-regression using a restricted cubic spline model. Analyses were performed using the metafor package (version 4.4-0) in R (19). Effect sizes (SMDs) and standard errors were extracted from the random-effects meta-analysis model of studies reporting inflammatory markers outcomes (TNF-α, IL-1β, IL-6, or high-sensitivity C-reactive protein) in the intensive NSPT subgroup. The time variable was defined as the number of days between the intervention and post-intervention cytokine measurement. To allow for potential non-linear relationships between elapsed time from intensive NSPT and effect size, we applied a meta-regression with restricted cubic splines (3 degrees of freedom). This approach provides a flexible framework to model gradual changes in effect estimates over time without imposing a linearity constraint (20, 21). Predictions were generated across the observed range of timepoints (0–365 days) to derive the fitted SMD trajectory along with 95% confidence intervals. These predictions were plotted to visualize the time-dependent trend in treatment effects. All models used the DerSimonian and Laird estimator for between-study variance (τ^2^) (19). We verified the stability of the spline model and inspected residuals to ensure model adequacy.

All code used in the analysis has been deposited in the GitHub repository (https://github.org/pietropaolilab).

## Results

### Search results and description of included studies

**Figure 1:**
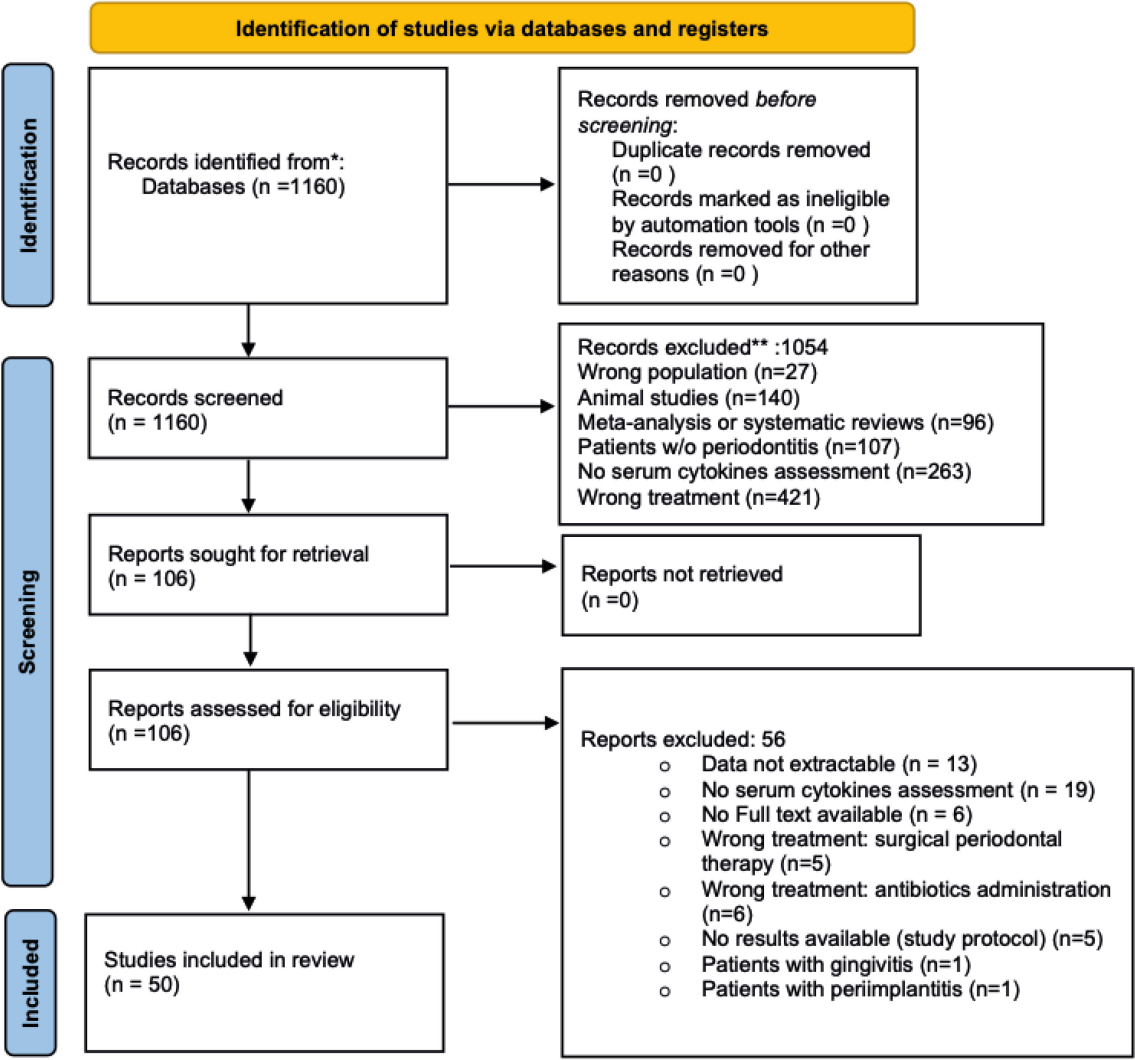
PRISMA flow diagram.

On January 16th, 2024, 1160 citations were retrieved, and 106 articles were screened for eligibility, of which 50 met our predefined inclusion criteria, for a total of 2340 patients included (45.5% women, mean age 50,47 ± 8,9 years) (see **Figure 1** for data reduction). The included studies spanned a timeframe of 33 years (1990 - 2023). The descriptive characteristics of the included studies are presented in **Table 1**. Briefly, 498 (21,3%) participants underwent standard NSPT, while 1842 (78,3%) received intensive NSPT. One study evaluated TNF-α only, while two focused on IL-1β. One study assessed both TNF-α and IL-1β, two studies examined TNF-α and IL-6, and four studies analyzed TNF-α, IL-1β, and IL-6 simultaneously. Furthermore, 19 studies also investigated CRP, and 21 studies evaluated hs-CRP. Diabetes and smoking status were reported by 16 and 22 studies, respectively, with 10 studies evaluating both conditions. Of participants receiving standard NSPT, 21,5% suffered from diabetes and 23% were smokers. Among those receiving intensive NSPT, 34,5% suffered from diabetes and 15% were smokers. Assessment of changes in inflammatory biomarkers occurred between 21 days and 56 days for 5 studies, 1 month for 6 studies, within 2 months for 2 studies, 6 weeks for 5 studies, within 3 months for 15 studies, within 6 months for 14 studies, and beyond 6 months for 3 studies. Most studies used the ELISA assay. Thirty studies matched for age/sex and at least one other *a priori* defined confounding variable, whereas twenty studies only matched for age/sex.

**Table 1:** Characteristics of the included studies.

Source	Country	Population (n)	Mean age	Patients w/periodontitis (n)	Female (n)	Smokers (n)	Patients w/ diabetes (n)	Procedure intervention	Cytokine	Assay type	Follow up
M. Ide et al., 2003 (22)	United Kingdom	24	47.8 years	24	11	0	0	Standard treatment	TNF〈, IL-1®, IL-6, CRP	ELISA (TNF〈, IL-1®, IL-6) Monoclonal antibodies (CRP)	42 days
F. D’Aiuto et al., 2005 (23)	United Kingdom	21	48.7 years	21	9	6	0	Standard treatment	IL-6, CRP	ELISA (IL-6) Immunoturbidimetic assay (CRP)	60 days
S. Offenbacher et al., 2006 (24)	USA	28	26.8 years	28	28	0	0	Intensive treatment	IL-6, hs-CRP	ELISA (IL-6) Latex-enhanced nephelometry (hs-CRP)	42 days
M. Tonetti et al., 2018 (25)	Italy	120	Standard group: 47.8 years Intensive group: 47.7 years	120	Standard treatment (n= 29) Intensive treatment (n= 31)	Standard treatment (n= 20) Intensive treatment (n= 18)	0	Standard treatment (n= 59) Intensive treatment (n= 61)	IL-6, CRP	ELISA (IL-6) Immunoturbidimetic assay (CRP)	30 days 60 days 180 days
G. Tüter et al., 2007 (26)	Turkey	18	52.2 years	18	1	0	0	Intensive treatment	hs-CRP	Latex- enhanced nephelometry	42 days
Y. Ushida et al., 2008 (27)	Japan	24	Standard treatment full mouth disinfection: 50.7 years Standard treatment quadrant mouth disinfection: 51.7 years	24	Standard treatment full mouth disinfection (n=7) Standard treatment quadrant mouth disinfection (n=8)	0	0	Standard treatment full mouth disinfection (n=12) Standard treatment quadrant mouth disinfection (n=12)	IL-6, CRP	ELISA (IL-6) Latex-enhanced nephelometry (CRP)	30 days
S. Offenbacher et al., 2009 (28)	USA	126	59.5 years	126	47	29	30	Intensive treatment	hs-CRP	Latex-enhanced nephelometry	180 days
F. Vidal et al., 2009 (29)	Brazil	11	48.9 years	11	6	2	0	Standard treatment	IL-6, CRP	ELISA	90 days
S. Revert et al., 2009 (30)	Sweden	28	56.7 years	28	11	15	0	Standard treatment	hs-CRP	Immunoturbidimetic assay	49 days
L. Kardesxler et al., 2010 (31)	Turkey	40	52.29 years	40	13	14	25	Standard treatment	CRP, TNF〈, IL-6	ELISA	30 days 60 days 90 days
P.M. Duarte et al., 2010 (32)	Brazil	14	42.7 years	14	6	0	0	Intensive treatment	TNF〈	ELISA	180 days
G. Tüter et al., 2010 (33)	Turkey	21	45.33 years	21	7	0	0	Intensive treatment	hs-CRP	Latex-enhanced nephelometry	42 days
Y. Shimada et al., 2010 (34)	Japan	33	55.1 years	33	25	11	0	Standard treatment	CRP, TNF〈, IL-6	ELISA	30 days
O. Fentoğlu et al., 2010 (35)	Turkey	20	51.85 years	20	12	0	0	Intensive treatment	hs-CRP	Latex-enhanced nephelometry	90 days
F. Graziani et al., 2010 (36)	Italy	19	48.9 years	19	8	NA	0	Intensive treatment	CRP	ELISA	90 days 180 days
S. J. Lin et al., 2012 (37)	Taiwan	14	59 years	14	9	0	14	Intensive treatment	IL-6, CRP	ELISA	90 days 180 days
P.A. Koromantzoset al., 2012 (38)	Greece	53	Standard treatment: 59.42 years Intensive treatment: 59.62 years	53	Standard treatment (n= 14) Intensive treatment (n=13)	Standard treatment (n= 7) Intensive treatment (n=4)	Standard treatment (n= 27) Intensive treatment (n=26)	Standard treatment (n= 27) Intensive treatment (n=26)	hs-CRP	PCR	30 days 90 days 180 days
W. Kamil et al., 2011 (39)	Jordan	18	46.7 years	18	8	0	0	Intensive treatment	CRP	PCR	90 days
E.M. Vilela et al., 2011 (40)	Brazil	56	48.28 years	56	22	0	10	Intensive treatment	hs-CRP, IL-6	ELISA (hs-CRP) Latex-enhanced nephelometry (IL-6)	90 days
N.J. Lopez et al., 2012 (41)	Chile	81	56.3 years	81	59	22	NA	Standard treatment	CRP	Immunoturbidimetric assay	90 days 180 days 270 days 365 days
L. Chen et al., 2012 (42)	China	85	Standard treatment: 57.91 years Intensive treatment: 56.86 years	85	Standard treatment (n=17) Intensive treatment (n=19)	Standard treatment (n=10) Intensive treatment (n=7)	Standard treatment (n=43) Intensive treatment (n=42)	Standard treatment (n=43) Intensive treatment (n=42)	hs-CRP TNF〈	ELISA	45 days 90 days 180 days
F. Llambes et al., 2012 (43)	Spain	23	33.8 years	23	11	10	23	Intensive treatment	hs-CRP	Immunoturbidimetric assay	90 days
U. Altay et al., 2013 (44)	Turkey	46	44.05 years	46	32	0	46	Intensive treatment	hs-CRP TNF〈 IL-6	ELISA	90 days
M. Al-Zahrani et al., 2012 (45)	Saudi Arabia	40	35 years	40	40	0	0	Standard treatment	CRP	ELISA	56 days
T. Fiorini et al., 2012 (46)	Brazil	27	>18 years	27	27	6	0	Intensive treatment	TNF〈, IL-1®, IL-6	Flow-cytometry	21 days
S.A.H. Bokhari et al., 2013 (47)	Pakistan	180	49	180	32	80	0	Intensive treatment	CRP	ELISA	30 days (n=180) 60 days (n=161)
V. A. Patil et al., 2013 (48)	India	20	38,5	20	0	0	0	Intensive treatment	CRP	Immunoturbidimetic assay	90 days
P. Koppolu et al., 2013 (49)	India	20	56,13	20	5	0	0	Intensive treatment	hs-CRP, TNF〈	ELISA	56 days
R. Al Habashneh et al., 2014 (50)	Jordan	41	Intensive + ozonetherapy group: 39,7 Intensive group: 39	42	Intensive + ozonetherapy group (n=14) Intensive group (n=14)	0	0	Intensive treatment + ozonetherapy (n=20) Intensive treatment (n=21)	hs-CRP	ELISA	90 days
R. Patabi Cheta Raman et al., 2014 (51)	Malaysia	15	57,7	15	4	0	15	Intensive treatment	hs-CRP	ELISA	90 days
A.L. Caula et al., 2014 (52)	Brazil	32	44,4	32	12	4	5	Intensive treatment	CRP	Immunoturbidimetic assay	60 days 180 days
F.Fang et al., 2015 (53)	China	48	53,71	48	20	6	0	Intensive treatment	TNF〈, IL-6, hs-CRP	Immunoturbidimetic assay (hs-CRP) Immunoabsorbent assay (TNF〈, IL-6)	42 days 90 days 180 days
Carillo Artese et al., 2015 (54)	Brazil	24	Standard treatment group: 54,4 Intensive treatment group: 52	24	Standard treatment group (n=7) Intensive treatment group (n=6)	0	24	Standard treatment (n=12) Intensive treatment (n=12)	TNF〈, IL-6	ELISA	180 days
Y. Munenaga et al., 2013 (55)	Japan	80	Intensive group A: 67,18 Intensive group B: 66,21	80	Intensive group A (n=23) Intensive group B (n=19)	NA	Intensive group A (n=33) Intensive group B (n=47)	Intensive treatment group A (n=33) Intensive treatment group B (n=47)	hs-CRP	latex-enhanced_nephelometry	30 days
N. Markou et al., 2023 (56)	Greece	60	Intensive group: 49,5 Intensive group + Laser 3W: 49,3 Intensive group + Laser 2W: 50,9	60	Intensive group (n=7) Intensive group + Laser 3W (n=5) Intensive group + Laser 2W (n=7)	Intensive group (n=10) Intensive group + Laser 3W (n=10) Intensive group + Laser 2W (n=10)	NA	Intensive treatment (n=20) Intensive treatment + Laser 3W (n=20) Intensive treatment + Laser 2W (n=20)	IL-1®, IL-6, CRP	ELISA	42 days, 90 days, 180 days, 360 days
G. Isola et al., 2023 (57)	Italy	46	Intensive FMD group: 50,3 Q-SRP group: 51,16	46	Intensive FMD group (n=13) Q-SRP group (n=12)	Intensive FMD group (n=1) Q-SRP group (n=1)	0	Intensive treatment full mouth disinfection (n=23) Q-SRP Intensive treatment (n=23)	hs-CRP	Nephelometric assay kit	30 days 60 days 180 days
F. Graziani et al., 2023 (58)	Italy	40	Intensive treamene FMD group: 56,89 Q-SRP group 62,45	40	Intensive treamene FMD group (n=7) Q-SRP group (n=8)	Intensive treamene FMD group (n=8) Q-SRP group (n=5)	40	Intensive treatment full mouth disinfection (n=20) Q-SRP Intensive treatment (n=20)	CRP, IL-6	ELISA (IL-6) Immunoturbidimetic assay (CRP)	90 days
B. Shah et al., 2022 (59)	USA	30	38,5	30	19	NA	0	Intensive treatment	hs-CRP	Immunoturbidimetic assay	30 days
F. M. Escobar Arregocés et al., 2021 (60)	Colombia	19	57,6	19	10	0	0	Intensive treatment	IL-1®, IL-6, TNF〈		35 days
M. de Sousa Rabelo et al., 2021 (61)	Brazil	45	Intensive treatment, NG patients: 50,4 Intensive treatment, pre-diabetes patients: 54,1 Intensive treatment, TDM2: 56,1	45	Intensive treatment, NG patients (n=9) Intensive treatment, pre-diabetes patients (n=9) Intensive treatment, TDM2 (n=5)	Intensive treatment, NG patients (n=0) Intensive treatment, pre-diabetes patients (n=0) Intensive treatment, TDM2 (n=0)	Intensive treatment, NG patients (n=0) Intensive treatment, pre-diabetes patients (n=0) Intensive treatment, TDM2 (n=15)	Intensive treatment (n=15) Intensive treatment (n=15) Intensive treatment (n=15)	IL-1®, IL-6, TNF〈	Ultrasensitive multiplex assay	30 days
C. A. Mohammad, 2020 (62)	Iraq	60	36,73	60	19	0	NA	Intensive treatment + curcumin gel (n=30) Intensive treatment (n=30)	IL-1®, TNF〈	Enzyme-linked immunosorbent assay	30 days
W. J. M Lobao et al., 2018 (63)	Brazil	33	41,1	33	20	0	0	Intensive treatment	IL-6, CRP	ELISA	90 days
S. Wang et al., 2017 (64)	China	19	61,58	19	7	6	19	Intensive treatment	IL-6, TNF〈	ELISA	90 days
H. Balci Yuce et al., 2016 (65)	Turkey	18	42,5	18	9	4	0	Intensive treatment	TNF〈	ELISA	42 days
Deepti et al., 2017 (66)	India	26	24	26	26	0	0	Intensive treatment	hs-CRP	Konelab Clinical Chemistry Analyzer	90 days 180 days
G. Zekonis et al., 2016 (67)	Lithuania	34	41,2	34	19	0	0	Intensive treatment + Weekly H2O2 irrigation	hs-CRP	Particle-enhanced turbidimetric assay	365 days 730 days 1095 days
K. Kapellas et al., 2017 (68)	Australia	24	48,5	24	13	12	24	Intensive treatment	hs-CRP, IL-6	Particle-enhanced immunonephelometry (hs-CRP) ELISA (IL-6)	90 days
M. L Geisinger et al., 2016 (69)	USA	240	56,8	240	107	37	240	Intensive treatment	hs-CRP, TNF〈, IL- 6	Latex-particle enhanced immunoturbidimetric assay (hs-CRP) ELISA (TNF〈, IL-6)	180 days
F. Javed et al., 2016 (70)	USA	87	NSPT alone group (PD+CAD): 52,4 NSPT + Laser (PD+CAD) group: 58,2 NSPT alone group (PD): 55,7 NSPT + Laser group (PD): 60	87	0	0	0	Intensive treatment (n=22) Intensive treatment + laser Nd: YAG (n=22) Intensive treatment (n=22) Intensive treatment + Laser Nd: YAG (n=21)	IL-1®	ELISA	90 days
Yong-Wei Fu et al., 2015 (71)	China	109	Standard treatment group: 47,25 FMD group: 46,61	109	Standard treatment group (n=23) FMD group (n=25)	Standard treatment group (n=7) FMD group (n=10)	0	Standard treatment (n=55) Intensive treatment Full Mouth Disinfection protocol (n=54)	TNF〈, IL-1®, IL-6	ELISA	60 days 180 days

### Quality assessment

The methodological quality of the included studies based on the Newcastle–Ottawa scale and the Cochrane Risk of Bias template (RoB 2.0) is described in **Supplementary Table 2** and **Supplementary Figure 1**. Of the 50 studies analyzed, 36 were classified as having a high or unclear risk of bias, mainly due to the lack of double-blinding or issues related to the randomization methods used. However, 14 studies were identified as being at low risk of bias, with participants and/or investigators involved in outcomes assessment being blinded to the patients’ group assignment.

### Description of excluded studies

56 studies did not meet the inclusion criteria and were considered ineligible. Briefly, 18 studies were excluded due to lack of cytokines assessment (72–90), while data were unavailable for 13 studies (91–103). In 11 studies, periodontal therapy involved surgical procedures or antibiotic use (104–114), and in two studies, patients presented with gingivitis or peri-implantitis (115, 116). Lastly, 11 of the 56 excluded studies were either study protocols or lacked full-text availability (117–126). For detailed information see **Supplementary Table 3**.

### Comparative effects of interventions for the primary outcome

A total of 216 observations from 64 observations in the standard treatment group and 152 observations in the intensive treatment group were included in the meta-analysis, comprising 14,374 paired pre- and post-treatment data points.

### Standard treatment

**Figure 2:**
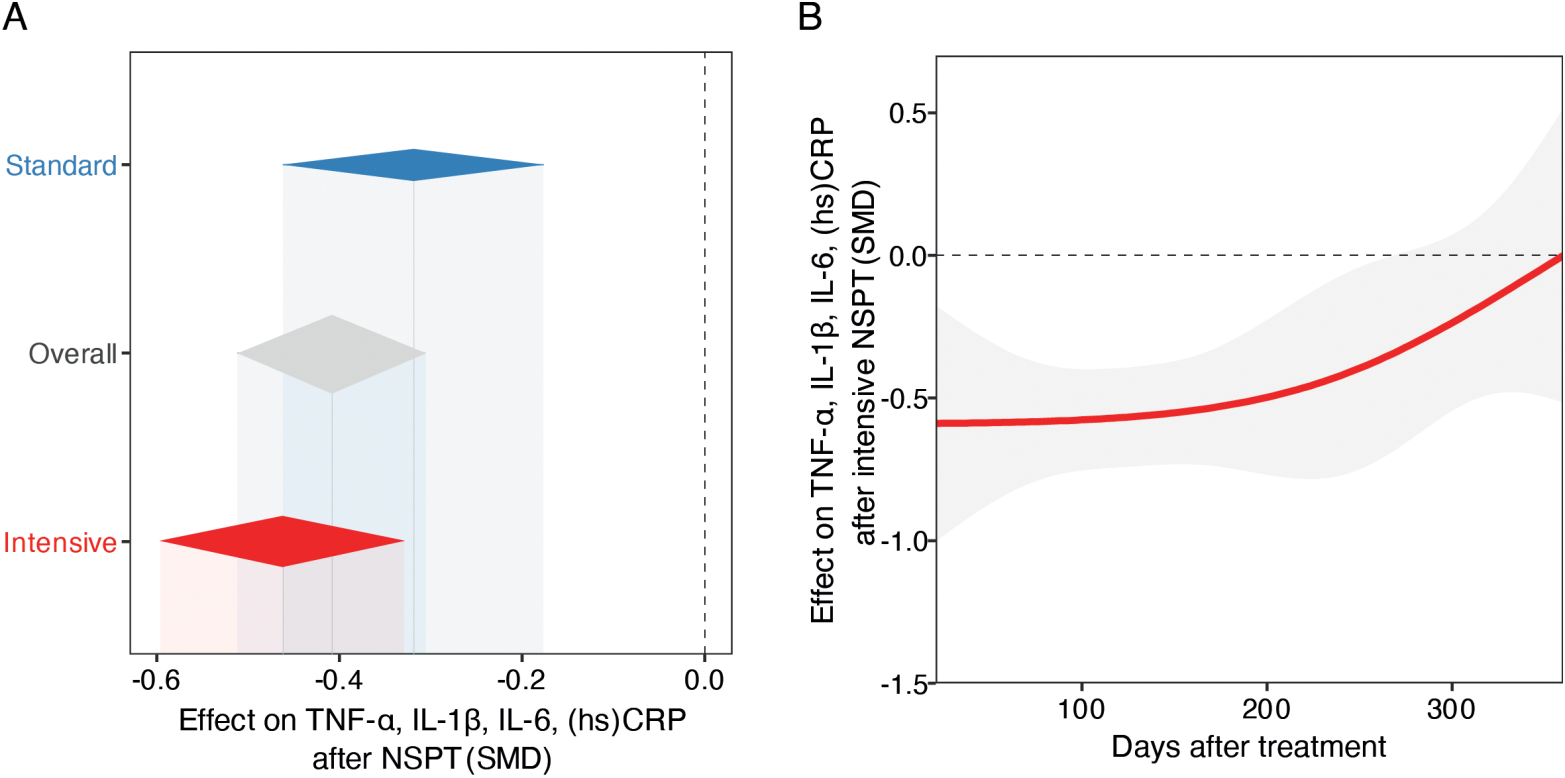
Meta-analysis of periodontal therapy effects on systemic inflammation and temporal treatment dynamics. **(A)** Forest plot of pooled SMDs and 95% CIs for early-phase inflammatory markers level changes following standard or intensive NSPT, using random-effects models. Diamonds represent pooled SMDs; the dashed vertical line indicates no effect (SMD = 0). **(B)** Temporal trend in cytokine reduction post-intensive NSPT, modeled with mixed-effects meta-regression using restricted cubic splines. The red line shows estimated SMD change over time; the shaded area indicates 95% CI. The analysis suggests an early anti-inflammatory response, with potential attenuation or rebound over time.

In the standard treatment subgroup (k = 64), the pooled SMD between post- and pre-treatment cytokine levels was statistically significant. Under the common-effect model, the SMD was –0.2573 (95% CI: –0.3199 to –0.1946; z = –8.05; p < 0.0001), indicating a small but consistent reduction in inflammatory markers following standard periodontal therapy. The random-effects model yielded a slightly larger effect size (SMD = –0.3192; 95% CI: –0.4621 to –0.1764; z = –4.38; p < 0.0001). Between-study heterogeneity was substantial (I² = 78.8%, 95% CI: 73.2% to 83.1%; τ² = 0.2443), suggesting variability in treatment effects across studies (**Figure 2A**).

### Intensive treatment

The intensive treatment subgroup (k = 152) demonstrated a more pronounced reduction in cytokine levels. The common-effect model estimated an SMD of –0.2575 (95% CI: –0.2980 to –0.2170; z = –12.46; p < 0.0001), while the random-effects model showed a larger effect size of –0.4623 (95% CI: –0.5962 to –0.3283; z = –6.76; p < 0.0001). This heterogeneity may reflect differences in the biological roles and regulatory dynamics of the inflammatory markers investigated, as well as potential batch effects related to clinical procedures or sample processing (**Figure 2A**).

### Subgroup comparison

A stratified meta-analysis comparing standard and intensive treatments was conducted (k = 216). The overall random-effects model yielded an SMD of –0.4090 (95% CI: –0.5120 to –0.3060; z = –7.78; p < 0.0001), with substantial heterogeneity (I² = 88.2%). Subgroup analysis revealed a non-significant difference in effect size between the two treatment modalities (Q = 2.05, df = 1, p = 0.1523), although the point estimate favored the intensive treatment. The test for subgroup differences under the fixed-effect model was similarly non-significant (Q = 0.00, p = 0.9958), suggesting that, while both approaches are associated with reductions in systemic inflammatory markers, the superiority of intensive therapy remains inconclusive (**Figure 2A**).

### Temporal dynamics of the overall anti-inflammatory effect of NSPT

To further explore whether the time elapsed between treatment and follow-up assessment influenced the magnitude of effect in the intensive group, we conducted a mixed-effects meta-regression using restricted cubic splines (df = 3). This model, based on 152 observations, showed high residual heterogeneity (τ² = 0.6085, I² = 90.1%) and did not significantly reduce unexplained variance (R² = 0.0%). The overall test for the spline terms was not statistically significant (QM = 4.23, df = 3, p = 0.2372), indicating no strong evidence for a nonlinear relationship between elapsed time and treatment effect. Nonetheless, one spline term reached nominal statistical significance (estimate = 0.5835, p = 0.048), suggesting a possible late-phase attenuation of effect or rebound, although the clinical relevance remains to be confirmed. The intercept remained significantly negative (–0.5889, p = 0.0053), consistent with an overall reduction in inflammatory markers post-treatment. These findings suggest that while periodontal treatment exerts a measurable anti-inflammatory effect, its temporal dynamics across the first months post-intervention are not clearly delineated by the current evidence base (**Figure 2B**).

### Effect on single inflammatory markers

**Figure 3:**
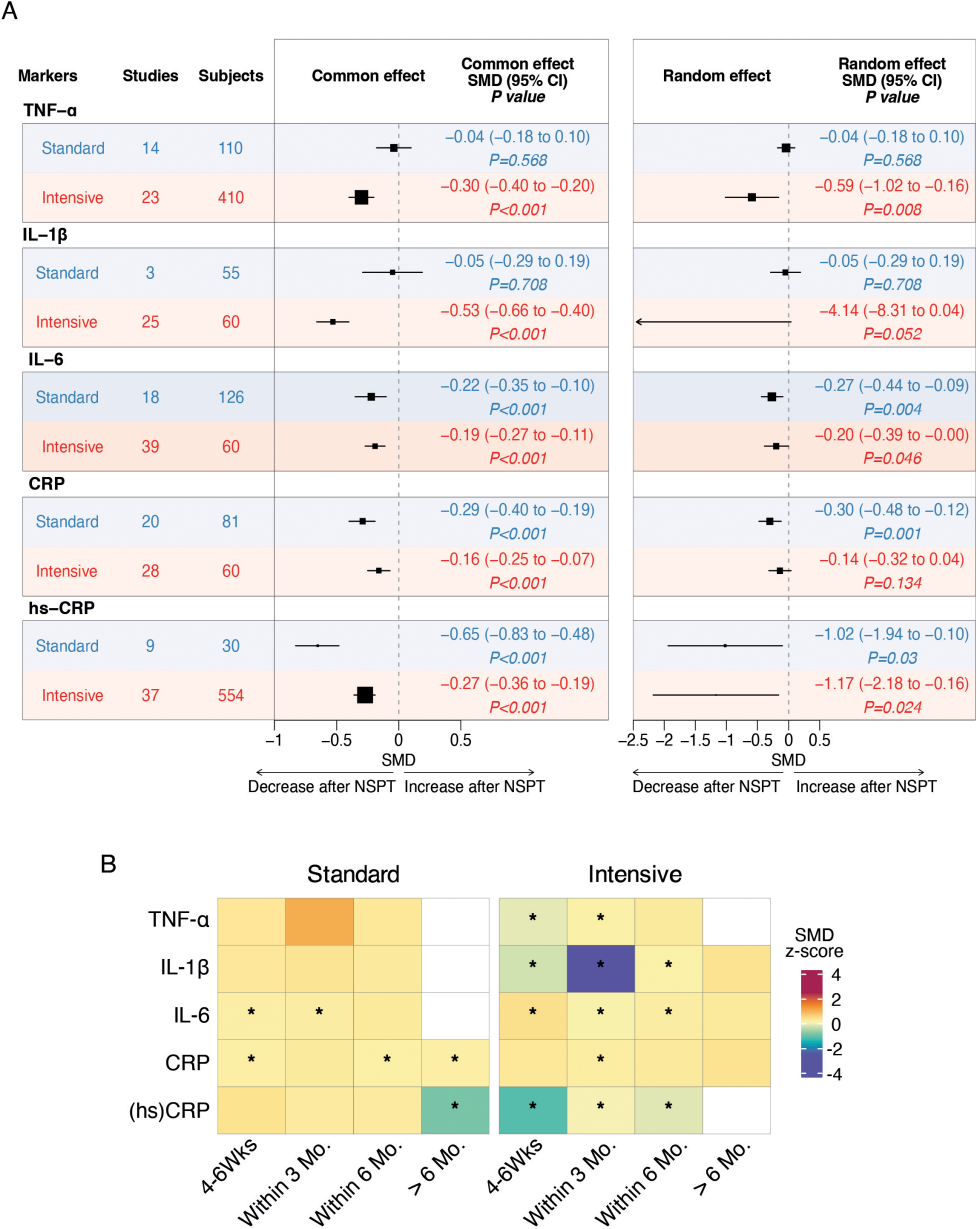
Forest plots and heatmaps summarizing the effects of standard and intensive nonsurgical periodontal therapy on systemic inflammatory markers. **(A)** Forest plots of meta-analyses evaluating the impact of standard and intensive treatment on serum levels of TNF-α, IL-1β, IL-6, CRP, and high-sensitivity CRP. Pooled standardized mean differences with 95% confidence intervals are shown for post-treatment changes, stratified by treatment intensity. Fixed-effect model results are presented on the left, and random-effects model results are shown on the right. Negative values indicate a decrease in inflammatory marker levels following treatment. The vertical dashed line represents no effect (SMD = 0). Statistical significance is indicated by P values. **(B)** Heatmaps showing subgroup meta-analysis results for the temporal effects of standard and intensive treatment on inflammatory markers. Each cell represents the SMD (z-score) for a specific follow-up interval (4–6 weeks, within 3 months, within 6 months, and >6 months). Color intensity reflects the magnitude and direction of change, with cooler colors indicating greater reductions. Asterisks (*) indicate statistically significant changes (P < 0.05) based on the common-effect model.

Results of the meta-analyses indicated a significant post-treatment reduction in serum levels of TNFα (random effect SMD -0.59, 95% CI -1.02 to -0.16; *P*=0.008) in favor of the intensive NSPT group (**Figure 3**). A similar trend was observed for IL-1β (random effect SMD -4.14, 95% CI -8.31 to -0.04; *P*=0.052) (**Figure 3**). In addition, we found a significant post-treatment reduction in serum levels of IL-6 following both the intensive (random effect SMD -0.20, 95% CI -0.39 to -0.00; *P*=0.046) and the standard (random effect SMD -0.27, 95% CI -0.44 to -0-09; *P*=0.004) NSPT (**Figure 3**). Heterogeneity among the included studies was substantial in the intensive group (I² = 81%, τ² = 0.2995, P < 0.01), whereas was moderate (I² = 43%, τ² = 0.0567, P = 0.03) in the standard group.

### Secondary outcomes

A significant post-treatment reduction in serum levels of hs-CRP following both the intensive (random effect SMD -1.17, 95% CI -2.18 to -0.16; *P*=0.024) and the standard (random effect SMD -1.02, 95% CI -1.94 to -0-10; *P*=0.030) NSPT resulted from the meta-analysis (**Figure 3A**). Heterogeneity among the included studies was considerable in both groups: in the intensive group (I² = 94%, τ² = 0.6291, P < 0.01) and in the standard group (I² = 94%, τ² = 1.9142, P < 0.01).

As to CRP levels, a significant post-treatment reduction was found in favor of the standard NSPT (random effect SMD -0.30, 95% CI -0.48 to -0.12; *P*=0.001) (**Figure 3A**).

Heterogeneity among the included studies was moderate in the intensive group (I² = 68%, τ² = 0.1590, P < 0.01), and similarly moderate in the standard group (I² = 58%, τ² = 0.0879, P < 0.01). Temporal subgroup analysis revealed that reductions in inflammatory markers varied across follow-up intervals. In particular, significant short-term reductions (within 6 months) were consistently observed for IL-6, CRP, and hs-CRP in both treatment strategies. For intensive treatment, IL-1β and TNF-α also showed significant reductions within the first 3 months, highlighting a potential early anti-inflammatory benefit (**Figure 3B**).

### Meta-regression

A meta-regression was performed with different moderators (i.e., elapsed time to assessment of change, study year, mean age, female sex ratio, number of smokers, number of teeth before treatment, diabetes ratio).

*TNFα*. Within the intensive NSPT group, meta-regression indicated that only mean age and study year had a significant effect on SMD (R^2^: 37.74, Slope: 0.097, *P*=0.004; and R^2^: 17.10, Slope: −0.144, *P*=0.045, respectively), with younger individuals showing greater decrease, and more recent studies capturing larger reductions, in TNFα levels (**Supplementary File 1**).

*IL-1β*. Within the intensive NSPT group, meta-regression showed only a borderline effect of smoke on SMD (R^2^: 24.56, Slope: 0.976, *P*=0.049) (**Supplementary File 1**).

*IL-6*. Meta-regression indicated that the number of teeth before treatment had a significant effect on SMD in individuals receiving intensive NSPT (R^2^: 100, Slope: 0.156, *P*=0.001), indicating larger decrease in IL-6 with lower number of teeth (**Supplementary File 1**), while mean age affected the outcome in the standard treatment group (R^2^: 100, Slope: -0.007, *P*=0.013), with older individuals showing greater reduction in IL-6 (**Supplementary File 1**).

*Hs-CRP*. Within the intensive NSPT group, meta-regression indicated that only study year had a significant effect on SMD (R^2^: 88.37, Slope: −0.302, *P*=0.000), with more recent studies capturing larger reductions in hs-CRP levels (**Supplementary File 1**). As to the standard treated participants, younger age (*P*=0.0), longer elapsed time to assessment (*P*=0.0), more recent study year (*P*=0.0), greater number of teeth (*P*=0.0), higher female sex ratio (*P*=0.048), lower diabetes ratio (*P*=0.005) and lower number of smokers in the studies (*P*=0.002) significantly impacted on larger reductions in hs-CRP levels (**Supplementary File 1**).

*CRP.* Meta-regression indicated no effect of the examined moderators on SMD in the standard NSPT group (**Supplementary File 1**).

### Stratified analyses

Stratified analyses were conducted for each outcome within the relevant treatment group(s), provided that at least two studies were available within each stratification, as specified in the methods.

### Elapsed time to change assessment

*TNFα*. Significant reductions in TNFα levels in the intensive NSPT group occurred as early as within 3 months of treatment and lost significance thereafter. No significant changes in TNFα levels were detected following standard NSPT at any timepoint (**Supplementary File 1**).

*IL-1β*. Reductions in IL-1β levels with intensive NSPT were no longer appreciated after 6 months following treatment. Stratification was not possible for standard NSPT (**Supplementary File 1**).

*IL-6*. Irrespective of treatment group, significant reductions in IL-6 occurred between 6 weeks to 3 months after treatment (**Supplementary File 1**). Interestingly, a trend to an early rise in IL-6 was observed 4 to 6 weeks following intensive NSPT.

*Hs-CRP*. Significant reductions in hs-CRP levels occurred between 6 weeks to 3 months of treatment in the intensive NSPT group, and after 6 months in the standard treatment group (**Supplementary File 1**).

*CRP.* Reductions in CRP levels were more evident between 3 to 6 months, especially in the standard treatment group (**Supplementary File 1**).

### Single sex studies

*IL-1β*. Among intensive-treated participants, male-specific studies showed significant post-treatment reductions in IL-1β levels (**Supplementary File 1**). Only one study assessing IL-1β changes following intensive NSPT was female-specific, and the effect was neutral.

No sex-specific studies were conducted on standard NSPT (**Supplementary File 1**).

*IL-6*. Based on the findings from two female-specific studies, post-treatment increases in IL-6 levels were observed in intensive-treated females (**Supplementary File 1**). No male-specific studies assessing IL-6 changes were available.

*Hs-CRP*. Among intensive-treated participants, female-specific studies uniquely showed post-treatment increases in hs-CRP levels (**Supplementary File 1**). No male-specific studies assessing hs-CRP changes were available. No sex-specific studies were conducted on hs-CRP changes following standard NSPT.

### Smoking habits

*TNFα and IL-1β*. Based on the findings from intensive NSPT, post-treatment reductions in TNFα and IL-1β were particularly evident in non-smokers (**Supplementary File 1**) Only one study assessing TNFα and IL-1β changes after standard NSPT was conducted in non-smokers.

*IL-6*. Post-treatment reductions in IL-6 were particularly evident among smokers, irrespective of treatment modality (**Supplementary File 1**).

*Hs-CRP and CRP.* Within the intensive NSPT group, reductions in hs-CRP and CRP levels were more evident among smokers (**Supplementary File 1**); among standard-treated individuals, they were more pronounced in non-smokers (**Supplementary File 1**).

### Diabetes status

*TNFα and IL-1β*. Based on the findings from intensive NSPT, post-treatment reductions in TNFα were particularly evident in non-diabetic individuals (**Supplementary File 1**). Only one study assessing IL-1β changes was conducted on diabetic individuals.

*IL-6*. Within the standard NSPT group, reductions in IL-6 levels were observed in both diabetic and non-diabetic participants (**Supplementary File 1**).

*Hs-CRP and CRP*. Reductions in hs-CRP and CRP levels were observed in standard-treated non-diabetic participants (**Supplementary File 1**).

### Full mouth disinfection (intensive NSPT group)

All the examined inflammatory markers, except for CRP, were significantly reduced with FMD (**Supplementary Files 1**). No changes were observed with laser treatments and curcumin gel use (data not shown).

## Discussion

This meta-analysis provides evidence on the effectiveness of NSPT in modulating systemic inflammation. Intensive NSPT demonstrated a more pronounced effect than the standard NSPT, with no strong evidence of a temporal relationship between treatment and anti-inflammatory effect. Specifically, intensive NSPT proved more effective in reducing key inflammatory markers such as TNF-α, IL-1β and hs-CRP, while standard treatment showed favorable outcomes in the reduction of IL-6 and hs-CRP. Meta-regression analyses indicate that individual factors such as age, smoking habits, residual dentition, and the presence of comorbidities like diabetes significantly influence therapeutic outcomes.

Our meta-analysis particularly focused on specific cytokines and acute phase proteins involved in acute systemic inflammation. Among these, TNF-α plays a central role in the inflammatory cascade: it acts upstream by stimulating the production of downstream mediators such as IL-1β, IL-6 and CRP and, in synergy with IL-1β, contributes to the acute phases of the inflammatory response (127). The clinical relevance of IL-1β is further supported by therapeutic strategies targeting it. Inhibition of the inflammasome pathway involving IL-1β has been associated with reduced cardiovascular events in the CANTOS trial, underscoring its critical role in regulating systemic inflammation (128, 129).

Consistently, chronic periodontitis is associated with elevated levels of systemic inflammatory markers and an increase in circulating neutrophils. The early and intense activation of these pathways contributes both to local tissue destruction and to the systemic spread of inflammation, supporting the hypothesis that periodontitis may represent a chronic and often underdiagnosed infectious condition associated with systemic diseases (130, 131).

Based on this premise, our findings reinforce the hypothesis that periodontal therapy may have measurable systemic effects. In particular, improving oral health through NSPT appears not only as a local intervention but also as a potentially effective complementary strategy for managing chronic systemic inflammatory conditions. This interpretation aligns with the contemporary view of periodontitis as a chronic immunoinflammatory disease not confined to the oral cavity, but capable of significantly influencing systemic homeostasis (132).

Non-surgical periodontal approaches have also shown favorable effects on blood pressure profiles in hypertensive patients, with evidence indicating that a 30% reduction in gingival bleeding following NSPT is potentially associated with a decrease of approximately 11 mmHg in systolic blood pressure (133). This improvement is thought to result from a reduction in the oral biofilm burden, leading to decreased circulating levels of pro-inflammatory cytokines (TNF-α, IL-1, IL-6, CRP) and subsequent stabilization of systemic inflammation. Indeed, a recent meta-analysis proposed NSPT as a potential non-pharmacological intervention for blood pressure control, following the cardiovascular benefits observed in both hypertensive and pre-hypertensive individuals (134).

Similarly, periodontal therapy has been shown to improve glycemic control, significantly reducing HbA1c levels in patients with type 2 diabetes, thus confirming the bidirectional relationship between periodontal disease and diabetes (135). In line with this, our meta-analysis observed a reduction in IL-6 levels in both diabetic and non-diabetic individuals undergoing standard treatment. However, the reduction in TNF-α and hs-CRP/CRP was more pronounced in non-diabetic patients treated with intensive and standard therapy, respectively, suggesting that the patient’s metabolic status may influence the extent of the systemic inflammatory response to periodontal therapy.

Smoking also emerged as a determining factor in terms of response to NSPT: non-smokers experienced greater reductions in TNF-α and IL-1β following intensive treatment, while smokers showed a more significant reduction in IL-6, regardless of the therapeutic approach. These differences may be attributed to the immunomodulatory effects of smoking: some studies (136, 137) have shown that substances found in tobacco, such as nicotine, catechol, and hydroquinone, inhibit the production of pro-inflammatory cytokines like IL-1β, IL-2, IFN-γ, and TNF-α; others have reported increased systemic levels of IL-6 and CRP in female smokers (138, 139). These findings suggest that baseline inflammatory profiles, differing between smokers and non-smokers, may variably modulate cytokine responses to periodontal therapy, thus contributing to the heterogeneity in clinical outcomes.

Stratified time analyses further revealed a distinct kinetic profile in the systemic inflammatory response based on treatment intensity. Intensive NSPT led to a rapid and marked reduction of TNF-α, IL-6, and hs-CRP, with the lowest levels observed within the first three months after treatment. The observed reduction in IL-1β occurred within six months after treatment but was not sustained in the long term. Altogether, there findings suggest a time-dependent modulation of early inflammatory cytokines. In contrast, standard therapy showed a more gradual effect, with noticeable reductions in hs-CRP only after six months and significant CRP decreases between three and six months. These findings highlight the importance of the temporal component in the efficacy of periodontal therapy: while intensive treatment yields rapid biochemical responses, standard treatment proves equally effective in the long-term modulation of systemic inflammatory markers. Although the pooled comparison between treatment modalities did not reveal statistical significance for all markers, stratified analyses uncovered clinically meaningful distinctions in the timing and magnitude of the inflammatory response. Accordingly, the efficacy of periodontal therapy may depend not only on intensity, but also on individual patient characteristics. Such findings underscore the importance of moving toward personalized therapeutic strategies in periodontology, tailored to the patient’s systemic condition and risk factors.

Some sex-specific trends emerged in the modulation of inflammatory markers following periodontal treatment, with preliminary indications of more favorable responses in women. However, due to limited number of sex-specific studies these findings must be interpreted with caution. As such, the current evidence remains insufficient to support firm conclusions regarding sex-based differences in the systemic response to NSPT. Nonetheless, these preliminary observations point a potential role of sexual dimorphism in the inflammatory response, which should be further explored through well-powered, sex-stratified studies. This need is further supported by growing evidence of immunological dimorphism between sexes (140). Women generally exhibit stronger innate and adaptive immune responses compared to men, as evidenced by elevated expression of pro-inflammatory cytokines, increased activation of inflammatory T cells, and a heightened overall inflammatory state (141). In addition, we have recently shown a female-specific association between active periodontal inflammation and metabolic syndrome, a cluster of metabolic risk factors underpinned by low-grade systemic inflammation, with higher CRP levels unique to women with both conditions (142), which is relevant in the perspective of gender and precision medicine.

To the best of our knowledge, this is the first meta-analysis specifically evaluating the effect of NSPT on circulating inflammatory cytokines and their temporal dynamics. However, our study is not without limitations. Firstly, the reduced number of sex-specific studies limits the possibility of drawing any definite conclusions on sex-related differences in the inflammatory response, and this topic deserves investigation in future, dedicated research. While this meta-analysis aimed to distinguish between intensive and standard NSPT, we acknowledge the variability across studies in terms of instrumentation, number and type of sessions (e.g., FMD vs. quadrant-based scaling and root planning) and the use of adjunctive procedures represents a limitation. Notably, only a minority of studies explicitly reported using FMD or Q-SRP protocols, limiting the possibility of formal meta-regression on these variables. In addition, many of the included studies exhibited moderate-high risk of bias, particularly due to limitations in study design and reporting. This, combined with high heterogeneity and possible publication bias, warrants caution in interpreting the results. Most importantly, the duration of follow-up in the included studies was generally limited to 6-months. Future trials should therefore prioritize extended follow-up to clarify the durability of NSPT’s systemic anti-inflammatory effects. While our analysis revealed a rebound effect a few months after treatment, the long-term trajectory of that effect remains unknown. Finally, even though the majority of the included studies employed ELISA assays, variability in the specific testing methods used introduces a source of potential heterogeneity across the findings. These limitations highlight the urgent need for future high-quality randomized controlled trials that adopt standardized NSPT protocols, include comprehensive procedural descriptions, and account for relevant patient characteristics. Furthermore, extended follow-up durations and integrated evaluations of both periodontal and systemic outcomes will be essential to clarify the long-term impact of NSPT on systemic inflammation and support its role as a component of broader interdisciplinary care.

## Conclusion

In summary, our findings support the notion that NSPT can have a significant impact on the systemic inflammatory burden, with potentially important implications for the management of conditions such as diabetes and other chronic inflammation-related disorders. However, the variability in clinical response based on individual factors such as age, sex, diabetes status, and smoking underscore the need for personalized therapeutic approaches to optimize the benefits of periodontal treatment in specific and high-risk populations.

## Data Availability

The raw data supporting the conclusions of this article will be made available by the authors, without undue reservation.
