# Supplementary material for: Temporal dynamics of early inflammatory markers after professional dental cleaning: a meta-analysis and spline-based meta-regression of TNF-α, IL-1β, IL-6, and (hs)CRP
Source: Front Immunol. 2025 Aug 28;16:1634622. doi: 10.3389/fimmu.2025.1634622 (PMC12423065; doi:10.3389/fimmu.2025.1634622)
Supplement: Supplementary file 1 [file DataSheet1.zip › Supplementary materials/PDF/CRP_Intensive_results.pdf]

Cytokine: CRP − Treatment: Intensive

Study	After TRT Total	After TRT Mean	After TRT SD	Before TRT Total	Before TRT Mean	Before TRT SD	SMD	95%−CI	Weight (common)	Weight (random)
**Days after treatment:30**										
Tonetti 2018	61	31.00	5.0000	61	25.00	27.0000	0.31	[−0.05; 0.66]	6.2%	4.5%
Bokhari SAH 2013	180	34.10	27.6000	180	43.50	33.5000	−0.31	[−0.51; −0.10]	18.2%	5.1%
**Common effect model**	**241**			**241**			**−0.15**	**[−0.33; 0.03]**	**24.3%**	**.**
**Random effects model**							**−0.02**	**[−0.62; 0.58]**	**.**	**9.6%**
Heterogeneity: I^2 = 88%, τ^2 = 0.1655, p < 0.01										
**Days after treatment:42**										
Nikolaos Markou 2023	20	16.64	10.3980	20	14.43	10.2750	0.21	[−0.41; 0.83]	2.0%	3.3%
Nikolaos Markou 2023	20	15.17	7.9510	20	18.23	10.7640	−0.32	[−0.94; 0.31]	2.0%	3.3%
Nikolaos Markou 2023	20	13.33	9.2990	20	12.36	7.9520	0.11	[−0.51; 0.73]	2.0%	3.4%
**Common effect model**	**60**			**60**			**0.00**	**[−0.36; 0.36]**	**6.1%**	**.**
**Random effects model**							**0.00**	**[−0.36; 0.36]**	**.**	**10.0%**
Heterogeneity: I^2 = 0%, τ^2 = 0, p = 0.46										
**Days after treatment:60**										
Tonetti 2018	61	30.00	8.0000	61	25.00	27.0000	0.25	[−0.11; 0.61]	6.2%	4.5%
Bokhari SAH 2013	161	30.70	30.5000	161	43.50	33.5000	−0.40	[−0.62; −0.18]	16.1%	5.1%
Caula AL 2014	32	8.70	2.3000	32	9.60	3.9000	−0.28	[−0.77; 0.21]	3.2%	3.9%
**Common effect model**	**254**			**254**			**−0.23**	**[−0.40; −0.05]**	**25.5%**	**.**
**Random effects model**							**−0.15**	**[−0.56; 0.26]**	**.**	**13.5%**
Heterogeneity: I^2 = 78%, τ^2 = 0.0972, p < 0.01										
**Days after treatment:90**										
Graziani F 2010	19	21.00	2.4000	19	22.70	4.3000	−0.48	[−1.12; 0.17]	1.9%	3.2%
Shih−Jung Lin 2011	14	0.00	0.0002	14	0.00	0.0001	0.00	[−0.74; 0.74]	1.4%	2.9%
Kamil W 2011	18	180.00	60.0000	18	230.00	70.0000	−0.75	[−1.43; −0.07]	1.7%	3.1%
Veena A Patil 2013	20	0.01	0.0035	20	0.02	0.0047	−2.81	[−3.71; −1.92]	1.0%	2.4%
Nikolaos Markou 2023	20	15.78	13.2110	20	14.43	10.2750	0.11	[−0.51; 0.73]	2.0%	3.4%
Nikolaos Markou 2023	20	17.98	9.2970	20	18.23	10.7640	−0.02	[−0.64; 0.60]	2.0%	3.4%
Nikolaos Markou 2023	20	16.39	9.1750	20	12.36	7.9520	0.46	[−0.17; 1.09]	2.0%	3.3%
Filippo Graziani 2022	20	36.70	57.3000	20	33.20	61.8000	0.06	[−0.56; 0.68]	2.0%	3.4%
Filippo Graziani 2022	20	39.80	61.6000	20	42.00	44.8000	−0.04	[−0.66; 0.58]	2.0%	3.4%
Lobao 2018	33	2.40	3.4000	33	2.70	3.3000	−0.09	[−0.57; 0.39]	3.4%	4.0%
**Common effect model**	**204**			**204**			**−0.21**	**[−0.41; −0.01]**	**19.5%**	**.**
**Random effects model**							**−0.32**	**[−0.84; 0.20]**	**.**	**32.3%**
Heterogeneity: I^2 = 79%, τ^2 = 0.5888, p < 0.01										
**Days after treatment:180**										
Tonetti 2018	61	25.00	7.0000	61	25.00	27.0000	0.00	[−0.35; 0.35]	6.2%	4.5%
Graziani F 2010	19	21.20	2.7000	19	22.70	4.3000	−0.41	[−1.05; 0.23]	1.9%	3.3%
Shih−Jung Lin 2011	14	0.00	0.0002	14	0.00	0.0001	0.34	[−0.41; 1.08]	1.4%	2.9%
Caula AL 2014	32	5.40	3.6000	32	9.60	3.9000	−1.11	[−1.63; −0.58]	2.8%	3.8%
Nikolaos Markou 2023	20	16.51	12.5990	20	14.43	10.2750	0.18	[−0.44; 0.80]	2.0%	3.4%
Nikolaos Markou 2023	20	16.64	9.0520	20	18.23	10.7640	−0.16	[−0.78; 0.46]	2.0%	3.4%
Nikolaos Markou 2023	20	13.70	7.5840	20	12.36	7.9520	0.17	[−0.45; 0.79]	2.0%	3.4%
**Common effect model**	**186**			**186**			**−0.16**	**[−0.37; 0.04]**	**18.4%**	**.**
**Random effects model**							**−0.16**	**[−0.53; 0.21]**	**.**	**24.5%**
Heterogeneity: I^2 = 66%, τ^2 = 0.1607, p < 0.01										
**Days after treatment:360**										
Nikolaos Markou 2023	20	17.62	12.9660	20	14.43	10.2750	0.27	[−0.36; 0.89]	2.0%	3.3%
Nikolaos Markou 2023	20	19.82	12.4780	20	18.23	10.7640	0.13	[−0.49; 0.75]	2.0%	3.4%
Nikolaos Markou 2023	20	11.62	7.5840	20	12.36	7.9520	−0.09	[−0.71; 0.53]	2.0%	3.4%
**Common effect model**	**60**			**60**			**0.10**	**[−0.26; 0.46]**	**6.1%**	**.**
**Random effects model**							**0.10**	**[−0.26; 0.46]**	**.**	**10.1%**
Heterogeneity: I^2 = 0%, τ^2 = 0, p = 0.72										
**Common effect model**	**1005**			**1005**			**−0.16**	**[−0.25; −0.07]**	**100.0%**	**.**
**Random effects model**							**−0.14**	**[−0.32; 0.04]**	**.**	**100.0%**

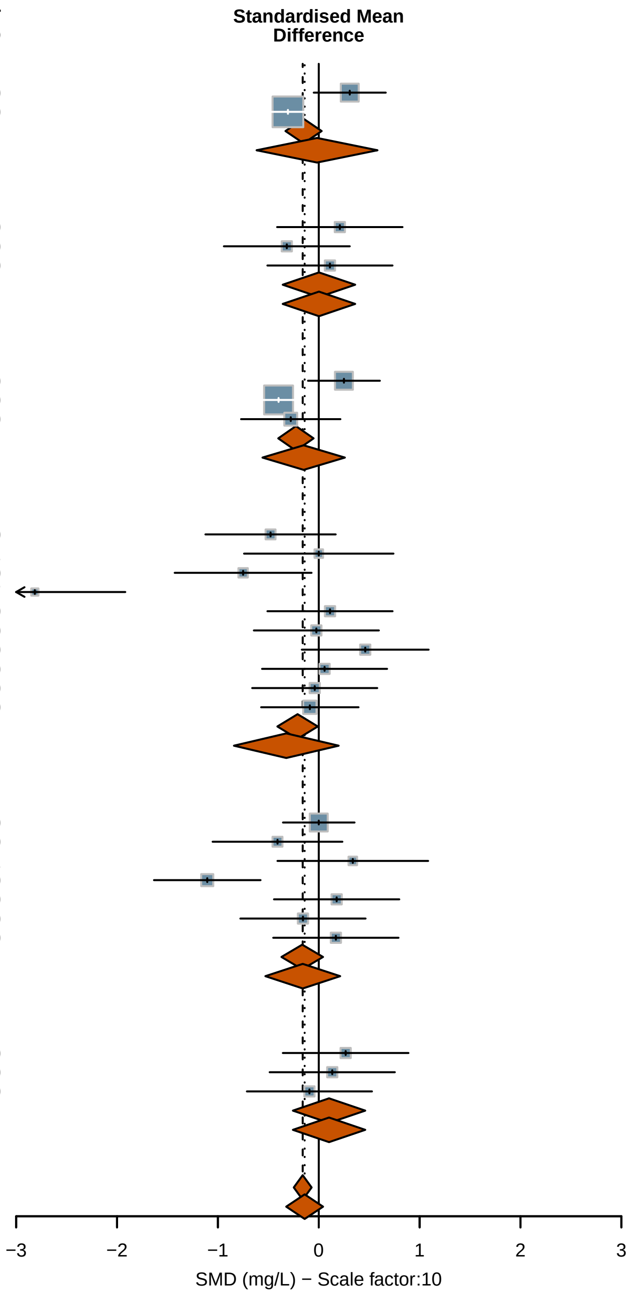

Heterogeneity: I^2 = 68%, τ^2 = 0.1590, p < 0.01
Test for subgroup differences (common effect): χ^2_5 = 3.63, df = 5 (p = 0.60)
Test for subgroup differences (random effects): χ^2_5 = 2.34, df = 5 (p = 0.80)

SMD: −0.16; 95%C.I.[−0.25; −0.07] P value for common effect= 4e−04

SMD: −0.14; 95%C.I.[−0.32; 0.04] P value for random effect= 0.1335

Cytokine: CRP − Treatment: Intensive

Study	After TRT Total	After TRT Mean	After TRT SD	Before TRT Total	Before TRT Mean	Before TRT SD	SMD	95%−CI	Weight (common)	Weight (random)
**MonthsAfterTRT:4−6Wks**										
Tonetti 2018	61	31.00	5.0000	61	25.00	27.0000	0.31	[−0.05; 0.66]	6.2%	4.5%
Bokhari SAH 2013	180	34.10	27.6000	180	43.50	33.5000	−0.31	[−0.51; −0.10]	18.2%	5.1%
Nikolaos Markou 2023	20	16.64	10.3980	20	14.43	10.2750	0.21	[−0.41; 0.83]	2.0%	3.3%
Nikolaos Markou 2023	20	15.17	7.9510	20	18.23	10.7640	−0.32	[−0.94; 0.31]	2.0%	3.3%
Nikolaos Markou 2023	20	13.33	9.2990	20	12.36	7.9520	0.11	[−0.51; 0.73]	2.0%	3.4%
**Common effect model**	**301**			**301**			**−0.12**	**[−0.28; 0.04]**	**30.4%**	**.**
**Random effects model**							**−0.02**	**[−0.32; 0.28]**	**.**	**19.7%**
Heterogeneity: $I^2 = 62\%$, $\tau^2 = 0.0618$, $p = 0.03$										
**MonthsAfterTRT:Within 3 Mo.**										
Tonetti 2018	61	30.00	8.0000	61	25.00	27.0000	0.25	[−0.11; 0.61]	6.2%	4.5%
Graziani F 2010	19	21.00	2.4000	19	22.70	4.3000	−0.48	[−1.12; 0.17]	1.9%	3.2%
Shih−Jung Lin 2011	14	0.00	0.0002	14	0.00	0.0001	0.00	[−0.74; 0.74]	1.4%	2.9%
Kamil W 2011	18	180.00	60.0000	18	230.00	70.0000	−0.75	[−1.43; −0.07]	1.7%	3.1%
Bokhari SAH 2013	161	30.70	30.5000	161	43.50	33.5000	−0.40	[−0.62; −0.18]	16.1%	5.1%
Veena A Patil 2013	20	0.01	0.0035	20	0.02	0.0047	−2.81	[−3.71; −1.92]	1.0%	2.4%
Caula AL 2014	32	8.70	2.3000	32	9.60	3.9000	−0.28	[−0.77; 0.21]	3.2%	3.9%
Nikolaos Markou 2023	20	15.78	13.2110	20	14.43	10.2750	0.11	[−0.51; 0.73]	2.0%	3.4%
Nikolaos Markou 2023	20	17.98	9.2970	20	18.23	10.7640	−0.02	[−0.64; 0.60]	2.0%	3.4%
Nikolaos Markou 2023	20	16.39	9.1750	20	12.36	7.9520	0.46	[−0.17; 1.09]	2.0%	3.3%
Filippo Graziani 2022	20	36.70	57.3000	20	33.20	61.8000	0.06	[−0.56; 0.68]	2.0%	3.4%
Filippo Graziani 2022	20	39.80	61.6000	20	42.00	44.8000	−0.04	[−0.66; 0.58]	2.0%	3.4%
Lobao 2018	33	2.40	3.4000	33	2.70	3.3000	−0.09	[−0.57; 0.39]	3.4%	4.0%
**Common effect model**	**458**			**458**			**−0.22**	**[−0.35; −0.09]**	**45.0%**	**.**
**Random effects model**							**−0.27**	**[−0.64; 0.11]**	**.**	**45.8%**
Heterogeneity: $I^2 = 77\%$, $\tau^2 = 0.3886$, $p < 0.01$										
**MonthsAfterTRT:Within 6 Mo.**										
Tonetti 2018	61	25.00	7.0000	61	25.00	27.0000	0.00	[−0.35; 0.35]	6.2%	4.5%
Graziani F 2010	19	21.20	2.7000	19	22.70	4.3000	−0.41	[−1.05; 0.23]	1.9%	3.3%
Shih−Jung Lin 2011	14	0.00	0.0002	14	0.00	0.0001	0.34	[−0.41; 1.08]	1.4%	2.9%
Caula AL 2014	32	5.40	3.6000	32	9.60	3.9000	−1.11	[−1.63; −0.58]	2.8%	3.8%
Nikolaos Markou 2023	20	16.51	12.5990	20	14.43	10.2750	0.18	[−0.44; 0.80]	2.0%	3.4%
Nikolaos Markou 2023	20	16.64	9.0520	20	18.23	10.7640	−0.16	[−0.78; 0.46]	2.0%	3.4%
Nikolaos Markou 2023	20	13.70	7.5840	20	12.36	7.9520	0.17	[−0.45; 0.79]	2.0%	3.4%
**Common effect model**	**186**			**186**			**−0.16**	**[−0.37; 0.04]**	**18.4%**	**.**
**Random effects model**							**−0.16**	**[−0.53; 0.21]**	**.**	**24.5%**
Heterogeneity: $I^2 = 66\%$, $\tau^2 = 0.1607$, $p < 0.01$										
**MonthsAfterTRT:6Mo. and more**										
Nikolaos Markou 2023	20	17.62	12.9660	20	14.43	10.2750	0.27	[−0.36; 0.89]	2.0%	3.3%
Nikolaos Markou 2023	20	19.82	12.4780	20	18.23	10.7640	0.13	[−0.49; 0.75]	2.0%	3.4%
Nikolaos Markou 2023	20	11.62	7.5840	20	12.36	7.9520	−0.09	[−0.71; 0.53]	2.0%	3.4%
**Common effect model**	**60**			**60**			**0.10**	**[−0.26; 0.46]**	**6.1%**	**.**
**Random effects model**							**0.10**	**[−0.26; 0.46]**	**.**	**10.1%**
Heterogeneity: $I^2 = 0\%$, $\tau^2 = 0$, $p = 0.72$										
**Common effect model**	**1005**			**1005**			**−0.16**	**[−0.25; −0.07]**	**100.0%**	**.**
**Random effects model**							**−0.14**	**[−0.32; 0.04]**	**.**	**100.0%**

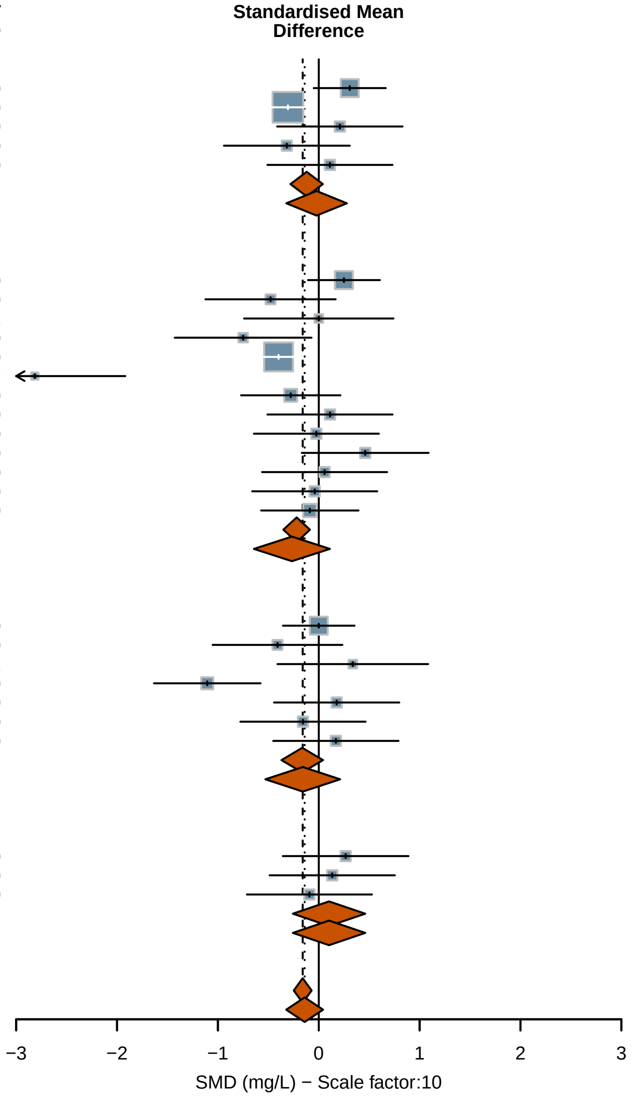

Heterogeneity: $I^2 = 68\%$, $\tau^2 = 0.1590$, $p < 0.01$
Test for subgroup differences (common effect): $\chi^2_3 = 3.06$, df = 3 ($p = 0.38$)
Test for subgroup differences (random effects): $\chi^2_3 = 2.24$, df = 3 ($p = 0.52$)

SMD: −0.16; 95%C.I.[−0.25; −0.07] P value for common effect= 4e−04

SMD: −0.14; 95%C.I.[−0.32; 0.04] P value for random effect= 0.1335

Cytokine: CRP − Treatment: Intensive

Study	After TRT Total	After TRT Mean	After TRT SD	Before TRT Total	Before TRT Mean	Before TRT SD	SMD	95%−CI	Weight (common)	Weight (random)
**Assay type:ELISA**										
Graziani F 2010	19	21.00	2.4000	19	22.70	4.3000	−0.48	[−1.12; 0.17]	1.9%	3.2%
Graziani F 2010	19	21.20	2.7000	19	22.70	4.3000	−0.41	[−1.05; 0.23]	1.9%	3.3%
Shih−Jung Lin 2011	14	0.00	0.0002	14	0.00	0.0001	0.00	[−0.74; 0.74]	1.4%	2.9%
Shih−Jung Lin 2011	14	0.00	0.0002	14	0.00	0.0001	0.34	[−0.41; 1.08]	1.4%	2.9%
Bokhari SAH 2013	180	34.10	27.6000	180	43.50	33.5000	−0.31	[−0.51; −0.10]	18.2%	5.1%
Bokhari SAH 2013	161	30.70	30.5000	161	43.50	33.5000	−0.40	[−0.62; −0.18]	16.1%	5.1%
Nikolaos Markou 2023	20	16.64	10.3980	20	14.43	10.2750	0.21	[−0.41; 0.83]	2.0%	3.3%
Nikolaos Markou 2023	20	15.78	13.2110	20	14.43	10.2750	0.11	[−0.51; 0.73]	2.0%	3.4%
Nikolaos Markou 2023	20	16.51	12.5990	20	14.43	10.2750	0.18	[−0.44; 0.80]	2.0%	3.4%
Nikolaos Markou 2023	20	17.62	12.9660	20	14.43	10.2750	0.27	[−0.36; 0.89]	2.0%	3.3%
Nikolaos Markou 2023	20	15.17	7.9510	20	18.23	10.7640	−0.32	[−0.94; 0.31]	2.0%	3.3%
Nikolaos Markou 2023	20	17.98	9.2970	20	18.23	10.7640	−0.02	[−0.64; 0.60]	2.0%	3.4%
Nikolaos Markou 2023	20	16.64	9.0520	20	18.23	10.7640	−0.16	[−0.78; 0.46]	2.0%	3.4%
Nikolaos Markou 2023	20	19.82	12.4780	20	18.23	10.7640	0.13	[−0.49; 0.75]	2.0%	3.4%
Nikolaos Markou 2023	20	13.33	9.2990	20	12.36	7.9520	0.11	[−0.51; 0.73]	2.0%	3.4%
Nikolaos Markou 2023	20	16.39	9.1750	20	12.36	7.9520	0.46	[−0.17; 1.09]	2.0%	3.3%
Nikolaos Markou 2023	20	13.70	7.5840	20	12.36	7.9520	0.17	[−0.45; 0.79]	2.0%	3.4%
Nikolaos Markou 2023	20	11.62	7.5840	20	12.36	7.9520	−0.09	[−0.71; 0.53]	2.0%	3.4%
Lobao 2018	33	2.40	3.4000	33	2.70	3.3000	−0.09	[−0.57; 0.39]	3.4%	4.0%
**Common effect model**	**680**			**680**			**−0.17**	**[−0.27; −0.06]**	**68.6%**	**.**
**Random effects model**							**−0.09**	**[−0.23; 0.06]**	**.**	**66.6%**
Heterogeneity: $I^2 = 17\%$, $\tau^2 = 0.0259$, $p = 0.24$										
**Assay type:immunoturbidimetic assay**										
Tonetti 2018	61	31.00	5.0000	61	25.00	27.0000	0.31	[−0.05; 0.66]	6.2%	4.5%
Tonetti 2018	61	30.00	8.0000	61	25.00	27.0000	0.25	[−0.11; 0.61]	6.2%	4.5%
Tonetti 2018	61	25.00	7.0000	61	25.00	27.0000	0.00	[−0.35; 0.35]	6.2%	4.5%
Veena A Patil 2013	20	0.01	0.0035	20	0.02	0.0047	−2.81	[−3.71; −1.92]	1.0%	2.4%
Caula AL 2014	32	8.70	2.3000	32	9.60	3.9000	−0.28	[−0.77; 0.21]	3.2%	3.9%
Caula AL 2014	32	5.40	3.6000	32	9.60	3.9000	−1.11	[−1.63; −0.58]	2.8%	3.8%
**Common effect model**	**267**			**267**			**−0.13**	**[−0.30; 0.05]**	**25.6%**	**.**
**Random effects model**							**−0.56**	**[−1.46; 0.34]**	**.**	**23.6%**
Heterogeneity: $I^2 = 91\%$, $\tau^2 = 1.1974$, $p < 0.01$										
**Assay type:immunoturbidimetry**										
Filippo Graziani 2022	20	36.70	57.3000	20	33.20	61.8000	0.06	[−0.56; 0.68]	2.0%	3.4%
Filippo Graziani 2022	20	39.80	61.6000	20	42.00	44.8000	−0.04	[−0.66; 0.58]	2.0%	3.4%
**Common effect model**	**40**			**40**			**0.01**	**[−0.43; 0.45]**	**4.1%**	**.**
**Random effects model**							**0.01**	**[−0.43; 0.45]**	**.**	**6.7%**
Heterogeneity: $I^2 = 0\%$, $\tau^2 = 0$, $p = 0.83$										
**Assay type:PCR**										
Kamil W 2011	18	180.00	60.0000	18	230.00	70.0000	−0.75	[−1.43; −0.07]	1.7%	3.1%
**Common effect model**	**1005**			**1005**			**−0.16**	**[−0.25; −0.07]**	**100.0%**	**.**
**Random effects model**							**−0.14**	**[−0.32; 0.04]**	**.**	**100.0%**

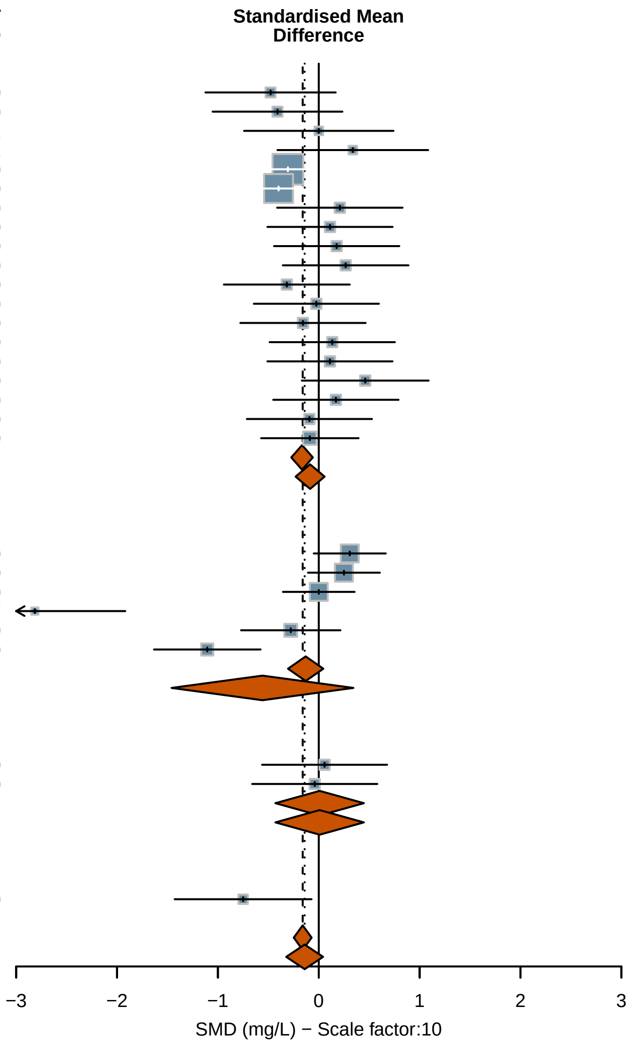

Heterogeneity: $I^2 = 68\%$, $\tau^2 = 0.1590$, $p < 0.01$
Test for subgroup differences (common effect): $\chi^2_3 = 3.60$, df = 3 ($p = 0.31$)
Test for subgroup differences (random effects): $\chi^2_3 = 4.75$, df = 3 ($p = 0.19$)

SMD: −0.16; 95%C.I.[−0.25; −0.07] P value for common effect= 4e−04

SMD: −0.14; 95%C.I.[−0.32; 0.04] P value for random effect= 0.1335

Cytokine: CRP − Treatment: Intensive

Study	After TRT Total	After TRT Mean	After TRT SD	Before TRT Total	Before TRT Mean	Before TRT SD	SMD	95%−CI	Weight (common)	Weight (random)
**Bias judgment:High risk**										
Nikolaos Markou 2023	20	16.64	10.3980	20	14.43	10.2750	0.21	[−0.41; 0.83]	2.0%	3.3%
Nikolaos Markou 2023	20	15.78	13.2110	20	14.43	10.2750	0.11	[−0.51; 0.73]	2.0%	3.4%
Nikolaos Markou 2023	20	16.51	12.5990	20	14.43	10.2750	0.18	[−0.44; 0.80]	2.0%	3.4%
Nikolaos Markou 2023	20	17.62	12.9660	20	14.43	10.2750	0.27	[−0.36; 0.89]	2.0%	3.3%
Nikolaos Markou 2023	20	15.17	7.9510	20	18.23	10.7640	−0.32	[−0.94; 0.31]	2.0%	3.3%
Nikolaos Markou 2023	20	17.98	9.2970	20	18.23	10.7640	−0.02	[−0.64; 0.60]	2.0%	3.4%
Nikolaos Markou 2023	20	16.64	9.0520	20	18.23	10.7640	−0.16	[−0.78; 0.46]	2.0%	3.4%
Nikolaos Markou 2023	20	19.82	12.4780	20	18.23	10.7640	0.13	[−0.49; 0.75]	2.0%	3.4%
Nikolaos Markou 2023	20	13.33	9.2990	20	12.36	7.9520	0.11	[−0.51; 0.73]	2.0%	3.4%
Nikolaos Markou 2023	20	16.39	9.1750	20	12.36	7.9520	0.46	[−0.17; 1.09]	2.0%	3.3%
Nikolaos Markou 2023	20	13.70	7.5840	20	12.36	7.9520	0.17	[−0.45; 0.79]	2.0%	3.4%
Nikolaos Markou 2023	20	11.62	7.5840	20	12.36	7.9520	−0.09	[−0.71; 0.53]	2.0%	3.4%
Lobao 2018	33	2.40	3.4000	33	2.70	3.3000	−0.09	[−0.57; 0.39]	3.4%	4.0%
**Common effect model**	**273**			**273**			**0.07**	**[−0.10; 0.23]**	**27.7%**	**.**
**Random effects model**							**0.07**	**[−0.10; 0.23]**	**.**	**44.1%**
Heterogeneity: I^2 = 0%, τ^2 = 0, p = 0.95										
**Bias judgment:Low risk**										
Tonetti 2018	61	31.00	5.0000	61	25.00	27.0000	0.31	[−0.05; 0.66]	6.2%	4.5%
Tonetti 2018	61	30.00	8.0000	61	25.00	27.0000	0.25	[−0.11; 0.61]	6.2%	4.5%
Tonetti 2018	61	25.00	7.0000	61	25.00	27.0000	0.00	[−0.35; 0.35]	6.2%	4.5%
Graziani F 2010	19	21.00	2.4000	19	22.70	4.3000	−0.48	[−1.12; 0.17]	1.9%	3.2%
Graziani F 2010	19	21.20	2.7000	19	22.70	4.3000	−0.41	[−1.05; 0.23]	1.9%	3.3%
Shih−Jung Lin 2011	14	0.00	0.0002	14	0.00	0.0001	0.00	[−0.74; 0.74]	1.4%	2.9%
Shih−Jung Lin 2011	14	0.00	0.0002	14	0.00	0.0001	0.34	[−0.41; 1.08]	1.4%	2.9%
Bokhari SAH 2013	180	34.10	27.6000	180	43.50	33.5000	−0.31	[−0.51; −0.10]	18.2%	5.1%
Bokhari SAH 2013	161	30.70	30.5000	161	43.50	33.5000	−0.40	[−0.62; −0.18]	16.1%	5.1%
Veena A Patil 2013	20	0.01	0.0035	20	0.02	0.0047	−2.81	[−3.71; −1.92]	1.0%	2.4%
**Common effect model**	**610**			**610**			**−0.21**	**[−0.32; −0.09]**	**60.4%**	**.**
**Random effects model**							**−0.31**	**[−0.80; 0.19]**	**.**	**38.4%**
Heterogeneity: I^2 = 84%, τ^2 = 0.5526, p < 0.01										
**Bias judgment:Some concerns**										
Kamil W 2011	18	180.00	60.0000	18	230.00	70.0000	−0.75	[−1.43; −0.07]	1.7%	3.1%
Caula AL 2014	32	8.70	2.3000	32	9.60	3.9000	−0.28	[−0.77; 0.21]	3.2%	3.9%
Caula AL 2014	32	5.40	3.6000	32	9.60	3.9000	−1.11	[−1.63; −0.58]	2.8%	3.8%
Filippo Graziani 2022	20	36.70	57.3000	20	33.20	61.8000	0.06	[−0.56; 0.68]	2.0%	3.4%
Filippo Graziani 2022	20	39.80	61.6000	20	42.00	44.8000	−0.04	[−0.66; 0.58]	2.0%	3.4%
**Common effect model**	**122**			**122**			**−0.44**	**[−0.70; −0.19]**	**11.8%**	**.**
**Random effects model**							**−0.43**	**[−0.87; 0.01]**	**.**	**17.5%**
Heterogeneity: I^2 = 65%, τ^2 = 0.1594, p = 0.02										
**Common effect model**	**1005**			**1005**			**−0.16**	**[−0.25; −0.07]**	**100.0%**	**.**
**Random effects model**							**−0.14**	**[−0.32; 0.04]**	**.**	**100.0%**

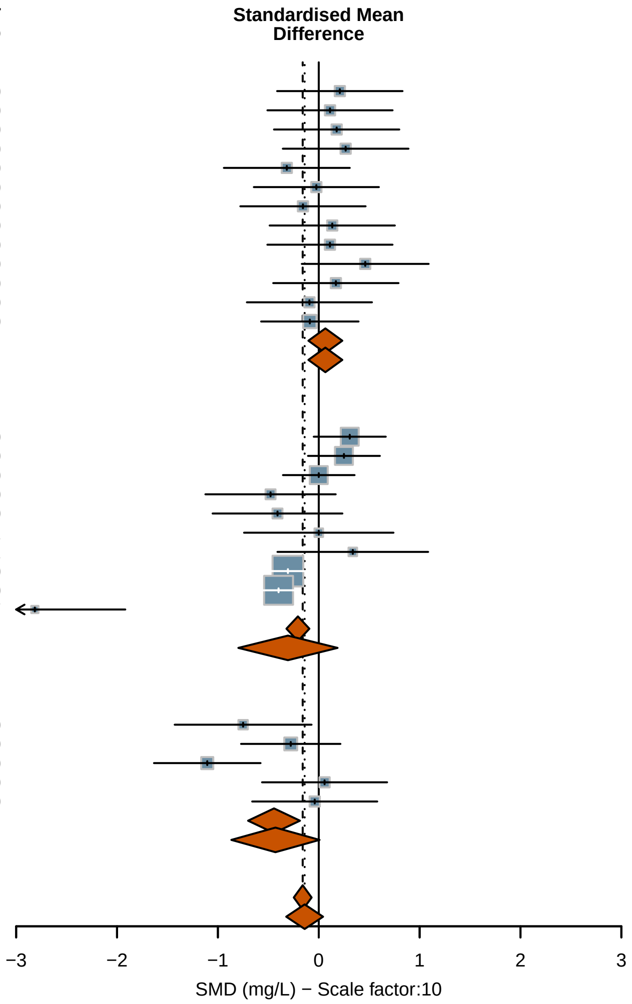

Heterogeneity: I^2 = 68%, τ^2 = 0.1590, p < 0.01
Test for subgroup differences (common effect): χ^2_2 = 12.21, df = 2 (p < 0.01)
Test for subgroup differences (random effects): χ^2_2 = 5.65, df = 2 (p = 0.06)

SMD: −0.16; 95%C.I.[−0.25; −0.07] P value for common effect= 4e−04

SMD: −0.14; 95%C.I.[−0.32; 0.04] P value for random effect= 0.1335

Cytokine: CRP − Treatment: Intensive

Study	Total (After TRT)	Mean (After TRT)	SD (After TRT)	Total (Before TRT)	Mean (Before TRT)	SD (Before TRT)	SMD	95%−CI	Weight (common)	Weight (random)
**Study design:Clinical trial**										
Lobao 2018	33	2.40	3.4000	33	2.70	3.3000	−0.09	[−0.57; 0.39]	3.4%	4.0%
**Study design:Prospective exploratory monocentric trial**										
Graziani F 2010	19	21.00	2.4000	19	22.70	4.3000	−0.48	[−1.12; 0.17]	1.9%	3.2%
Graziani F 2010	19	21.20	2.7000	19	22.70	4.3000	−0.41	[−1.05; 0.23]	1.9%	3.3%
**Common effect model**	**38**			**38**			**−0.44**	**[−0.90; 0.01]**	**3.8%**	**.**
**Random effects model**							**−0.44**	**[−0.90; 0.01]**	**.**	**6.5%**
Heterogeneity: $I^2 = 0\%$, $\tau^2 = 0$, $p = 0.88$										
**Study design:RCT**										
Tonetti 2018	61	31.00	5.0000	61	25.00	27.0000	0.31	[−0.05; 0.66]	6.2%	4.5%
Tonetti 2018	61	30.00	8.0000	61	25.00	27.0000	0.25	[−0.11; 0.61]	6.2%	4.5%
Tonetti 2018	61	25.00	7.0000	61	25.00	27.0000	0.00	[−0.35; 0.35]	6.2%	4.5%
Shih−Jung Lin 2011	14	0.00	0.0002	14	0.00	0.0001	0.00	[−0.74; 0.74]	1.4%	2.9%
Shih−Jung Lin 2011	14	0.00	0.0002	14	0.00	0.0001	0.34	[−0.41; 1.08]	1.4%	2.9%
Kamil W 2011	18	180.00	60.0000	18	230.00	70.0000	−0.75	[−1.43; −0.07]	1.7%	3.1%
Bokhari SAH 2013	180	34.10	27.6000	180	43.50	33.5000	−0.31	[−0.51; −0.10]	18.2%	5.1%
Bokhari SAH 2013	161	30.70	30.5000	161	43.50	33.5000	−0.40	[−0.62; −0.18]	16.1%	5.1%
Caula AL 2014	32	8.70	2.3000	32	9.60	3.9000	−0.28	[−0.77; 0.21]	3.2%	3.9%
Caula AL 2014	32	5.40	3.6000	32	9.60	3.9000	−1.11	[−1.63; −0.58]	2.8%	3.8%
Nikolaos Markou 2023	20	16.64	10.3980	20	14.43	10.2750	0.21	[−0.41; 0.83]	2.0%	3.3%
Nikolaos Markou 2023	20	15.78	13.2110	20	14.43	10.2750	0.11	[−0.51; 0.73]	2.0%	3.4%
Nikolaos Markou 2023	20	16.51	12.5990	20	14.43	10.2750	0.18	[−0.44; 0.80]	2.0%	3.4%
Nikolaos Markou 2023	20	17.62	12.9660	20	14.43	10.2750	0.27	[−0.36; 0.89]	2.0%	3.3%
Nikolaos Markou 2023	20	15.17	7.9510	20	18.23	10.7640	−0.32	[−0.94; 0.31]	2.0%	3.3%
Nikolaos Markou 2023	20	17.98	9.2970	20	18.23	10.7640	−0.02	[−0.64; 0.60]	2.0%	3.4%
Nikolaos Markou 2023	20	16.64	9.0520	20	18.23	10.7640	−0.16	[−0.78; 0.46]	2.0%	3.4%
Nikolaos Markou 2023	20	19.82	12.4780	20	18.23	10.7640	0.13	[−0.49; 0.75]	2.0%	3.4%
Nikolaos Markou 2023	20	13.33	9.2990	20	12.36	7.9520	0.11	[−0.51; 0.73]	2.0%	3.4%
Nikolaos Markou 2023	20	16.39	9.1750	20	12.36	7.9520	0.46	[−0.17; 1.09]	2.0%	3.3%
Nikolaos Markou 2023	20	13.70	7.5840	20	12.36	7.9520	0.17	[−0.45; 0.79]	2.0%	3.4%
Nikolaos Markou 2023	20	11.62	7.5840	20	12.36	7.9520	−0.09	[−0.71; 0.53]	2.0%	3.4%
Filippo Graziani 2022	20	36.70	57.3000	20	33.20	61.8000	0.06	[−0.56; 0.68]	2.0%	3.4%
Filippo Graziani 2022	20	39.80	61.6000	20	42.00	44.8000	−0.04	[−0.66; 0.58]	2.0%	3.4%
**Common effect model**	**914**			**914**			**−0.12**	**[−0.21; −0.03]**	**91.9%**	**.**
**Random effects model**							**−0.06**	**[−0.21; 0.09]**	**.**	**87.2%**
Heterogeneity: $I^2 = 52\%$, $\tau^2 = 0.0621$, $p < 0.01$										
**Study design:Single−center Interventional study**										
Veena A Patil 2013	20	0.01	0.0035	20	0.02	0.0047	−2.81	[−3.71; −1.92]	1.0%	2.4%
**Common effect model**	**1005**			**1005**			**−0.16**	**[−0.25; −0.07]**	**100.0%**	**.**
**Random effects model**							**−0.14**	**[−0.32; 0.04]**	**.**	**100.0%**

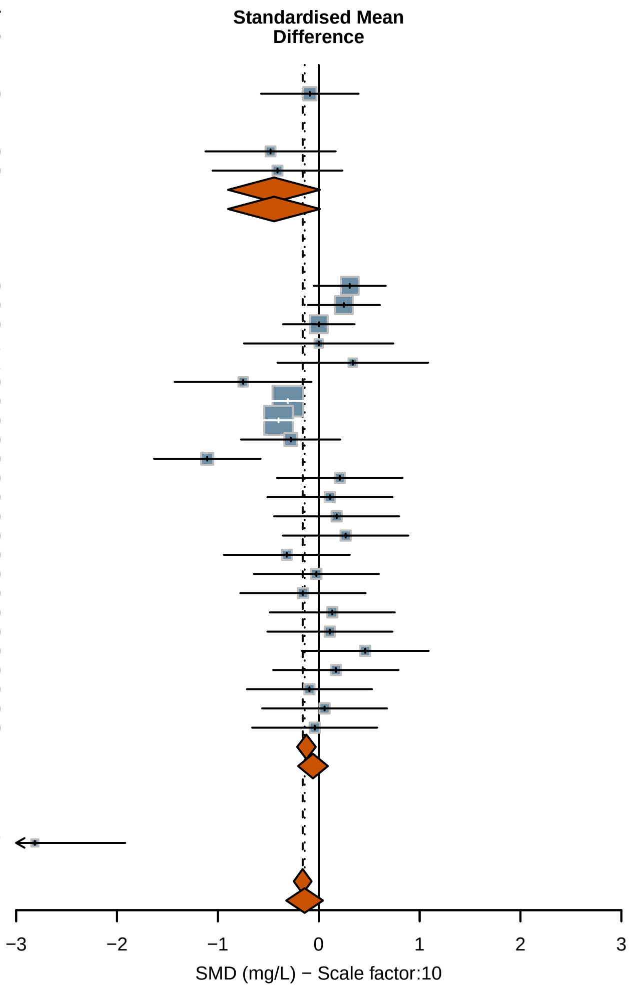

Heterogeneity: $I^2 = 68\%$, $\tau^2 = 0.1590$, $p < 0.01$
Test for subgroup differences (common effect): $\chi^2_3 = 35.90$, df = 3 ($p < 0.01$)
Test for subgroup differences (random effects): $\chi^2_3 = 37.09$, df = 3 ($p < 0.01$)

SMD: −0.16; 95%C.I.[−0.25; −0.07] P value for common effect= 4e−04

SMD: −0.14; 95%C.I.[−0.32; 0.04] P value for random effect= 0.1335

Cytokine: CRP − Treatment: Intensive

Study	After TRT Total	After TRT Mean	After TRT SD	Before TRT Total	Before TRT Mean	Before TRT SD	SMD	95%−CI	Weight (common)	Weight (random)
**Additional_devices:FMD**										
Filippo Graziani 2022	20	36.70	57.3000	20	33.20	61.8000	0.06	[−0.56; 0.68]	10.1%	10.1%
**Additional_devices:laser 2 W, 200 mJ, 10Hz**										
Nikolaos Markou 2023	20	13.33	9.2990	20	12.36	7.9520	0.11	[−0.51; 0.73]	10.0%	10.0%
Nikolaos Markou 2023	20	16.39	9.1750	20	12.36	7.9520	0.46	[−0.17; 1.09]	9.8%	9.8%
Nikolaos Markou 2023	20	13.70	7.5840	20	12.36	7.9520	0.17	[−0.45; 0.79]	10.0%	10.0%
Nikolaos Markou 2023	20	11.62	7.5840	20	12.36	7.9520	−0.09	[−0.71; 0.53]	10.0%	10.0%
**Common effect model**	**80**			**80**			**0.16**	**[−0.15; 0.47]**	**39.9%**	**.**
**Random effects model**							**0.16**	**[−0.15; 0.47]**	**.**	**39.9%**
Heterogeneity: $I^2 = 0\%$, $\tau^2 = 0$, $p = 0.67$										
**Additional_devices:Laser 3 W, 150mJ, 20Hz**										
Nikolaos Markou 2023	20	15.17	7.9510	20	18.23	10.7640	−0.32	[−0.94; 0.31]	9.9%	9.9%
Nikolaos Markou 2023	20	17.98	9.2970	20	18.23	10.7640	−0.02	[−0.64; 0.60]	10.1%	10.1%
Nikolaos Markou 2023	20	16.64	9.0520	20	18.23	10.7640	−0.16	[−0.78; 0.46]	10.0%	10.0%
Nikolaos Markou 2023	20	19.82	12.4780	20	18.23	10.7640	0.13	[−0.49; 0.75]	10.0%	10.0%
**Common effect model**	**80**			**80**			**−0.09**	**[−0.40; 0.22]**	**40.0%**	**.**
**Random effects model**							**−0.09**	**[−0.40; 0.22]**	**.**	**40.0%**
Heterogeneity: $I^2 = 0\%$, $\tau^2 = 0$, $p = 0.78$										
**Additional_devices:QMD**										
Filippo Graziani 2022	20	39.80	61.6000	20	42.00	44.8000	−0.04	[−0.66; 0.58]	10.1%	10.1%
**Common effect model**	**200**			**200**			**0.03**	**[−0.17; 0.23]**	**100.0%**	**.**
**Random effects model**							**0.03**	**[−0.17; 0.23]**	**.**	**100.0%**

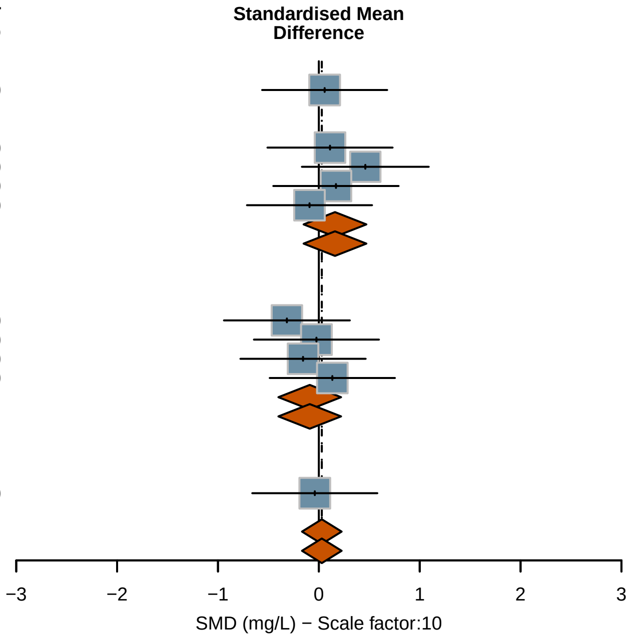

Heterogeneity: $I^2 = 0\%$, $\tau^2 = 0$, $p = 0.92$
Test for subgroup differences (common effect): $\chi^2_3 = 1.30$, df = 3 ($p = 0.73$)
Test for subgroup differences (random effects): $\chi^2_3 = 1.30$, df = 3 ($p = 0.73$)

SMD: 0.03; 95%C.I.[−0.17; 0.23] P value for common effect= 0.7687

SMD: 0.03; 95%C.I.[−0.17; 0.23] P value for random effect= 0.7687

Cytokine: CRP − Treatment: Intensive

Study	After TRT Total	After TRT Mean	After TRT SD	Before TRT Total	Before TRT Mean	Before TRT SD	Standardised Mean Difference	SMD	95%−CI	Weight (common)	Weight (random)
**Female_binary:Sex mixed**											
Tonetti 2018	61	31.00	5.0000	61	25.00	27.0000		0.31	[−0.05; 0.66]	6.2%	4.5%
Tonetti 2018	61	30.00	8.0000	61	25.00	27.0000		0.25	[−0.11; 0.61]	6.2%	4.5%
Tonetti 2018	61	25.00	7.0000	61	25.00	27.0000		0.00	[−0.35; 0.35]	6.2%	4.5%
Graziani F 2010	19	21.00	2.4000	19	22.70	4.3000		−0.48	[−1.12; 0.17]	1.9%	3.2%
Graziani F 2010	19	21.20	2.7000	19	22.70	4.3000		−0.41	[−1.05; 0.23]	1.9%	3.3%
Shih−Jung Lin 2011	14	0.00	0.0002	14	0.00	0.0001		0.00	[−0.74; 0.74]	1.4%	2.9%
Shih−Jung Lin 2011	14	0.00	0.0002	14	0.00	0.0001		0.34	[−0.41; 1.08]	1.4%	2.9%
Kamil W 2011	18	180.00	60.0000	18	230.00	70.0000		−0.75	[−1.43; −0.07]	1.7%	3.1%
Bokhari SAH 2013	180	34.10	27.6000	180	43.50	33.5000		−0.31	[−0.51; −0.10]	18.2%	5.1%
Bokhari SAH 2013	161	30.70	30.5000	161	43.50	33.5000		−0.40	[−0.62; −0.18]	16.1%	5.1%
Veena A Patil 2013	20	0.01	0.0035	20	0.02	0.0047		−2.81	[−3.71; −1.92]	1.0%	2.4%
Caula AL 2014	32	8.70	2.3000	32	9.60	3.9000		−0.28	[−0.77; 0.21]	3.2%	3.9%
Caula AL 2014	32	5.40	3.6000	32	9.60	3.9000		−1.11	[−1.63; −0.58]	2.8%	3.8%
Nikolaos Markou 2023	20	16.64	10.3980	20	14.43	10.2750		0.21	[−0.41; 0.83]	2.0%	3.3%
Nikolaos Markou 2023	20	15.78	13.2110	20	14.43	10.2750		0.11	[−0.51; 0.73]	2.0%	3.4%
Nikolaos Markou 2023	20	16.51	12.5990	20	14.43	10.2750		0.18	[−0.44; 0.80]	2.0%	3.4%
Nikolaos Markou 2023	20	17.62	12.9660	20	14.43	10.2750		0.27	[−0.36; 0.89]	2.0%	3.3%
Nikolaos Markou 2023	20	15.17	7.9510	20	18.23	10.7640		−0.32	[−0.94; 0.31]	2.0%	3.3%
Nikolaos Markou 2023	20	17.98	9.2970	20	18.23	10.7640		−0.02	[−0.64; 0.60]	2.0%	3.4%
Nikolaos Markou 2023	20	16.64	9.0520	20	18.23	10.7640		−0.16	[−0.78; 0.46]	2.0%	3.4%
Nikolaos Markou 2023	20	19.82	12.4780	20	18.23	10.7640		0.13	[−0.49; 0.75]	2.0%	3.4%
Nikolaos Markou 2023	20	13.33	9.2990	20	12.36	7.9520		0.11	[−0.51; 0.73]	2.0%	3.4%
Nikolaos Markou 2023	20	16.39	9.1750	20	12.36	7.9520		0.46	[−0.17; 1.09]	2.0%	3.3%
Nikolaos Markou 2023	20	13.70	7.5840	20	12.36	7.9520		0.17	[−0.45; 0.79]	2.0%	3.4%
Nikolaos Markou 2023	20	11.62	7.5840	20	12.36	7.9520		−0.09	[−0.71; 0.53]	2.0%	3.4%
Filippo Graziani 2022	20	36.70	57.3000	20	33.20	61.8000		0.06	[−0.56; 0.68]	2.0%	3.4%
Filippo Graziani 2022	20	39.80	61.6000	20	42.00	44.8000		−0.04	[−0.66; 0.58]	2.0%	3.4%
Lobao 2018	33	2.40	3.4000	33	2.70	3.3000		−0.09	[−0.57; 0.39]	3.4%	4.0%
**Common effect model**	**1005**			**1005**				**−0.16**	**[−0.25; −0.07]**	**100.0%**	**.**
**Random effects model**								**−0.14**	**[−0.32; 0.04]**	**.**	**100.0%**
Heterogeneity: $I^2 = 68\%$, $\tau^2 = 0.1590$, $p < 0.01$											
**Common effect model**	**1005**			**1005**				**−0.16**	**[−0.25; −0.07]**	**100.0%**	**.**
**Random effects model**								**−0.14**	**[−0.32; 0.04]**	**.**	**100.0%**

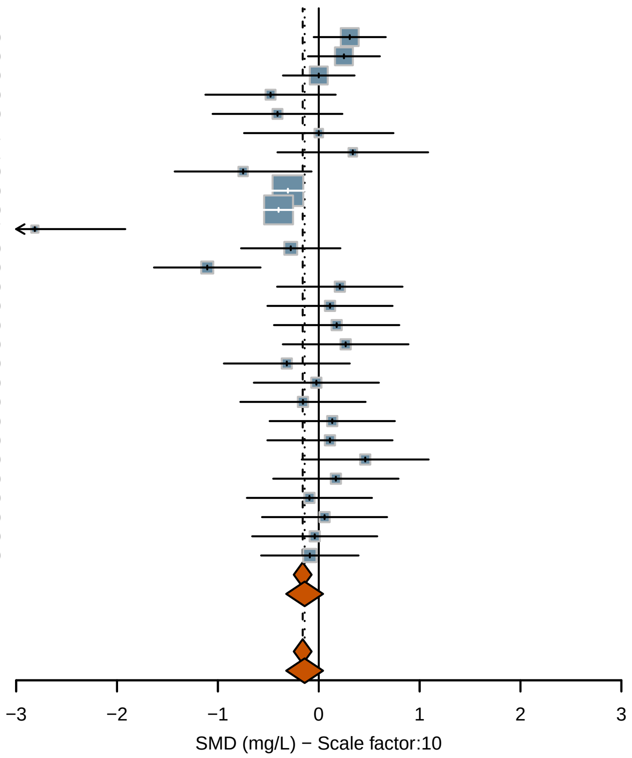

Heterogeneity: $I^2 = 68\%$, $\tau^2 = 0.1590$, $p < 0.01$
Test for subgroup differences (common effect): $\chi^2_0 = 0.00$, df = 0 (p = NA)
Test for subgroup differences (random effects): $\chi^2_0 = 0.00$, df = 0 (p = NA)

SMD: −0.16; 95%C.I.[−0.25; −0.07] P value for common effect= 4e−04

SMD: −0.14; 95%C.I.[−0.32; 0.04] P value for random effect= 0.1335

Cytokine: CRP − Treatment: Intensive

Study	After TRT Total	After TRT Mean	After TRT SD	Before TRT Total	Before TRT Mean	Before TRT SD	SMD	95%−CI	Weight (common)	Weight (random)
**Smoke_binary:Sample with smokers**										
Tonetti 2018	61	31.00	5.0000	61	25.00	27.0000	0.31	[−0.05; 0.66]	6.6%	5.0%
Tonetti 2018	61	30.00	8.0000	61	25.00	27.0000	0.25	[−0.11; 0.61]	6.6%	5.0%
Tonetti 2018	61	25.00	7.0000	61	25.00	27.0000	0.00	[−0.35; 0.35]	6.7%	5.0%
Bokhari SAH 2013	180	34.10	27.6000	180	43.50	33.5000	−0.31	[−0.51; −0.10]	19.5%	5.6%
Bokhari SAH 2013	161	30.70	30.5000	161	43.50	33.5000	−0.40	[−0.62; −0.18]	17.3%	5.5%
Caula AL 2014	32	8.70	2.3000	32	9.60	3.9000	−0.28	[−0.77; 0.21]	3.5%	4.4%
Caula AL 2014	32	5.40	3.6000	32	9.60	3.9000	−1.11	[−1.63; −0.58]	3.0%	4.3%
Nikolaos Markou 2023	20	16.64	10.3980	20	14.43	10.2750	0.21	[−0.41; 0.83]	2.2%	3.9%
Nikolaos Markou 2023	20	15.78	13.2110	20	14.43	10.2750	0.11	[−0.51; 0.73]	2.2%	3.9%
Nikolaos Markou 2023	20	16.51	12.5990	20	14.43	10.2750	0.18	[−0.44; 0.80]	2.2%	3.9%
Nikolaos Markou 2023	20	17.62	12.9660	20	14.43	10.2750	0.27	[−0.36; 0.89]	2.2%	3.9%
Nikolaos Markou 2023	20	15.17	7.9510	20	18.23	10.7640	−0.32	[−0.94; 0.31]	2.2%	3.9%
Nikolaos Markou 2023	20	17.98	9.2970	20	18.23	10.7640	−0.02	[−0.64; 0.60]	2.2%	3.9%
Nikolaos Markou 2023	20	16.64	9.0520	20	18.23	10.7640	−0.16	[−0.78; 0.46]	2.2%	3.9%
Nikolaos Markou 2023	20	19.82	12.4780	20	18.23	10.7640	0.13	[−0.49; 0.75]	2.2%	3.9%
Nikolaos Markou 2023	20	13.33	9.2990	20	12.36	7.9520	0.11	[−0.51; 0.73]	2.2%	3.9%
Nikolaos Markou 2023	20	16.39	9.1750	20	12.36	7.9520	0.46	[−0.17; 1.09]	2.1%	3.8%
Nikolaos Markou 2023	20	13.70	7.5840	20	12.36	7.9520	0.17	[−0.45; 0.79]	2.2%	3.9%
Nikolaos Markou 2023	20	11.62	7.5840	20	12.36	7.9520	−0.09	[−0.71; 0.53]	2.2%	3.9%
Filippo Graziani 2022	20	36.70	57.3000	20	33.20	61.8000	0.06	[−0.56; 0.68]	2.2%	3.9%
Filippo Graziani 2022	20	39.80	61.6000	20	42.00	44.8000	−0.04	[−0.66; 0.58]	2.2%	3.9%
**Common effect model**	**868**			**868**			**−0.12**	**[−0.21; −0.02]**	**93.5%**	**.**
**Random effects model**							**−0.05**	**[−0.20; 0.11]**	**.**	**89.1%**
Heterogeneity: $I^2 = 54\%$, $\tau^2 = 0.0607$, $p < 0.01$										
**Smoke_binary:Sample without smokers**										
Kamil W 2011	18	180.00	60.0000	18	230.00	70.0000	−0.75	[−1.43; −0.07]	1.8%	3.6%
Veena A Patil 2013	20	0.01	0.0035	20	0.02	0.0047	−2.81	[−3.71; −1.92]	1.0%	2.8%
Lobao 2018	33	2.40	3.4000	33	2.70	3.3000	−0.09	[−0.57; 0.39]	3.6%	4.5%
**Common effect model**	**71**			**71**			**−0.71**	**[−1.08; −0.35]**	**6.5%**	**.**
**Random effects model**							**−1.18**	**[−2.77; 0.40]**	**.**	**10.9%**
Heterogeneity: $I^2 = 93\%$, $\tau^2 = 1.8374$, $p < 0.01$										
**Common effect model**	**939**			**939**			**−0.16**	**[−0.25; −0.07]**	**100.0%**	**.**
**Random effects model**							**−0.14**	**[−0.35; 0.07]**	**.**	**100.0%**

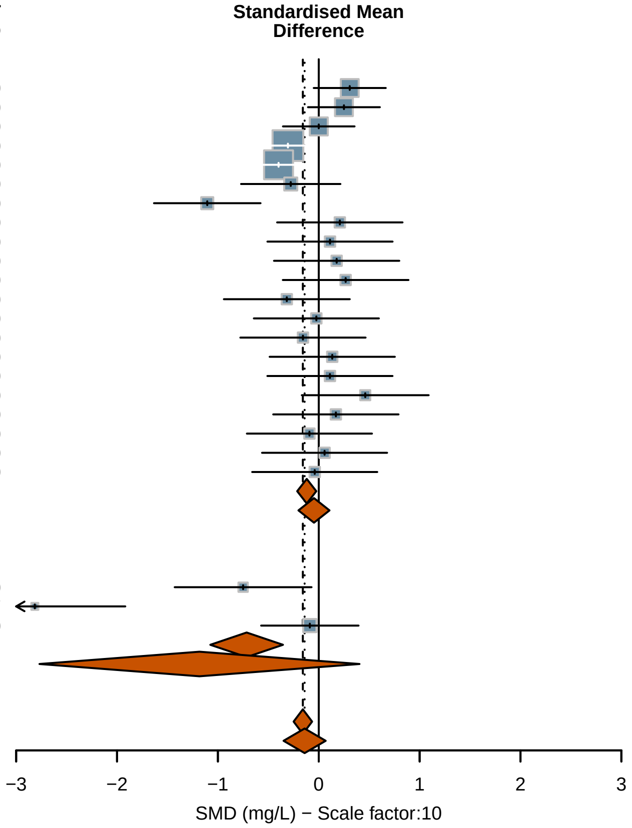

Heterogeneity: $I^2 = 71\%$, $\tau^2 = 0.1929$, $p < 0.01$
Test for subgroup differences (common effect): $\chi^2_1 = 9.83$, df = 1 ($p < 0.01$)
Test for subgroup differences (random effects): $\chi^2_1 = 1.95$, df = 1 ($p = 0.16$)

SMD: −0.16; 95%C.I.[−0.25; −0.07] P value for common effect= 7e−04

SMD: −0.14; 95%C.I.[−0.35; 0.07] P value for random effect= 0.1884

Cytokine: CRP − Treatment: Intensive

Study	After TRT Total	After TRT Mean	After TRT SD	Before TRT Total	Before TRT Mean	Before TRT SD	SMD	95%−CI	Weight (common)	Weight (random)
**Diabetes_binary:Sample with diabetic**										
Shih−Jung Lin 2011	14	0.00	0.0002	14	0.00	0.0001	0.00	[−0.74; 0.74]	2.0%	6.3%
Shih−Jung Lin 2011	14	0.00	0.0002	14	0.00	0.0001	0.34	[−0.41; 1.08]	2.0%	6.2%
Caula AL 2014	32	8.70	2.3000	32	9.60	3.9000	−0.28	[−0.77; 0.21]	4.5%	7.3%
Caula AL 2014	32	5.40	3.6000	32	9.60	3.9000	−1.11	[−1.63; −0.58]	3.9%	7.2%
Filippo Graziani 2022	20	36.70	57.3000	20	33.20	61.8000	0.06	[−0.56; 0.68]	2.8%	6.8%
Filippo Graziani 2022	20	39.80	61.6000	20	42.00	44.8000	−0.04	[−0.66; 0.58]	2.8%	6.8%
**Common effect model**	**132**			**132**			**−0.27**	**[−0.52; −0.02]**	**18.0%**	**.**
**Random effects model**							**−0.21**	**[−0.63; 0.21]**	**.**	**40.6%**
Heterogeneity: $I^2 = 65\%$, $\tau^2 = 0.1716$, $p = 0.01$										
**Diabetes_binary:Sample without diabetic**										
Tonetti 2018	61	31.00	5.0000	61	25.00	27.0000	0.31	[−0.05; 0.66]	8.6%	7.8%
Tonetti 2018	61	30.00	8.0000	61	25.00	27.0000	0.25	[−0.11; 0.61]	8.6%	7.8%
Tonetti 2018	61	25.00	7.0000	61	25.00	27.0000	0.00	[−0.35; 0.35]	8.7%	7.8%
Kamil W 2011	18	180.00	60.0000	18	230.00	70.0000	−0.75	[−1.43; −0.07]	2.4%	6.5%
Bokhari SAH 2013	180	34.10	27.6000	180	43.50	33.5000	−0.31	[−0.51; −0.10]	25.3%	8.2%
Bokhari SAH 2013	161	30.70	30.5000	161	43.50	33.5000	−0.40	[−0.62; −0.18]	22.4%	8.2%
Veena A Patil 2013	20	0.01	0.0035	20	0.02	0.0047	−2.81	[−3.71; −1.92]	1.4%	5.6%
Lobao 2018	33	2.40	3.4000	33	2.70	3.3000	−0.09	[−0.57; 0.39]	4.7%	7.4%
**Common effect model**	**595**			**595**			**−0.22**	**[−0.33; −0.10]**	**82.0%**	**.**
**Random effects model**							**−0.42**	**[−1.03; 0.20]**	**.**	**59.4%**
Heterogeneity: $I^2 = 87\%$, $\tau^2 = 0.7260$, $p < 0.01$										
**Common effect model**	**727**			**727**			**−0.23**	**[−0.33; −0.12]**	**100.0%**	**.**
**Random effects model**							**−0.30**	**[−0.67; 0.06]**	**.**	**100.0%**

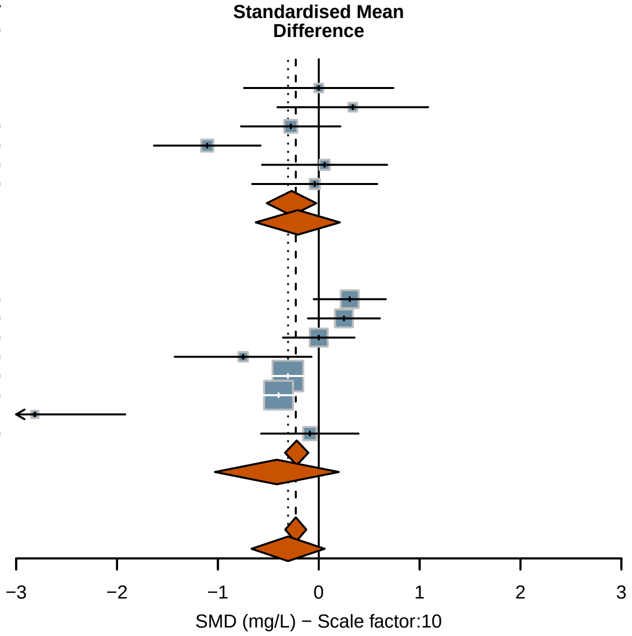

Heterogeneity: $I^2 = 81\%$, $\tau^2 = 0.4035$, $p < 0.01$
Test for subgroup differences (common effect): $\chi^2_1 = 0.14$, df = 1 ($p = 0.71$)
Test for subgroup differences (random effects): $\chi^2_1 = 0.30$, df = 1 ($p = 0.58$)

SMD: −0.23; 95%C.I.[−0.33; −0.12] P value for common effect= 0

SMD: −0.3; 95%C.I.[−0.67; 0.06] P value for random effect= 0.0997

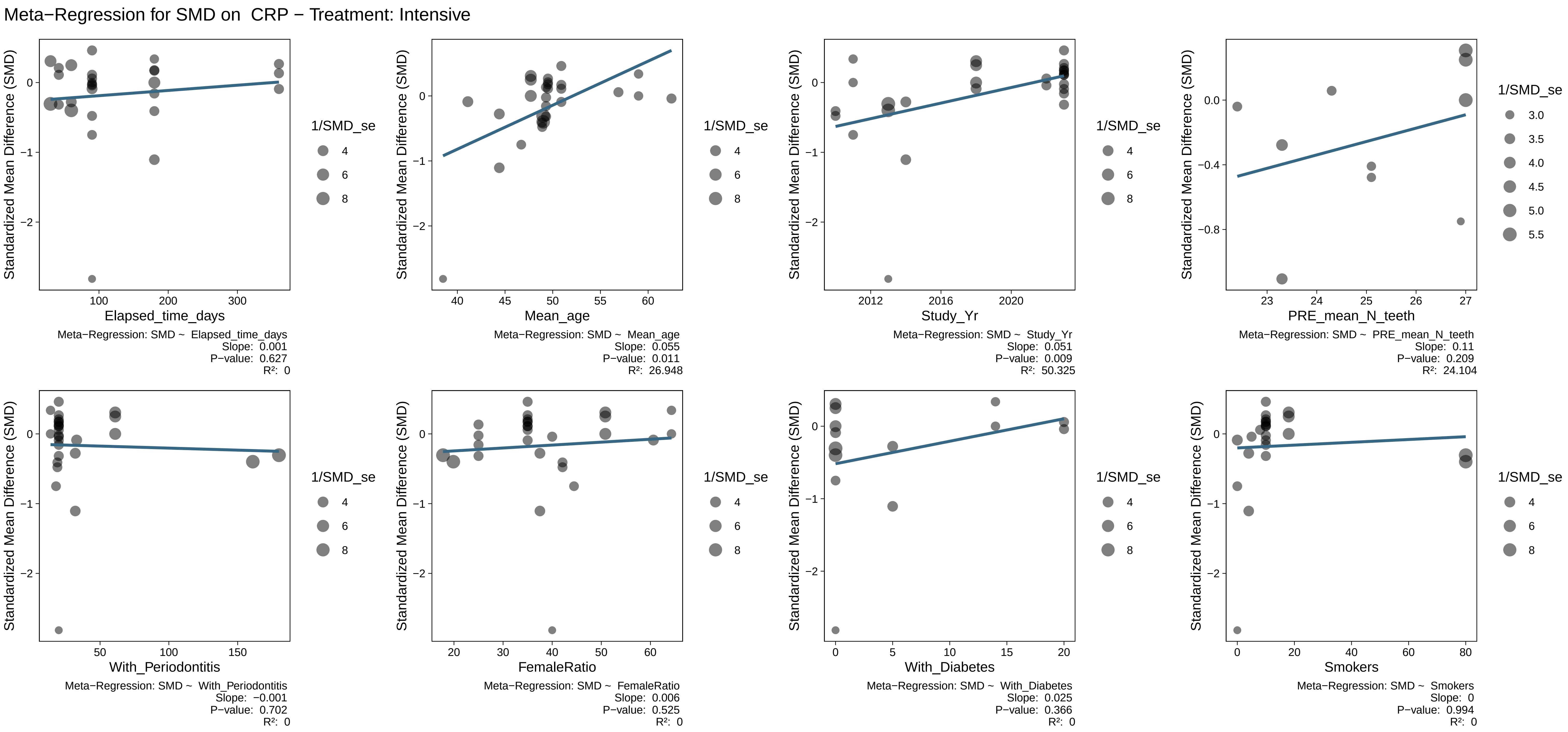
Meta−Regression for SMD on CRP − Treatment: Intensive
Standardized Mean Difference (SMD)
1/SMD_se
Elapsed_time_days
Meta−Regression: SMD ~ Elapsed_time_days
Slope: 0.001
P−value: 0.627
R²: 0
Mean_age
Meta−Regression: SMD ~ Mean_age
Slope: 0.055
P−value: 0.011
R²: 26.948
Study_Yr
Meta−Regression: SMD ~ Study_Yr
Slope: 0.051
P−value: 0.009
R²: 50.325
PRE_mean_N_teeth
Meta−Regression: SMD ~ PRE_mean_N_teeth
Slope: 0.11
P−value: 0.209
R²: 24.104
With_Periodontitis
Meta−Regression: SMD ~ With_Periodontitis
Slope: −0.001
P−value: 0.702
R²: 0
FemaleRatio
Meta−Regression: SMD ~ FemaleRatio
Slope: 0.006
P−value: 0.525
R²: 0
With_Diabetes
Meta−Regression: SMD ~ With_Diabetes
Slope: 0.025
P−value: 0.366
R²: 0
Smokers
Meta−Regression: SMD ~ Smokers
Slope: 0
P−value: 0.994
R²: 0
