# Supplementary material for: Temporal dynamics of early inflammatory markers after professional dental cleaning: a meta-analysis and spline-based meta-regression of TNF-α, IL-1β, IL-6, and (hs)CRP
Source: Front Immunol. 2025 Aug 28;16:1634622. doi: 10.3389/fimmu.2025.1634622 (PMC12423065; doi:10.3389/fimmu.2025.1634622)

Cytokine: CRP − Treatment: Standard

Study	After TRT Total	After TRT Mean	After TRT SD	Before TRT Total	Before TRT Mean	Before TRT SD	SMD	95%−CI	Weight (common)	Weight (random)
**Days after treatment:30**										
Tonetti 2018	59	22.00	3.0000	59	38.00	53.0000	−0.42	[−0.79; −0.06]	8.1%	6.8%
Ushida 2008	12	8.26	6.1480	12	4.17	2.5320	0.84	[−0.00; 1.68]	1.5%	3.1%
Ushida 2008	12	2.73	1.6650	12	3.51	2.3400	−0.37	[−1.18; 0.43]	1.6%	3.2%
Levent Kardesxler 2010	13	0.27	0.1440	13	0.26	0.1570	0.04	[−0.72; 0.81]	1.8%	3.4%
Levent Kardesxler 2010	12	0.35	0.3130	12	0.40	0.2530	−0.14	[−0.94; 0.66]	1.7%	3.3%
Levent Kardesxler 2010	15	0.22	0.2770	15	0.23	0.2000	−0.04	[−0.76; 0.68]	2.1%	3.8%
Yasuko Shimada 2010	33	4.91	4.7900	33	8.02	10.6500	−0.37	[−0.86; 0.11]	4.5%	5.6%
**Common effect model**	**156**			**156**			**−0.22**	**[−0.44; 0.01]**	**21.4%**	**.**
**Random effects model**							**−0.17**	**[−0.45; 0.11]**	**.**	**29.1%**
Heterogeneity: $I^2 = 30\%$, $\tau^2 = 0.0359$, $p = 0.20$										
**Days after treatment:42**										
Ide M 2003	24	18.00	8.0000	24	41.00	14.0000	−1.98	[−2.68; −1.28]	2.2%	3.9%
**Days after treatment:56**										
Mohammad S. Al−Zahrani 2012	40	5.50	4.1000	40	7.80	5.1000	−0.49	[−0.94; −0.05]	5.4%	6.0%
**Days after treatment:60**										
F. D'Aiuto 2005	21	29.00	23.0000	21	29.00	22.0000	0.00	[−0.60; 0.60]	2.9%	4.5%
Tonetti 2018	59	32.00	4.0000	59	38.00	53.0000	−0.16	[−0.52; 0.20]	8.2%	6.8%
**Common effect model**	**80**			**80**			**−0.12**	**[−0.43; 0.19]**	**11.2%**	**.**
**Random effects model**							**−0.12**	**[−0.43; 0.19]**	**.**	**11.4%**
Heterogeneity: $I^2 = 0\%$, $\tau^2 = 0$, $p = 0.66$										
**Days after treatment:90**										
Vidal 2009	11	90.00	80.0000	11	140.00	100.0000	−0.53	[−1.38; 0.32]	1.5%	3.0%
Levent Kardesxler 2010	13	0.21	0.1590	13	0.26	0.1570	−0.30	[−1.07; 0.47]	1.8%	3.4%
Levent Kardesxler 2010	12	0.37	0.2700	12	0.40	0.2530	−0.09	[−0.89; 0.71]	1.7%	3.3%
Levent Kardesxler 2010	15	0.18	0.1160	15	0.23	0.2000	−0.32	[−1.04; 0.40]	2.1%	3.7%
Nestor J. Lopez, 2012	81	43.90	3.3000	81	43.90	31.7000	0.00	[−0.31; 0.31]	11.3%	7.4%
**Common effect model**	**132**			**132**			**−0.12**	**[−0.36; 0.13]**	**18.4%**	**.**
**Random effects model**							**−0.12**	**[−0.36; 0.13]**	**.**	**20.8%**
Heterogeneity: $I^2 = 0\%$, $\tau^2 = 0$, $p = 0.74$										
**Days after treatment:180**										
Tonetti 2018	59	25.00	3.0000	59	38.00	53.0000	−0.34	[−0.71; 0.02]	8.1%	6.8%
Nestor J. Lopez, 2012	81	35.70	3.4000	81	43.90	31.7000	−0.36	[−0.67; −0.05]	11.2%	7.4%
**Common effect model**	**140**			**140**			**−0.35**	**[−0.59; −0.12]**	**19.3%**	**.**
**Random effects model**							**−0.35**	**[−0.59; −0.12]**	**.**	**14.2%**
Heterogeneity: $I^2 = 0\%$, $\tau^2 = 0$, $p = 0.94$										
**Days after treatment:270**										
Nestor J. Lopez, 2012	81	43.20	6.1000	81	43.90	31.7000	−0.03	[−0.34; 0.28]	11.3%	7.4%
**Days after treatment:365**										
Nestor J. Lopez, 2012	81	29.20	5.4000	81	43.90	31.7000	−0.64	[−0.96; −0.33]	10.8%	7.3%
**Common effect model**	**734**			**734**			**−0.29**	**[−0.40; −0.19]**	**100.0%**	**.**
**Random effects model**							**−0.30**	**[−0.48; −0.12]**	**.**	**100.0%**

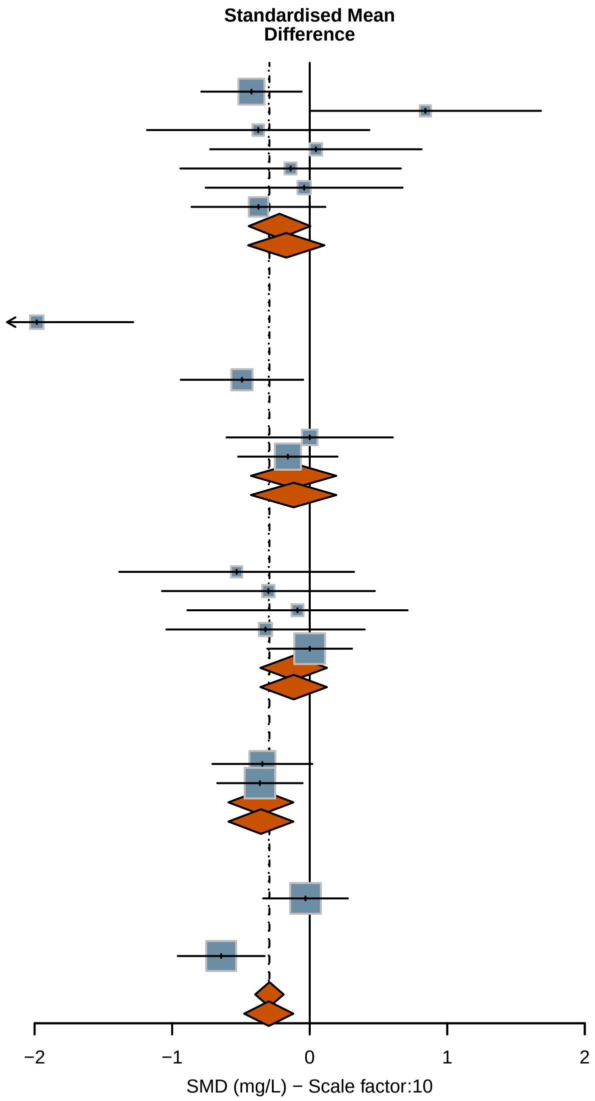

Heterogeneity: $I^2 = 58\%$, $\tau^2 = 0.0879$, $p < 0.01$
Test for subgroup differences (common effect): $\chi^2_7 = 34.62$, df = 7 ($p < 0.01$)
Test for subgroup differences (random effects): $\chi^2_7 = 34.95$, df = 7 ($p < 0.01$)

SMD: −0.29; 95%C.I.[−0.4; −0.19] P value for common effect= 0

SMD: −0.3; 95%C.I.[−0.48; −0.12] P value for random effect= 0.0011

Cytokine: CRP − Treatment: Standard

Study	After TRT Total	After TRT Mean	After TRT SD	Before TRT Total	Before TRT Mean	Before TRT SD	SMD	95%−CI	Weight (common)	Weight (random)
**MonthsAfterTRT:4−6Wks**										
Ide M 2003	24	18.00	8.0000	24	41.00	14.0000	−1.98	[−2.68; −1.28]	2.2%	3.9%
Tonetti 2018	59	22.00	3.0000	59	38.00	53.0000	−0.42	[−0.79; −0.06]	8.1%	6.8%
Ushida 2008	12	8.26	6.1480	12	4.17	2.5320	0.84	[−0.00; 1.68]	1.5%	3.1%
Ushida 2008	12	2.73	1.6650	12	3.51	2.3400	−0.37	[−1.18; 0.43]	1.6%	3.2%
Levent Kardesxler 2010	13	0.27	0.1440	13	0.26	0.1570	0.04	[−0.72; 0.81]	1.8%	3.4%
Levent Kardesxler 2010	12	0.35	0.3130	12	0.40	0.2530	−0.14	[−0.94; 0.66]	1.7%	3.3%
Levent Kardesxler 2010	15	0.22	0.2770	15	0.23	0.2000	−0.04	[−0.76; 0.68]	2.1%	3.8%
Yasuko Shimada 2010	33	4.91	4.7900	33	8.02	10.6500	−0.37	[−0.86; 0.11]	4.5%	5.6%
**Common effect model**	**180**			**180**			**−0.38**	**[−0.60; −0.17]**	**23.6%**	**.**
**Random effects model**							**−0.33**	**[−0.86; 0.21]**	**.**	**33.0%**
Heterogeneity: $I^2 = 77\%$, $\tau^2 = 0.4609$, $p < 0.01$										
**MonthsAfterTRT:Within 3 Mo.**										
F. D'Aiuto 2005	21	29.00	23.0000	21	29.00	22.0000	0.00	[−0.60; 0.60]	2.9%	4.5%
Tonetti 2018	59	32.00	4.0000	59	38.00	53.0000	−0.16	[−0.52; 0.20]	8.2%	6.8%
Vidal 2009	11	90.00	80.0000	11	140.00	100.0000	−0.53	[−1.38; 0.32]	1.5%	3.0%
Levent Kardesxler 2010	13	0.21	0.1590	13	0.26	0.1570	−0.30	[−1.07; 0.47]	1.8%	3.4%
Levent Kardesxler 2010	12	0.37	0.2700	12	0.40	0.2530	−0.09	[−0.89; 0.71]	1.7%	3.3%
Levent Kardesxler 2010	15	0.18	0.1160	15	0.23	0.2000	−0.32	[−1.04; 0.40]	2.1%	3.7%
Nestor J. Lopez, 2012	81	43.90	3.3000	81	43.90	31.7000	0.00	[−0.31; 0.31]	11.3%	7.4%
Mohammad S. Al−Zahrani 2012	40	5.50	4.1000	40	7.80	5.1000	−0.49	[−0.94; −0.05]	5.4%	6.0%
**Common effect model**	**252**			**252**			**−0.17**	**[−0.35; 0.00]**	**35.0%**	**.**
**Random effects model**							**−0.17**	**[−0.35; 0.00]**	**.**	**38.2%**
Heterogeneity: $I^2 = 0\%$, $\tau^2 = 0$, $p = 0.72$										
**MonthsAfterTRT:Within 6 Mo.**										
Tonetti 2018	59	25.00	3.0000	59	38.00	53.0000	−0.34	[−0.71; 0.02]	8.1%	6.8%
Nestor J. Lopez, 2012	81	35.70	3.4000	81	43.90	31.7000	−0.36	[−0.67; −0.05]	11.2%	7.4%
**Common effect model**	**140**			**140**			**−0.35**	**[−0.59; −0.12]**	**19.3%**	**.**
**Random effects model**							**−0.35**	**[−0.59; −0.12]**	**.**	**14.2%**
Heterogeneity: $I^2 = 0\%$, $\tau^2 = 0$, $p = 0.94$										
**MonthsAfterTRT:6Mo. and more**										
Nestor J. Lopez, 2012	81	43.20	6.1000	81	43.90	31.7000	−0.03	[−0.34; 0.28]	11.3%	7.4%
Nestor J. Lopez, 2012	81	29.20	5.4000	81	43.90	31.7000	−0.64	[−0.96; −0.33]	10.8%	7.3%
**Common effect model**	**162**			**162**			**−0.33**	**[−0.55; −0.11]**	**22.1%**	**.**
**Random effects model**							**−0.34**	**[−0.94; 0.26]**	**.**	**14.7%**
Heterogeneity: $I^2 = 87\%$, $\tau^2 = 0.1625$, $p < 0.01$										
**Common effect model**	**734**			**734**			**−0.29**	**[−0.40; −0.19]**	**100.0%**	**.**
**Random effects model**							**−0.30**	**[−0.48; −0.12]**	**.**	**100.0%**

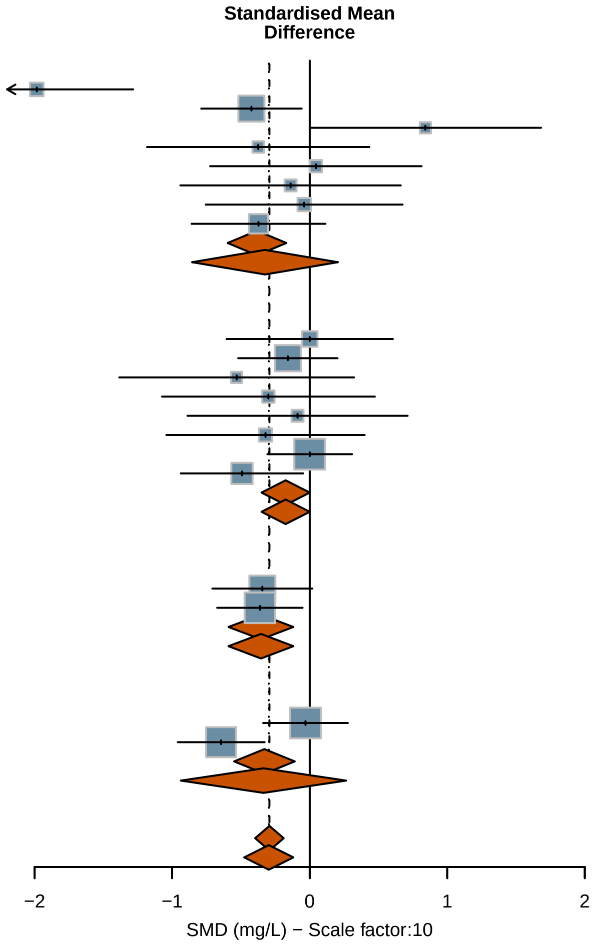

Heterogeneity: $I^2 = 58\%$, $\tau^2 = 0.0879$, $p < 0.01$
Test for subgroup differences (common effect): $\chi^2_3 = 2.79$, df = 3 ($p = 0.43$)
Test for subgroup differences (random effects): $\chi^2_3 = 1.61$, df = 3 ($p = 0.66$)

SMD: −0.29; 95%C.I.[−0.4; −0.19] P value for common effect= 0

SMD: −0.3; 95%C.I.[−0.48; −0.12] P value for random effect= 0.0011

Cytokine: CRP − Treatment: Standard

Study	After TRT Total	After TRT Mean	After TRT SD	Before TRT Total	Before TRT Mean	Before TRT SD	SMD	95%−CI	Weight (common)	Weight (random)
**Assay type:ELISA**										
Vidal 2009	11	90.00	80.0000	11	140.00	100.0000	−0.53	[−1.38; 0.32]	1.5%	3.0%
Levent Kardesxler 2010	13	0.27	0.1440	13	0.26	0.1570	0.04	[−0.72; 0.81]	1.8%	3.4%
Levent Kardesxler 2010	13	0.21	0.1590	13	0.26	0.1570	−0.30	[−1.07; 0.47]	1.8%	3.4%
Levent Kardesxler 2010	12	0.35	0.3130	12	0.40	0.2530	−0.14	[−0.94; 0.66]	1.7%	3.3%
Levent Kardesxler 2010	12	0.37	0.2700	12	0.40	0.2530	−0.09	[−0.89; 0.71]	1.7%	3.3%
Levent Kardesxler 2010	15	0.22	0.2770	15	0.23	0.2000	−0.04	[−0.76; 0.68]	2.1%	3.8%
Levent Kardesxler 2010	15	0.18	0.1160	15	0.23	0.2000	−0.32	[−1.04; 0.40]	2.1%	3.7%
Yasuko Shimada 2010	33	4.91	4.7900	33	8.02	10.6500	−0.37	[−0.86; 0.11]	4.5%	5.6%
Mohammad S. Al−Zahrani 2012	40	5.50	4.1000	40	7.80	5.1000	−0.49	[−0.94; −0.05]	5.4%	6.0%
**Common effect model**	**164**			**164**			**−0.30**	**[−0.52; −0.08]**	**22.6%**	**.**
**Random effects model**							**−0.30**	**[−0.52; −0.08]**	**.**	**35.4%**
Heterogeneity: $I^2 = 0\%$, $\tau^2 = 0$, $p = 0.95$										
**Assay type:immunoturbidimetic assay**										
F. D'Aiuto 2005	21	29.00	23.0000	21	29.00	22.0000	0.00	[−0.60; 0.60]	2.9%	4.5%
Tonetti 2018	59	22.00	3.0000	59	38.00	53.0000	−0.42	[−0.79; −0.06]	8.1%	6.8%
Tonetti 2018	59	32.00	4.0000	59	38.00	53.0000	−0.16	[−0.52; 0.20]	8.2%	6.8%
Tonetti 2018	59	25.00	3.0000	59	38.00	53.0000	−0.34	[−0.71; 0.02]	8.1%	6.8%
Nestor J. Lopez, 2012	81	43.90	3.3000	81	43.90	31.7000	0.00	[−0.31; 0.31]	11.3%	7.4%
Nestor J. Lopez, 2012	81	35.70	3.4000	81	43.90	31.7000	−0.36	[−0.67; −0.05]	11.2%	7.4%
Nestor J. Lopez, 2012	81	43.20	6.1000	81	43.90	31.7000	−0.03	[−0.34; 0.28]	11.3%	7.4%
Nestor J. Lopez, 2012	81	29.20	5.4000	81	43.90	31.7000	−0.64	[−0.96; −0.33]	10.8%	7.3%
**Common effect model**	**522**			**522**			**−0.26**	**[−0.38; −0.14]**	**72.0%**	**.**
**Random effects model**							**−0.26**	**[−0.43; −0.09]**	**.**	**54.4%**
Heterogeneity: $I^2 = 46\%$, $\tau^2 = 0.0275$, $p = 0.07$										
**Assay type:latex−enhanced nephelometry**										
Ushida 2008	12	8.26	6.1480	12	4.17	2.5320	0.84	[−0.00; 1.68]	1.5%	3.1%
Ushida 2008	12	2.73	1.6650	12	3.51	2.3400	−0.37	[−1.18; 0.43]	1.6%	3.2%
**Common effect model**	**24**			**24**			**0.21**	**[−0.37; 0.79]**	**3.2%**	**.**
**Random effects model**							**0.23**	**[−0.96; 1.42]**	**.**	**6.3%**
Heterogeneity: $I^2 = 76\%$, $\tau^2 = 0.5611$, $p = 0.04$										
**Assay type:Monoclonal Antibodies**										
Ide M 2003	24	18.00	8.0000	24	41.00	14.0000	−1.98	[−2.68; −1.28]	2.2%	3.9%
**Common effect model**	**734**			**734**			**−0.29**	**[−0.40; −0.19]**	**100.0%**	**.**
**Random effects model**							**−0.30**	**[−0.48; −0.12]**	**.**	**100.0%**

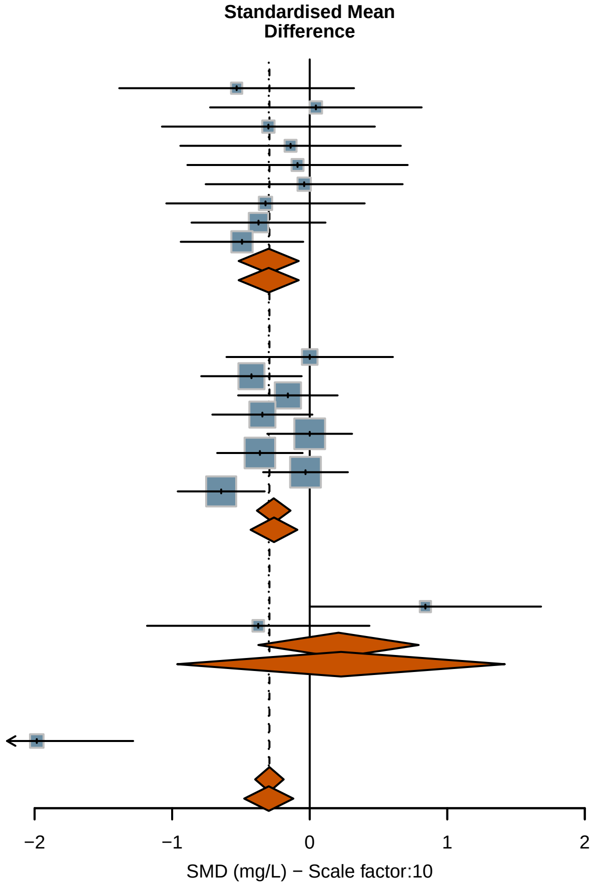

Heterogeneity: $I^2 = 58\%$, $\tau^2 = 0.0879$, $p < 0.01$
Test for subgroup differences (common effect): $\chi^2_3 = 25.48$, df = 3 ($p < 0.01$)
Test for subgroup differences (random effects): $\chi^2_3 = 22.99$, df = 3 ($p < 0.01$)

SMD: −0.29; 95%C.I.[−0.4; −0.19] P value for common effect= 0

SMD: −0.3; 95%C.I.[−0.48; −0.12] P value for random effect= 0.0011

Cytokine: CRP − Treatment: Standard

Study	After TRT Total	After TRT Mean	After TRT SD	Before TRT Total	Before TRT Mean	Before TRT SD	SMD	95%−CI	Weight (common)	Weight (random)
**Bias judgment:High risk**										
Ushida 2008	12	8.26	6.1480	12	4.17	2.5320	0.84	[−0.00; 1.68]	1.5%	3.1%
Ushida 2008	12	2.73	1.6650	12	3.51	2.3400	−0.37	[−1.18; 0.43]	1.6%	3.2%
Vidal 2009	11	90.00	80.0000	11	140.00	100.0000	−0.53	[−1.38; 0.32]	1.5%	3.0%
Yasuko Shimada 2010	33	4.91	4.7900	33	8.02	10.6500	−0.37	[−0.86; 0.11]	4.5%	5.6%
Mohammad S. Al−Zahrani 2012	40	5.50	4.1000	40	7.80	5.1000	−0.49	[−0.94; −0.05]	5.4%	6.0%
**Common effect model**	**108**			**108**			**−0.31**	**[−0.58; −0.04]**	**14.6%**	**.**
**Random effects model**							**−0.24**	**[−0.66; 0.17]**	**.**	**20.8%**
Heterogeneity: $I^2 = 51\%$, $\tau^2 = 0.1130$, $p = 0.09$										
**Bias judgment:Low risk**										
Ide M 2003	24	18.00	8.0000	24	41.00	14.0000	−1.98	[−2.68; −1.28]	2.2%	3.9%
Tonetti 2018	59	22.00	3.0000	59	38.00	53.0000	−0.42	[−0.79; −0.06]	8.1%	6.8%
Tonetti 2018	59	32.00	4.0000	59	38.00	53.0000	−0.16	[−0.52; 0.20]	8.2%	6.8%
Tonetti 2018	59	25.00	3.0000	59	38.00	53.0000	−0.34	[−0.71; 0.02]	8.1%	6.8%
Levent Kardesxler 2010	13	0.27	0.1440	13	0.26	0.1570	0.04	[−0.72; 0.81]	1.8%	3.4%
Levent Kardesxler 2010	13	0.21	0.1590	13	0.26	0.1570	−0.30	[−1.07; 0.47]	1.8%	3.4%
Levent Kardesxler 2010	12	0.35	0.3130	12	0.40	0.2530	−0.14	[−0.94; 0.66]	1.7%	3.3%
Levent Kardesxler 2010	12	0.37	0.2700	12	0.40	0.2530	−0.09	[−0.89; 0.71]	1.7%	3.3%
Levent Kardesxler 2010	15	0.22	0.2770	15	0.23	0.2000	−0.04	[−0.76; 0.68]	2.1%	3.8%
Levent Kardesxler 2010	15	0.18	0.1160	15	0.23	0.2000	−0.32	[−1.04; 0.40]	2.1%	3.7%
Nestor J. Lopez, 2012	81	43.90	3.3000	81	43.90	31.7000	0.00	[−0.31; 0.31]	11.3%	7.4%
Nestor J. Lopez, 2012	81	35.70	3.4000	81	43.90	31.7000	−0.36	[−0.67; −0.05]	11.2%	7.4%
Nestor J. Lopez, 2012	81	43.20	6.1000	81	43.90	31.7000	−0.03	[−0.34; 0.28]	11.3%	7.4%
Nestor J. Lopez, 2012	81	29.20	5.4000	81	43.90	31.7000	−0.64	[−0.96; −0.33]	10.8%	7.3%
**Common effect model**	**605**			**605**			**−0.30**	**[−0.42; −0.19]**	**82.4%**	**.**
**Random effects model**							**−0.33**	**[−0.55; −0.11]**	**.**	**74.6%**
Heterogeneity: $I^2 = 64\%$, $\tau^2 = 0.1091$, $p < 0.01$										
**Bias judgment:Some concerns**										
F. D'Aiuto 2005	21	29.00	23.0000	21	29.00	22.0000	0.00	[−0.60; 0.60]	2.9%	4.5%
**Common effect model**	**734**			**734**			**−0.29**	**[−0.40; −0.19]**	**100.0%**	**.**
**Random effects model**							**−0.30**	**[−0.48; −0.12]**	**.**	**100.0%**

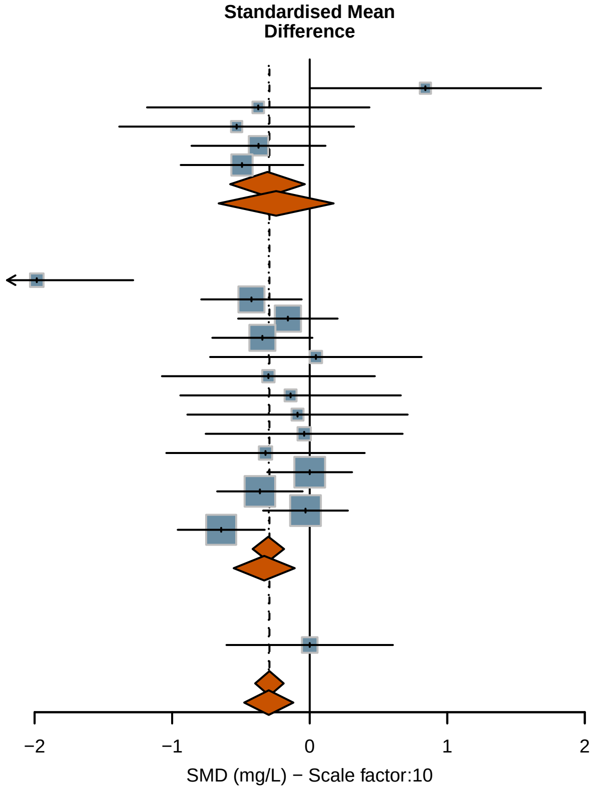

Heterogeneity: $I^2 = 58\%$, $\tau^2 = 0.0879$, $p < 0.01$
Test for subgroup differences (common effect): $\chi^2_2 = 0.93$, df = 2 ($p = 0.63$)
Test for subgroup differences (random effects): $\chi^2_2 = 1.05$, df = 2 ($p = 0.59$)

SMD: −0.29; 95%C.I.[−0.4; −0.19] P value for common effect= 0

SMD: −0.3; 95%C.I.[−0.48; −0.12] P value for random effect= 0.0011

Cytokine: CRP − Treatment: Standard

Study	After TRT Total	After TRT Mean	After TRT SD	Before TRT Total	Before TRT Mean	Before TRT SD	SMD	95%−CI	Weight (common)	Weight (random)
**Study design:Comparative study**										
Levent Kardesxler 2010	13	0.27	0.1440	13	0.26	0.1570	0.04	[−0.72; 0.81]	1.8%	3.4%
Levent Kardesxler 2010	13	0.21	0.1590	13	0.26	0.1570	−0.30	[−1.07; 0.47]	1.8%	3.4%
Levent Kardesxler 2010	12	0.35	0.3130	12	0.40	0.2530	−0.14	[−0.94; 0.66]	1.7%	3.3%
Levent Kardesxler 2010	12	0.37	0.2700	12	0.40	0.2530	−0.09	[−0.89; 0.71]	1.7%	3.3%
Levent Kardesxler 2010	15	0.22	0.2770	15	0.23	0.2000	−0.04	[−0.76; 0.68]	2.1%	3.8%
Levent Kardesxler 2010	15	0.18	0.1160	15	0.23	0.2000	−0.32	[−1.04; 0.40]	2.1%	3.7%
Yasuko Shimada 2010	33	4.91	4.7900	33	8.02	10.6500	−0.37	[−0.86; 0.11]	4.5%	5.6%
**Common effect model**	**113**			**113**			**−0.21**	**[−0.47; 0.05]**	**15.7%**	**.**
**Random effects model**							**−0.21**	**[−0.47; 0.05]**	**.**	**26.4%**
Heterogeneity: $I^2 = 0\%$, $\tau^2 = 0$, $p = 0.97$										
**Study design:Controlled clinical trial**										
Nestor J. Lopez, 2012	81	43.90	3.3000	81	43.90	31.7000	0.00	[−0.31; 0.31]	11.3%	7.4%
Nestor J. Lopez, 2012	81	35.70	3.4000	81	43.90	31.7000	−0.36	[−0.67; −0.05]	11.2%	7.4%
Nestor J. Lopez, 2012	81	43.20	6.1000	81	43.90	31.7000	−0.03	[−0.34; 0.28]	11.3%	7.4%
Nestor J. Lopez, 2012	81	29.20	5.4000	81	43.90	31.7000	−0.64	[−0.96; −0.33]	10.8%	7.3%
Mohammad S. Al−Zahrani 2012	40	5.50	4.1000	40	7.80	5.1000	−0.49	[−0.94; −0.05]	5.4%	6.0%
**Common effect model**	**364**			**364**			**−0.28**	**[−0.43; −0.13]**	**50.1%**	**.**
**Random effects model**							**−0.29**	**[−0.55; −0.04]**	**.**	**35.4%**
Heterogeneity: $I^2 = 66\%$, $\tau^2 = 0.0550$, $p = 0.02$										
**Study design:Prospective Interventional cohort study**										
Ide M 2003	24	18.00	8.0000	24	41.00	14.0000	−1.98	[−2.68; −1.28]	2.2%	3.9%
**Study design:RCT**										
F. D'Aiuto 2005	21	29.00	23.0000	21	29.00	22.0000	0.00	[−0.60; 0.60]	2.9%	4.5%
Tonetti 2018	59	22.00	3.0000	59	38.00	53.0000	−0.42	[−0.79; −0.06]	8.1%	6.8%
Tonetti 2018	59	32.00	4.0000	59	38.00	53.0000	−0.16	[−0.52; 0.20]	8.2%	6.8%
Tonetti 2018	59	25.00	3.0000	59	38.00	53.0000	−0.34	[−0.71; 0.02]	8.1%	6.8%
Ushida 2008	12	8.26	6.1480	12	4.17	2.5320	0.84	[−0.00; 1.68]	1.5%	3.1%
Ushida 2008	12	2.73	1.6650	12	3.51	2.3400	−0.37	[−1.18; 0.43]	1.6%	3.2%
Vidal 2009	11	90.00	80.0000	11	140.00	100.0000	−0.53	[−1.38; 0.32]	1.5%	3.0%
**Common effect model**	**233**			**233**			**−0.24**	**[−0.42; −0.06]**	**32.1%**	**.**
**Random effects model**							**−0.24**	**[−0.42; −0.06]**	**.**	**34.3%**
Heterogeneity: $I^2 = 33\%$, $\tau^2 = < 0.0001$, $p = 0.17$										
**Common effect model**	**734**			**734**			**−0.29**	**[−0.40; −0.19]**	**100.0%**	**.**
**Random effects model**							**−0.30**	**[−0.48; −0.12]**	**.**	**100.0%**

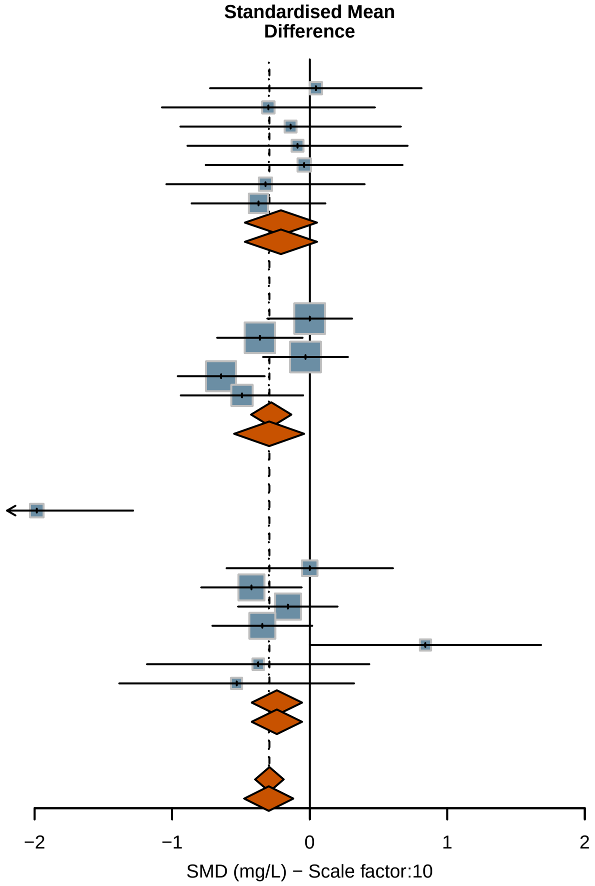

Heterogeneity: $I^2 = 58\%$, $\tau^2 = 0.0879$, $p < 0.01$
Test for subgroup differences (common effect): $\chi^2_3 = 23.14$, df = 3 ($p < 0.01$)
Test for subgroup differences (random effects): $\chi^2_3 = 23.08$, df = 3 ($p < 0.01$)

SMD: −0.29; 95%C.I.[−0.4; −0.19] P value for common effect= 0

SMD: −0.3; 95%C.I.[−0.48; −0.12] P value for random effect= 0.0011

Cytokine: CRP − Treatment: Standard

Study	After TRT Total	After TRT Mean	After TRT SD	Before TRT Total	Before TRT Mean	Before TRT SD	SMD	95%−CI	Weight (common)	Weight (random)
**Additional_devices:FMD and water**										
Ushida 2008	12	8.26	6.1480	12	4.17	2.5320	0.84	[−0.00; 1.68]	48.0%	49.5%
**Additional_devices:QMD**										
Ushida 2008	12	2.73	1.6650	12	3.51	2.3400	−0.37	[−1.18; 0.43]	52.0%	50.5%
**Common effect model**	**24**			**24**			**0.21**	**[−0.37; 0.79]**	**100.0%**	**.**
**Random effects model**							**0.23**	**[−0.96; 1.42]**	**.**	**100.0%**

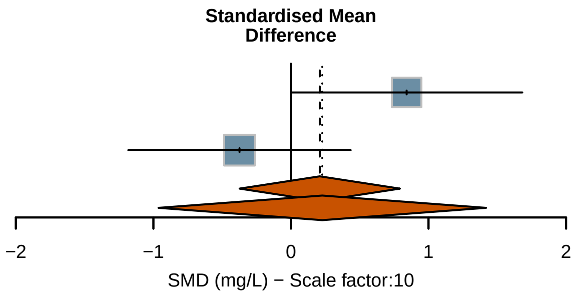

Heterogeneity: $I^2 = 76\%$, $\tau^2 = 0.5611$, $p = 0.04$
Test for subgroup differences (common effect): $\chi^2_1 = 4.17$, df = 1 ($p = 0.04$)
Test for subgroup differences (random effects): $\chi^2_1 = 4.17$, df = 1 ($p = 0.04$)

SMD: 0.21; 95%C.I.[−0.37; 0.79] P value for common effect= 0.4814

SMD: 0.23; 95%C.I.[−0.96; 1.42] P value for random effect= 0.7079

Cytokine: CRP − Treatment: Standard

Study	After TRT Total	After TRT Mean	After TRT SD	Before TRT Total	Before TRT Mean	Before TRT SD	SMD	95%−CI	Weight (common)	Weight (random)
**Female_binary:Only Female**										
Mohammad S. Al−Zahrani 2012	40	5.50	4.1000	40	7.80	5.1000	−0.49	[−0.94; −0.05]	5.4%	6.0%
**Female_binary:Sex mixed**										
Ide M 2003	24	18.00	8.0000	24	41.00	14.0000	−1.98	[−2.68; −1.28]	2.2%	3.9%
F. D'Aiuto 2005	21	29.00	23.0000	21	29.00	22.0000	0.00	[−0.60; 0.60]	2.9%	4.5%
Tonetti 2018	59	22.00	3.0000	59	38.00	53.0000	−0.42	[−0.79; −0.06]	8.1%	6.8%
Tonetti 2018	59	32.00	4.0000	59	38.00	53.0000	−0.16	[−0.52; 0.20]	8.2%	6.8%
Tonetti 2018	59	25.00	3.0000	59	38.00	53.0000	−0.34	[−0.71; 0.02]	8.1%	6.8%
Ushida 2008	12	8.26	6.1480	12	4.17	2.5320	0.84	[−0.00; 1.68]	1.5%	3.1%
Ushida 2008	12	2.73	1.6650	12	3.51	2.3400	−0.37	[−1.18; 0.43]	1.6%	3.2%
Vidal 2009	11	90.00	80.0000	11	140.00	100.0000	−0.53	[−1.38; 0.32]	1.5%	3.0%
Levent Kardesxler 2010	13	0.27	0.1440	13	0.26	0.1570	0.04	[−0.72; 0.81]	1.8%	3.4%
Levent Kardesxler 2010	13	0.21	0.1590	13	0.26	0.1570	−0.30	[−1.07; 0.47]	1.8%	3.4%
Levent Kardesxler 2010	12	0.35	0.3130	12	0.40	0.2530	−0.14	[−0.94; 0.66]	1.7%	3.3%
Levent Kardesxler 2010	12	0.37	0.2700	12	0.40	0.2530	−0.09	[−0.89; 0.71]	1.7%	3.3%
Levent Kardesxler 2010	15	0.22	0.2770	15	0.23	0.2000	−0.04	[−0.76; 0.68]	2.1%	3.8%
Levent Kardesxler 2010	15	0.18	0.1160	15	0.23	0.2000	−0.32	[−1.04; 0.40]	2.1%	3.7%
Yasuko Shimada 2010	33	4.91	4.7900	33	8.02	10.6500	−0.37	[−0.86; 0.11]	4.5%	5.6%
Nestor J. Lopez, 2012	81	43.90	3.3000	81	43.90	31.7000	0.00	[−0.31; 0.31]	11.3%	7.4%
Nestor J. Lopez, 2012	81	35.70	3.4000	81	43.90	31.7000	−0.36	[−0.67; −0.05]	11.2%	7.4%
Nestor J. Lopez, 2012	81	43.20	6.1000	81	43.90	31.7000	−0.03	[−0.34; 0.28]	11.3%	7.4%
Nestor J. Lopez, 2012	81	29.20	5.4000	81	43.90	31.7000	−0.64	[−0.96; −0.33]	10.8%	7.3%
**Common effect model**	**694**			**694**			**−0.28**	**[−0.39; −0.17]**	**94.6%**	**.**
**Random effects model**							**−0.28**	**[−0.48; −0.09]**	**.**	**94.0%**
Heterogeneity: $I^2 = 60\%$, $\tau^2 = 0.0995$, $p < 0.01$										
**Common effect model**	**734**			**734**			**−0.29**	**[−0.40; −0.19]**	**100.0%**	**.**
**Random effects model**							**−0.30**	**[−0.48; −0.12]**	**.**	**100.0%**

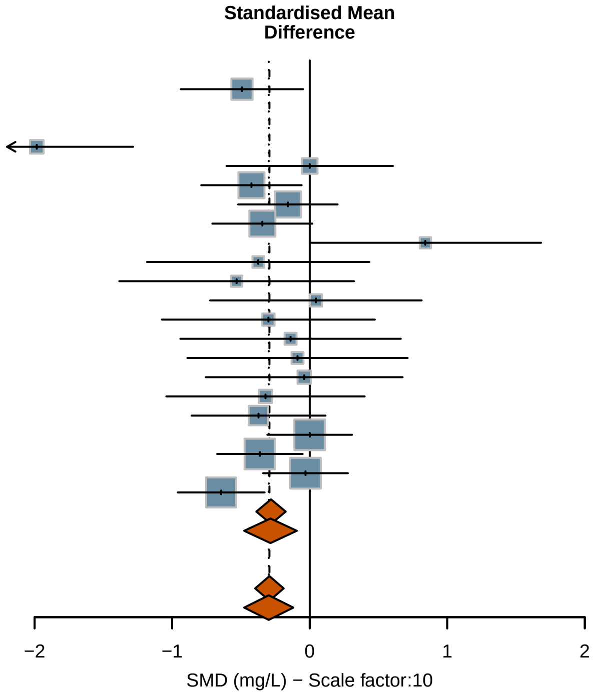

Heterogeneity: $I^2 = 58\%$, $\tau^2 = 0.0879$, $p < 0.01$
Test for subgroup differences (common effect): $\chi^2_1 = 0.82$, df = 1 ($p = 0.37$)
Test for subgroup differences (random effects): $\chi^2_1 = 0.70$, df = 1 ($p = 0.40$)

SMD: −0.29; 95%C.I.[−0.4; −0.19] P value for common effect= 0

SMD: −0.3; 95%C.I.[−0.48; −0.12] P value for random effect= 0.0011

Cytokine: CRP − Treatment: Standard

Study	After TRT Total	After TRT Mean	After TRT SD	Before TRT Total	Before TRT Mean	Before TRT SD	SMD	95%−CI	Weight (common)	Weight (random)
**Smoke_binary:Sample with smokers**										
F. D'Aiuto 2005	21	29.00	23.0000	21	29.00	22.0000	0.00	[−0.60; 0.60]	2.9%	4.5%
Tonetti 2018	59	22.00	3.0000	59	38.00	53.0000	−0.42	[−0.79; −0.06]	8.1%	6.8%
Tonetti 2018	59	32.00	4.0000	59	38.00	53.0000	−0.16	[−0.52; 0.20]	8.2%	6.8%
Tonetti 2018	59	25.00	3.0000	59	38.00	53.0000	−0.34	[−0.71; 0.02]	8.1%	6.8%
Vidal 2009	11	90.00	80.0000	11	140.00	100.0000	−0.53	[−1.38; 0.32]	1.5%	3.0%
Levent Kardesxler 2010	13	0.27	0.1440	13	0.26	0.1570	0.04	[−0.72; 0.81]	1.8%	3.4%
Levent Kardesxler 2010	13	0.21	0.1590	13	0.26	0.1570	−0.30	[−1.07; 0.47]	1.8%	3.4%
Levent Kardesxler 2010	12	0.35	0.3130	12	0.40	0.2530	−0.14	[−0.94; 0.66]	1.7%	3.3%
Levent Kardesxler 2010	12	0.37	0.2700	12	0.40	0.2530	−0.09	[−0.89; 0.71]	1.7%	3.3%
Levent Kardesxler 2010	15	0.22	0.2770	15	0.23	0.2000	−0.04	[−0.76; 0.68]	2.1%	3.8%
Levent Kardesxler 2010	15	0.18	0.1160	15	0.23	0.2000	−0.32	[−1.04; 0.40]	2.1%	3.7%
Yasuko Shimada 2010	33	4.91	4.7900	33	8.02	10.6500	−0.37	[−0.86; 0.11]	4.5%	5.6%
Nestor J. Lopez, 2012	81	43.90	3.3000	81	43.90	31.7000	0.00	[−0.31; 0.31]	11.3%	7.4%
Nestor J. Lopez, 2012	81	35.70	3.4000	81	43.90	31.7000	−0.36	[−0.67; −0.05]	11.2%	7.4%
Nestor J. Lopez, 2012	81	43.20	6.1000	81	43.90	31.7000	−0.03	[−0.34; 0.28]	11.3%	7.4%
Nestor J. Lopez, 2012	81	29.20	5.4000	81	43.90	31.7000	−0.64	[−0.96; −0.33]	10.8%	7.3%
**Common effect model**	**646**			**646**			**−0.26**	**[−0.37; −0.15]**	**89.2%**	**.**
**Random effects model**							**−0.25**	**[−0.39; −0.12]**	**.**	**83.9%**
Heterogeneity: $I^2 = 0\%$, $\tau^2 = 0.0151$, $p = 0.47$										
**Smoke_binary:Sample without smokers**										
Ide M 2003	24	18.00	8.0000	24	41.00	14.0000	−1.98	[−2.68; −1.28]	2.2%	3.9%
Ushida 2008	12	8.26	6.1480	12	4.17	2.5320	0.84	[−0.00; 1.68]	1.5%	3.1%
Ushida 2008	12	2.73	1.6650	12	3.51	2.3400	−0.37	[−1.18; 0.43]	1.6%	3.2%
Mohammad S. Al−Zahrani 2012	40	5.50	4.1000	40	7.80	5.1000	−0.49	[−0.94; −0.05]	5.4%	6.0%
**Common effect model**	**88**			**88**			**−0.59**	**[−0.91; −0.27]**	**10.8%**	**.**
**Random effects model**							**−0.52**	**[−1.63; 0.60]**	**.**	**16.1%**
Heterogeneity: $I^2 = 89\%$, $\tau^2 = 1.1649$, $p < 0.01$										
**Common effect model**	**734**			**734**			**−0.29**	**[−0.40; −0.19]**	**100.0%**	**.**
**Random effects model**							**−0.30**	**[−0.48; −0.12]**	**.**	**100.0%**

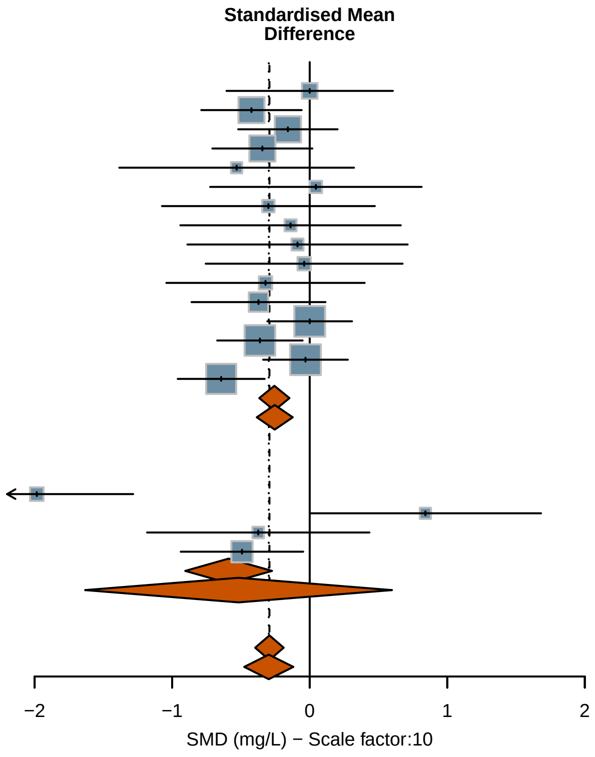

Heterogeneity: $I^2 = 58\%$, $\tau^2 = 0.0879$, $p < 0.01$
Test for subgroup differences (common effect): $\chi^2_1 = 3.80$, df = 1 ($p = 0.05$)
Test for subgroup differences (random effects): $\chi^2_1 = 0.21$, df = 1 ($p = 0.65$)

SMD: −0.29; 95%C.I.[−0.4; −0.19] P value for common effect= 0

SMD: −0.3; 95%C.I.[−0.48; −0.12] P value for random effect= 0.0011

Cytokine: CRP − Treatment: Standard

Study	After TRT Total	After TRT Mean	After TRT SD	Before TRT Total	Before TRT Mean	Before TRT SD	SMD	95%−CI	Weight (common)	Weight (random)
**Diabetes_binary:Sample with diabetic**										
Levent Kardesxler 2010	13	0.27	0.1440	13	0.26	0.1570	0.04	[−0.72; 0.81]	3.3%	5.2%
Levent Kardesxler 2010	13	0.21	0.1590	13	0.26	0.1570	−0.30	[−1.07; 0.47]	3.2%	5.1%
Levent Kardesxler 2010	12	0.35	0.3130	12	0.40	0.2530	−0.14	[−0.94; 0.66]	3.0%	4.9%
Levent Kardesxler 2010	12	0.37	0.2700	12	0.40	0.2530	−0.09	[−0.89; 0.71]	3.0%	4.9%
**Common effect model**	**50**			**50**			**−0.12**	**[−0.51; 0.27]**	**12.6%**	**.**
**Random effects model**							**−0.12**	**[−0.51; 0.27]**	**.**	**20.2%**
Heterogeneity: $I^2 = 0\%$, $\tau^2 = 0$, $p = 0.94$										
**Diabetes_binary:Sample without diabetic**										
Ide M 2003	24	18.00	8.0000	24	41.00	14.0000	−1.98	[−2.68; −1.28]	4.0%	5.7%
F. D'Aiuto 2005	21	29.00	23.0000	21	29.00	22.0000	0.00	[−0.60; 0.60]	5.3%	6.5%
Tonetti 2018	59	22.00	3.0000	59	38.00	53.0000	−0.42	[−0.79; −0.06]	14.6%	8.9%
Tonetti 2018	59	32.00	4.0000	59	38.00	53.0000	−0.16	[−0.52; 0.20]	14.9%	8.9%
Tonetti 2018	59	25.00	3.0000	59	38.00	53.0000	−0.34	[−0.71; 0.02]	14.7%	8.9%
Ushida 2008	12	8.26	6.1480	12	4.17	2.5320	0.84	[−0.00; 1.68]	2.7%	4.7%
Ushida 2008	12	2.73	1.6650	12	3.51	2.3400	−0.37	[−1.18; 0.43]	3.0%	4.9%
Vidal 2009	11	90.00	80.0000	11	140.00	100.0000	−0.53	[−1.38; 0.32]	2.7%	4.6%
Levent Kardesxler 2010	15	0.22	0.2770	15	0.23	0.2000	−0.04	[−0.76; 0.68]	3.8%	5.6%
Levent Kardesxler 2010	15	0.18	0.1160	15	0.23	0.2000	−0.32	[−1.04; 0.40]	3.7%	5.5%
Yasuko Shimada 2010	33	4.91	4.7900	33	8.02	10.6500	−0.37	[−0.86; 0.11]	8.2%	7.6%
Mohammad S. Al−Zahrani 2012	40	5.50	4.1000	40	7.80	5.1000	−0.49	[−0.94; −0.05]	9.8%	8.1%
**Common effect model**	**360**			**360**			**−0.35**	**[−0.50; −0.20]**	**87.4%**	**.**
**Random effects model**							**−0.36**	**[−0.67; −0.05]**	**.**	**79.8%**
Heterogeneity: $I^2 = 66\%$, $\tau^2 = 0.2074$, $p < 0.01$										
**Common effect model**	**410**			**410**			**−0.32**	**[−0.46; −0.18]**	**100.0%**	**.**
**Random effects model**							**−0.31**	**[−0.55; −0.07]**	**.**	**100.0%**

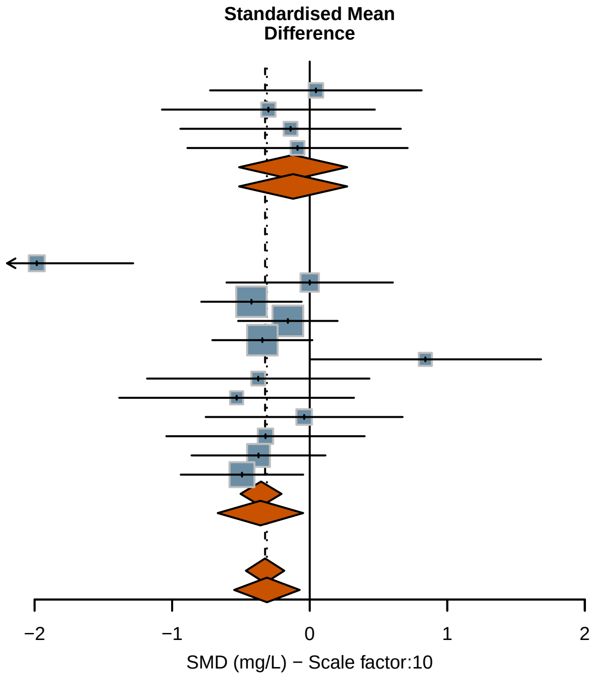

Heterogeneity: $I^2 = 56\%$, $\tau^2 = 0.1314$, $p < 0.01$
Test for subgroup differences (common effect): $\chi^2_1 = 1.18$, df = 1 ($p = 0.28$)
Test for subgroup differences (random effects): $\chi^2_1 = 0.87$, df = 1 ($p = 0.35$)

SMD: −0.32; 95%C.I.[−0.46; −0.18] P value for common effect= 0

SMD: −0.31; 95%C.I.[−0.55; −0.07] P value for random effect= 0.0104

Meta−Regression for SMD on CRP − Treatment: Standard

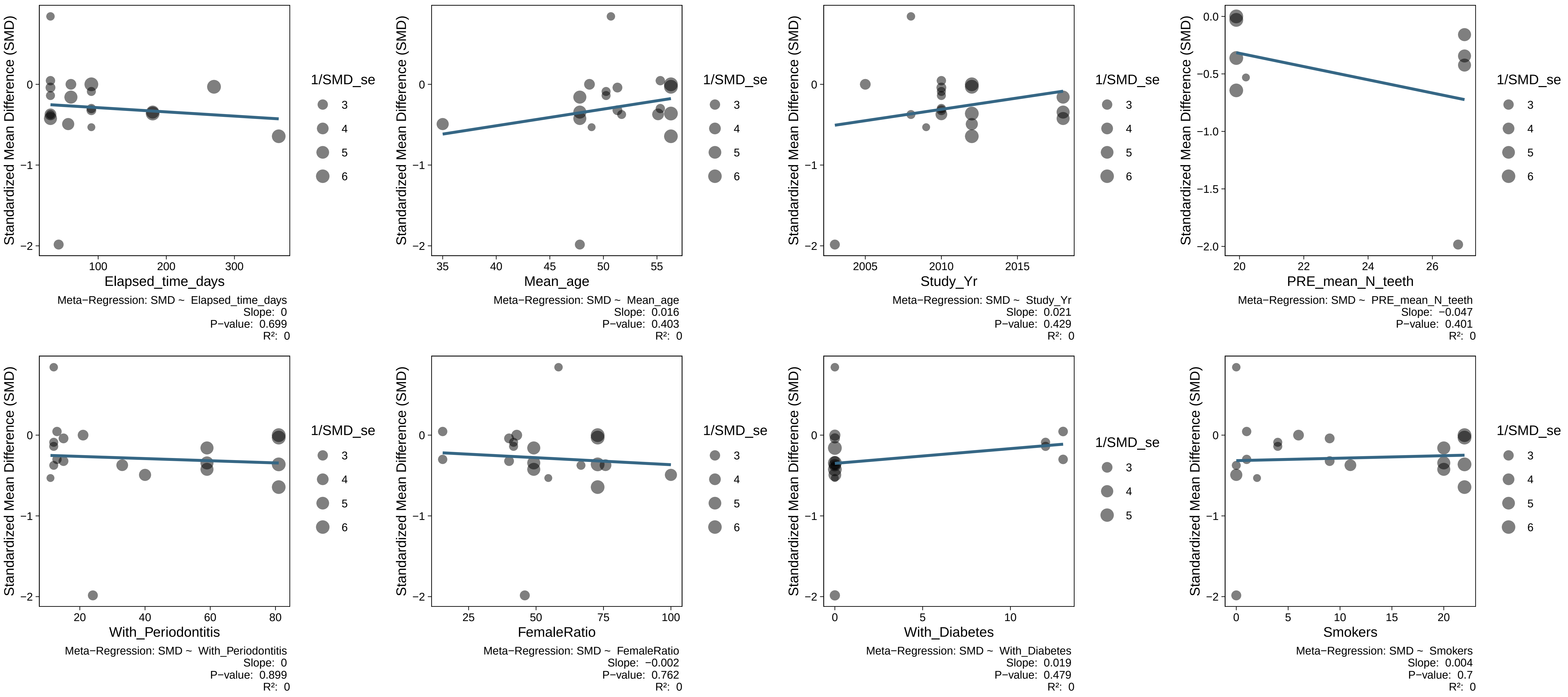

Supplement: Supplementary file 1 [file DataSheet1.zip › Supplementary materials/PDF/CRP_Standard_results.pdf]
