# Supplementary material for: Temporal dynamics of early inflammatory markers after professional dental cleaning: a meta-analysis and spline-based meta-regression of TNF-α, IL-1β, IL-6, and (hs)CRP
Source: Front Immunol. 2025 Aug 28;16:1634622. doi: 10.3389/fimmu.2025.1634622 (PMC12423065; doi:10.3389/fimmu.2025.1634622)
Supplement: Supplementary file 1 [file DataSheet1.zip › Supplementary materials/PDF/hs-CRP_Intensive_results.pdf]

Cytokine: hs−CRP − Treatment: Intensive

Study	Total (After TRT)	Mean (After TRT)	SD (After TRT)	Total (Before TRT)	Mean (Before TRT)	SD (Before TRT)	SMD	95%−CI	Weight (common)	Weight (random)
**Days after treatment:30**										
Panagiotis A. Koromantzos 2012	26	15.70	10.7000	26	15.20	9.7000	0.05	[−0.50; 0.59]	2.2%	2.7%
Yasuichi Munenaga 2013	33	9.10	8.6000	33	18.30	19.0000	−0.62	[−1.11; −0.12]	2.6%	2.8%
Yasuichi Munenaga 2013	47	3.80	3.7000	47	2.30	1.1000	0.55	[0.13; 0.96]	3.8%	2.8%
Gaetano Isola 2023	23	392.00	12.0000	23	417.00	10.0000	−2.22	[−2.97; −1.48]	1.2%	2.7%
Gaetano Isola 2023	23	355.00	19.0000	23	421.00	19.0000	−3.41	[−4.34; −2.48]	0.7%	2.7%
Bansari Shah 2022	30	10.14	0.1414	30	19.45	0.3540	−34.09	[−40.41; −27.77]	0.0%	1.3%
**Common effect model**	**182**			**182**			**−0.49**	**[−0.73; −0.24]**	**10.6%**	**.**
**Random effects model**							**−6.36**	**[−16.77; 4.05]**	**.**	**15.0%**
Heterogeneity: $I^2 = 97\%$, $\tau^2 = 167.5591$, $p < 0.01$										
**Days after treatment:42**										
Steven Offenbacher 2006	25	185.40	45.0000	25	89.00	18.3000	2.76	[1.97; 3.55]	1.0%	2.7%
Tuter G 2017	18	27.70	22.6000	18	49.60	39.1000	−0.67	[−1.34; 0.00]	1.4%	2.7%
Gulay Tuter 2010	21	6.60	1.9000	21	8.00	2.0000	−0.70	[−1.33; −0.08]	1.7%	2.7%
Fang F 2015	48	23.40	14.4000	48	30.10	17.0000	−0.42	[−0.83; −0.02]	3.9%	2.8%
**Common effect model**	**112**			**112**			**−0.11**	**[−0.40; 0.17]**	**8.1%**	**.**
**Random effects model**							**0.23**	**[−1.40; 1.85]**	**.**	**11.0%**
Heterogeneity: $I^2 = 95\%$, $\tau^2 = 2.6543$, $p < 0.01$										
**Days after treatment:45**										
Lei Chen 2012	42	23.80	28.8000	42	32.10	44.5000	−0.22	[−0.65; 0.21]	3.5%	2.8%
**Days after treatment:49**										
Stefan Renvert	28	57.40	85.3000	28	41.90	28.0000	0.24	[−0.29; 0.77]	2.3%	2.8%
**Days after treatment:56**										
Pradeep Koppolu 2013	20	2.90	1.2000	20	4.50	1.2000	−1.31	[−2.00; −0.62]	1.4%	2.7%
**Days after treatment:60**										
Gaetano Isola 2023	23	382.00	10.0000	23	417.00	10.0000	−3.44	[−4.37; −2.51]	0.7%	2.7%
Gaetano Isola 2023	23	358.00	14.0000	23	421.00	19.0000	−3.71	[−4.69; −2.73]	0.7%	2.7%
**Common effect model**	**46**			**46**			**−3.57**	**[−4.24; −2.89]**	**1.4%**	**.**
**Random effects model**							**−3.57**	**[−4.24; −2.89]**	**.**	**5.4%**
Heterogeneity: $I^2 = 0\%$, $\tau^2 = 0$, $p = 0.70$										
**Days after treatment:90**										
O fentog lu 2010	20	114.00	171.0000	20	63.00	120.0000	0.34	[−0.29; 0.96]	1.7%	2.7%
Panagiotis A. Koromantzos 2012	26	12.50	13.3000	26	15.20	9.7000	−0.23	[−0.77; 0.32]	2.2%	2.7%
Eduardo Machado Vilela 2011	36	32.80	21.0000	36	40.40	24.0000	−0.33	[−0.80; 0.13]	3.0%	2.8%
Eduardo Machado Vilela 2011	20	22.00	23.0000	20	30.40	38.0000	−0.26	[−0.88; 0.36]	1.7%	2.7%
Lei Chen 2012	42	14.80	10.0000	42	32.10	44.5000	−0.53	[−0.97; −0.10]	3.4%	2.8%
Fernando Llambes 2012	23	18.80	15.2000	23	20.00	15.2000	−0.08	[−0.66; 0.50]	1.9%	2.7%
Umut Altay 2013	22	34.00	7.9000	22	41.70	2.2000	−1.30	[−1.96; −0.65]	1.5%	2.7%
Umut Altay 2013	24	33.00	9.5000	24	35.00	9.5000	−0.21	[−0.77; 0.36]	2.0%	2.7%
Al Habashneh R 2014	20	16.00	41.0000	20	36.00	51.0000	−0.42	[−1.05; 0.20]	1.6%	2.7%
Al Habashneh R 2014	21	13.00	28.0000	21	54.00	75.0000	−0.71	[−1.34; −0.09]	1.7%	2.7%
Renukanth Patabi Cheta Raman 2014	15	70.00	134.0000	15	105.00	158.0000	−0.23	[−0.95; 0.49]	1.3%	2.7%
Fang F 2015	48	22.40	16.1000	48	30.10	17.0000	−0.46	[−0.87; −0.06]	3.9%	2.8%
Deepti 2017	26	39.50	3.5000	26	34.20	8.6000	0.80	[0.23; 1.36]	2.0%	2.7%
Kapellas 2016	24	41.00	37.0000	24	61.00	68.0000	−0.36	[−0.93; 0.21]	2.0%	2.7%
**Common effect model**	**367**			**367**			**−0.29**	**[−0.44; −0.15]**	**29.8%**	**.**
**Random effects model**							**−0.29**	**[−0.52; −0.05]**	**.**	**38.5%**
Heterogeneity: $I^2 = 59\%$, $\tau^2 = 0.1221$, $p < 0.01$										
**Days after treatment:180**										
Offenbacher 2009	126	31.20	3.8000	126	31.70	3.9000	−0.13	[−0.38; 0.12]	10.6%	2.8%
Panagiotis A. Koromantzos 2012	26	10.80	13.3000	26	15.20	9.7000	−0.37	[−0.92; 0.18]	2.1%	2.7%
Lei Chen 2012	42	15.80	13.1000	42	32.10	44.5000	−0.49	[−0.93; −0.06]	3.4%	2.8%
Fang F 2015	48	22.30	15.0000	48	30.10	17.0000	−0.48	[−0.89; −0.08]	3.9%	2.8%
Gaetano Isola 2023	23	365.00	10.0000	23	417.00	10.0000	−5.11	[−6.34; −3.88]	0.4%	2.7%
Gaetano Isola 2023	23	331.00	23.0000	23	421.00	19.0000	−4.19	[−5.26; −3.13]	0.6%	2.7%
Deepti 2017	26	53.90	18.9000	26	34.20	8.6000	1.32	[0.72; 1.93]	1.8%	2.7%
Geisinger 2016	240	56.30	51.6000	240	49.80	58.8000	0.12	[−0.06; 0.30]	20.1%	2.8%
**Common effect model**	**554**			**554**			**−0.13**	**[−0.25; −0.01]**	**43.0%**	**.**
**Random effects model**							**−1.11**	**[−2.62; 0.39]**	**.**	**21.9%**
Heterogeneity: $I^2 = 95\%$, $\tau^2 = 4.5999$, $p < 0.01$										
**Common effect model**	**1351**			**1351**			**−0.27**	**[−0.36; −0.19]**	**100.0%**	**.**
**Random effects model**							**−1.17**	**[−2.18; −0.16]**	**.**	**100.0%**

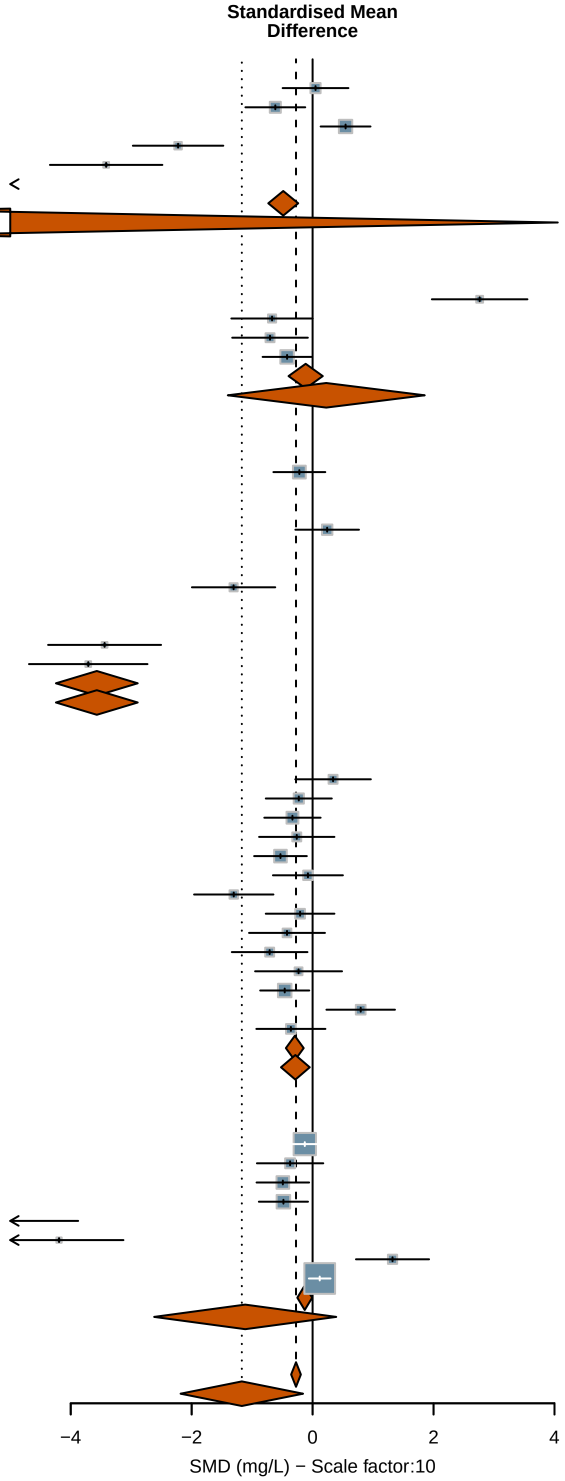

Heterogeneity: $I^2 = 94\%$, $\tau^2 = 9.6291$, $p < 0.01$
Test for subgroup differences (common effect): $\chi^2_7 = 113.02$, df = 7 ($p < 0.01$)
Test for subgroup differences (random effects): $\chi^2_7 = 99.44$, df = 7 ($p < 0.01$)

SMD: −0.27; 95%C.I.[−0.36; −0.19] P value for common effect= 0

SMD: −1.17; 95%C.I.[−2.18; −0.16] P value for random effect= 0.0235

Cytokine: hs−CRP − Treatment: Intensive

Study	After TRT Total	After TRT Mean	After TRT SD	Before TRT Total	Before TRT Mean	Before TRT SD	SMD	95%−CI	Weight (common)	Weight (random)
**MonthsAfterTRT:4−6Wks**										
Steven Offenbacher 2006	25	185.40	45.0000	25	89.00	18.3000	2.76	[1.97; 3.55]	1.0%	2.7%
Tuter G 2017	18	27.70	22.6000	18	49.60	39.1000	−0.67	[−1.34; 0.00]	1.4%	2.7%
Gulay Tuter 2010	21	6.60	1.9000	21	8.00	2.0000	−0.70	[−1.33; −0.08]	1.7%	2.7%
Panagiotis A. Koromantzos 2012	26	15.70	10.7000	26	15.20	9.7000	0.05	[−0.50; 0.59]	2.2%	2.7%
Fang F 2015	48	23.40	14.4000	48	30.10	17.0000	−0.42	[−0.83; −0.02]	3.9%	2.8%
Yasuichi Munenaga 2013	33	9.10	8.6000	33	18.30	19.0000	−0.62	[−1.11; −0.12]	2.6%	2.8%
Yasuichi Munenaga 2013	47	3.80	3.7000	47	2.30	1.1000	0.55	[0.13; 0.96]	3.8%	2.8%
Gaetano Isola 2023	23	392.00	12.0000	23	417.00	10.0000	−2.22	[−2.97; −1.48]	1.2%	2.7%
Gaetano Isola 2023	23	355.00	19.0000	23	421.00	19.0000	−3.41	[−4.34; −2.48]	0.7%	2.7%
Bansari Shah 2022	30	10.14	0.1414	30	19.45	0.3540	−34.09	[−40.41; −27.77]	0.0%	1.3%
**Common effect model**	**294**			**294**			**−0.33**	**[−0.51; −0.14]**	**18.6%**	**.**
**Random effects model**							**−3.59**	**[−9.74; 2.56]**	**.**	**26.0%**
Heterogeneity: $I^2 = 97\%$, $\tau^2 = 97.4467$, $p < 0.01$										
**MonthsAfterTRT:Within 3 Mo.**										
Stefan Renvert	28	57.40	85.3000	28	41.90	28.0000	0.24	[−0.29; 0.77]	2.3%	2.8%
O fentog lu 2010	20	114.00	171.0000	20	63.00	120.0000	0.34	[−0.29; 0.96]	1.7%	2.7%
Panagiotis A. Koromantzos 2012	26	12.50	13.3000	26	15.20	9.7000	−0.23	[−0.77; 0.32]	2.2%	2.7%
Eduardo Machado Vilela 2011	36	32.80	21.0000	36	40.40	24.0000	−0.33	[−0.80; 0.13]	3.0%	2.8%
Eduardo Machado Vilela 2011	20	22.00	23.0000	20	30.40	38.0000	−0.26	[−0.88; 0.36]	1.7%	2.7%
Lei Chen 2012	42	23.80	28.8000	42	32.10	44.5000	−0.22	[−0.65; 0.21]	3.5%	2.8%
Lei Chen 2012	42	14.80	10.0000	42	32.10	44.5000	−0.53	[−0.97; −0.10]	3.4%	2.8%
Fernando Llambes 2012	23	18.80	15.2000	23	20.00	15.2000	−0.08	[−0.66; 0.50]	1.9%	2.7%
Umut Altay 2013	22	34.00	7.9000	22	41.70	2.2000	−1.30	[−1.96; −0.65]	1.5%	2.7%
Umut Altay 2013	24	33.00	9.5000	24	35.00	9.5000	−0.21	[−0.77; 0.36]	2.0%	2.7%
Pradeep Koppolu 2013	20	2.90	1.2000	20	4.50	1.2000	−1.31	[−2.00; −0.62]	1.4%	2.7%
Al Habashneh R 2014	20	16.00	41.0000	20	36.00	51.0000	−0.42	[−1.05; 0.20]	1.6%	2.7%
Al Habashneh R 2014	21	13.00	28.0000	21	54.00	75.0000	−0.71	[−1.34; −0.09]	1.7%	2.7%
Renukanth Patabi Cheta Raman 2014	15	70.00	134.0000	15	105.00	158.0000	−0.23	[−0.95; 0.49]	1.3%	2.7%
Fang F 2015	48	22.40	16.1000	48	30.10	17.0000	−0.46	[−0.87; −0.06]	3.9%	2.8%
Gaetano Isola 2023	23	382.00	10.0000	23	417.00	10.0000	−3.44	[−4.37; −2.51]	0.7%	2.7%
Gaetano Isola 2023	23	358.00	14.0000	23	421.00	19.0000	−3.71	[−4.69; −2.73]	0.7%	2.7%
Deepti 2017	26	39.50	3.5000	26	34.20	8.6000	0.80	[0.23; 1.36]	2.0%	2.7%
Kapellas 2016	24	41.00	37.0000	24	61.00	68.0000	−0.36	[−0.93; 0.21]	2.0%	2.7%
**Common effect model**	**503**			**503**			**−0.41**	**[−0.54; −0.28]**	**38.4%**	**.**
**Random effects model**							**−0.61**	**[−1.08; −0.14]**	**.**	**52.1%**
Heterogeneity: $I^2 = 86\%$, $\tau^2 = 0.9980$, $p < 0.01$										
**MonthsAfterTRT:Within 6 Mo.**										
Offenbacher 2009	126	31.20	3.8000	126	31.70	3.9000	−0.13	[−0.38; 0.12]	10.6%	2.8%
Panagiotis A. Koromantzos 2012	26	10.80	13.3000	26	15.20	9.7000	−0.37	[−0.92; 0.18]	2.1%	2.7%
Lei Chen 2012	42	15.80	13.1000	42	32.10	44.5000	−0.49	[−0.93; −0.06]	3.4%	2.8%
Fang F 2015	48	22.30	15.0000	48	30.10	17.0000	−0.48	[−0.89; −0.08]	3.9%	2.8%
Gaetano Isola 2023	23	365.00	10.0000	23	417.00	10.0000	−5.11	[−6.34; −3.88]	0.4%	2.7%
Gaetano Isola 2023	23	331.00	23.0000	23	421.00	19.0000	−4.19	[−5.26; −3.13]	0.6%	2.7%
Deepti 2017	26	53.90	18.9000	26	34.20	8.6000	1.32	[0.72; 1.93]	1.8%	2.7%
Geisinger 2016	240	56.30	51.6000	240	49.80	58.8000	0.12	[−0.06; 0.30]	20.1%	2.8%
**Common effect model**	**554**			**554**			**−0.13**	**[−0.25; −0.01]**	**43.0%**	**.**
**Random effects model**							**−1.11**	**[−2.62; 0.39]**	**.**	**21.9%**
Heterogeneity: $I^2 = 95\%$, $\tau^2 = 4.5999$, $p < 0.01$										
**Common effect model**	**1351**			**1351**			**−0.27**	**[−0.36; −0.19]**	**100.0%**	**.**
**Random effects model**							**−1.17**	**[−2.18; −0.16]**	**.**	**100.0%**

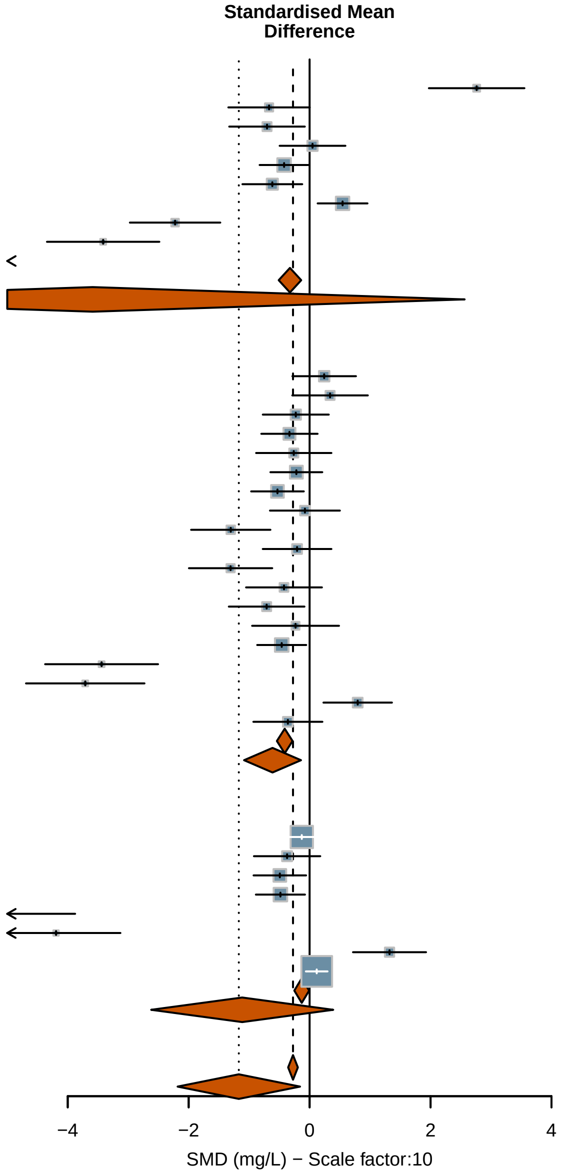

Heterogeneity: $I^2 = 94\%$, $\tau^2 = 9.6291$, $p < 0.01$
Test for subgroup differences (common effect): $\chi^2_2 = 9.89$, df = 2 ($p < 0.01$)
Test for subgroup differences (random effects): $\chi^2_2 = 1.26$, df = 2 ($p = 0.53$)

SMD: −0.27; 95%C.I.[−0.36; −0.19] P value for common effect= 0

SMD: −1.17; 95%C.I.[−2.18; −0.16] P value for random effect= 0.0235

Cytokine: hs−CRP − Treatment: Intensive

Study	After TRT Total	After TRT Mean	After TRT SD	Before TRT Total	Before TRT Mean	Before TRT SD	SMD	95%−CI	Weight (common)	Weight (random)
**Assay type:ELISA**										
Eduardo Machado Vilela 2011	36	32.80	21.0000	36	40.40	24.0000	−0.33	[−0.80; 0.13]	3.0%	2.8%
Eduardo Machado Vilela 2011	20	22.00	23.0000	20	30.40	38.0000	−0.26	[−0.88; 0.36]	1.7%	2.7%
Lei Chen 2012	42	23.80	28.8000	42	32.10	44.5000	−0.22	[−0.65; 0.21]	3.5%	2.8%
Lei Chen 2012	42	14.80	10.0000	42	32.10	44.5000	−0.53	[−0.97; −0.10]	3.4%	2.8%
Lei Chen 2012	42	15.80	13.1000	42	32.10	44.5000	−0.49	[−0.93; −0.06]	3.4%	2.8%
Umut Altay 2013	22	34.00	7.9000	22	41.70	2.2000	−1.30	[−1.96; −0.65]	1.5%	2.7%
Umut Altay 2013	24	33.00	9.5000	24	35.00	9.5000	−0.21	[−0.77; 0.36]	2.0%	2.7%
Pradeep Koppolu 2013	20	2.90	1.2000	20	4.50	1.2000	−1.31	[−2.00; −0.62]	1.4%	2.7%
Al Habashneh R 2014	20	16.00	41.0000	20	36.00	51.0000	−0.42	[−1.05; 0.20]	1.6%	2.7%
Al Habashneh R 2014	21	13.00	28.0000	21	54.00	75.0000	−0.71	[−1.34; −0.09]	1.7%	2.7%
Renukanth Patabi Cheta Raman 2014	15	70.00	134.0000	15	105.00	158.0000	−0.23	[−0.95; 0.49]	1.3%	2.7%
**Common effect model**	**304**			**304**			**−0.49**	**[−0.65; −0.33]**	**24.4%**	**.**
**Random effects model**							**−0.51**	**[−0.71; −0.31]**	**.**	**30.2%**
Heterogeneity: I^2 = 37%, τ^2 = 0.0326, p = 0.11										
**Assay type:immunoturbidimetic assay**										
Stefan Renvert	28	57.40	85.3000	28	41.90	28.0000	0.24	[−0.29; 0.77]	2.3%	2.8%
Fernando Llambes 2012	23	18.80	15.2000	23	20.00	15.2000	−0.08	[−0.66; 0.50]	1.9%	2.7%
Fang F 2015	48	23.40	14.4000	48	30.10	17.0000	−0.42	[−0.83; −0.02]	3.9%	2.8%
Fang F 2015	48	22.40	16.1000	48	30.10	17.0000	−0.46	[−0.87; −0.06]	3.9%	2.8%
Fang F 2015	48	22.30	15.0000	48	30.10	17.0000	−0.48	[−0.89; −0.08]	3.9%	2.8%
Bansari Shah 2022	30	10.14	0.1414	30	19.45	0.3540	−34.09	[−40.41; −27.77]	0.0%	1.3%
**Common effect model**	**225**			**225**			**−0.34**	**[−0.54; −0.14]**	**16.1%**	**.**
**Random effects model**							**−5.62**	**[−16.26; 5.02]**	**.**	**15.1%**
Heterogeneity: I^2 = 96%, τ^2 = 175.1468, p < 0.01										
**Assay type:Konelab Clinical Chemistry Analyzer**										
Deepti 2017	26	39.50	3.5000	26	34.20	8.6000	0.80	[0.23; 1.36]	2.0%	2.7%
Deepti 2017	26	53.90	18.9000	26	34.20	8.6000	1.32	[0.72; 1.93]	1.8%	2.7%
**Common effect model**	**52**			**52**			**1.04**	**[0.63; 1.45]**	**3.8%**	**.**
**Random effects model**							**1.05**	**[0.53; 1.56]**	**.**	**5.5%**
Heterogeneity: I^2 = 36%, τ^2 = 0.0493, p = 0.21										
**Assay type:latex−enhanced nephelometry**										
Steven Offenbacher 2006	25	185.40	45.0000	25	89.00	18.3000	2.76	[1.97; 3.55]	1.0%	2.7%
Tuter G 2017	18	27.70	22.6000	18	49.60	39.1000	−0.67	[−1.34; 0.00]	1.4%	2.7%
Offenbacher 2009	126	31.20	3.8000	126	31.70	3.9000	−0.13	[−0.38; 0.12]	10.6%	2.8%
Gulay Tuter 2010	21	6.60	1.9000	21	8.00	2.0000	−0.70	[−1.33; −0.08]	1.7%	2.7%
O fentog lu 2010	20	114.00	171.0000	20	63.00	120.0000	0.34	[−0.29; 0.96]	1.7%	2.7%
Yasuichi Munenaga 2013	33	9.10	8.6000	33	18.30	19.0000	−0.62	[−1.11; −0.12]	2.6%	2.8%
Yasuichi Munenaga 2013	47	3.80	3.7000	47	2.30	1.1000	0.55	[0.13; 0.96]	3.8%	2.8%
**Common effect model**	**290**			**290**			**0.02**	**[−0.15; 0.18]**	**22.8%**	**.**
**Random effects model**							**0.20**	**[−0.68; 1.08]**	**.**	**19.2%**
Heterogeneity: I^2 = 91%, τ^2 = 1.3100, p < 0.01										
**Assay type:latex−particle enhanced immunoturbidimetric assay**										
Geisinger 2016	240	56.30	51.6000	240	49.80	58.8000	0.12	[−0.06; 0.30]	20.1%	2.8%
**Assay type:nephelometric assay kit**										
Gaetano Isola 2023	23	392.00	12.0000	23	417.00	10.0000	−2.22	[−2.97; −1.48]	1.2%	2.7%
Gaetano Isola 2023	23	382.00	10.0000	23	417.00	10.0000	−3.44	[−4.37; −2.51]	0.7%	2.7%
Gaetano Isola 2023	23	365.00	10.0000	23	417.00	10.0000	−5.11	[−6.34; −3.88]	0.4%	2.7%
Gaetano Isola 2023	23	355.00	19.0000	23	421.00	19.0000	−3.41	[−4.34; −2.48]	0.7%	2.7%
Gaetano Isola 2023	23	358.00	14.0000	23	421.00	19.0000	−3.71	[−4.69; −2.73]	0.7%	2.7%
Gaetano Isola 2023	23	331.00	23.0000	23	421.00	19.0000	−4.19	[−5.26; −3.13]	0.6%	2.7%
**Common effect model**	**138**			**138**			**−3.42**	**[−3.80; −3.03]**	**4.3%**	**.**
**Random effects model**							**−3.61**	**[−4.37; −2.85]**	**.**	**16.2%**
Heterogeneity: I^2 = 74%, τ^2 = 0.6541, p < 0.01										
**Assay type:particle−enhanced immunonephelometry**										
Kapellas 2016	24	41.00	37.0000	24	61.00	68.0000	−0.36	[−0.93; 0.21]	2.0%	2.7%
**Assay type:PCR**										
Panagiotis A. Koromantzos 2012	26	15.70	10.7000	26	15.20	9.7000	0.05	[−0.50; 0.59]	2.2%	2.7%
Panagiotis A. Koromantzos 2012	26	12.50	13.3000	26	15.20	9.7000	−0.23	[−0.77; 0.32]	2.2%	2.7%
Panagiotis A. Koromantzos 2012	26	10.80	13.3000	26	15.20	9.7000	−0.37	[−0.92; 0.18]	2.1%	2.7%
**Common effect model**	**78**			**78**			**−0.18**	**[−0.50; 0.13]**	**6.5%**	**.**
**Random effects model**							**−0.18**	**[−0.50; 0.13]**	**.**	**8.2%**
Heterogeneity: I^2 = 0%, τ^2 = 0, p = 0.55										
**Common effect model**	**1351**			**1351**			**−0.27**	**[−0.36; −0.19]**	**100.0%**	**.**
**Random effects model**							**−1.17**	**[−2.18; −0.16]**	**.**	**100.0%**

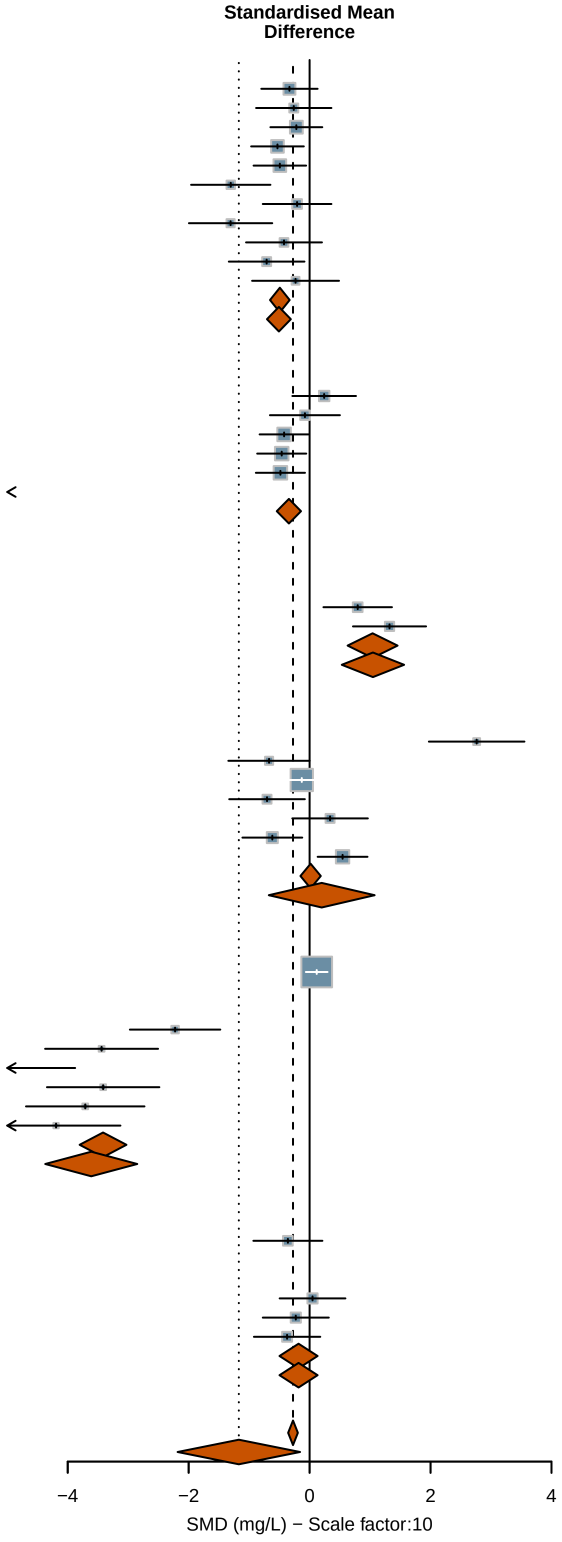

Heterogeneity: I^2 = 94%, τ^2 = 9.6291, p < 0.01
Test for subgroup differences (common effect): χ^2_7 = 329.61, df = 7 (p < 0.01)
Test for subgroup differences (random effects): χ^2_7 = 123.13, df = 7 (p < 0.01)

SMD: −0.27; 95%C.I.[−0.36; −0.19] P value for common effect= 0

SMD: −1.17; 95%C.I.[−2.18; −0.16] P value for random effect= 0.0235

Cytokine: hs−CRP − Treatment: Intensive

Study	After TRT Total	After TRT Mean	After TRT SD	Before TRT Total	Before TRT Mean	Before TRT SD	SMD	95%−CI	Weight (common)	Weight (random)
**Bias judgment:High risk**										
Steven Offenbacher 2006	25	185.40	45.0000	25	89.00	18.3000	2.76	[1.97; 3.55]	1.0%	2.7%
Umut Altay 2013	22	34.00	7.9000	22	41.70	2.2000	−1.30	[−1.96; −0.65]	1.5%	2.7%
Umut Altay 2013	24	33.00	9.5000	24	35.00	9.5000	−0.21	[−0.77; 0.36]	2.0%	2.7%
Pradeep Koppolu 2013	20	2.90	1.2000	20	4.50	1.2000	−1.31	[−2.00; −0.62]	1.4%	2.7%
Renukanth Patabi Cheta Raman 2014	15	70.00	134.0000	15	105.00	158.0000	−0.23	[−0.95; 0.49]	1.3%	2.7%
Gaetano Isola 2023	23	392.00	12.0000	23	417.00	10.0000	−2.22	[−2.97; −1.48]	1.2%	2.7%
Gaetano Isola 2023	23	382.00	10.0000	23	417.00	10.0000	−3.44	[−4.37; −2.51]	0.7%	2.7%
Gaetano Isola 2023	23	365.00	10.0000	23	417.00	10.0000	−5.11	[−6.34; −3.88]	0.4%	2.7%
Gaetano Isola 2023	23	355.00	19.0000	23	421.00	19.0000	−3.41	[−4.34; −2.48]	0.7%	2.7%
Gaetano Isola 2023	23	358.00	14.0000	23	421.00	19.0000	−3.71	[−4.69; −2.73]	0.7%	2.7%
Gaetano Isola 2023	23	331.00	23.0000	23	421.00	19.0000	−4.19	[−5.26; −3.13]	0.6%	2.7%
Kapellas 2016	24	41.00	37.0000	24	61.00	68.0000	−0.36	[−0.93; 0.21]	2.0%	2.7%
Bansari Shah 2022	30	10.14	0.1414	30	19.45	0.3540	−34.09	[−40.41; −27.77]	0.0%	1.3%
**Common effect model**	**298**			**298**			**−1.31**	**[−1.52; −1.09]**	**13.5%**	**.**
**Random effects model**							**−4.06**	**[−8.51; 0.39]**	**.**	**34.0%**
Heterogeneity: $I^2 = 97\%$, $\tau^2 = 66.1501$, $p < 0.01$										
**Bias judgment:Low risk**										
Tuter G 2017	18	27.70	22.6000	18	49.60	39.1000	−0.67	[−1.34; 0.00]	1.4%	2.7%
O fentog lu 2010	20	114.00	171.0000	20	63.00	120.0000	0.34	[−0.29; 0.96]	1.7%	2.7%
Panagiotis A. Koromantzos 2012	26	15.70	10.7000	26	15.20	9.7000	0.05	[−0.50; 0.59]	2.2%	2.7%
Panagiotis A. Koromantzos 2012	26	12.50	13.3000	26	15.20	9.7000	−0.23	[−0.77; 0.32]	2.2%	2.7%
Panagiotis A. Koromantzos 2012	26	10.80	13.3000	26	15.20	9.7000	−0.37	[−0.92; 0.18]	2.1%	2.7%
Eduardo Machado Vilela 2011	36	32.80	21.0000	36	40.40	24.0000	−0.33	[−0.80; 0.13]	3.0%	2.8%
Eduardo Machado Vilela 2011	20	22.00	23.0000	20	30.40	38.0000	−0.26	[−0.88; 0.36]	1.7%	2.7%
Fang F 2015	48	23.40	14.4000	48	30.10	17.0000	−0.42	[−0.83; −0.02]	3.9%	2.8%
Fang F 2015	48	22.40	16.1000	48	30.10	17.0000	−0.46	[−0.87; −0.06]	3.9%	2.8%
Fang F 2015	48	22.30	15.0000	48	30.10	17.0000	−0.48	[−0.89; −0.08]	3.9%	2.8%
Yasuichi Munenaga 2013	33	9.10	8.6000	33	18.30	19.0000	−0.62	[−1.11; −0.12]	2.6%	2.8%
Yasuichi Munenaga 2013	47	3.80	3.7000	47	2.30	1.1000	0.55	[0.13; 0.96]	3.8%	2.8%
**Common effect model**	**396**			**396**			**−0.24**	**[−0.39; −0.10]**	**32.5%**	**.**
**Random effects model**							**−0.24**	**[−0.46; −0.03]**	**.**	**33.0%**
Heterogeneity: $I^2 = 57\%$, $\tau^2 = 0.0812$, $p < 0.01$										
**Bias judgment:Some concerns**										
Offenbacher 2009	126	31.20	3.8000	126	31.70	3.9000	−0.13	[−0.38; 0.12]	10.6%	2.8%
Stefan Renvert	28	57.40	85.3000	28	41.90	28.0000	0.24	[−0.29; 0.77]	2.3%	2.8%
Gulay Tuter 2010	21	6.60	1.9000	21	8.00	2.0000	−0.70	[−1.33; −0.08]	1.7%	2.7%
Lei Chen 2012	42	23.80	28.8000	42	32.10	44.5000	−0.22	[−0.65; 0.21]	3.5%	2.8%
Lei Chen 2012	42	14.80	10.0000	42	32.10	44.5000	−0.53	[−0.97; −0.10]	3.4%	2.8%
Lei Chen 2012	42	15.80	13.1000	42	32.10	44.5000	−0.49	[−0.93; −0.06]	3.4%	2.8%
Fernando Llambes 2012	23	18.80	15.2000	23	20.00	15.2000	−0.08	[−0.66; 0.50]	1.9%	2.7%
Al Habashneh R 2014	20	16.00	41.0000	20	36.00	51.0000	−0.42	[−1.05; 0.20]	1.6%	2.7%
Al Habashneh R 2014	21	13.00	28.0000	21	54.00	75.0000	−0.71	[−1.34; −0.09]	1.7%	2.7%
Deepti 2017	26	39.50	3.5000	26	34.20	8.6000	0.80	[0.23; 1.36]	2.0%	2.7%
Deepti 2017	26	53.90	18.9000	26	34.20	8.6000	1.32	[0.72; 1.93]	1.8%	2.7%
Geisinger 2016	240	56.30	51.6000	240	49.80	58.8000	0.12	[−0.06; 0.30]	20.1%	2.8%
**Common effect model**	**657**			**657**			**−0.04**	**[−0.15; 0.07]**	**54.1%**	**.**
**Random effects model**							**−0.07**	**[−0.40; 0.26]**	**.**	**33.0%**
Heterogeneity: $I^2 = 79\%$, $\tau^2 = 0.2682$, $p < 0.01$										
**Common effect model**	**1351**			**1351**			**−0.27**	**[−0.36; −0.19]**	**100.0%**	**.**
**Random effects model**							**−1.17**	**[−2.18; −0.16]**	**.**	**100.0%**

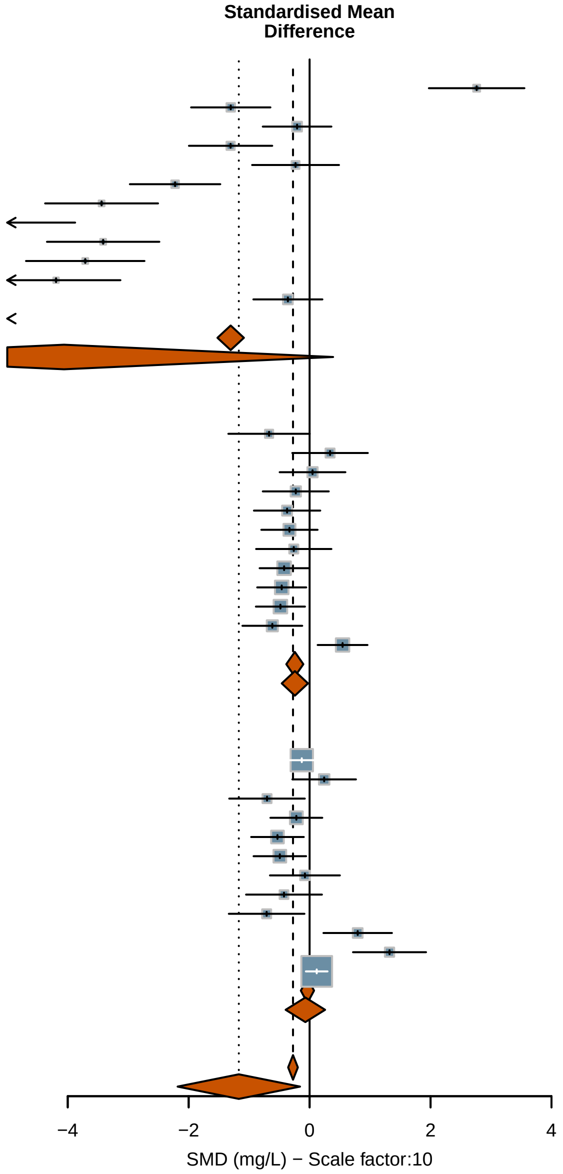

Heterogeneity: $I^2 = 94\%$, $\tau^2 = 9.6291$, $p < 0.01$
Test for subgroup differences (common effect): $\chi^2_2 = 103.53$, df = 2 ($p < 0.01$)
Test for subgroup differences (random effects): $\chi^2_2 = 3.65$, df = 2 ($p = 0.16$)

SMD: −0.27; 95%C.I.[−0.36; −0.19] P value for common effect= 0

SMD: −1.17; 95%C.I.[−2.18; −0.16] P value for random effect= 0.0235

Cytokine: hs−CRP − Treatment: Intensive

Study	After TRT Total	After TRT Mean	After TRT SD	Before TRT Total	Before TRT Mean	Before TRT SD	SMD	95%−CI	Weight (common)	Weight (random)
**Study design:clinical trial**										
Renukanth Patabi Cheta Raman 2014	15	70.00	134.0000	15	105.00	158.0000	−0.23	[−0.95; 0.49]	1.3%	2.7%
**Study design:Comparative study**										
O fentog lu 2010	20	114.00	171.0000	20	63.00	120.0000	0.34	[−0.29; 0.96]	1.7%	2.7%
Umut Altay 2013	22	34.00	7.9000	22	41.70	2.2000	−1.30	[−1.96; −0.65]	1.5%	2.7%
Umut Altay 2013	24	33.00	9.5000	24	35.00	9.5000	−0.21	[−0.77; 0.36]	2.0%	2.7%
Yasuichi Munenaga 2013	33	9.10	8.6000	33	18.30	19.0000	−0.62	[−1.11; −0.12]	2.6%	2.8%
Yasuichi Munenaga 2013	47	3.80	3.7000	47	2.30	1.1000	0.55	[0.13; 0.96]	3.8%	2.8%
**Common effect model**	**146**			**146**			**−0.12**	**[−0.35; 0.12]**	**11.6%**	**.**
**Random effects model**							**−0.23**	**[−0.88; 0.41]**	**.**	**13.7%**
Heterogeneity: I^2 = 86%, τ^2 = 0.4641, p < 0.01										
**Study design:Interventional controlled non−RCT**										
Eduardo Machado Vilela 2011	36	32.80	21.0000	36	40.40	24.0000	−0.33	[−0.80; 0.13]	3.0%	2.8%
Eduardo Machado Vilela 2011	20	22.00	23.0000	20	30.40	38.0000	−0.26	[−0.88; 0.36]	1.7%	2.7%
**Common effect model**	**56**			**56**			**−0.31**	**[−0.68; 0.06]**	**4.6%**	**.**
**Random effects model**							**−0.31**	**[−0.68; 0.06]**	**.**	**5.5%**
Heterogeneity: I^2 = 0%, τ^2 = 0, p = 0.86										
**Study design:Randomized blinded clinical trial**										
Al Habashneh R 2014	20	16.00	41.0000	20	36.00	51.0000	−0.42	[−1.05; 0.20]	1.6%	2.7%
Al Habashneh R 2014	21	13.00	28.0000	21	54.00	75.0000	−0.71	[−1.34; −0.09]	1.7%	2.7%
**Common effect model**	**41**			**41**			**−0.57**	**[−1.01; −0.12]**	**3.3%**	**.**
**Random effects model**							**−0.57**	**[−1.01; −0.12]**	**.**	**5.5%**
Heterogeneity: I^2 = 0%, τ^2 = 0, p = 0.53										
**Study design:RCT**										
Steven Offenbacher 2006	25	185.40	45.0000	25	89.00	18.3000	2.76	[1.97; 3.55]	1.0%	2.7%
Tuter G 2017	18	27.70	22.6000	18	49.60	39.1000	−0.67	[−1.34; 0.00]	1.4%	2.7%
Offenbacher 2009	126	31.20	3.8000	126	31.70	3.9000	−0.13	[−0.38; 0.12]	10.6%	2.8%
Stefan Renvert	28	57.40	85.3000	28	41.90	28.0000	0.24	[−0.29; 0.77]	2.3%	2.8%
Gulay Tuter 2010	21	6.60	1.9000	21	8.00	2.0000	−0.70	[−1.33; −0.08]	1.7%	2.7%
Panagiotis A. Koromantzos 2012	26	15.70	10.7000	26	15.20	9.7000	0.05	[−0.50; 0.59]	2.2%	2.7%
Panagiotis A. Koromantzos 2012	26	12.50	13.3000	26	15.20	9.7000	−0.23	[−0.77; 0.32]	2.2%	2.7%
Panagiotis A. Koromantzos 2012	26	10.80	13.3000	26	15.20	9.7000	−0.37	[−0.92; 0.18]	2.1%	2.7%
Lei Chen 2012	42	23.80	28.8000	42	32.10	44.5000	−0.22	[−0.65; 0.21]	3.5%	2.8%
Lei Chen 2012	42	14.80	10.0000	42	32.10	44.5000	−0.53	[−0.97; −0.10]	3.4%	2.8%
Lei Chen 2012	42	15.80	13.1000	42	32.10	44.5000	−0.49	[−0.93; −0.06]	3.4%	2.8%
Fernando Llambes 2012	23	18.80	15.2000	23	20.00	15.2000	−0.08	[−0.66; 0.50]	1.9%	2.7%
Pradeep Koppolu 2013	20	2.90	1.2000	20	4.50	1.2000	−1.31	[−2.00; −0.62]	1.4%	2.7%
Fang F 2015	48	23.40	14.4000	48	30.10	17.0000	−0.42	[−0.83; −0.02]	3.9%	2.8%
Fang F 2015	48	22.40	16.1000	48	30.10	17.0000	−0.46	[−0.87; −0.06]	3.9%	2.8%
Fang F 2015	48	22.30	15.0000	48	30.10	17.0000	−0.48	[−0.89; −0.08]	3.9%	2.8%
Gaetano Isola 2023	23	392.00	12.0000	23	417.00	10.0000	−2.22	[−2.97; −1.48]	1.2%	2.7%
Gaetano Isola 2023	23	382.00	10.0000	23	417.00	10.0000	−3.44	[−4.37; −2.51]	0.7%	2.7%
Gaetano Isola 2023	23	365.00	10.0000	23	417.00	10.0000	−5.11	[−6.34; −3.88]	0.4%	2.7%
Gaetano Isola 2023	23	355.00	19.0000	23	421.00	19.0000	−3.41	[−4.34; −2.48]	0.7%	2.7%
Gaetano Isola 2023	23	358.00	14.0000	23	421.00	19.0000	−3.71	[−4.69; −2.73]	0.7%	2.7%
Gaetano Isola 2023	23	331.00	23.0000	23	421.00	19.0000	−4.19	[−5.26; −3.13]	0.6%	2.7%
Deepti 2017	26	39.50	3.5000	26	34.20	8.6000	0.80	[0.23; 1.36]	2.0%	2.7%
Deepti 2017	26	53.90	18.9000	26	34.20	8.6000	1.32	[0.72; 1.93]	1.8%	2.7%
Kapellas 2016	24	41.00	37.0000	24	61.00	68.0000	−0.36	[−0.93; 0.21]	2.0%	2.7%
Geisinger 2016	240	56.30	51.6000	240	49.80	58.8000	0.12	[−0.06; 0.30]	20.1%	2.8%
**Common effect model**	**1063**			**1063**			**−0.28**	**[−0.37; −0.19]**	**79.2%**	**.**
**Random effects model**							**−0.85**	**[−1.51; −0.20]**	**.**	**71.2%**
Heterogeneity: I^2 = 94%, τ^2 = 2.8189, p < 0.01										
**Study design:Single arm Prospective clinical trial**										
Bansari Shah 2022	30	10.14	0.1414	30	19.45	0.3540	−34.09	[−40.41; −27.77]	0.0%	1.3%
**Common effect model**	**1351**			**1351**			**−0.27**	**[−0.36; −0.19]**	**100.0%**	**.**
**Random effects model**							**−1.17**	**[−2.18; −0.16]**	**.**	**100.0%**

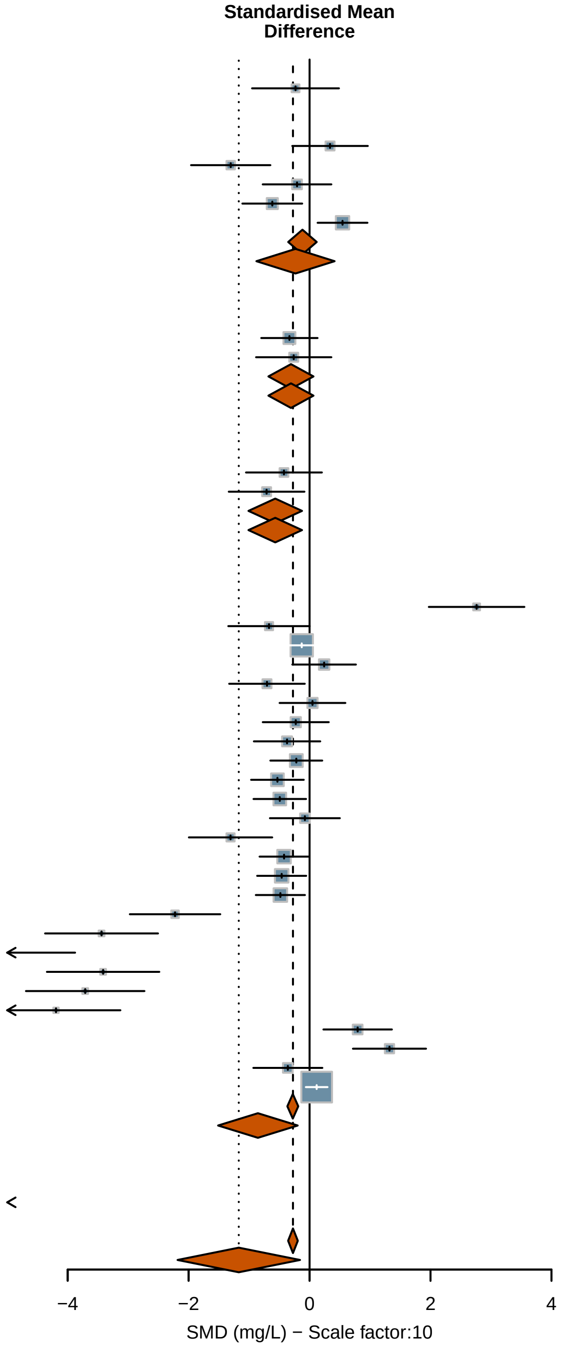

Heterogeneity: I^2 = 94%, τ^2 = 9.6291, p < 0.01
Test for subgroup differences (common effect): χ^2_5 = 113.42, df = 5 (p < 0.01)
Test for subgroup differences (random effects): χ^2_5 = 111.89, df = 5 (p < 0.01)

SMD: −0.27; 95%C.I.[−0.36; −0.19] P value for common effect= 0

SMD: −1.17; 95%C.I.[−2.18; −0.16] P value for random effect= 0.0235

Cytokine: hs−CRP − Treatment: Intensive

Study	After TRT Total	After TRT Mean	After TRT SD	Before TRT Total	Before TRT Mean	Before TRT SD	SMD	95%−CI	Weight (common)	Weight (random)
**Additional_devices:FMD**										
Gaetano Isola 2023	23	392.00	12.0000	23	417.00	10.0000	−2.22	[−2.97; −1.48]	4.4%	12.7%
Gaetano Isola 2023	23	382.00	10.0000	23	417.00	10.0000	−3.44	[−4.37; −2.51]	2.8%	12.4%
Gaetano Isola 2023	23	365.00	10.0000	23	417.00	10.0000	−5.11	[−6.34; −3.88]	1.6%	11.8%
**Common effect model**	**69**			**69**			**−3.14**	**[−3.67; −2.61]**	**8.9%**	**.**
**Random effects model**							**−3.53**	**[−5.15; −1.92]**	**.**	**37.0%**
Heterogeneity: I^2 = 87%, τ^2 = 1.7928, p < 0.01										
**Additional_devices:Ozonetherapy**										
Al Habashneh R 2014	20	16.00	41.0000	20	36.00	51.0000	−0.42	[−1.05; 0.20]	6.3%	12.9%
**Additional_devices:QMD**										
Gaetano Isola 2023	23	355.00	19.0000	23	421.00	19.0000	−3.41	[−4.34; −2.48]	2.9%	12.4%
Gaetano Isola 2023	23	358.00	14.0000	23	421.00	19.0000	−3.71	[−4.69; −2.73]	2.6%	12.3%
Gaetano Isola 2023	23	331.00	23.0000	23	421.00	19.0000	−4.19	[−5.26; −3.13]	2.2%	12.2%
**Common effect model**	**69**			**69**			**−3.74**	**[−4.31; −3.17]**	**7.6%**	**.**
**Random effects model**							**−3.74**	**[−4.31; −3.17]**	**.**	**36.9%**
Heterogeneity: I^2 = 0%, τ^2 = 0, p = 0.56										
**Additional_devices:SPC a 3 mesi**										
Geisinger 2016	240	56.30	51.6000	240	49.80	58.8000	0.12	[−0.06; 0.30]	77.2%	13.2%
**Common effect model**	**398**			**398**			**−0.50**	**[−0.66; −0.34]**	**100.0%**	**.**
**Random effects model**							**−2.75**	**[−4.03; −1.46]**	**.**	**100.0%**

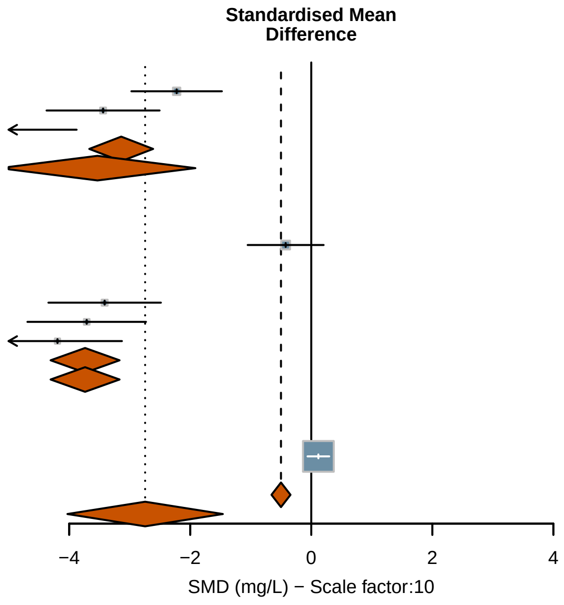

Heterogeneity: I^2 = 98%, τ^2 = 3.2309, p < 0.01
Test for subgroup differences (common effect): χ^2_3 = 266.01, df = 3 (p < 0.01)
Test for subgroup differences (random effects): χ^2_3 = 176.15, df = 3 (p < 0.01)

SMD: −0.5; 95%C.I.[−0.66; −0.34] P value for common effect= 0

SMD: −2.75; 95%C.I.[−4.03; −1.46] P value for random effect= 0

Cytokine: hs−CRP − Treatment: Intensive

Study	After TRT Total	After TRT Mean	After TRT SD	Before TRT Total	Before TRT Mean	Before TRT SD	SMD	95%−CI	Weight (common)	Weight (random)
**Female_binary:Only Female**										
Steven Offenbacher 2006	25	185.40	45.0000	25	89.00	18.3000	2.76	[1.97; 3.55]	1.0%	2.7%
Deepti 2017	26	39.50	3.5000	26	34.20	8.6000	0.80	[0.23; 1.36]	2.0%	2.7%
Deepti 2017	26	53.90	18.9000	26	34.20	8.6000	1.32	[0.72; 1.93]	1.8%	2.7%
**Common effect model**	**77**			**77**			**1.41**	**[1.04; 1.78]**	**4.8%**	**.**
**Random effects model**							**1.60**	**[0.47; 2.73]**	**.**	**8.2%**
Heterogeneity: $I^2 = 87\%$, $\tau^2 = 0.8842$, $p < 0.01$										
**Female_binary:Sex mixed**										
Tuter G 2017	18	27.70	22.6000	18	49.60	39.1000	−0.67	[−1.34; 0.00]	1.4%	2.7%
Offenbacher 2009	126	31.20	3.8000	126	31.70	3.9000	−0.13	[−0.38; 0.12]	10.6%	2.8%
Stefan Renvert	28	57.40	85.3000	28	41.90	28.0000	0.24	[−0.29; 0.77]	2.3%	2.8%
Gulay Tuter 2010	21	6.60	1.9000	21	8.00	2.0000	−0.70	[−1.33; −0.08]	1.7%	2.7%
O fentog lu 2010	20	114.00	171.0000	20	63.00	120.0000	0.34	[−0.29; 0.96]	1.7%	2.7%
Panagiotis A. Koromantzos 2012	26	15.70	10.7000	26	15.20	9.7000	0.05	[−0.50; 0.59]	2.2%	2.7%
Panagiotis A. Koromantzos 2012	26	12.50	13.3000	26	15.20	9.7000	−0.23	[−0.77; 0.32]	2.2%	2.7%
Panagiotis A. Koromantzos 2012	26	10.80	13.3000	26	15.20	9.7000	−0.37	[−0.92; 0.18]	2.1%	2.7%
Eduardo Machado Vilela 2011	36	32.80	21.0000	36	40.40	24.0000	−0.33	[−0.80; 0.13]	3.0%	2.8%
Eduardo Machado Vilela 2011	20	22.00	23.0000	20	30.40	38.0000	−0.26	[−0.88; 0.36]	1.7%	2.7%
Lei Chen 2012	42	23.80	28.8000	42	32.10	44.5000	−0.22	[−0.65; 0.21]	3.5%	2.8%
Lei Chen 2012	42	14.80	10.0000	42	32.10	44.5000	−0.53	[−0.97; −0.10]	3.4%	2.8%
Lei Chen 2012	42	15.80	13.1000	42	32.10	44.5000	−0.49	[−0.93; −0.06]	3.4%	2.8%
Fernando Llambes 2012	23	18.80	15.2000	23	20.00	15.2000	−0.08	[−0.66; 0.50]	1.9%	2.7%
Umut Altay 2013	22	34.00	7.9000	22	41.70	2.2000	−1.30	[−1.96; −0.65]	1.5%	2.7%
Umut Altay 2013	24	33.00	9.5000	24	35.00	9.5000	−0.21	[−0.77; 0.36]	2.0%	2.7%
Pradeep Koppolu 2013	20	2.90	1.2000	20	4.50	1.2000	−1.31	[−2.00; −0.62]	1.4%	2.7%
Al Habashneh R 2014	20	16.00	41.0000	20	36.00	51.0000	−0.42	[−1.05; 0.20]	1.6%	2.7%
Al Habashneh R 2014	21	13.00	28.0000	21	54.00	75.0000	−0.71	[−1.34; −0.09]	1.7%	2.7%
Renukanth Patabi Cheta Raman 2014	15	70.00	134.0000	15	105.00	158.0000	−0.23	[−0.95; 0.49]	1.3%	2.7%
Fang F 2015	48	23.40	14.4000	48	30.10	17.0000	−0.42	[−0.83; −0.02]	3.9%	2.8%
Fang F 2015	48	22.40	16.1000	48	30.10	17.0000	−0.46	[−0.87; −0.06]	3.9%	2.8%
Fang F 2015	48	22.30	15.0000	48	30.10	17.0000	−0.48	[−0.89; −0.08]	3.9%	2.8%
Yasuichi Munenaga 2013	33	9.10	8.6000	33	18.30	19.0000	−0.62	[−1.11; −0.12]	2.6%	2.8%
Yasuichi Munenaga 2013	47	3.80	3.7000	47	2.30	1.1000	0.55	[0.13; 0.96]	3.8%	2.8%
Gaetano Isola 2023	23	392.00	12.0000	23	417.00	10.0000	−2.22	[−2.97; −1.48]	1.2%	2.7%
Gaetano Isola 2023	23	382.00	10.0000	23	417.00	10.0000	−3.44	[−4.37; −2.51]	0.7%	2.7%
Gaetano Isola 2023	23	365.00	10.0000	23	417.00	10.0000	−5.11	[−6.34; −3.88]	0.4%	2.7%
Gaetano Isola 2023	23	355.00	19.0000	23	421.00	19.0000	−3.41	[−4.34; −2.48]	0.7%	2.7%
Gaetano Isola 2023	23	358.00	14.0000	23	421.00	19.0000	−3.71	[−4.69; −2.73]	0.7%	2.7%
Gaetano Isola 2023	23	331.00	23.0000	23	421.00	19.0000	−4.19	[−5.26; −3.13]	0.6%	2.7%
Kapellas 2016	24	41.00	37.0000	24	61.00	68.0000	−0.36	[−0.93; 0.21]	2.0%	2.7%
Geisinger 2016	240	56.30	51.6000	240	49.80	58.8000	0.12	[−0.06; 0.30]	20.1%	2.8%
Bansari Shah 2022	30	10.14	0.1414	30	19.45	0.3540	−34.09	[−40.41; −27.77]	0.0%	1.3%
**Common effect model**	**1274**			**1274**			**−0.36**	**[−0.44; −0.28]**	**95.2%**	**.**
**Random effects model**							**−1.44**	**[−2.55; −0.34]**	**.**	**91.8%**
Heterogeneity: $I^2 = 93\%$, $\tau^2 = 10.5869$, $p < 0.01$										
**Common effect model**	**1351**			**1351**			**−0.27**	**[−0.36; −0.19]**	**100.0%**	**.**
**Random effects model**							**−1.17**	**[−2.18; −0.16]**	**.**	**100.0%**

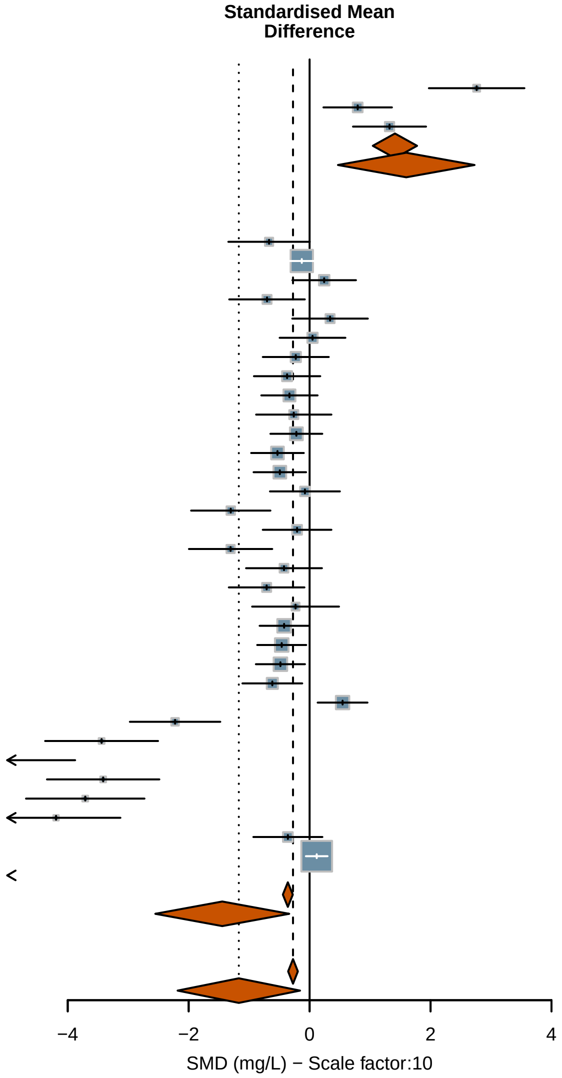

Heterogeneity: $I^2 = 94\%$, $\tau^2 = 9.6291$, $p < 0.01$
Test for subgroup differences (common effect): $\chi^2_1 = 85.54$, df = 1 ($p < 0.01$)
Test for subgroup differences (random effects): $\chi^2_1 = 14.21$, df = 1 ($p < 0.01$)

SMD: −0.27; 95%C.I.[−0.36; −0.19] P value for common effect= 0

SMD: −1.17; 95%C.I.[−2.18; −0.16] P value for random effect= 0.0235

Cytokine: hs−CRP − Treatment: Intensive

Study	After TRT Total	After TRT Mean	After TRT SD	Before TRT Total	Before TRT Mean	Before TRT SD	SMD	95%−CI	Weight (common)	Weight (random)
**Smoke_binary:Sample with smokers**										
Offenbacher 2009	126	31.20	3.8000	126	31.70	3.9000	−0.13	[−0.38; 0.12]	12.5%	3.6%
Stefan Renvert	28	57.40	85.3000	28	41.90	28.0000	0.24	[−0.29; 0.77]	2.8%	3.5%
Panagiotis A. Koromantzos 2012	26	15.70	10.7000	26	15.20	9.7000	0.05	[−0.50; 0.59]	2.6%	3.5%
Panagiotis A. Koromantzos 2012	26	12.50	13.3000	26	15.20	9.7000	−0.23	[−0.77; 0.32]	2.6%	3.5%
Panagiotis A. Koromantzos 2012	26	10.80	13.3000	26	15.20	9.7000	−0.37	[−0.92; 0.18]	2.5%	3.5%
Lei Chen 2012	42	23.80	28.8000	42	32.10	44.5000	−0.22	[−0.65; 0.21]	4.2%	3.5%
Lei Chen 2012	42	14.80	10.0000	42	32.10	44.5000	−0.53	[−0.97; −0.10]	4.0%	3.5%
Lei Chen 2012	42	15.80	13.1000	42	32.10	44.5000	−0.49	[−0.93; −0.06]	4.0%	3.5%
Fernando Llambes 2012	23	18.80	15.2000	23	20.00	15.2000	−0.08	[−0.66; 0.50]	2.3%	3.5%
Umut Altay 2013	22	34.00	7.9000	22	41.70	2.2000	−1.30	[−1.96; −0.65]	1.8%	3.4%
Umut Altay 2013	24	33.00	9.5000	24	35.00	9.5000	−0.21	[−0.77; 0.36]	2.4%	3.5%
Fang F 2015	48	23.40	14.4000	48	30.10	17.0000	−0.42	[−0.83; −0.02]	4.7%	3.5%
Fang F 2015	48	22.40	16.1000	48	30.10	17.0000	−0.46	[−0.87; −0.06]	4.6%	3.5%
Fang F 2015	48	22.30	15.0000	48	30.10	17.0000	−0.48	[−0.89; −0.08]	4.6%	3.5%
Gaetano Isola 2023	23	392.00	12.0000	23	417.00	10.0000	−2.22	[−2.97; −1.48]	1.4%	3.4%
Gaetano Isola 2023	23	382.00	10.0000	23	417.00	10.0000	−3.44	[−4.37; −2.51]	0.9%	3.3%
Gaetano Isola 2023	23	365.00	10.0000	23	417.00	10.0000	−5.11	[−6.34; −3.88]	0.5%	3.1%
Gaetano Isola 2023	23	355.00	19.0000	23	421.00	19.0000	−3.41	[−4.34; −2.48]	0.9%	3.3%
Gaetano Isola 2023	23	358.00	14.0000	23	421.00	19.0000	−3.71	[−4.69; −2.73]	0.8%	3.3%
Gaetano Isola 2023	23	331.00	23.0000	23	421.00	19.0000	−4.19	[−5.26; −3.13]	0.7%	3.2%
Kapellas 2016	24	41.00	37.0000	24	61.00	68.0000	−0.36	[−0.93; 0.21]	2.3%	3.5%
Geisinger 2016	240	56.30	51.6000	240	49.80	58.8000	0.12	[−0.06; 0.30]	23.8%	3.6%
**Common effect model**	**973**			**973**			**−0.37**	**[−0.46; −0.28]**	**86.8%**	**.**
**Random effects model**							**−1.17**	**[−1.82; −0.52]**	**.**	**75.9%**
Heterogeneity: $I^2 = 93\%$, $\tau^2 = 2.3140$, $p < 0.01$										
**Smoke_binary:Sample without smokers**										
Steven Offenbacher 2006	25	185.40	45.0000	25	89.00	18.3000	2.76	[1.97; 3.55]	1.2%	3.4%
O fentog lu 2010	20	114.00	171.0000	20	63.00	120.0000	0.34	[−0.29; 0.96]	2.0%	3.5%
Pradeep Koppolu 2013	20	2.90	1.2000	20	4.50	1.2000	−1.31	[−2.00; −0.62]	1.6%	3.4%
Al Habashneh R 2014	20	16.00	41.0000	20	36.00	51.0000	−0.42	[−1.05; 0.20]	1.9%	3.5%
Al Habashneh R 2014	21	13.00	28.0000	21	54.00	75.0000	−0.71	[−1.34; −0.09]	2.0%	3.5%
Deepti 2017	26	39.50	3.5000	26	34.20	8.6000	0.80	[0.23; 1.36]	2.4%	3.5%
Deepti 2017	26	53.90	18.9000	26	34.20	8.6000	1.32	[0.72; 1.93]	2.1%	3.5%
**Common effect model**	**158**			**158**			**0.33**	**[0.09; 0.57]**	**13.2%**	**.**
**Random effects model**							**0.39**	**[−0.62; 1.40]**	**.**	**24.1%**
Heterogeneity: $I^2 = 93\%$, $\tau^2 = 1.7401$, $p < 0.01$										
**Common effect model**	**1131**			**1131**			**−0.28**	**[−0.37; −0.19]**	**100.0%**	**.**
**Random effects model**							**−0.79**	**[−1.39; −0.20]**	**.**	**100.0%**

Standardised Mean Difference

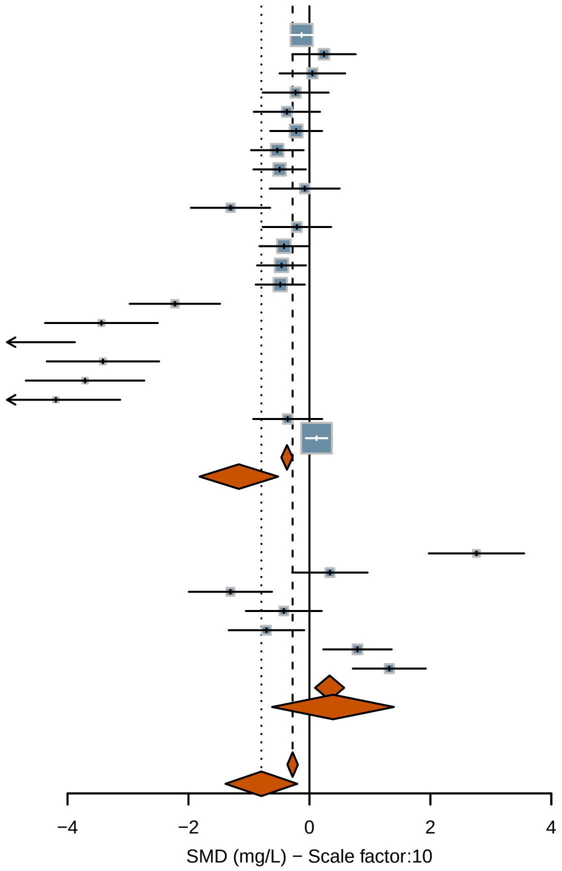

Heterogeneity: $I^2 = 93\%$, $\tau^2 = 2.5541$, $p < 0.01$
Test for subgroup differences (common effect): $\chi^2_1 = 28.52$, df = 1 ($p < 0.01$)
Test for subgroup differences (random effects): $\chi^2_1 = 6.47$, df = 1 ($p = 0.01$)

SMD: −0.28; 95%C.I.[−0.37; −0.19] P value for common effect= 0

SMD: −0.79; 95%C.I.[−1.39; −0.2] P value for random effect= 0.0088

Cytokine: hs−CRP − Treatment: Intensive

Study	Total (After TRT)	Mean (After TRT)	SD (After TRT)	Total (Before TRT)	Mean (Before TRT)	SD (Before TRT)	SMD	95%−CI	Weight (common)	Weight (random)
**Diabetes_binary:Sample with diabetic**										
Offenbacher 2009	126	31.20	3.8000	126	31.70	3.9000	−0.13	[−0.38; 0.12]	12.9%	3.8%
Panagiotis A. Koromantzos 2012	26	15.70	10.7000	26	15.20	9.7000	0.05	[−0.50; 0.59]	2.7%	3.8%
Panagiotis A. Koromantzos 2012	26	12.50	13.3000	26	15.20	9.7000	−0.23	[−0.77; 0.32]	2.6%	3.8%
Panagiotis A. Koromantzos 2012	26	10.80	13.3000	26	15.20	9.7000	−0.37	[−0.92; 0.18]	2.6%	3.8%
Eduardo Machado Vilela 2011	36	32.80	21.0000	36	40.40	24.0000	−0.33	[−0.80; 0.13]	3.6%	3.8%
Lei Chen 2012	42	23.80	28.8000	42	32.10	44.5000	−0.22	[−0.65; 0.21]	4.3%	3.8%
Lei Chen 2012	42	14.80	10.0000	42	32.10	44.5000	−0.53	[−0.97; −0.10]	4.1%	3.8%
Lei Chen 2012	42	15.80	13.1000	42	32.10	44.5000	−0.49	[−0.93; −0.06]	4.2%	3.8%
Fernando Llambes 2012	23	18.80	15.2000	23	20.00	15.2000	−0.08	[−0.66; 0.50]	2.3%	3.8%
Renukanth Patabi Cheta Raman 2014	15	70.00	134.0000	15	105.00	158.0000	−0.23	[−0.95; 0.49]	1.5%	3.7%
Yasuichi Munenaga 2013	33	9.10	8.6000	33	18.30	19.0000	−0.62	[−1.11; −0.12]	3.2%	3.8%
Yasuichi Munenaga 2013	47	3.80	3.7000	47	2.30	1.1000	0.55	[0.13; 0.96]	4.6%	3.8%
Kapellas 2016	24	41.00	37.0000	24	61.00	68.0000	−0.36	[−0.93; 0.21]	2.4%	3.8%
Geisinger 2016	240	56.30	51.6000	240	49.80	58.8000	0.12	[−0.06; 0.30]	24.5%	3.8%
**Common effect model**	**748**			**748**			**−0.10**	**[−0.20; 0.00]**	**75.6%**	**.**
**Random effects model**							**−0.18**	**[−0.35; −0.01]**	**.**	**52.6%**
Heterogeneity: $I^2 = 56\%$, $\tau^2 = 0.0557$, $p < 0.01$										
**Diabetes_binary:Sample without diabetic**										
Steven Offenbacher 2006	25	185.40	45.0000	25	89.00	18.3000	2.76	[1.97; 3.55]	1.3%	3.7%
Tuter G 2017	18	27.70	22.6000	18	49.60	39.1000	−0.67	[−1.34; 0.00]	1.7%	3.7%
Stefan Renvert	28	57.40	85.3000	28	41.90	28.0000	0.24	[−0.29; 0.77]	2.8%	3.8%
O fentog lu 2010	20	114.00	171.0000	20	63.00	120.0000	0.34	[−0.29; 0.96]	2.0%	3.7%
Eduardo Machado Vilela 2011	20	22.00	23.0000	20	30.40	38.0000	−0.26	[−0.88; 0.36]	2.0%	3.7%
Umut Altay 2013	22	34.00	7.9000	22	41.70	2.2000	−1.30	[−1.96; −0.65]	1.8%	3.7%
Umut Altay 2013	24	33.00	9.5000	24	35.00	9.5000	−0.21	[−0.77; 0.36]	2.4%	3.8%
Pradeep Koppolu 2013	20	2.90	1.2000	20	4.50	1.2000	−1.31	[−2.00; −0.62]	1.7%	3.7%
Al Habashneh R 2014	20	16.00	41.0000	20	36.00	51.0000	−0.42	[−1.05; 0.20]	2.0%	3.7%
Al Habashneh R 2014	21	13.00	28.0000	21	54.00	75.0000	−0.71	[−1.34; −0.09]	2.0%	3.7%
Deepti 2017	26	39.50	3.5000	26	34.20	8.6000	0.80	[0.23; 1.36]	2.4%	3.8%
Deepti 2017	26	53.90	18.9000	26	34.20	8.6000	1.32	[0.72; 1.93]	2.2%	3.7%
Bansari Shah 2022	30	10.14	0.1414	30	19.45	0.3540	−34.09	[−40.41; −27.77]	0.0%	2.5%
**Common effect model**	**300**			**300**			**−0.00**	**[−0.18; 0.18]**	**24.4%**	**.**
**Random effects model**							**−2.27**	**[−6.90; 2.36]**	**.**	**47.4%**
Heterogeneity: $I^2 = 95\%$, $\tau^2 = 71.7608$, $p < 0.01$										
**Common effect model**	**1048**			**1048**			**−0.08**	**[−0.16; 0.01]**	**100.0%**	**.**
**Random effects model**							**−0.93**	**[−2.63; 0.77]**	**.**	**100.0%**

Standardised Mean Difference

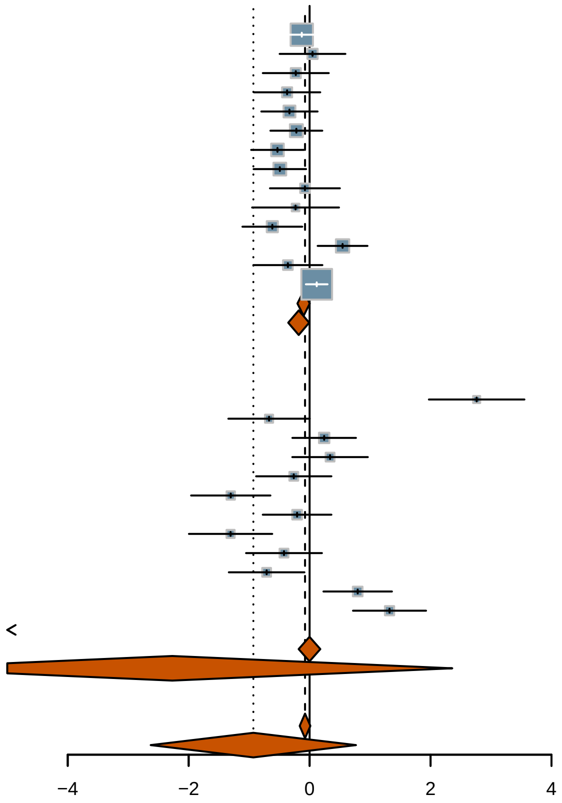

Heterogeneity: $I^2 = 90\%$, $\tau^2 = 19.9153$, $p < 0.01$
Test for subgroup differences (common effect): $\chi^2_1 = 0.85$, df = 1 ($p = 0.36$)
Test for subgroup differences (random effects): $\chi^2_1 = 0.78$, df = 1 ($p = 0.38$)

SMD: −0.08; 95%C.I.[−0.16; 0.01] P value for common effect= 0.0952

SMD: −0.93; 95%C.I.[−2.63; 0.77] P value for random effect= 0.2827

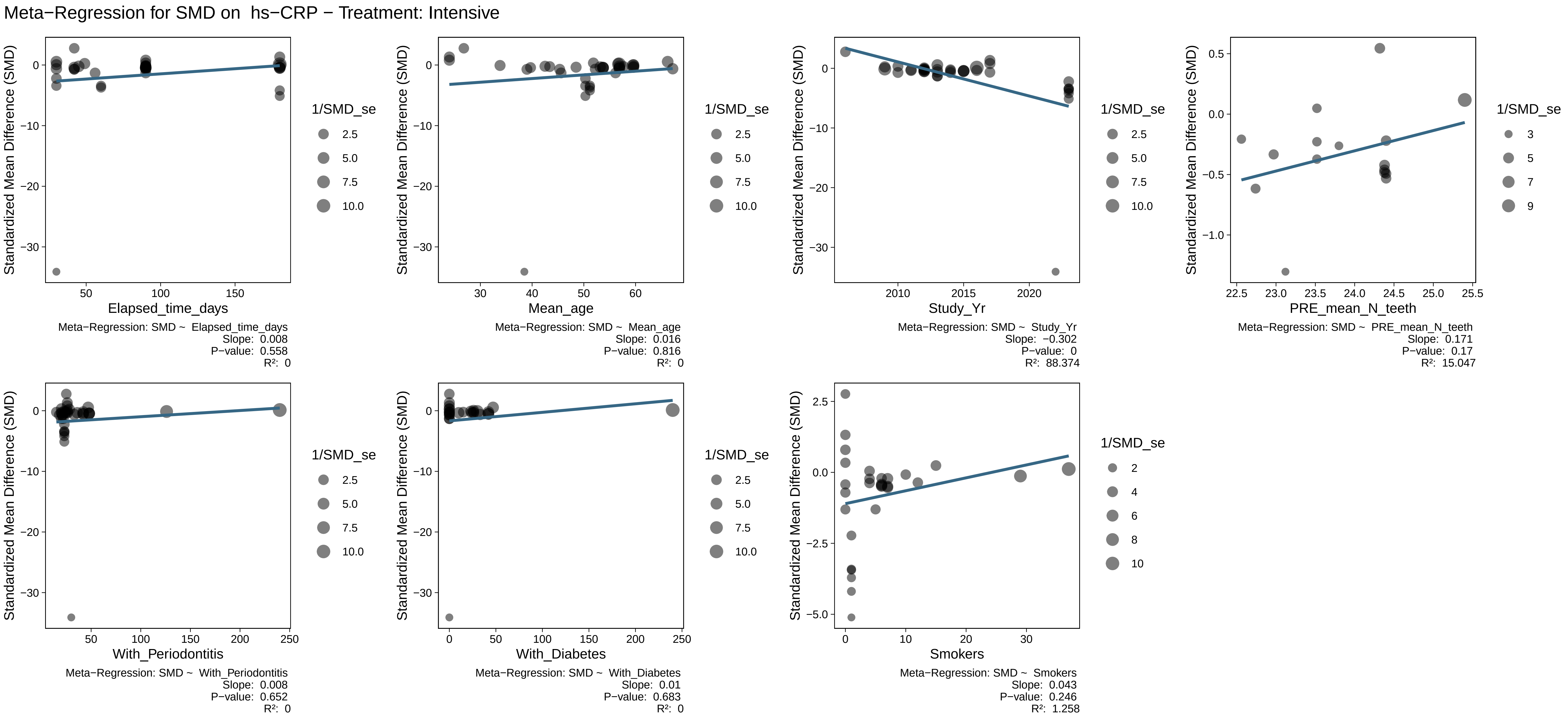
Meta−Regression for SMD on hs−CRP − Treatment: Intensive
Standardized Mean Difference (SMD)
1/SMD_se
Elapsed_time_days
Meta−Regression: SMD ~ Elapsed_time_days
Slope: 0.008
P−value: 0.558
R²: 0
Mean_age
Meta−Regression: SMD ~ Mean_age
Slope: 0.016
P−value: 0.816
R²: 0
Study_Yr
Meta−Regression: SMD ~ Study_Yr
Slope: −0.302
P−value: 0
R²: 88.374
PRE_mean_N_teeth
Meta−Regression: SMD ~ PRE_mean_N_teeth
Slope: 0.171
P−value: 0.17
R²: 15.047
With_Periodontitis
Meta−Regression: SMD ~ With_Periodontitis
Slope: 0.008
P−value: 0.652
R²: 0
With_Diabetes
Meta−Regression: SMD ~ With_Diabetes
Slope: 0.01
P−value: 0.683
R²: 0
Smokers
Meta−Regression: SMD ~ Smokers
Slope: 0.043
P−value: 0.246
R²: 1.258
