# Supplementary material for: Temporal dynamics of early inflammatory markers after professional dental cleaning: a meta-analysis and spline-based meta-regression of TNF-α, IL-1β, IL-6, and (hs)CRP
Source: Front Immunol. 2025 Aug 28;16:1634622. doi: 10.3389/fimmu.2025.1634622 (PMC12423065; doi:10.3389/fimmu.2025.1634622)

Cytokine: hs−CRP − Treatment: Standard

Study	After TRT Total	After TRT Mean	After TRT SD	Before TRT Total	Before TRT Mean	Before TRT SD	SMD	95%−CI	Weight (common)	Weight (random)
**Days after treatment:30**										
Panagiotis A. Koromantzos 2012	27	15.20	9.7000	27	13.40	12.7000	0.16	[−0.38; 0.69]	10.6%	11.2%
**Days after treatment:45**										
Lei Chen 2012	43	0.00	0.0000	43	0.00	0.0000	−0.31	[−0.74; 0.11]	16.7%	11.3%
**Days after treatment:90**										
Panagiotis A. Koromantzos 2012	27	15.40	11.7000	27	13.40	12.7000	0.16	[−0.37; 0.70]	10.6%	11.2%
Lei Chen 2012	43	19.60	19.8000	43	30.90	46.4000	−0.31	[−0.74; 0.11]	16.7%	11.3%
**Common effect model**	**70**			**70**			**−0.13**	**[−0.46; 0.20]**	**27.3%**	**.**
**Random effects model**							**−0.10**	**[−0.57; 0.36]**	**.**	**22.5%**
Heterogeneity: $I^2 = 46\%$, $\tau^2 = 0.0523$, $p = 0.17$										
**Days after treatment:180**										
Panagiotis A. Koromantzos 2012	27	14.50	9.7000	27	13.40	12.7000	0.10	[−0.44; 0.63]	10.6%	11.2%
Lei Chen 2012	43	15.30	12.7000	43	30.90	46.4000	−0.45	[−0.88; −0.03]	16.5%	11.3%
**Common effect model**	**70**			**70**			**−0.24**	**[−0.57; 0.10]**	**27.1%**	**.**
**Random effects model**							**−0.20**	**[−0.74; 0.33]**	**.**	**22.5%**
Heterogeneity: $I^2 = 60\%$, $\tau^2 = 0.0905$, $p = 0.11$										
**Days after treatment:365**										
Zekonis 2016	34	15.20	3.6000	34	24.80	4.5000	−2.33	[−2.95; −1.71]	7.8%	11.0%
**Days after treatment:730**										
Zekonis 2016	33	13.10	2.9000	33	24.80	4.5000	−3.05	[−3.78; −2.33]	5.8%	10.8%
**Days after treatment:1095**										
Zekonis 2016	30	12.40	2.6000	30	24.80	4.5000	−3.33	[−4.13; −2.53]	4.8%	10.7%
**Common effect model**	**307**			**307**			**−0.65**	**[−0.83; −0.48]**	**100.0%**	**.**
**Random effects model**							**−1.02**	**[−1.94; −0.10]**	**.**	**100.0%**

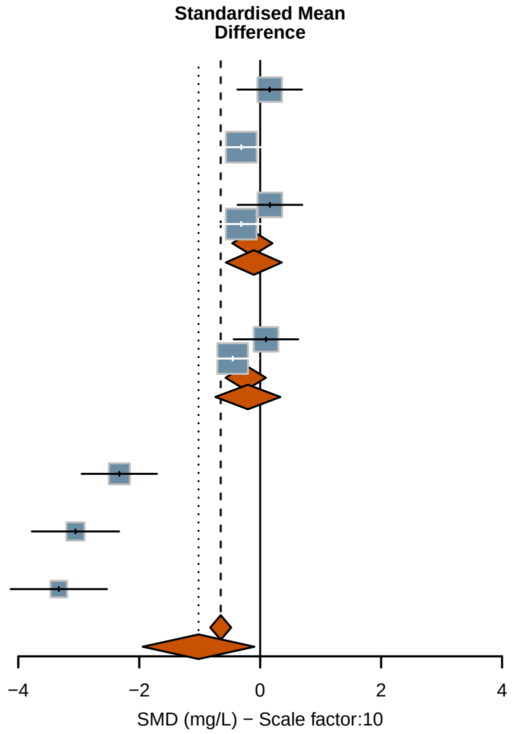

Heterogeneity: $I^2 = 94\%$, $\tau^2 = 1.9142$, $p < 0.01$
Test for subgroup differences (common effect): $\chi^2_6 = 140.43$, df = 6 ($p < 0.01$)
Test for subgroup differences (random effects): $\chi^2_6 = 130.05$, df = 6 ($p < 0.01$)

SMD: −0.65; 95%C.I.[−0.83; −0.48] P value for common effect= 0

SMD: −1.02; 95%C.I.[−1.94; −0.1] P value for random effect= 0.0305

Cytokine: hs−CRP − Treatment: Standard

Study	After TRT Total	After TRT Mean	After TRT SD	Before TRT Total	Before TRT Mean	Before TRT SD	SMD	95%−CI	Weight (common)	Weight (random)
**MonthsAfterTRT:4−6Wks**										
Panagiotis A. Koromantzos 2012	27	15.20	9.7000	27	13.40	12.7000	0.16	[−0.38; 0.69]	10.6%	11.2%
**MonthsAfterTRT:Within 3 Mo.**										
Panagiotis A. Koromantzos 2012	27	15.40	11.7000	27	13.40	12.7000	0.16	[−0.37; 0.70]	10.6%	11.2%
Lei Chen 2012	43	0.00	0.0000	43	0.00	0.0000	−0.31	[−0.74; 0.11]	16.7%	11.3%
Lei Chen 2012	43	19.60	19.8000	43	30.90	46.4000	−0.31	[−0.74; 0.11]	16.7%	11.3%
**Common effect model**	**113**			**113**			**−0.20**	**[−0.46; 0.06]**	**44.0%**	**.**
**Random effects model**							**−0.20**	**[−0.46; 0.06]**	**.**	**33.8%**
Heterogeneity: $I^2 = 13\%$, $\tau^2 = < 0.0001$, $p = 0.32$										
**MonthsAfterTRT:Within 6 Mo.**										
Panagiotis A. Koromantzos 2012	27	14.50	9.7000	27	13.40	12.7000	0.10	[−0.44; 0.63]	10.6%	11.2%
Lei Chen 2012	43	15.30	12.7000	43	30.90	46.4000	−0.45	[−0.88; −0.03]	16.5%	11.3%
**Common effect model**	**70**			**70**			**−0.24**	**[−0.57; 0.10]**	**27.1%**	**.**
**Random effects model**							**−0.20**	**[−0.74; 0.33]**	**.**	**22.5%**
Heterogeneity: $I^2 = 60\%$, $\tau^2 = 0.0905$, $p = 0.11$										
**MonthsAfterTRT:6Mo. and more**										
Zekonis 2016	34	15.20	3.6000	34	24.80	4.5000	−2.33	[−2.95; −1.71]	7.8%	11.0%
Zekonis 2016	33	13.10	2.9000	33	24.80	4.5000	−3.05	[−3.78; −2.33]	5.8%	10.8%
Zekonis 2016	30	12.40	2.6000	30	24.80	4.5000	−3.33	[−4.13; −2.53]	4.8%	10.7%
**Common effect model**	**97**			**97**			**−2.82**	**[−3.22; −2.41]**	**18.4%**	**.**
**Random effects model**							**−2.87**	**[−3.47; −2.26]**	**.**	**32.5%**
Heterogeneity: $I^2 = 54\%$, $\tau^2 = 0.1539$, $p = 0.11$										
**Common effect model**	**307**			**307**			**−0.65**	**[−0.83; −0.48]**	**100.0%**	**.**
**Random effects model**							**−1.02**	**[−1.94; −0.10]**	**.**	**100.0%**

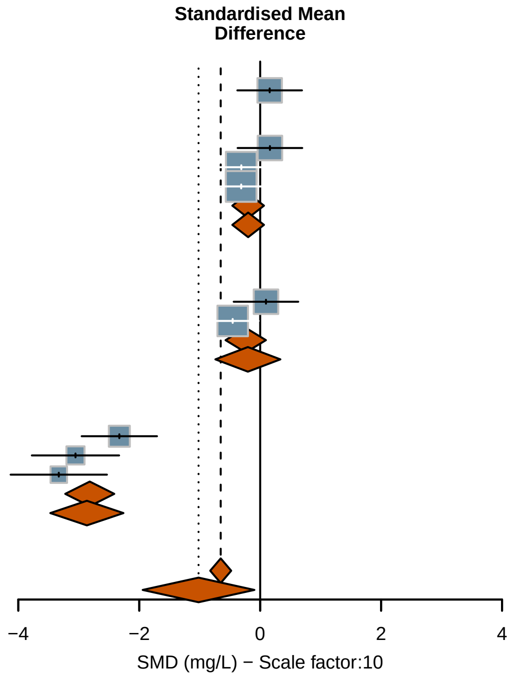

Heterogeneity: $I^2 = 94\%$, $\tau^2 = 1.9142$, $p < 0.01$
Test for subgroup differences (common effect): $\chi^2_3 = 135.62$, df = 3 ($p < 0.01$)
Test for subgroup differences (random effects): $\chi^2_3 = 70.64$, df = 3 ($p < 0.01$)

SMD: −0.65; 95%C.I.[−0.83; −0.48] P value for common effect= 0

SMD: −1.02; 95%C.I.[−1.94; −0.1] P value for random effect= 0.0305

Cytokine: hs−CRP − Treatment: Standard

Study	After TRT Total	After TRT Mean	After TRT SD	Before TRT Total	Before TRT Mean	Before TRT SD	SMD	95%−CI	Weight (common)	Weight (random)
**Assay type:ELISA**										
Lei Chen 2012	43	0.00	0.0000	43	0.00	0.0000	−0.31	[−0.74; 0.11]	16.7%	11.3%
Lei Chen 2012	43	19.60	19.8000	43	30.90	46.4000	−0.31	[−0.74; 0.11]	16.7%	11.3%
Lei Chen 2012	43	15.30	12.7000	43	30.90	46.4000	−0.45	[−0.88; −0.03]	16.5%	11.3%
**Common effect model**	**129**			**129**			**−0.36**	**[−0.61; −0.11]**	**49.9%**	**.**
**Random effects model**							**−0.36**	**[−0.61; −0.11]**	**.**	**34.0%**
Heterogeneity: $I^2 = 0\%$, $\tau^2 = 0$, $p = 0.87$										
**Assay type:particle−enhanced turbidimetric assay**										
Zekonis 2016	34	15.20	3.6000	34	24.80	4.5000	−2.33	[−2.95; −1.71]	7.8%	11.0%
Zekonis 2016	33	13.10	2.9000	33	24.80	4.5000	−3.05	[−3.78; −2.33]	5.8%	10.8%
Zekonis 2016	30	12.40	2.6000	30	24.80	4.5000	−3.33	[−4.13; −2.53]	4.8%	10.7%
**Common effect model**	**97**			**97**			**−2.82**	**[−3.22; −2.41]**	**18.4%**	**.**
**Random effects model**							**−2.87**	**[−3.47; −2.26]**	**.**	**32.5%**
Heterogeneity: $I^2 = 54\%$, $\tau^2 = 0.1539$, $p = 0.11$										
**Assay type:PCR**										
Panagiotis A. Koromantzos 2012	27	15.20	9.7000	27	13.40	12.7000	0.16	[−0.38; 0.69]	10.6%	11.2%
Panagiotis A. Koromantzos 2012	27	15.40	11.7000	27	13.40	12.7000	0.16	[−0.37; 0.70]	10.6%	11.2%
Panagiotis A. Koromantzos 2012	27	14.50	9.7000	27	13.40	12.7000	0.10	[−0.44; 0.63]	10.6%	11.2%
**Common effect model**	**81**			**81**			**0.14**	**[−0.17; 0.45]**	**31.8%**	**.**
**Random effects model**							**0.14**	**[−0.17; 0.45]**	**.**	**33.5%**
Heterogeneity: $I^2 = 0\%$, $\tau^2 = 0$, $p = 0.98$										
**Common effect model**	**307**			**307**			**−0.65**	**[−0.83; −0.48]**	**100.0%**	**.**
**Random effects model**							**−1.02**	**[−1.94; −0.10]**	**.**	**100.0%**

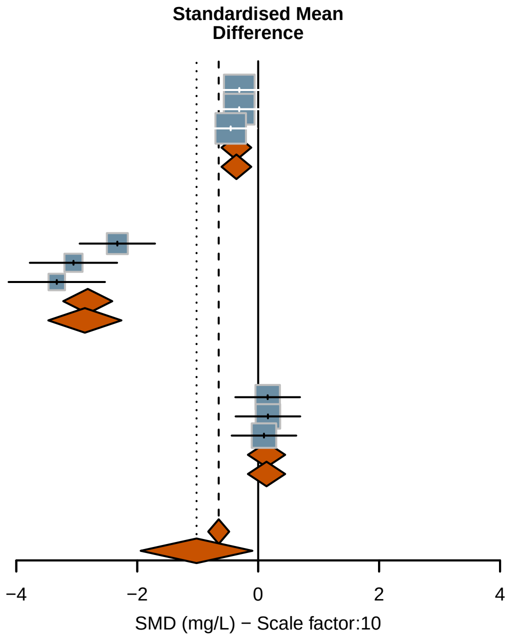

Heterogeneity: $I^2 = 94\%$, $\tau^2 = 1.9142$, $p < 0.01$
Test for subgroup differences (common effect): $\chi^2_2 = 140.09$, df = 2 ($p < 0.01$)
Test for subgroup differences (random effects): $\chi^2_2 = 75.69$, df = 2 ($p < 0.01$)

SMD: −0.65; 95%C.I.[−0.83; −0.48] P value for common effect= 0

SMD: −1.02; 95%C.I.[−1.94; −0.1] P value for random effect= 0.0305

Cytokine: hs−CRP − Treatment: Standard

Study	After TRT Total	After TRT Mean	After TRT SD	Before TRT Total	Before TRT Mean	Before TRT SD	SMD	95%−CI	Weight (common)	Weight (random)
**Bias judgment:Low risk**										
Panagiotis A. Koromantzos 2012	27	15.20	9.7000	27	13.40	12.7000	0.16	[−0.38; 0.69]	10.6%	11.2%
Panagiotis A. Koromantzos 2012	27	15.40	11.7000	27	13.40	12.7000	0.16	[−0.37; 0.70]	10.6%	11.2%
Panagiotis A. Koromantzos 2012	27	14.50	9.7000	27	13.40	12.7000	0.10	[−0.44; 0.63]	10.6%	11.2%
Zekonis 2016	34	15.20	3.6000	34	24.80	4.5000	−2.33	[−2.95; −1.71]	7.8%	11.0%
Zekonis 2016	33	13.10	2.9000	33	24.80	4.5000	−3.05	[−3.78; −2.33]	5.8%	10.8%
Zekonis 2016	30	12.40	2.6000	30	24.80	4.5000	−3.33	[−4.13; −2.53]	4.8%	10.7%
**Common effect model**	**178**			**178**			**−0.94**	**[−1.19; −0.70]**	**50.1%**	**.**
**Random effects model**							**−1.37**	**[−2.72; −0.01]**	**.**	**66.0%**
Heterogeneity: $I^2 = 96\%$, $\tau^2 = 2.7657$, $p < 0.01$										
**Bias judgment:Some concerns**										
Lei Chen 2012	43	0.00	0.0000	43	0.00	0.0000	−0.31	[−0.74; 0.11]	16.7%	11.3%
Lei Chen 2012	43	19.60	19.8000	43	30.90	46.4000	−0.31	[−0.74; 0.11]	16.7%	11.3%
Lei Chen 2012	43	15.30	12.7000	43	30.90	46.4000	−0.45	[−0.88; −0.03]	16.5%	11.3%
**Common effect model**	**129**			**129**			**−0.36**	**[−0.61; −0.11]**	**49.9%**	**.**
**Random effects model**							**−0.36**	**[−0.61; −0.11]**	**.**	**34.0%**
Heterogeneity: $I^2 = 0\%$, $\tau^2 = 0$, $p = 0.87$										
**Common effect model**	**307**			**307**			**−0.65**	**[−0.83; −0.48]**	**100.0%**	**.**
**Random effects model**							**−1.02**	**[−1.94; −0.10]**	**.**	**100.0%**

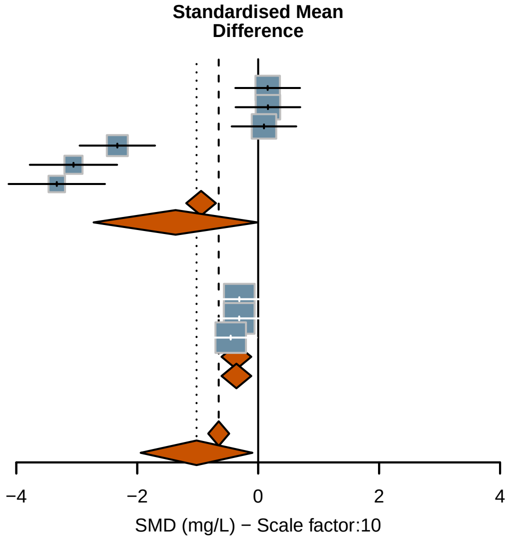

Heterogeneity: $I^2 = 94\%$, $\tau^2 = 1.9142$, $p < 0.01$
Test for subgroup differences (common effect): $\chi^2_1 = 10.86$, df = 1 ($p < 0.01$)
Test for subgroup differences (random effects): $\chi^2_1 = 2.05$, df = 1 ($p = 0.15$)

SMD: −0.65; 95%C.I.[−0.83; −0.48] P value for common effect= 0

SMD: −1.02; 95%C.I.[−1.94; −0.1] P value for random effect= 0.0305

Cytokine: hs−CRP − Treatment: Standard

Study	After TRT Total	After TRT Mean	After TRT SD	Before TRT Total	Before TRT Mean	Before TRT SD	SMD	95%−CI	Weight (common)	Weight (random)
**Study design:RCT**										
Panagiotis A. Koromantzos 2012	27	15.20	9.7000	27	13.40	12.7000	0.16	[−0.38; 0.69]	10.6%	11.2%
Panagiotis A. Koromantzos 2012	27	15.40	11.7000	27	13.40	12.7000	0.16	[−0.37; 0.70]	10.6%	11.2%
Panagiotis A. Koromantzos 2012	27	14.50	9.7000	27	13.40	12.7000	0.10	[−0.44; 0.63]	10.6%	11.2%
Lei Chen 2012	43	0.00	0.0000	43	0.00	0.0000	−0.31	[−0.74; 0.11]	16.7%	11.3%
Lei Chen 2012	43	19.60	19.8000	43	30.90	46.4000	−0.31	[−0.74; 0.11]	16.7%	11.3%
Lei Chen 2012	43	15.30	12.7000	43	30.90	46.4000	−0.45	[−0.88; −0.03]	16.5%	11.3%
Zekonis 2016	34	15.20	3.6000	34	24.80	4.5000	−2.33	[−2.95; −1.71]	7.8%	11.0%
Zekonis 2016	33	13.10	2.9000	33	24.80	4.5000	−3.05	[−3.78; −2.33]	5.8%	10.8%
Zekonis 2016	30	12.40	2.6000	30	24.80	4.5000	−3.33	[−4.13; −2.53]	4.8%	10.7%
**Common effect model**	**307**			**307**			**−0.65**	**[−0.83; −0.48]**	**100.0%**	**.**
**Random effects model**							**−1.02**	**[−1.94; −0.10]**	**.**	**100.0%**
Heterogeneity: $I^2 = 94\%$, $\tau^2 = 1.9142$, $p < 0.01$										
**Common effect model**	**307**			**307**			**−0.65**	**[−0.83; −0.48]**	**100.0%**	**.**
**Random effects model**							**−1.02**	**[−1.94; −0.10]**	**.**	**100.0%**

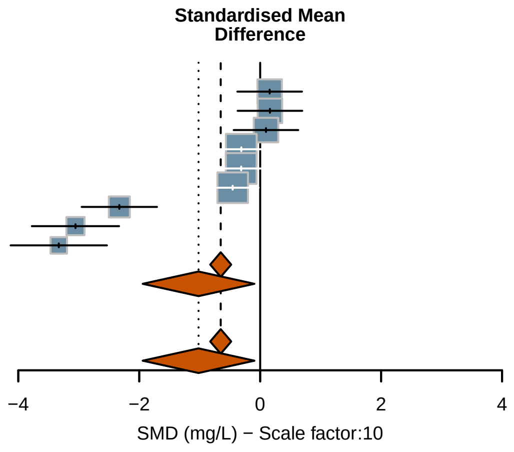

Heterogeneity: $I^2 = 94\%$, $\tau^2 = 1.9142$, $p < 0.01$
Test for subgroup differences (common effect): $\chi^2_0 = 0.00$, df = 0 (p = NA)
Test for subgroup differences (random effects): $\chi^2_0 = 0.00$, df = 0 (p = NA)

SMD: −0.65; 95%C.I.[−0.83; −0.48] P value for common effect= 0

SMD: −1.02; 95%C.I.[−1.94; −0.1] P value for random effect= 0.0305

Cytokine: hs−CRP − Treatment: Standard

Study	After TRT Total	After TRT Mean	After TRT SD	Before TRT Total	Before TRT Mean	Before TRT SD	SMD	95%−CI	Weight (common)	Weight (random)
**Additional_devices:weekely H2O2 0,5% irrigation**										
Zekonis 2016	34	15.20	3.6000	34	24.80	4.5000	−2.33	[−2.95; −1.71]	42.4%	37.3%
Zekonis 2016	33	13.10	2.9000	33	24.80	4.5000	−3.05	[−3.78; −2.33]	31.6%	32.9%
Zekonis 2016	30	12.40	2.6000	30	24.80	4.5000	−3.33	[−4.13; −2.53]	25.9%	29.8%
**Common effect model**	**97**			**97**			**−2.82**	**[−3.22; −2.41]**	**100.0%**	**.**
**Random effects model**							**−2.87**	**[−3.47; −2.26]**	**.**	**100.0%**
Heterogeneity: I^2 = 54%, τ^2 = 0.1539, p = 0.11										
**Common effect model**	**97**			**97**			**−2.82**	**[−3.22; −2.41]**	**100.0%**	**.**
**Random effects model**							**−2.87**	**[−3.47; −2.26]**	**.**	**100.0%**

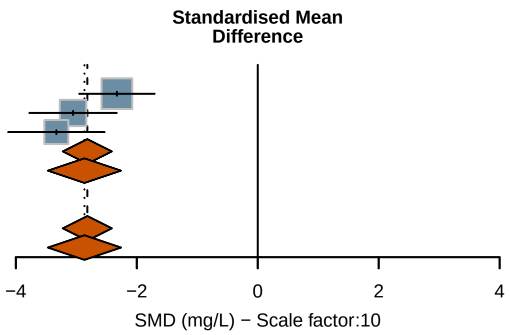

Heterogeneity: I^2 = 54%, τ^2 = 0.1539, p = 0.11
Test for subgroup differences (common effect): χ^2_0 = 0.00, df = 0 (p = NA)
Test for subgroup differences (random effects): χ^2_0 = 0.00, df = 0 (p = NA)

SMD: −2.82; 95%C.I.[−3.22; −2.41] P value for common effect= 0

SMD: −2.87; 95%C.I.[−3.47; −2.26] P value for random effect= 0

Cytokine: hs−CRP − Treatment: Standard

Study	After TRT Total	After TRT Mean	After TRT SD	Before TRT Total	Before TRT Mean	Before TRT SD	SMD	95%−CI	Weight (common)	Weight (random)
**Female_binary:Sex mixed**										
Panagiotis A. Koromantzos 2012	27	15.20	9.7000	27	13.40	12.7000	0.16	[−0.38; 0.69]	10.6%	11.2%
Panagiotis A. Koromantzos 2012	27	15.40	11.7000	27	13.40	12.7000	0.16	[−0.37; 0.70]	10.6%	11.2%
Panagiotis A. Koromantzos 2012	27	14.50	9.7000	27	13.40	12.7000	0.10	[−0.44; 0.63]	10.6%	11.2%
Lei Chen 2012	43	0.00	0.0000	43	0.00	0.0000	−0.31	[−0.74; 0.11]	16.7%	11.3%
Lei Chen 2012	43	19.60	19.8000	43	30.90	46.4000	−0.31	[−0.74; 0.11]	16.7%	11.3%
Lei Chen 2012	43	15.30	12.7000	43	30.90	46.4000	−0.45	[−0.88; −0.03]	16.5%	11.3%
Zekonis 2016	34	15.20	3.6000	34	24.80	4.5000	−2.33	[−2.95; −1.71]	7.8%	11.0%
Zekonis 2016	33	13.10	2.9000	33	24.80	4.5000	−3.05	[−3.78; −2.33]	5.8%	10.8%
Zekonis 2016	30	12.40	2.6000	30	24.80	4.5000	−3.33	[−4.13; −2.53]	4.8%	10.7%
**Common effect model**	**307**			**307**			**−0.65**	**[−0.83; −0.48]**	**100.0%**	**.**
**Random effects model**							**−1.02**	**[−1.94; −0.10]**	**.**	**100.0%**
Heterogeneity: $I^2 = 94\%$, $\tau^2 = 1.9142$, $p < 0.01$										
**Common effect model**	**307**			**307**			**−0.65**	**[−0.83; −0.48]**	**100.0%**	**.**
**Random effects model**							**−1.02**	**[−1.94; −0.10]**	**.**	**100.0%**

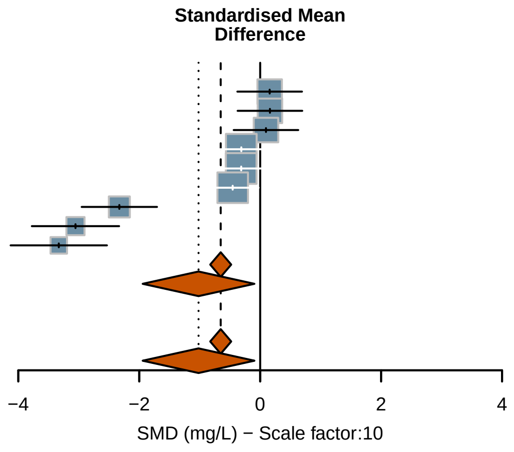

Heterogeneity: $I^2 = 94\%$, $\tau^2 = 1.9142$, $p < 0.01$
Test for subgroup differences (common effect): $\chi^2_0 = 0.00$, df = 0 (p = NA)
Test for subgroup differences (random effects): $\chi^2_0 = 0.00$, df = 0 (p = NA)

SMD: −0.65; 95%C.I.[−0.83; −0.48] P value for common effect= 0

SMD: −1.02; 95%C.I.[−1.94; −0.1] P value for random effect= 0.0305

Cytokine: hs−CRP − Treatment: Standard

Study	After TRT Total	After TRT Mean	After TRT SD	Before TRT Total	Before TRT Mean	Before TRT SD	SMD	95%−CI	Weight (common)	Weight (random)
**Smoke_binary:Sample with smokers**										
Panagiotis A. Koromantzos 2012	27	15.20	9.7000	27	13.40	12.7000	0.16	[−0.38; 0.69]	10.6%	11.2%
Panagiotis A. Koromantzos 2012	27	15.40	11.7000	27	13.40	12.7000	0.16	[−0.37; 0.70]	10.6%	11.2%
Panagiotis A. Koromantzos 2012	27	14.50	9.7000	27	13.40	12.7000	0.10	[−0.44; 0.63]	10.6%	11.2%
Lei Chen 2012	43	0.00	0.0000	43	0.00	0.0000	−0.31	[−0.74; 0.11]	16.7%	11.3%
Lei Chen 2012	43	19.60	19.8000	43	30.90	46.4000	−0.31	[−0.74; 0.11]	16.7%	11.3%
Lei Chen 2012	43	15.30	12.7000	43	30.90	46.4000	−0.45	[−0.88; −0.03]	16.5%	11.3%
**Common effect model**	**210**			**210**			**−0.17**	**[−0.36; 0.03]**	**81.6%**	**.**
**Random effects model**							**−0.15**	**[−0.37; 0.06]**	**.**	**67.5%**
Heterogeneity: $I^2 = 22\%$, $\tau^2 = 0.0158$, $p = 0.27$										
**Smoke_binary:Sample without smokers**										
Zekonis 2016	34	15.20	3.6000	34	24.80	4.5000	−2.33	[−2.95; −1.71]	7.8%	11.0%
Zekonis 2016	33	13.10	2.9000	33	24.80	4.5000	−3.05	[−3.78; −2.33]	5.8%	10.8%
Zekonis 2016	30	12.40	2.6000	30	24.80	4.5000	−3.33	[−4.13; −2.53]	4.8%	10.7%
**Common effect model**	**97**			**97**			**−2.82**	**[−3.22; −2.41]**	**18.4%**	**.**
**Random effects model**							**−2.87**	**[−3.47; −2.26]**	**.**	**32.5%**
Heterogeneity: $I^2 = 54\%$, $\tau^2 = 0.1539$, $p = 0.11$										
**Common effect model**	**307**			**307**			**−0.65**	**[−0.83; −0.48]**	**100.0%**	**.**
**Random effects model**							**−1.02**	**[−1.94; −0.10]**	**.**	**100.0%**

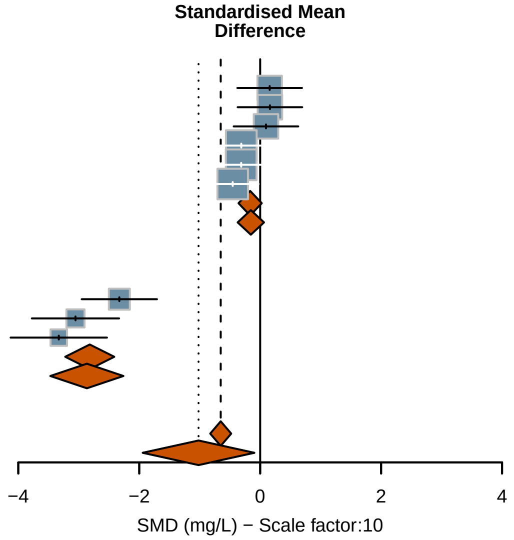

Heterogeneity: $I^2 = 94\%$, $\tau^2 = 1.9142$, $p < 0.01$
Test for subgroup differences (common effect): $\chi^2_1 = 133.98$, df = 1 ($p < 0.01$)
Test for subgroup differences (random effects): $\chi^2_1 = 68.37$, df = 1 ($p < 0.01$)

SMD: −0.65; 95%C.I.[−0.83; −0.48] P value for common effect= 0

SMD: −1.02; 95%C.I.[−1.94; −0.1] P value for random effect= 0.0305

Cytokine: hs−CRP − Treatment: Standard

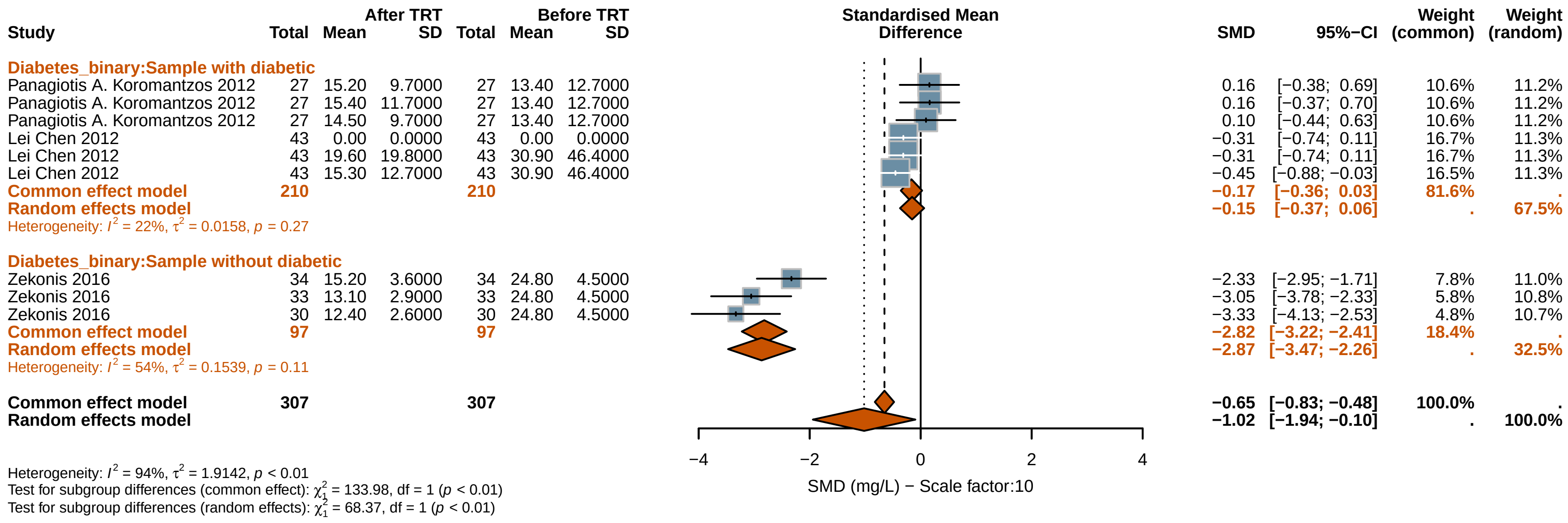

SMD: −0.65; 95%C.I.[−0.83; −0.48] P value for common effect= 0

SMD: −1.02; 95%C.I.[−1.94; −0.1] P value for random effect= 0.0305

Meta−Regression for SMD on hs−CRP − Treatment: Standard

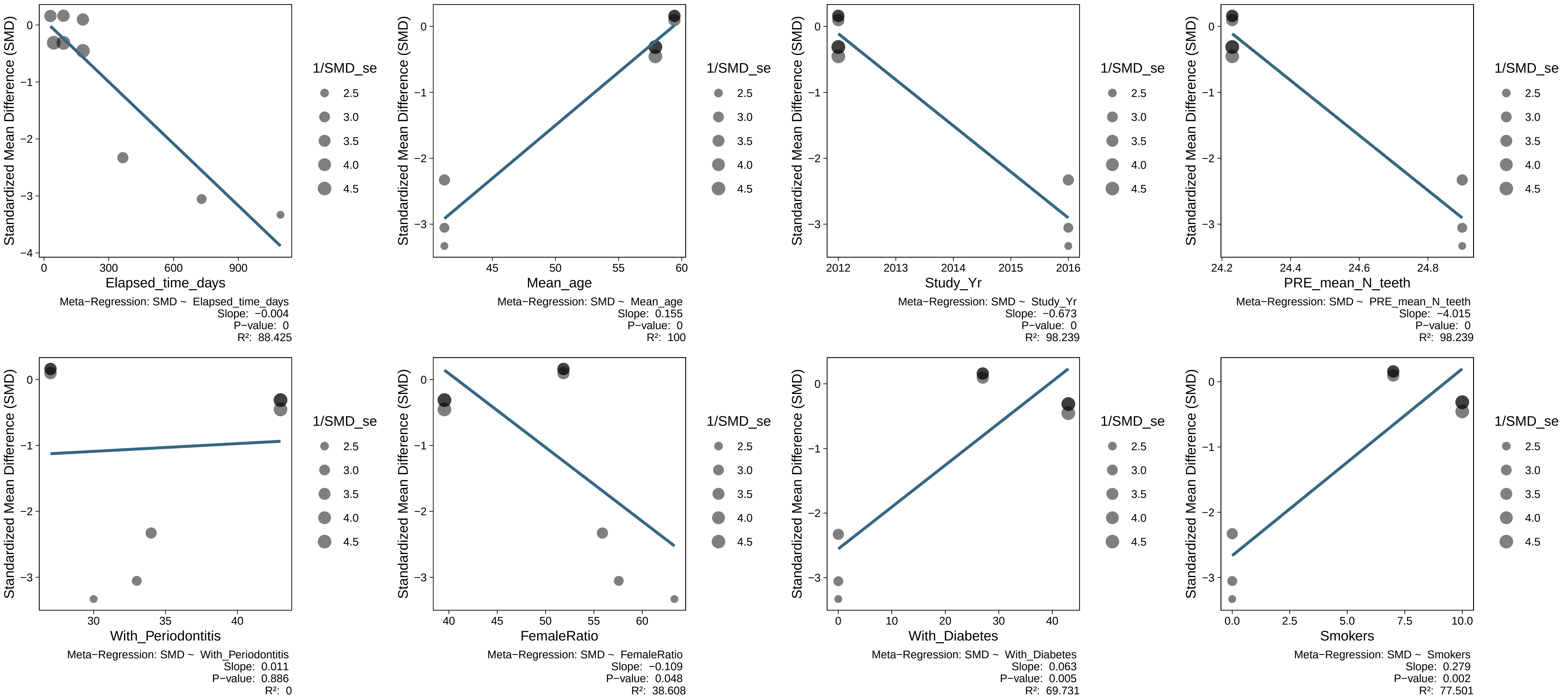

Supplement: Supplementary file 1 [file DataSheet1.zip › Supplementary materials/PDF/hs-CRP_Standard_results.pdf]
