# Supplementary material for: Temporal dynamics of early inflammatory markers after professional dental cleaning: a meta-analysis and spline-based meta-regression of TNF-α, IL-1β, IL-6, and (hs)CRP
Source: Front Immunol. 2025 Aug 28;16:1634622. doi: 10.3389/fimmu.2025.1634622 (PMC12423065; doi:10.3389/fimmu.2025.1634622)
Supplement: Supplementary file 1 [file DataSheet1.zip › Supplementary materials/PDF/IL-1beta_Intensive_results.pdf]

Cytokine: IL−1beta − Treatment: Intensive

Study	After TRT Total	After TRT Mean	After TRT SD	Before TRT Total	Before TRT Mean	Before TRT SD	SMD	95%−CI	Weight (common)	Weight (random)
**Days after treatment:21**										
Fiorini T 2012	27	1.70	0.6300	27	1.70	0.4700	0.00	[−0.53; 0.53]	6.1%	4.1%
**Days after treatment:30**										
de Sousa Rabelo 2020	15	0.17	0.4090	15	0.15	0.1940	0.08	[−0.64; 0.80]	3.4%	4.1%
de Sousa Rabelo 2020	15	0.13	0.2780	15	0.07	0.1060	0.28	[−0.44; 1.00]	3.4%	4.1%
de Sousa Rabelo 2020	15	0.20	0.3930	15	0.09	0.0900	0.39	[−0.34; 1.11]	3.3%	4.1%
Anwar Mohammad 2020	30	3.45	0.4860	30	5.04	0.6090	−2.84	[−3.57; −2.11]	3.3%	4.1%
Anwar Mohammad 2020	30	4.53	0.5290	30	10.56	0.6060	−10.46	[−12.46; −8.46]	0.4%	4.0%
**Common effect model**	**105**			**105**			**−0.82**	**[−1.18; −0.47]**	**13.9%**	**.**
**Random effects model**							**−2.44**	**[−6.42; 1.53]**	**.**	**20.3%**
Heterogeneity: $I^2 = 97\%$, $\tau^2 = 20.2480$, $p < 0.01$										
**Days after treatment:35**										
Escobar Arregoces 2021	19	0.30	0.0700	19	0.53	0.1400	−2.03	[−2.83; −1.24]	2.7%	4.1%
**Days after treatment:42**										
Nikolaos Markou 2023	20	19.58	28.3000	20	12.43	17.7000	0.30	[−0.33; 0.92]	4.5%	4.1%
Nikolaos Markou 2023	20	30.60	57.9000	20	32.70	63.3330	−0.03	[−0.65; 0.59]	4.6%	4.1%
Nikolaos Markou 2023	20	9.39	9.9000	20	10.60	13.0000	−0.10	[−0.72; 0.52]	4.5%	4.1%
**Common effect model**	**60**			**60**			**0.05**	**[−0.31; 0.41]**	**13.6%**	**.**
**Random effects model**							**0.05**	**[−0.31; 0.41]**	**.**	**12.2%**
Heterogeneity: $I^2 = 0\%$, $\tau^2 = 0$, $p = 0.64$										
**Days after treatment:60**										
Yong−Wei Fu 2015	54	1.49	0.5900	54	2.23	0.5800	−1.26	[−1.67; −0.84]	10.2%	4.1%
**Days after treatment:90**										
Nikolaos Markou 2023	20	16.90	27.0000	20	12.43	17.7000	0.19	[−0.43; 0.81]	4.5%	4.1%
Nikolaos Markou 2023	20	28.80	47.9000	20	32.70	63.3330	−0.07	[−0.69; 0.55]	4.6%	4.1%
Nikolaos Markou 2023	20	8.79	13.3330	20	10.60	13.0000	−0.13	[−0.76; 0.49]	4.5%	4.1%
Javed 2016	22	32.40	0.4000	22	67.50	2.6000	−18.53	[−22.62; −14.44]	0.1%	3.9%
Javed 2016	22	4.50	0.4000	22	70.70	1.2000	−72.68	[−88.57; −56.80]	0.0%	2.6%
Javed 2016	22	13.50	9.6000	22	30.50	3.1000	−2.34	[−3.12; −1.56]	2.9%	4.1%
Javed 2016	21	3.70	0.5000	21	27.40	1.6000	−19.62	[−24.05; −15.18]	0.1%	3.9%
**Common effect model**	**147**			**147**			**−0.66**	**[−0.98; −0.33]**	**16.7%**	**.**
**Random effects model**							**−15.36**	**[−33.76; 3.03]**	**.**	**26.7%**
Heterogeneity: $I^2 = 98\%$, $\tau^2 = 606.5642$, $p < 0.01$										
**Days after treatment:180**										
Nikolaos Markou 2023	20	17.70	26.4600	20	12.43	17.7000	0.23	[−0.39; 0.85]	4.5%	4.1%
Nikolaos Markou 2023	20	16.67	27.3000	20	32.70	63.3330	−0.32	[−0.95; 0.30]	4.5%	4.1%
Nikolaos Markou 2023	20	10.91	12.3910	20	10.60	13.0000	0.02	[−0.60; 0.64]	4.6%	4.1%
Yong−Wei Fu 2015	54	1.47	0.4200	54	2.23	0.5800	−1.49	[−1.92; −1.06]	9.6%	4.1%
**Common effect model**	**114**			**114**			**−0.63**	**[−0.90; −0.35]**	**23.1%**	**.**
**Random effects model**							**−0.41**	**[−1.19; 0.37]**	**.**	**16.3%**
Heterogeneity: $I^2 = 89\%$, $\tau^2 = 0.5480$, $p < 0.01$										
**Days after treatment:360**										
Nikolaos Markou 2023	20	18.25	27.2500	20	12.43	17.7000	0.25	[−0.37; 0.87]	4.5%	4.1%
Nikolaos Markou 2023	20	31.50	51.5150	20	32.70	63.3330	−0.02	[−0.64; 0.60]	4.6%	4.1%
Nikolaos Markou 2023	20	13.00	24.8000	20	10.60	13.0000	0.12	[−0.50; 0.74]	4.5%	4.1%
**Common effect model**	**60**			**60**			**0.12**	**[−0.24; 0.47]**	**13.6%**	**.**
**Random effects model**							**0.12**	**[−0.24; 0.47]**	**.**	**12.2%**
Heterogeneity: $I^2 = 0\%$, $\tau^2 = 0$, $p = 0.84$										
**Common effect model**	**586**			**586**			**−0.53**	**[−0.66; −0.40]**	**100.0%**	**.**
**Random effects model**							**−4.14**	**[−8.31; 0.04]**	**.**	**100.0%**

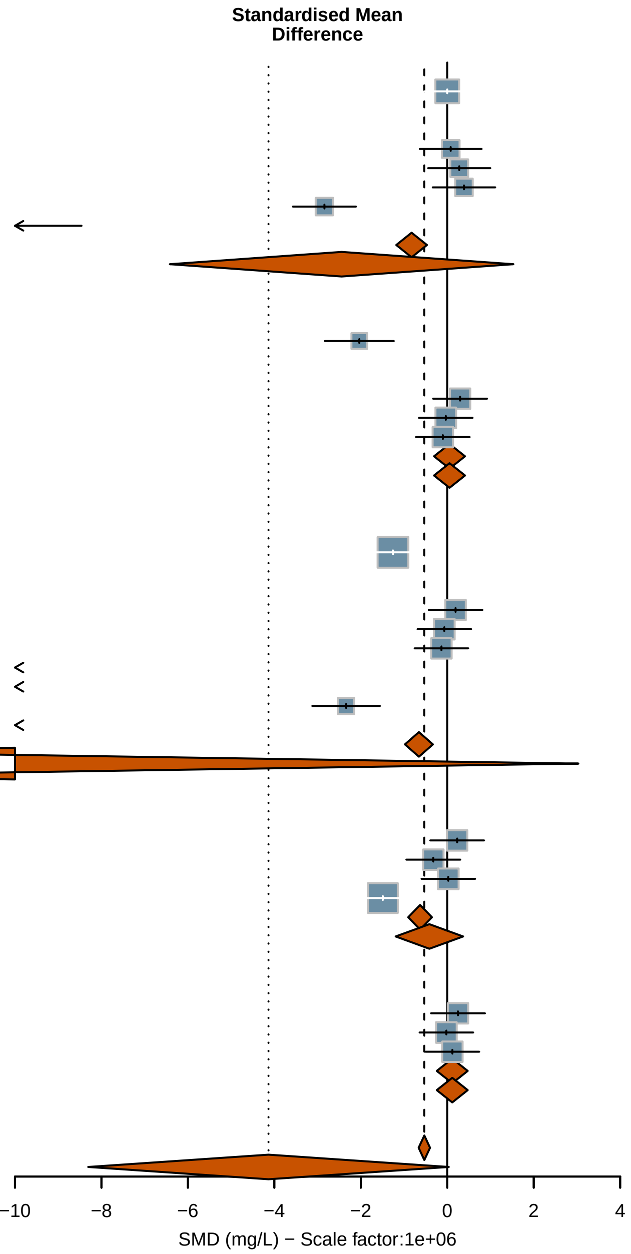

Heterogeneity: $I^2 = 95\%$, $\tau^2 = 111.0134$, $p < 0.01$
Test for subgroup differences (common effect): $\chi^2_7 = 55.56$, df = 7 ($p < 0.01$)
Test for subgroup differences (random effects): $\chi^2_7 = 52.13$, df = 7 ($p < 0.01$)

SMD: −0.53; 95%C.I.[−0.66; −0.4] P value for common effect= 0

SMD: −4.14; 95%C.I.[−8.31; 0.04] P value for random effect= 0.052

Cytokine: IL−1beta − Treatment: Intensive

Study	After TRT Total	After TRT Mean	After TRT SD	Before TRT Total	Before TRT Mean	Before TRT SD	SMD	95%−CI	Weight (common)	Weight (random)
**MonthsAfterTRT:4−6Wks**										
Fiorini T 2012	27	1.70	0.6300	27	1.70	0.4700	0.00	[−0.53; 0.53]	6.1%	4.1%
Nikolaos Markou 2023	20	19.58	28.3000	20	12.43	17.7000	0.30	[−0.33; 0.92]	4.5%	4.1%
Nikolaos Markou 2023	20	30.60	57.9000	20	32.70	63.3330	−0.03	[−0.65; 0.59]	4.6%	4.1%
Nikolaos Markou 2023	20	9.39	9.9000	20	10.60	13.0000	−0.10	[−0.72; 0.52]	4.5%	4.1%
Escobar Arregoces 2021	19	0.30	0.0700	19	0.53	0.1400	−2.03	[−2.83; −1.24]	2.7%	4.1%
de Sousa Rabelo 2020	15	0.17	0.4090	15	0.15	0.1940	0.08	[−0.64; 0.80]	3.4%	4.1%
de Sousa Rabelo 2020	15	0.13	0.2780	15	0.07	0.1060	0.28	[−0.44; 1.00]	3.4%	4.1%
de Sousa Rabelo 2020	15	0.20	0.3930	15	0.09	0.0900	0.39	[−0.34; 1.11]	3.3%	4.1%
Anwar Mohammad 2020	30	3.45	0.4860	30	5.04	0.6090	−2.84	[−3.57; −2.11]	3.3%	4.1%
Anwar Mohammad 2020	30	4.53	0.5290	30	10.56	0.6060	−10.46	[−12.46; −8.46]	0.4%	4.0%
**Common effect model**	**211**			**211**			**−0.45**	**[−0.67; −0.23]**	**36.4%**	**.**
**Random effects model**							**−1.36**	**[−3.31; 0.59]**	**.**	**40.7%**
Heterogeneity: I^2 = 95%, τ^2 = 9.7002, p < 0.01										
**MonthsAfterTRT:Within 3 Mo.**										
Nikolaos Markou 2023	20	16.90	27.0000	20	12.43	17.7000	0.19	[−0.43; 0.81]	4.5%	4.1%
Nikolaos Markou 2023	20	28.80	47.9000	20	32.70	63.3330	−0.07	[−0.69; 0.55]	4.6%	4.1%
Nikolaos Markou 2023	20	8.79	13.3330	20	10.60	13.0000	−0.13	[−0.76; 0.49]	4.5%	4.1%
Javed 2016	22	32.40	0.4000	22	67.50	2.6000	−18.53	[−22.62; −14.44]	0.1%	3.9%
Javed 2016	22	4.50	0.4000	22	70.70	1.2000	−72.68	[−88.57; −56.80]	0.0%	2.6%
Javed 2016	22	13.50	9.6000	22	30.50	3.1000	−2.34	[−3.12; −1.56]	2.9%	4.1%
Javed 2016	21	3.70	0.5000	21	27.40	1.6000	−19.62	[−24.05; −15.18]	0.1%	3.9%
Yong−Wei Fu 2015	54	1.49	0.5900	54	2.23	0.5800	−1.26	[−1.67; −0.84]	10.2%	4.1%
**Common effect model**	**201**			**201**			**−0.88**	**[−1.14; −0.63]**	**26.9%**	**.**
**Random effects model**							**−13.48**	**[−29.57; 2.62]**	**.**	**30.8%**
Heterogeneity: I^2 = 97%, τ^2 = 530.8655, p < 0.01										
**MonthsAfterTRT:Within 6 Mo.**										
Nikolaos Markou 2023	20	17.70	26.4600	20	12.43	17.7000	0.23	[−0.39; 0.85]	4.5%	4.1%
Nikolaos Markou 2023	20	16.67	27.3000	20	32.70	63.3330	−0.32	[−0.95; 0.30]	4.5%	4.1%
Nikolaos Markou 2023	20	10.91	12.3910	20	10.60	13.0000	0.02	[−0.60; 0.64]	4.6%	4.1%
Yong−Wei Fu 2015	54	1.47	0.4200	54	2.23	0.5800	−1.49	[−1.92; −1.06]	9.6%	4.1%
**Common effect model**	**114**			**114**			**−0.63**	**[−0.90; −0.35]**	**23.1%**	**.**
**Random effects model**							**−0.41**	**[−1.19; 0.37]**	**.**	**16.3%**
Heterogeneity: I^2 = 89%, τ^2 = 0.5480, p < 0.01										
**MonthsAfterTRT:6Mo. and more**										
Nikolaos Markou 2023	20	18.25	27.2500	20	12.43	17.7000	0.25	[−0.37; 0.87]	4.5%	4.1%
Nikolaos Markou 2023	20	31.50	51.5150	20	32.70	63.3330	−0.02	[−0.64; 0.60]	4.6%	4.1%
Nikolaos Markou 2023	20	13.00	24.8000	20	10.60	13.0000	0.12	[−0.50; 0.74]	4.5%	4.1%
**Common effect model**	**60**			**60**			**0.12**	**[−0.24; 0.47]**	**13.6%**	**.**
**Random effects model**							**0.12**	**[−0.24; 0.47]**	**.**	**12.2%**
Heterogeneity: I^2 = 0%, τ^2 = 0, p = 0.84										
**Common effect model**	**586**			**586**			**−0.53**	**[−0.66; −0.40]**	**100.0%**	**.**
**Random effects model**							**−4.14**	**[−8.31; 0.04]**	**.**	**100.0%**

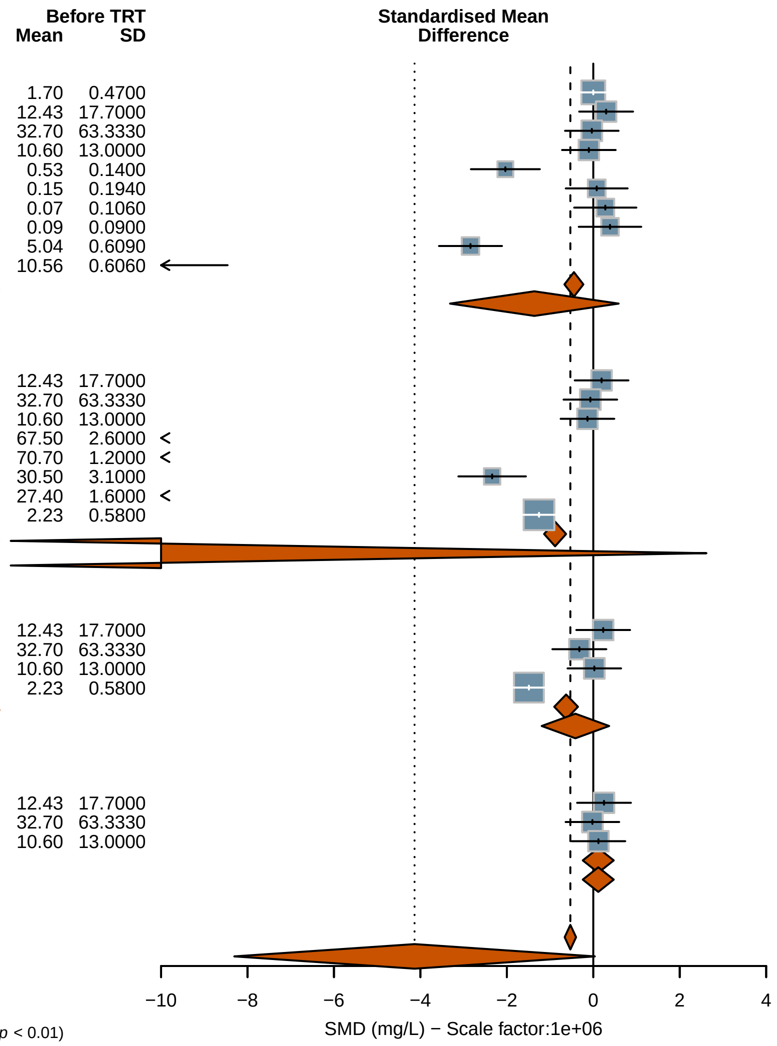

Heterogeneity: I^2 = 95%, τ^2 = 111.0134, p < 0.01
Test for subgroup differences (common effect): χ^2_3 = 20.85, df = 3 (p < 0.01)
Test for subgroup differences (random effects): χ^2_3 = 6.03, df = 3 (p = 0.11)

SMD: −0.53; 95%C.I.[−0.66; −0.4] P value for common effect= 0

SMD: −4.14; 95%C.I.[−8.31; 0.04] P value for random effect= 0.052

Cytokine: IL−1beta − Treatment: Intensive

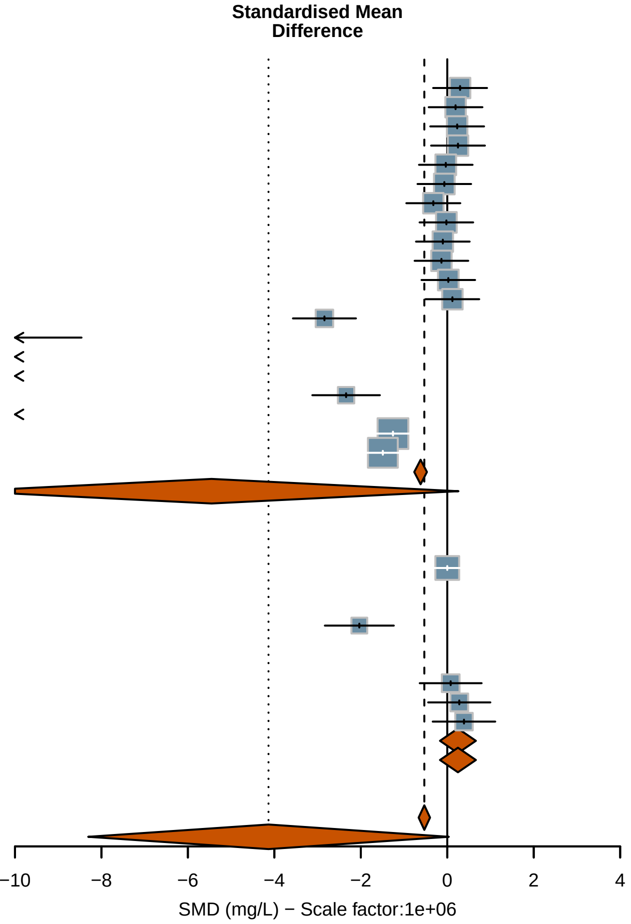

Study	After TRT Total	After TRT Mean	After TRT SD	Before TRT Total	Before TRT Mean	Before TRT SD	SMD	95%−CI	Weight (common)	Weight (random)
**Assay type:ELISA**										
Nikolaos Markou 2023	20	19.58	28.3000	20	12.43	17.7000	0.30	[−0.33; 0.92]	4.5%	4.1%
Nikolaos Markou 2023	20	16.90	27.0000	20	12.43	17.7000	0.19	[−0.43; 0.81]	4.5%	4.1%
Nikolaos Markou 2023	20	17.70	26.4600	20	12.43	17.7000	0.23	[−0.39; 0.85]	4.5%	4.1%
Nikolaos Markou 2023	20	18.25	27.2500	20	12.43	17.7000	0.25	[−0.37; 0.87]	4.5%	4.1%
Nikolaos Markou 2023	20	30.60	57.9000	20	32.70	63.3330	−0.03	[−0.65; 0.59]	4.6%	4.1%
Nikolaos Markou 2023	20	28.80	47.9000	20	32.70	63.3330	−0.07	[−0.69; 0.55]	4.6%	4.1%
Nikolaos Markou 2023	20	16.67	27.3000	20	32.70	63.3330	−0.32	[−0.95; 0.30]	4.5%	4.1%
Nikolaos Markou 2023	20	31.50	51.5150	20	32.70	63.3330	−0.02	[−0.64; 0.60]	4.6%	4.1%
Nikolaos Markou 2023	20	9.39	9.9000	20	10.60	13.0000	−0.10	[−0.72; 0.52]	4.5%	4.1%
Nikolaos Markou 2023	20	8.79	13.3330	20	10.60	13.0000	−0.13	[−0.76; 0.49]	4.5%	4.1%
Nikolaos Markou 2023	20	10.91	12.3910	20	10.60	13.0000	0.02	[−0.60; 0.64]	4.6%	4.1%
Nikolaos Markou 2023	20	13.00	24.8000	20	10.60	13.0000	0.12	[−0.50; 0.74]	4.5%	4.1%
Anwar Mohammad 2020	30	3.45	0.4860	30	5.04	0.6090	−2.84	[−3.57; −2.11]	3.3%	4.1%
Anwar Mohammad 2020	30	4.53	0.5290	30	10.56	0.6060	−10.46	[−12.46; −8.46]	0.4%	4.0%
Javed 2016	22	32.40	0.4000	22	67.50	2.6000	−18.53	[−22.62; −14.44]	0.1%	3.9%
Javed 2016	22	4.50	0.4000	22	70.70	1.2000	−72.68	[−88.57; −56.80]	0.0%	2.6%
Javed 2016	22	13.50	9.6000	22	30.50	3.1000	−2.34	[−3.12; −1.56]	2.9%	4.1%
Javed 2016	21	3.70	0.5000	21	27.40	1.6000	−19.62	[−24.05; −15.18]	0.1%	3.9%
Yong−Wei Fu 2015	54	1.49	0.5900	54	2.23	0.5800	−1.26	[−1.67; −0.84]	10.2%	4.1%
Yong−Wei Fu 2015	54	1.47	0.4200	54	2.23	0.5800	−1.49	[−1.92; −1.06]	9.6%	4.1%
**Common effect model**	**495**			**495**			**−0.62**	**[−0.76; −0.47]**	**81.0%**	**.**
**Random effects model**							**−5.45**	**[−11.17; 0.26]**	**.**	**79.6%**
Heterogeneity: $I^2 = 96\%$, $\tau^2 = 166.8784$, $p < 0.01$										
**Assay type:flow cytometry**										
Fiorini T 2012	27	1.70	0.6300	27	1.70	0.4700	0.00	[−0.53; 0.53]	6.1%	4.1%
**Assay type:Milliplex Inflammation Human Cytokine kit**										
Escobar Arregoces 2021	19	0.30	0.0700	19	0.53	0.1400	−2.03	[−2.83; −1.24]	2.7%	4.1%
**Assay type:ultrasensitive multiplex assay**										
de Sousa Rabelo 2020	15	0.17	0.4090	15	0.15	0.1940	0.08	[−0.64; 0.80]	3.4%	4.1%
de Sousa Rabelo 2020	15	0.13	0.2780	15	0.07	0.1060	0.28	[−0.44; 1.00]	3.4%	4.1%
de Sousa Rabelo 2020	15	0.20	0.3930	15	0.09	0.0900	0.39	[−0.34; 1.11]	3.3%	4.1%
**Common effect model**	**45**			**45**			**0.25**	**[−0.17; 0.66]**	**10.1%**	**.**
**Random effects model**							**0.25**	**[−0.17; 0.66]**	**.**	**12.2%**
Heterogeneity: $I^2 = 0\%$, $\tau^2 = 0$, $p = 0.83$										
**Common effect model**	**586**			**586**			**−0.53**	**[−0.66; −0.40]**	**100.0%**	**.**
**Random effects model**							**−4.14**	**[−8.31; 0.04]**	**.**	**100.0%**

Heterogeneity: $I^2 = 95\%$, $\tau^2 = 111.0134$, $p < 0.01$
Test for subgroup differences (common effect): $\chi^2_3 = 32.22$, df = 3 ($p < 0.01$)
Test for subgroup differences (random effects): $\chi^2_3 = 28.52$, df = 3 ($p < 0.01$)

SMD: −0.53; 95%C.I.[−0.66; −0.4] P value for common effect= 0

SMD: −4.14; 95%C.I.[−8.31; 0.04] P value for random effect= 0.052

Cytokine: IL−1beta − Treatment: Intensive

Study	After TRT Total	After TRT Mean	After TRT SD	Before TRT Total	Before TRT Mean	Before TRT SD	SMD	95%−CI	Weight (common)	Weight (random)
**Bias judgment:High risk**										
Fiorini T 2012	27	1.70	0.6300	27	1.70	0.4700	0.00	[−0.53; 0.53]	6.1%	4.1%
Nikolaos Markou 2023	20	19.58	28.3000	20	12.43	17.7000	0.30	[−0.33; 0.92]	4.5%	4.1%
Nikolaos Markou 2023	20	16.90	27.0000	20	12.43	17.7000	0.19	[−0.43; 0.81]	4.5%	4.1%
Nikolaos Markou 2023	20	17.70	26.4600	20	12.43	17.7000	0.23	[−0.39; 0.85]	4.5%	4.1%
Nikolaos Markou 2023	20	18.25	27.2500	20	12.43	17.7000	0.25	[−0.37; 0.87]	4.5%	4.1%
Nikolaos Markou 2023	20	30.60	57.9000	20	32.70	63.3330	−0.03	[−0.65; 0.59]	4.6%	4.1%
Nikolaos Markou 2023	20	28.80	47.9000	20	32.70	63.3330	−0.07	[−0.69; 0.55]	4.6%	4.1%
Nikolaos Markou 2023	20	16.67	27.3000	20	32.70	63.3330	−0.32	[−0.95; 0.30]	4.5%	4.1%
Nikolaos Markou 2023	20	31.50	51.5150	20	32.70	63.3330	−0.02	[−0.64; 0.60]	4.6%	4.1%
Nikolaos Markou 2023	20	9.39	9.9000	20	10.60	13.0000	−0.10	[−0.72; 0.52]	4.5%	4.1%
Nikolaos Markou 2023	20	8.79	13.3330	20	10.60	13.0000	−0.13	[−0.76; 0.49]	4.5%	4.1%
Nikolaos Markou 2023	20	10.91	12.3910	20	10.60	13.0000	0.02	[−0.60; 0.64]	4.6%	4.1%
Nikolaos Markou 2023	20	13.00	24.8000	20	10.60	13.0000	0.12	[−0.50; 0.74]	4.5%	4.1%
Escobar Arregoces 2021	19	0.30	0.0700	19	0.53	0.1400	−2.03	[−2.83; −1.24]	2.7%	4.1%
de Sousa Rabelo 2020	15	0.17	0.4090	15	0.15	0.1940	0.08	[−0.64; 0.80]	3.4%	4.1%
de Sousa Rabelo 2020	15	0.13	0.2780	15	0.07	0.1060	0.28	[−0.44; 1.00]	3.4%	4.1%
de Sousa Rabelo 2020	15	0.20	0.3930	15	0.09	0.0900	0.39	[−0.34; 1.11]	3.3%	4.1%
**Common effect model**	**331**			**331**			**−0.02**	**[−0.17; 0.14]**	**73.4%**	**.**
**Random effects model**							**−0.03**	**[−0.23; 0.18]**	**.**	**69.3%**
Heterogeneity: I^2 = 48%, τ^2 = 0.0802, p = 0.02										
**Bias judgment:Low risk**										
Anwar Mohammad 2020	30	3.45	0.4860	30	5.04	0.6090	−2.84	[−3.57; −2.11]	3.3%	4.1%
Anwar Mohammad 2020	30	4.53	0.5290	30	10.56	0.6060	−10.46	[−12.46; −8.46]	0.4%	4.0%
Yong−Wei Fu 2015	54	1.49	0.5900	54	2.23	0.5800	−1.26	[−1.67; −0.84]	10.2%	4.1%
Yong−Wei Fu 2015	54	1.47	0.4200	54	2.23	0.5800	−1.49	[−1.92; −1.06]	9.6%	4.1%
**Common effect model**	**168**			**168**			**−1.75**	**[−2.02; −1.47]**	**23.5%**	**.**
**Random effects model**							**−3.93**	**[−8.09; 0.23]**	**.**	**16.3%**
Heterogeneity: I^2 = 97%, τ^2 = 17.7144, p < 0.01										
**Bias judgment:Some concerns**										
Javed 2016	22	32.40	0.4000	22	67.50	2.6000	−18.53	[−22.62; −14.44]	0.1%	3.9%
Javed 2016	22	4.50	0.4000	22	70.70	1.2000	−72.68	[−88.57; −56.80]	0.0%	2.6%
Javed 2016	22	13.50	9.6000	22	30.50	3.1000	−2.34	[−3.12; −1.56]	2.9%	4.1%
Javed 2016	21	3.70	0.5000	21	27.40	1.6000	−19.62	[−24.05; −15.18]	0.1%	3.9%
**Common effect model**	**87**			**87**			**−3.55**	**[−4.31; −2.80]**	**3.1%**	**.**
**Random effects model**							**−27.52**	**[−56.62; 1.58]**	**.**	**14.5%**
Heterogeneity: I^2 = 98%, τ^2 = 863.5565, p < 0.01										
**Common effect model**	**586**			**586**			**−0.53**	**[−0.66; −0.40]**	**100.0%**	**.**
**Random effects model**							**−4.14**	**[−8.31; 0.04]**	**.**	**100.0%**

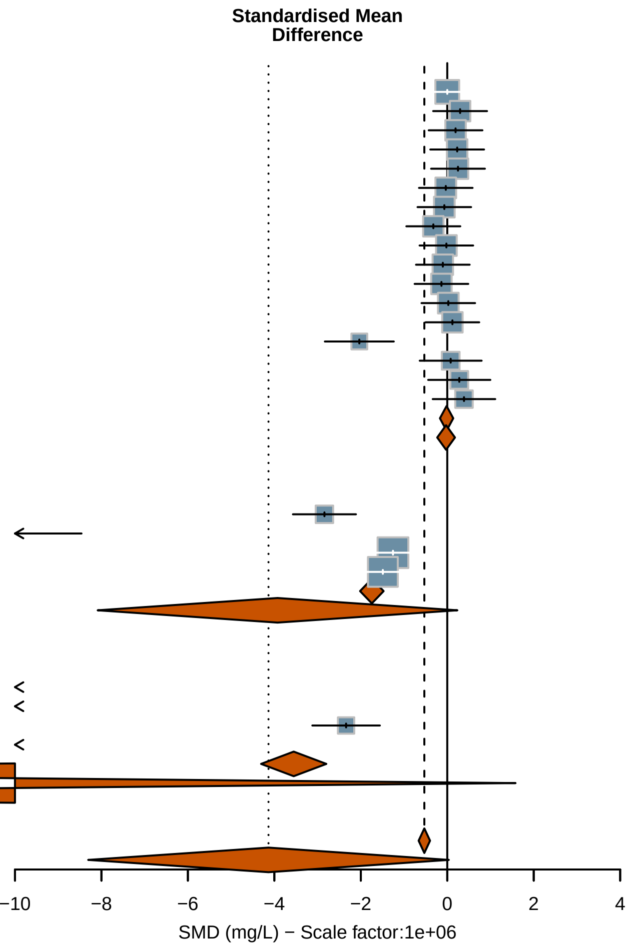

Heterogeneity: I^2 = 95%, τ^2 = 111.0134, p < 0.01
Test for subgroup differences (common effect): χ^2_2 = 180.26, df = 2 (p < 0.01)
Test for subgroup differences (random effects): χ^2_2 = 6.79, df = 2 (p = 0.03)

SMD: −0.53; 95%C.I.[−0.66; −0.4] P value for common effect= 0

SMD: −4.14; 95%C.I.[−8.31; 0.04] P value for random effect= 0.052

Cytokine: IL−1beta − Treatment: Intensive

Study	After TRT Total	After TRT Mean	After TRT SD	Before TRT Total	Before TRT Mean	Before TRT SD	SMD	95%−CI	Weight (common)	Weight (random)
**Study design:Clinical trial**										
de Sousa Rabelo 2020	15	0.17	0.4090	15	0.15	0.1940	0.08	[−0.64; 0.80]	3.4%	4.1%
de Sousa Rabelo 2020	15	0.13	0.2780	15	0.07	0.1060	0.28	[−0.44; 1.00]	3.4%	4.1%
de Sousa Rabelo 2020	15	0.20	0.3930	15	0.09	0.0900	0.39	[−0.34; 1.11]	3.3%	4.1%
**Common effect model**	**45**			**45**			**0.25**	**[−0.17; 0.66]**	**10.1%**	**.**
**Random effects model**							**0.25**	**[−0.17; 0.66]**	**.**	**12.2%**
Heterogeneity: $I^2 = 0\%$, $\tau^2 = 0$, $p = 0.83$										
**Study design:Comparative study**										
Anwar Mohammad 2020	30	3.45	0.4860	30	5.04	0.6090	−2.84	[−3.57; −2.11]	3.3%	4.1%
Anwar Mohammad 2020	30	4.53	0.5290	30	10.56	0.6060	−10.46	[−12.46; −8.46]	0.4%	4.0%
**Common effect model**	**60**			**60**			**−3.74**	**[−4.42; −3.05]**	**3.7%**	**.**
**Random effects model**							**−6.59**	**[−14.05; 0.87]**	**.**	**8.1%**
Heterogeneity: $I^2 = 98\%$, $\tau^2 = 28.4201$, $p < 0.01$										
**Study design:Observational study**										
Escobar Arregoces 2021	19	0.30	0.0700	19	0.53	0.1400	−2.03	[−2.83; −1.24]	2.7%	4.1%
**Study design:RCT**										
Fiorini T 2012	27	1.70	0.6300	27	1.70	0.4700	0.00	[−0.53; 0.53]	6.1%	4.1%
Nikolaos Markou 2023	20	19.58	28.3000	20	12.43	17.7000	0.30	[−0.33; 0.92]	4.5%	4.1%
Nikolaos Markou 2023	20	16.90	27.0000	20	12.43	17.7000	0.19	[−0.43; 0.81]	4.5%	4.1%
Nikolaos Markou 2023	20	17.70	26.4600	20	12.43	17.7000	0.23	[−0.39; 0.85]	4.5%	4.1%
Nikolaos Markou 2023	20	18.25	27.2500	20	12.43	17.7000	0.25	[−0.37; 0.87]	4.5%	4.1%
Nikolaos Markou 2023	20	30.60	57.9000	20	32.70	63.3330	−0.03	[−0.65; 0.59]	4.6%	4.1%
Nikolaos Markou 2023	20	28.80	47.9000	20	32.70	63.3330	−0.07	[−0.69; 0.55]	4.6%	4.1%
Nikolaos Markou 2023	20	16.67	27.3000	20	32.70	63.3330	−0.32	[−0.95; 0.30]	4.5%	4.1%
Nikolaos Markou 2023	20	31.50	51.5150	20	32.70	63.3330	−0.02	[−0.64; 0.60]	4.6%	4.1%
Nikolaos Markou 2023	20	9.39	9.9000	20	10.60	13.0000	−0.10	[−0.72; 0.52]	4.5%	4.1%
Nikolaos Markou 2023	20	8.79	13.3330	20	10.60	13.0000	−0.13	[−0.76; 0.49]	4.5%	4.1%
Nikolaos Markou 2023	20	10.91	12.3910	20	10.60	13.0000	0.02	[−0.60; 0.64]	4.6%	4.1%
Nikolaos Markou 2023	20	13.00	24.8000	20	10.60	13.0000	0.12	[−0.50; 0.74]	4.5%	4.1%
Javed 2016	22	32.40	0.4000	22	67.50	2.6000	−18.53	[−22.62; −14.44]	0.1%	3.9%
Javed 2016	22	4.50	0.4000	22	70.70	1.2000	−72.68	[−88.57; −56.80]	0.0%	2.6%
Javed 2016	22	13.50	9.6000	22	30.50	3.1000	−2.34	[−3.12; −1.56]	2.9%	4.1%
Javed 2016	21	3.70	0.5000	21	27.40	1.6000	−19.62	[−24.05; −15.18]	0.1%	3.9%
Yong−Wei Fu 2015	54	1.49	0.5900	54	2.23	0.5800	−1.26	[−1.67; −0.84]	10.2%	4.1%
Yong−Wei Fu 2015	54	1.47	0.4200	54	2.23	0.5800	−1.49	[−1.92; −1.06]	9.6%	4.1%
**Common effect model**	**462**			**462**			**−0.43**	**[−0.58; −0.29]**	**83.4%**	**.**
**Random effects model**							**−5.10**	**[−11.21; 1.01]**	**.**	**75.6%**
Heterogeneity: $I^2 = 94\%$, $\tau^2 = 181.5769$, $p < 0.01$										
**Common effect model**	**586**			**586**			**−0.53**	**[−0.66; −0.40]**	**100.0%**	**.**
**Random effects model**							**−4.14**	**[−8.31; 0.04]**	**.**	**100.0%**

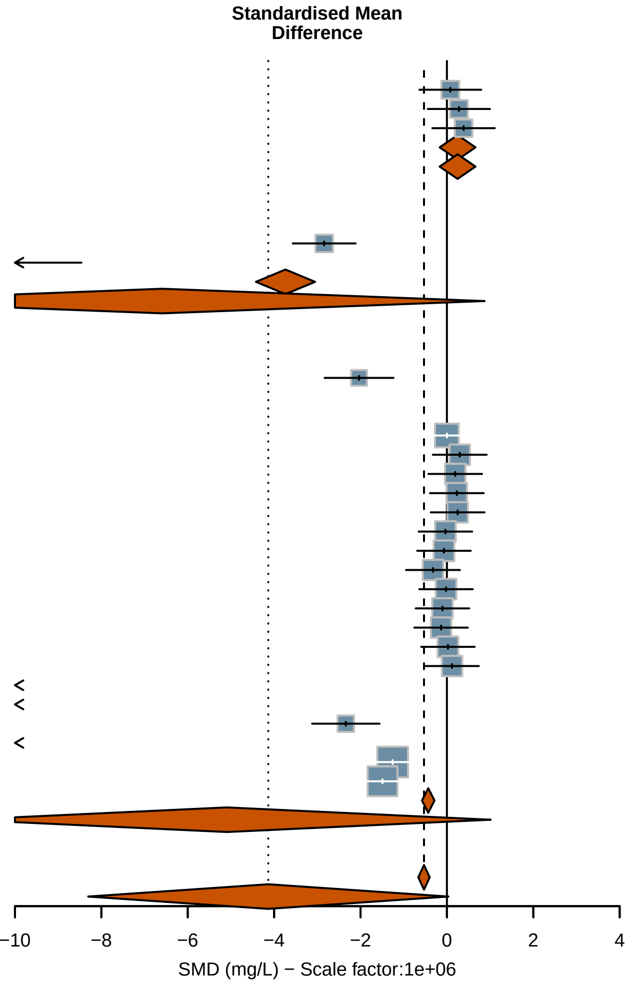

Heterogeneity: $I^2 = 95\%$, $\tau^2 = 111.0134$, $p < 0.01$
Test for subgroup differences (common effect): $\chi^2_3 = 113.04$, df = 3 ($p < 0.01$)
Test for subgroup differences (random effects): $\chi^2_3 = 29.88$, df = 3 ($p < 0.01$)

SMD: −0.53; 95%C.I.[−0.66; −0.4] P value for common effect= 0

SMD: −4.14; 95%C.I.[−8.31; 0.04] P value for random effect= 0.052

Cytokine: IL−1beta − Treatment: Intensive

Study	After TRT Total	After TRT Mean	After TRT SD	Before TRT Total	Before TRT Mean	Before TRT SD		SMD	95%−CI	Weight (common)	Weight (random)
**Additional_devices:curcumin gel**											
Anwar Mohammad 2020	30	3.45	0.4860	30	5.04	0.6090		−2.84	[−3.57; −2.11]	5.5%	7.8%
**Additional_devices:laser 2 W, 200 mJ, 10Hz**											
Nikolaos Markou 2023	20	9.39	9.9000	20	10.60	13.0000		−0.10	[−0.72; 0.52]	7.6%	7.8%
Nikolaos Markou 2023	20	8.79	13.3330	20	10.60	13.0000		−0.13	[−0.76; 0.49]	7.6%	7.8%
Nikolaos Markou 2023	20	10.91	12.3910	20	10.60	13.0000		0.02	[−0.60; 0.64]	7.7%	7.8%
Nikolaos Markou 2023	20	13.00	24.8000	20	10.60	13.0000		0.12	[−0.50; 0.74]	7.6%	7.8%
**Common effect model**	**80**			**80**				**−0.02**	**[−0.33; 0.29]**	**30.6%**	**.**
**Random effects model**								**−0.02**	**[−0.33; 0.29]**	**.**	**31.2%**
Heterogeneity: $I^2 = 0\%$, $\tau^2 = 0$, $p = 0.94$											
**Additional_devices:Laser 3 W, 150mJ, 20Hz**											
Nikolaos Markou 2023	20	30.60	57.9000	20	32.70	63.3330		−0.03	[−0.65; 0.59]	7.7%	7.8%
Nikolaos Markou 2023	20	28.80	47.9000	20	32.70	63.3330		−0.07	[−0.69; 0.55]	7.7%	7.8%
Nikolaos Markou 2023	20	16.67	27.3000	20	32.70	63.3330		−0.32	[−0.95; 0.30]	7.5%	7.8%
Nikolaos Markou 2023	20	31.50	51.5150	20	32.70	63.3330		−0.02	[−0.64; 0.60]	7.7%	7.8%
**Common effect model**	**80**			**80**				**−0.11**	**[−0.42; 0.20]**	**30.5%**	**.**
**Random effects model**								**−0.11**	**[−0.42; 0.20]**	**.**	**31.2%**
Heterogeneity: $I^2 = 0\%$, $\tau^2 = 0$, $p = 0.90$											
**Additional_devices:laser Nd:YAG**											
Javed 2016	22	4.50	0.4000	22	70.70	1.2000	<	−72.68	[−88.57; −56.80]	0.0%	6.4%
Javed 2016	21	3.70	0.5000	21	27.40	1.6000	<	−19.62	[−24.05; −15.18]	0.1%	7.7%
**Common effect model**	**43**			**43**				**−23.46**	**[−27.73; −19.18]**	**0.2%**	**.**
**Random effects model**								**−45.58**	**[−97.57; 6.41]**	**.**	**14.1%**
Heterogeneity: $I^2 = 97\%$, $\tau^2 = 1372.5887$, $p < 0.01$											
**Additional_devices:Protocollo full mouth disinfection**											
Yong−Wei Fu 2015	54	1.49	0.5900	54	2.23	0.5800		−1.26	[−1.67; −0.84]	17.2%	7.8%
Yong−Wei Fu 2015	54	1.47	0.4200	54	2.23	0.5800		−1.49	[−1.92; −1.06]	16.1%	7.8%
**Common effect model**	**108**			**108**				**−1.37**	**[−1.67; −1.07]**	**33.2%**	**.**
**Random effects model**								**−1.37**	**[−1.67; −1.07]**	**.**	**15.6%**
Heterogeneity: $I^2 = 0\%$, $\tau^2 = 0$, $p = 0.44$											
**Common effect model**	**341**			**341**				**−0.69**	**[−0.86; −0.52]**	**100.0%**	**.**
**Random effects model**								**−6.63**	**[−16.07; 2.80]**	**.**	**100.0%**

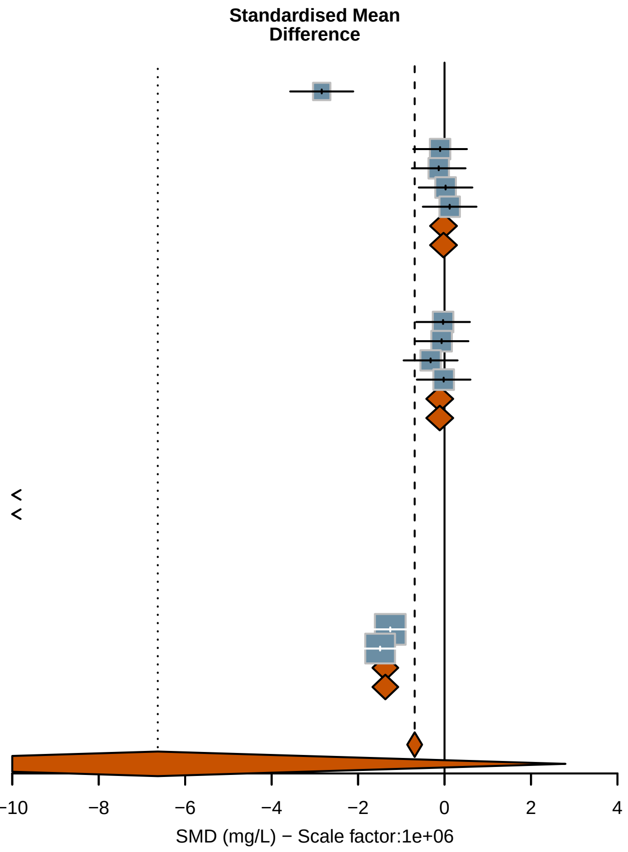

Heterogeneity: $I^2 = 95\%$, $\tau^2 = 296.3376$, $p < 0.01$
Test for subgroup differences (common effect): $\chi^2_4 = 193.59$, df = 4 ($p < 0.01$)
Test for subgroup differences (random effects): $\chi^2_4 = 87.27$, df = 4 ($p < 0.01$)

SMD: −0.69; 95%C.I.[−0.86; −0.52] P value for common effect= 0

SMD: −6.63; 95%C.I.[−16.07; 2.8] P value for random effect= 0.168

Cytokine: IL−1beta − Treatment: Intensive

Study	After TRT Total	After TRT Mean	After TRT SD	Before TRT Total	Before TRT Mean	Before TRT SD		SMD	95%−CI	Weight (common)	Weight (random)
**Female_binary:Only Female**											
Fiorini T 2012	27	1.70	0.6300	27	1.70	0.4700		0.00	[−0.53; 0.53]	6.1%	4.1%
**Female_binary:Only Male**											
Javed 2016	22	32.40	0.4000	22	67.50	2.6000	<	−18.53	[−22.62; −14.44]	0.1%	3.9%
Javed 2016	22	4.50	0.4000	22	70.70	1.2000	<	−72.68	[−88.57; −56.80]	0.0%	2.6%
Javed 2016	22	13.50	9.6000	22	30.50	3.1000		−2.34	[−3.12; −1.56]	2.9%	4.1%
Javed 2016	21	3.70	0.5000	21	27.40	1.6000	<	−19.62	[−24.05; −15.18]	0.1%	3.9%
**Common effect model**	**87**			**87**				**−3.55**	**[−4.31; −2.80]**	**3.1%**	**.**
**Random effects model**								**−27.52**	**[−56.62; 1.58]**	**.**	**14.5%**
Heterogeneity: I^2 = 98%, τ^2 = 863.5565, p < 0.01											
**Female_binary:Sex mixed**											
Nikolaos Markou 2023	20	19.58	28.3000	20	12.43	17.7000		0.30	[−0.33; 0.92]	4.5%	4.1%
Nikolaos Markou 2023	20	16.90	27.0000	20	12.43	17.7000		0.19	[−0.43; 0.81]	4.5%	4.1%
Nikolaos Markou 2023	20	17.70	26.4600	20	12.43	17.7000		0.23	[−0.39; 0.85]	4.5%	4.1%
Nikolaos Markou 2023	20	18.25	27.2500	20	12.43	17.7000		0.25	[−0.37; 0.87]	4.5%	4.1%
Nikolaos Markou 2023	20	30.60	57.9000	20	32.70	63.3330		−0.03	[−0.65; 0.59]	4.6%	4.1%
Nikolaos Markou 2023	20	28.80	47.9000	20	32.70	63.3330		−0.07	[−0.69; 0.55]	4.6%	4.1%
Nikolaos Markou 2023	20	16.67	27.3000	20	32.70	63.3330		−0.32	[−0.95; 0.30]	4.5%	4.1%
Nikolaos Markou 2023	20	31.50	51.5150	20	32.70	63.3330		−0.02	[−0.64; 0.60]	4.6%	4.1%
Nikolaos Markou 2023	20	9.39	9.9000	20	10.60	13.0000		−0.10	[−0.72; 0.52]	4.5%	4.1%
Nikolaos Markou 2023	20	8.79	13.3330	20	10.60	13.0000		−0.13	[−0.76; 0.49]	4.5%	4.1%
Nikolaos Markou 2023	20	10.91	12.3910	20	10.60	13.0000		0.02	[−0.60; 0.64]	4.6%	4.1%
Nikolaos Markou 2023	20	13.00	24.8000	20	10.60	13.0000		0.12	[−0.50; 0.74]	4.5%	4.1%
Escobar Arregoces 2021	19	0.30	0.0700	19	0.53	0.1400		−2.03	[−2.83; −1.24]	2.7%	4.1%
de Sousa Rabelo 2020	15	0.17	0.4090	15	0.15	0.1940		0.08	[−0.64; 0.80]	3.4%	4.1%
de Sousa Rabelo 2020	15	0.13	0.2780	15	0.07	0.1060		0.28	[−0.44; 1.00]	3.4%	4.1%
de Sousa Rabelo 2020	15	0.20	0.3930	15	0.09	0.0900		0.39	[−0.34; 1.11]	3.3%	4.1%
Anwar Mohammad 2020	30	3.45	0.4860	30	5.04	0.6090		−2.84	[−3.57; −2.11]	3.3%	4.1%
Anwar Mohammad 2020	30	4.53	0.5290	30	10.56	0.6060	←	−10.46	[−12.46; −8.46]	0.4%	4.0%
Yong−Wei Fu 2015	54	1.49	0.5900	54	2.23	0.5800		−1.26	[−1.67; −0.84]	10.2%	4.1%
Yong−Wei Fu 2015	54	1.47	0.4200	54	2.23	0.5800		−1.49	[−1.92; −1.06]	9.6%	4.1%
**Common effect model**	**472**			**472**				**−0.46**	**[−0.60; −0.33]**	**90.8%**	**.**
**Random effects model**								**−0.76**	**[−1.67; 0.16]**	**.**	**81.5%**
Heterogeneity: I^2 = 92%, τ^2 = 4.2106, p < 0.01											
**Common effect model**	**586**			**586**				**−0.53**	**[−0.66; −0.40]**	**100.0%**	**.**
**Random effects model**								**−4.14**	**[−8.31; 0.04]**	**.**	**100.0%**

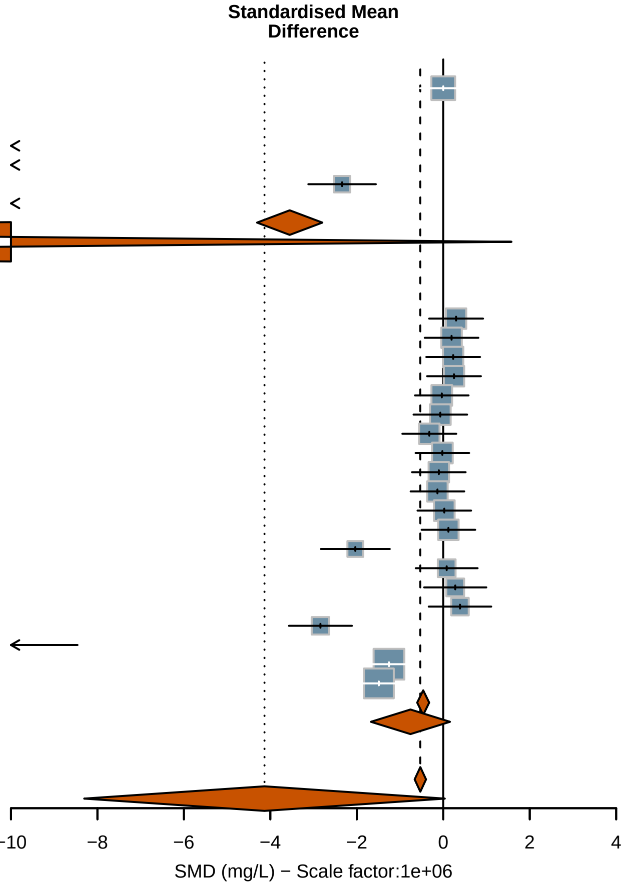

Heterogeneity: I^2 = 95%, τ^2 = 111.0134, p < 0.01
Test for subgroup differences (common effect): χ^2_2 = 66.14, df = 2 (p < 0.01)
Test for subgroup differences (random effects): χ^2_2 = 5.36, df = 2 (p = 0.07)

SMD: −0.53; 95%C.I.[−0.66; −0.4] P value for common effect= 0

SMD: −4.14; 95%C.I.[−8.31; 0.04] P value for random effect= 0.052

Cytokine: IL−1beta − Treatment: Intensive

Study	After TRT Total	After TRT Mean	After TRT SD	Before TRT Total	Before TRT Mean	Before TRT SD	SMD	95%−CI	Weight (common)	Weight (random)
**Smoke_binary:Sample with smokers**										
Fiorini T 2012	27	1.70	0.6300	27	1.70	0.4700	0.00	[−0.53; 0.53]	6.1%	4.1%
Nikolaos Markou 2023	20	19.58	28.3000	20	12.43	17.7000	0.30	[−0.33; 0.92]	4.5%	4.1%
Nikolaos Markou 2023	20	16.90	27.0000	20	12.43	17.7000	0.19	[−0.43; 0.81]	4.5%	4.1%
Nikolaos Markou 2023	20	17.70	26.4600	20	12.43	17.7000	0.23	[−0.39; 0.85]	4.5%	4.1%
Nikolaos Markou 2023	20	18.25	27.2500	20	12.43	17.7000	0.25	[−0.37; 0.87]	4.5%	4.1%
Nikolaos Markou 2023	20	30.60	57.9000	20	32.70	63.3330	−0.03	[−0.65; 0.59]	4.6%	4.1%
Nikolaos Markou 2023	20	28.80	47.9000	20	32.70	63.3330	−0.07	[−0.69; 0.55]	4.6%	4.1%
Nikolaos Markou 2023	20	16.67	27.3000	20	32.70	63.3330	−0.32	[−0.95; 0.30]	4.5%	4.1%
Nikolaos Markou 2023	20	31.50	51.5150	20	32.70	63.3330	−0.02	[−0.64; 0.60]	4.6%	4.1%
Nikolaos Markou 2023	20	9.39	9.9000	20	10.60	13.0000	−0.10	[−0.72; 0.52]	4.5%	4.1%
Nikolaos Markou 2023	20	8.79	13.3330	20	10.60	13.0000	−0.13	[−0.76; 0.49]	4.5%	4.1%
Nikolaos Markou 2023	20	10.91	12.3910	20	10.60	13.0000	0.02	[−0.60; 0.64]	4.6%	4.1%
Nikolaos Markou 2023	20	13.00	24.8000	20	10.60	13.0000	0.12	[−0.50; 0.74]	4.5%	4.1%
Yong−Wei Fu 2015	54	1.49	0.5900	54	2.23	0.5800	−1.26	[−1.67; −0.84]	10.2%	4.1%
Yong−Wei Fu 2015	54	1.47	0.4200	54	2.23	0.5800	−1.49	[−1.92; −1.06]	9.6%	4.1%
**Common effect model**	**375**			**375**			**−0.31**	**[−0.46; −0.17]**	**80.3%**	**.**
**Random effects model**							**−0.18**	**[−0.48; 0.11]**	**.**	**61.1%**
Heterogeneity: I^2 = 80%, τ^2 = 0.2464, p < 0.01										
**Smoke_binary:Sample without smokers**										
Escobar Arregoces 2021	19	0.30	0.0700	19	0.53	0.1400	−2.03	[−2.83; −1.24]	2.7%	4.1%
de Sousa Rabelo 2020	15	0.17	0.4090	15	0.15	0.1940	0.08	[−0.64; 0.80]	3.4%	4.1%
de Sousa Rabelo 2020	15	0.13	0.2780	15	0.07	0.1060	0.28	[−0.44; 1.00]	3.4%	4.1%
de Sousa Rabelo 2020	15	0.20	0.3930	15	0.09	0.0900	0.39	[−0.34; 1.11]	3.3%	4.1%
Anwar Mohammad 2020	30	3.45	0.4860	30	5.04	0.6090	−2.84	[−3.57; −2.11]	3.3%	4.1%
Anwar Mohammad 2020	30	4.53	0.5290	30	10.56	0.6060	−10.46	[−12.46; −8.46]	0.4%	4.0%
Javed 2016	22	32.40	0.4000	22	67.50	2.6000	−18.53	[−22.62; −14.44]	0.1%	3.9%
Javed 2016	22	4.50	0.4000	22	70.70	1.2000	−72.68	[−88.57; −56.80]	0.0%	2.6%
Javed 2016	22	13.50	9.6000	22	30.50	3.1000	−2.34	[−3.12; −1.56]	2.9%	4.1%
Javed 2016	21	3.70	0.5000	21	27.40	1.6000	−19.62	[−24.05; −15.18]	0.1%	3.9%
**Common effect model**	**211**			**211**			**−1.42**	**[−1.72; −1.12]**	**19.7%**	**.**
**Random effects model**							**−11.91**	**[−24.42; 0.60]**	**.**	**38.9%**
Heterogeneity: I^2 = 98%, τ^2 = 400.4881, p < 0.01										
**Common effect model**	**586**			**586**			**−0.53**	**[−0.66; −0.40]**	**100.0%**	**.**
**Random effects model**							**−4.14**	**[−8.31; 0.04]**	**.**	**100.0%**

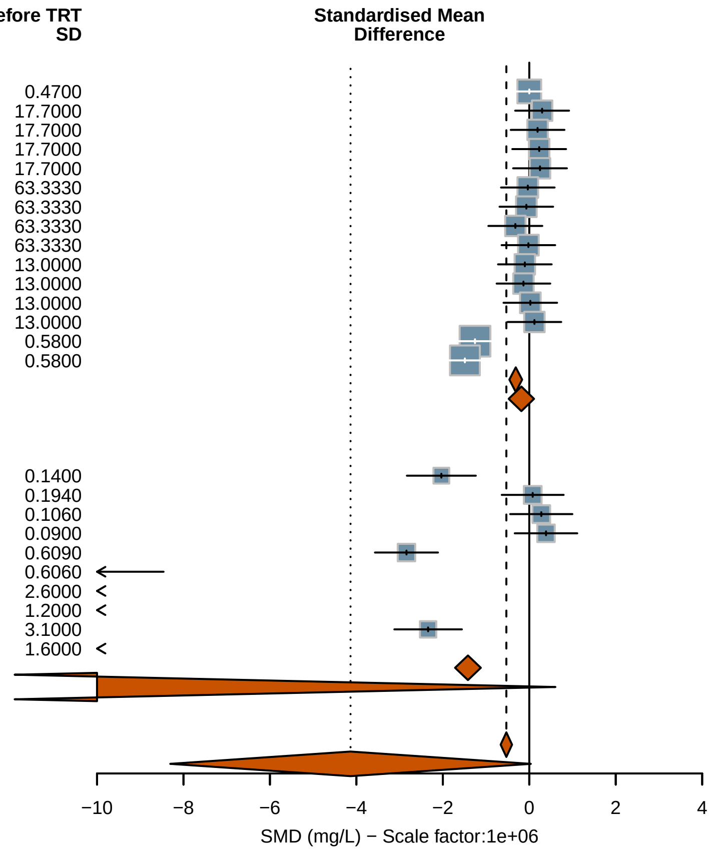

Heterogeneity: I^2 = 95%, τ^2 = 111.0134, p < 0.01
Test for subgroup differences (common effect): χ^2_1 = 42.43, df = 1 (p < 0.01)
Test for subgroup differences (random effects): χ^2_1 = 3.37, df = 1 (p = 0.07)

SMD: −0.53; 95%C.I.[−0.66; −0.4] P value for common effect= 0

SMD: −4.14; 95%C.I.[−8.31; 0.04] P value for random effect= 0.052

Cytokine: IL−1beta − Treatment: Intensive

Study	After TRT Total	After TRT Mean	After TRT SD	Before TRT Total	Before TRT Mean	Before TRT SD	SMD	95%−CI	Weight (common)	Weight (random)
**Diabetes_binary:Sample with diabetic**										
de Sousa Rabelo 2020	15	0.20	0.3930	15	0.09	0.0900	0.39	[−0.34; 1.11]	8.7%	10.2%
**Diabetes_binary:Sample without diabetic**										
Fiorini T 2012	27	1.70	0.6300	27	1.70	0.4700	0.00	[−0.53; 0.53]	16.0%	10.2%
Escobar Arregoces 2021	19	0.30	0.0700	19	0.53	0.1400	−2.03	[−2.83; −1.24]	7.1%	10.2%
de Sousa Rabelo 2020	15	0.17	0.4090	15	0.15	0.1940	0.08	[−0.64; 0.80]	8.9%	10.2%
Javed 2016	22	32.40	0.4000	22	67.50	2.6000	−18.53	[−22.62; −14.44]	0.3%	10.1%
Javed 2016	22	4.50	0.4000	22	70.70	1.2000	−72.68	[−88.57; −56.80]	0.0%	8.8%
Javed 2016	22	13.50	9.6000	22	30.50	3.1000	−2.34	[−3.12; −1.56]	7.4%	10.2%
Javed 2016	21	3.70	0.5000	21	27.40	1.6000	−19.62	[−24.05; −15.18]	0.2%	10.0%
Yong−Wei Fu 2015	54	1.49	0.5900	54	2.23	0.5800	−1.26	[−1.67; −0.84]	26.5%	10.2%
Yong−Wei Fu 2015	54	1.47	0.4200	54	2.23	0.5800	−1.49	[−1.92; −1.06]	24.8%	10.2%
**Common effect model**	**256**			**256**			**−1.23**	**[−1.45; −1.01]**	**91.3%**	**.**
**Random effects model**							**−12.25**	**[−26.40; 1.90]**	**.**	**89.8%**
Heterogeneity: $I^2 = 97\%$, $\tau^2 = 461.4865$, $p < 0.01$										
**Common effect model**	**271**			**271**			**−1.09**	**[−1.30; −0.88]**	**100.0%**	**.**
**Random effects model**							**−10.89**	**[−23.62; 1.84]**	**.**	**100.0%**

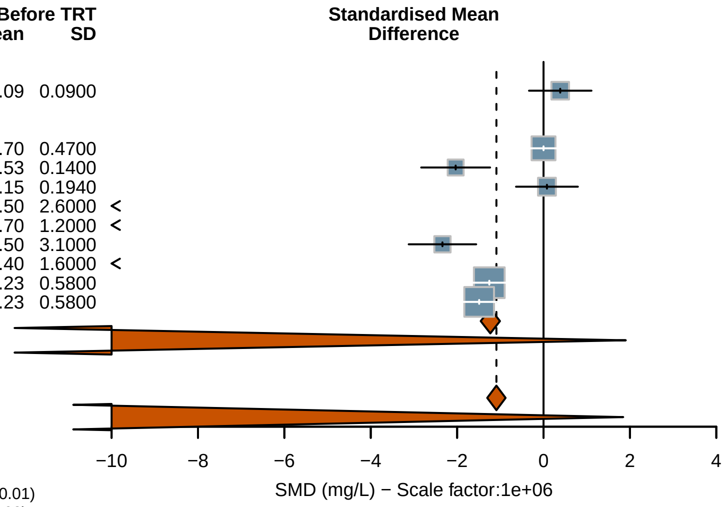

Heterogeneity: $I^2 = 97\%$, $\tau^2 = 415.2184$, $p < 0.01$
Test for subgroup differences (common effect): $\chi^2_1 = 17.56$, df = 1 ($p < 0.01$)
Test for subgroup differences (random effects): $\chi^2_1 = 3.05$, df = 1 ($p = 0.08$)

SMD: −1.09; 95%C.I.[−1.3; −0.88] P value for common effect= 0

SMD: −10.89; 95%C.I.[−23.62; 1.84] P value for random effect= 0.0937

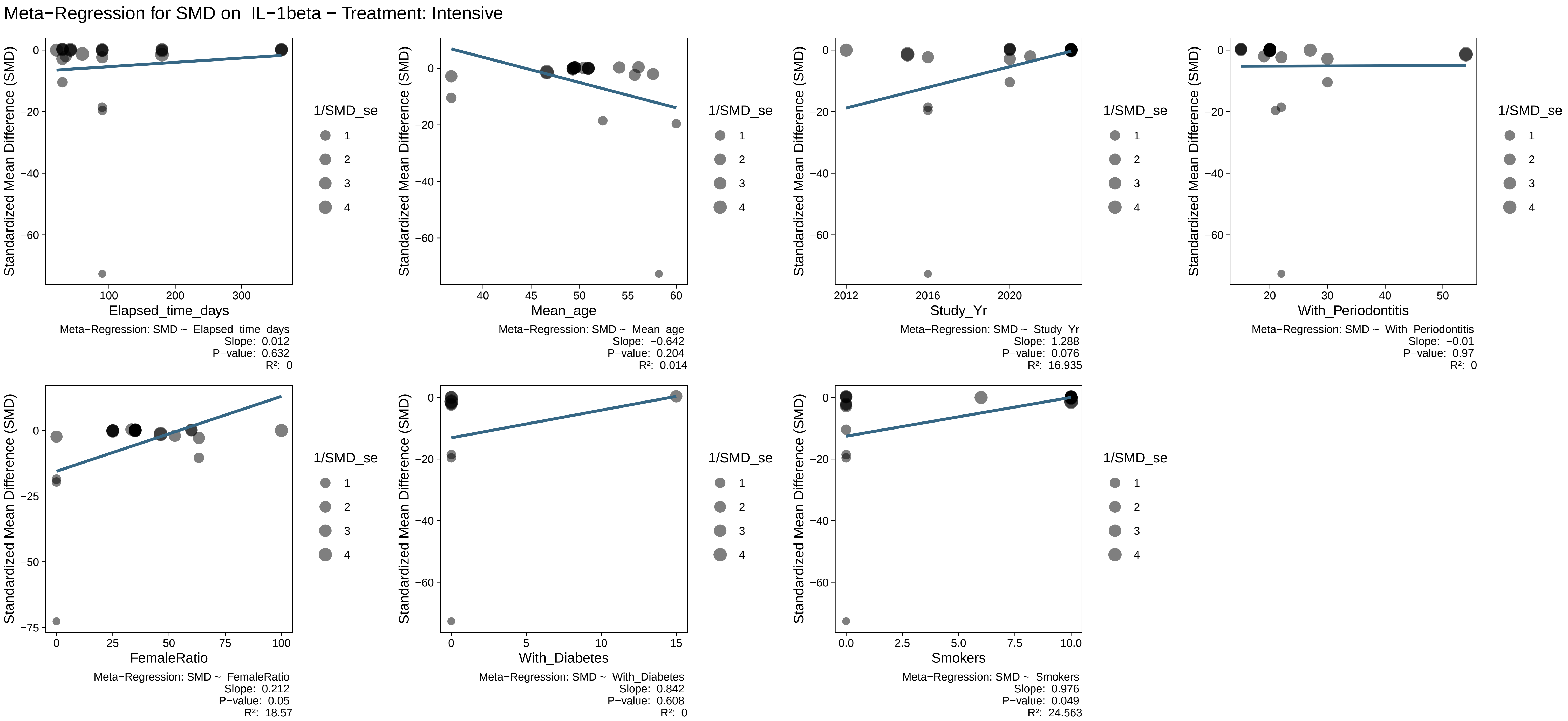

Meta−Regression for SMD on IL−1beta − Treatment: Intensive
Standardized Mean Difference (SMD)
1/SMD_se
Elapsed_time_days
Meta−Regression: SMD ~ Elapsed_time_days
Slope: 0.012
P−value: 0.632
R²: 0
Mean_age
Meta−Regression: SMD ~ Mean_age
Slope: −0.642
P−value: 0.204
R²: 0.014
Study_Yr
Meta−Regression: SMD ~ Study_Yr
Slope: 1.288
P−value: 0.076
R²: 16.935
With_Periodontitis
Meta−Regression: SMD ~ With_Periodontitis
Slope: −0.01
P−value: 0.97
R²: 0
FemaleRatio
Meta−Regression: SMD ~ FemaleRatio
Slope: 0.212
P−value: 0.05
R²: 18.57
With_Diabetes
Meta−Regression: SMD ~ With_Diabetes
Slope: 0.842
P−value: 0.608
R²: 0
Smokers
Meta−Regression: SMD ~ Smokers
Slope: 0.976
P−value: 0.049
R²: 24.563
