# Supplementary material for: Temporal dynamics of early inflammatory markers after professional dental cleaning: a meta-analysis and spline-based meta-regression of TNF-α, IL-1β, IL-6, and (hs)CRP
Source: Front Immunol. 2025 Aug 28;16:1634622. doi: 10.3389/fimmu.2025.1634622 (PMC12423065; doi:10.3389/fimmu.2025.1634622)
Supplement: Supplementary file 1 [file DataSheet1.zip › Supplementary materials/PDF/IL-1beta_Standard_results.pdf]

Cytokine: IL−1beta − Treatment: Standard

Study	After TRT Total	After TRT Mean	After TRT SD	Before TRT Total	Before TRT Mean	Before TRT SD	SMD	95%−CI	Weight (common)	Weight (random)
**Days after treatment:42**										
Ide M 2003	24	0.89	0.5900	24	0.95	0.6700	−0.09	[−0.66; 0.47]	17.9%	17.9%
**Days after treatment:60**										
Yong−Wei Fu 2015	55	2.34	0.6500	55	2.29	0.5900	0.08	[−0.29; 0.45]	41.1%	41.1%
**Days after treatment:180**										
Yong−Wei Fu 2015	55	2.19	0.7200	55	2.29	0.5900	−0.15	[−0.53; 0.22]	41.0%	41.0%
**Common effect model**	**134**			**134**			**−0.05**	**[−0.29; 0.19]**	**100.0%**	**.**
**Random effects model**							**−0.05**	**[−0.29; 0.19]**	**.**	**100.0%**

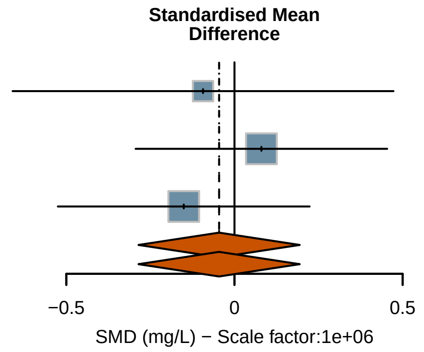

Heterogeneity: $I^2 = 0\%$, $\tau^2 = 0$, $p = 0.68$
Test for subgroup differences (common effect): $\chi^2_2 = 0.76$, df = 2 ($p = 0.68$)
Test for subgroup differences (random effects): $\chi^2_2 = 0.76$, df = 2 ($p = 0.68$)

SMD: −0.05; 95%C.I.[−0.29; 0.19] P value for common effect= 0.7083

SMD: −0.05; 95%C.I.[−0.29; 0.19] P value for random effect= 0.7083

Cytokine: IL−1beta − Treatment: Standard

Study	After TRT Total	After TRT Mean	After TRT SD	Before TRT Total	Before TRT Mean	Before TRT SD	SMD	95%−CI	Weight (common)	Weight (random)
**MonthsAfterTRT:4−6Wks**										
Ide M 2003	24	0.89	0.5900	24	0.95	0.6700	−0.09	[−0.66; 0.47]	17.9%	17.9%
**MonthsAfterTRT:Within 3 Mo.**										
Yong−Wei Fu 2015	55	2.34	0.6500	55	2.29	0.5900	0.08	[−0.29; 0.45]	41.1%	41.1%
**MonthsAfterTRT:Within 6 Mo.**										
Yong−Wei Fu 2015	55	2.19	0.7200	55	2.29	0.5900	−0.15	[−0.53; 0.22]	41.0%	41.0%
**Common effect model**	**134**			**134**			**−0.05**	**[−0.29; 0.19]**	**100.0%**	**.**
**Random effects model**							**−0.05**	**[−0.29; 0.19]**	**.**	**100.0%**

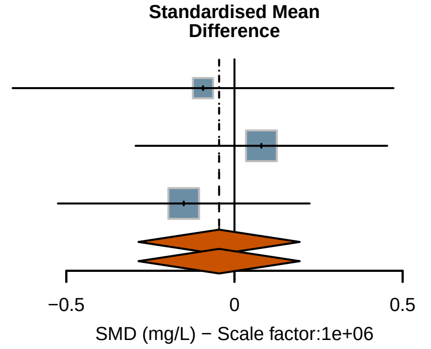

Heterogeneity: $I^2 = 0\%$, $\tau^2 = 0$, $p = 0.68$
Test for subgroup differences (common effect): $\chi^2_2 = 0.76$, df = 2 ($p = 0.68$)
Test for subgroup differences (random effects): $\chi^2_2 = 0.76$, df = 2 ($p = 0.68$)

SMD: −0.05; 95%C.I.[−0.29; 0.19] P value for common effect= 0.7083

SMD: −0.05; 95%C.I.[−0.29; 0.19] P value for random effect= 0.7083

Cytokine: IL−1beta − Treatment: Standard

Study	After TRT Total	After TRT Mean	After TRT SD	Before TRT Total	Before TRT Mean	Before TRT SD	SMD	95%−CI	Weight (common)	Weight (random)
**Assay type:ELISA**										
Ide M 2003	24	0.89	0.5900	24	0.95	0.6700	−0.09	[−0.66; 0.47]	17.9%	17.9%
Yong−Wei Fu 2015	55	2.34	0.6500	55	2.29	0.5900	0.08	[−0.29; 0.45]	41.1%	41.1%
Yong−Wei Fu 2015	55	2.19	0.7200	55	2.29	0.5900	−0.15	[−0.53; 0.22]	41.0%	41.0%
**Common effect model**	**134**			**134**			**−0.05**	**[−0.29; 0.19]**	**100.0%**	**.**
**Random effects model**							**−0.05**	**[−0.29; 0.19]**	**.**	**100.0%**
Heterogeneity: $I^2 = 0\%$, $\tau^2 = 0$, $p = 0.68$										
**Common effect model**	**134**			**134**			**−0.05**	**[−0.29; 0.19]**	**100.0%**	**.**
**Random effects model**							**−0.05**	**[−0.29; 0.19]**	**.**	**100.0%**

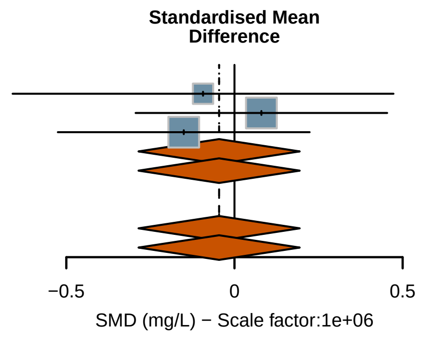

Heterogeneity: $I^2 = 0\%$, $\tau^2 = 0$, $p = 0.68$
Test for subgroup differences (common effect): $\chi^2_0 = 0.00$, df = 0 (p = NA)
Test for subgroup differences (random effects): $\chi^2_0 = 0.00$, df = 0 (p = NA)

SMD: −0.05; 95%C.I.[−0.29; 0.19] P value for common effect= 0.7083

SMD: −0.05; 95%C.I.[−0.29; 0.19] P value for random effect= 0.7083

Cytokine: IL−1beta − Treatment: Standard

Study	After TRT Total	After TRT Mean	After TRT SD	Before TRT Total	Before TRT Mean	Before TRT SD	SMD	95%−CI	Weight (common)	Weight (random)
**Bias judgment:Low risk**										
Ide M 2003	24	0.89	0.5900	24	0.95	0.6700	−0.09	[−0.66; 0.47]	17.9%	17.9%
Yong−Wei Fu 2015	55	2.34	0.6500	55	2.29	0.5900	0.08	[−0.29; 0.45]	41.1%	41.1%
Yong−Wei Fu 2015	55	2.19	0.7200	55	2.29	0.5900	−0.15	[−0.53; 0.22]	41.0%	41.0%
**Common effect model**	**134**			**134**			**−0.05**	**[−0.29; 0.19]**	**100.0%**	**.**
**Random effects model**							**−0.05**	**[−0.29; 0.19]**	**.**	**100.0%**
Heterogeneity: $I^2 = 0\%$, $\tau^2 = 0$, $p = 0.68$										
**Common effect model**	**134**			**134**			**−0.05**	**[−0.29; 0.19]**	**100.0%**	**.**
**Random effects model**							**−0.05**	**[−0.29; 0.19]**	**.**	**100.0%**

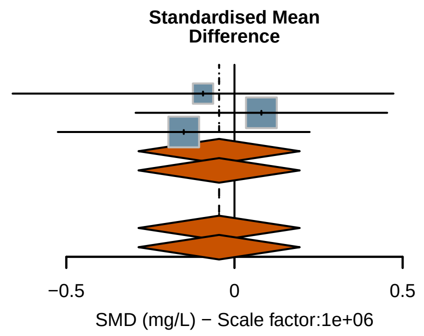

Heterogeneity: $I^2 = 0\%$, $\tau^2 = 0$, $p = 0.68$
Test for subgroup differences (common effect): $\chi^2_0 = 0.00$, df = 0 (p = NA)
Test for subgroup differences (random effects): $\chi^2_0 = 0.00$, df = 0 (p = NA)

SMD: −0.05; 95%C.I.[−0.29; 0.19] P value for common effect= 0.7083

SMD: −0.05; 95%C.I.[−0.29; 0.19] P value for random effect= 0.7083

Cytokine: IL−1beta − Treatment: Standard

Study	After TRT Total	After TRT Mean	After TRT SD	Before TRT Total	Before TRT Mean	Before TRT SD	SMD	95%−CI	Weight (common)	Weight (random)
**Study design:Prospective Interventional cohort study**										
Ide M 2003	24	0.89	0.5900	24	0.95	0.6700	−0.09	[−0.66; 0.47]	17.9%	17.9%
**Study design:RCT**										
Yong−Wei Fu 2015	55	2.34	0.6500	55	2.29	0.5900	0.08	[−0.29; 0.45]	41.1%	41.1%
Yong−Wei Fu 2015	55	2.19	0.7200	55	2.29	0.5900	−0.15	[−0.53; 0.22]	41.0%	41.0%
**Common effect model**	**110**			**110**			**−0.04**	**[−0.30; 0.23]**	**82.1%**	**.**
**Random effects model**							**−0.04**	**[−0.30; 0.23]**	**.**	**82.1%**
Heterogeneity: $I^2 = 0\%$, $\tau^2 = 0$, $p = 0.39$										
**Common effect model**	**134**			**134**			**−0.05**	**[−0.29; 0.19]**	**100.0%**	**.**
**Random effects model**							**−0.05**	**[−0.29; 0.19]**	**.**	**100.0%**

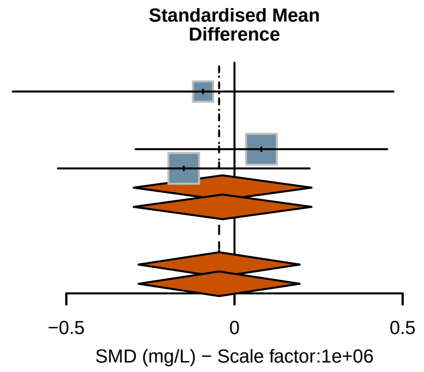

Heterogeneity: $I^2 = 0\%$, $\tau^2 = 0$, $p = 0.68$
Test for subgroup differences (common effect): $\chi^2_1 = 0.03$, df = 1 ($p = 0.86$)
Test for subgroup differences (random effects): $\chi^2_1 = 0.03$, df = 1 ($p = 0.86$)

SMD: −0.05; 95%C.I.[−0.29; 0.19] P value for common effect= 0.7083

SMD: −0.05; 95%C.I.[−0.29; 0.19] P value for random effect= 0.7083

Cytokine: IL−1beta − Treatment: Standard

Study	After TRT Total	After TRT Mean	After TRT SD	Before TRT Total	Before TRT Mean	Before TRT SD	SMD	95%−CI	Weight (common)	Weight (random)
**Additional_devices:Sex mixed**										
Ide M 2003	24	0.89	0.5900	24	0.95	0.6700	−0.09	[−0.66; 0.47]	17.9%	17.9%
Yong−Wei Fu 2015	55	2.34	0.6500	55	2.29	0.5900	0.08	[−0.29; 0.45]	41.1%	41.1%
Yong−Wei Fu 2015	55	2.19	0.7200	55	2.29	0.5900	−0.15	[−0.53; 0.22]	41.0%	41.0%
**Common effect model**	**134**			**134**			**−0.05**	**[−0.29; 0.19]**	**100.0%**	**.**
**Random effects model**							**−0.05**	**[−0.29; 0.19]**	**.**	**100.0%**
Heterogeneity: $I^2 = 0\%$, $\tau^2 = 0$, $p = 0.68$										
**Common effect model**	**134**			**134**			**−0.05**	**[−0.29; 0.19]**	**100.0%**	**.**
**Random effects model**							**−0.05**	**[−0.29; 0.19]**	**.**	**100.0%**

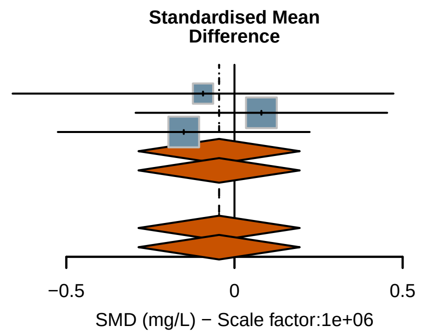

Heterogeneity: $I^2 = 0\%$, $\tau^2 = 0$, $p = 0.68$
Test for subgroup differences (common effect): $\chi^2_0 = 0.00$, df = 0 (p = NA)
Test for subgroup differences (random effects): $\chi^2_0 = 0.00$, df = 0 (p = NA)

SMD: −0.05; 95%C.I.[−0.29; 0.19] P value for common effect= 0.7083

SMD: −0.05; 95%C.I.[−0.29; 0.19] P value for random effect= 0.7083

Cytokine: IL−1beta − Treatment: Standard

Study	After TRT Total	After TRT Mean	After TRT SD	Before TRT Total	Before TRT Mean	Before TRT SD	SMD	95%−CI	Weight (common)	Weight (random)
**Female_binary:Sample with smokers**										
Yong−Wei Fu 2015	55	2.34	0.6500	55	2.29	0.5900	0.08	[−0.29; 0.45]	41.1%	41.1%
Yong−Wei Fu 2015	55	2.19	0.7200	55	2.29	0.5900	−0.15	[−0.53; 0.22]	41.0%	41.0%
**Common effect model**	**110**			**110**			**−0.04**	**[−0.30; 0.23]**	**82.1%**	**.**
**Random effects model**							**−0.04**	**[−0.30; 0.23]**	**.**	**82.1%**
Heterogeneity: $I^2 = 0\%$, $\tau^2 = 0$, $p = 0.39$										
**Female_binary:Sample without smokers**										
Ide M 2003	24	0.89	0.5900	24	0.95	0.6700	−0.09	[−0.66; 0.47]	17.9%	17.9%
**Common effect model**	**134**			**134**			**−0.05**	**[−0.29; 0.19]**	**100.0%**	**.**
**Random effects model**							**−0.05**	**[−0.29; 0.19]**	**.**	**100.0%**

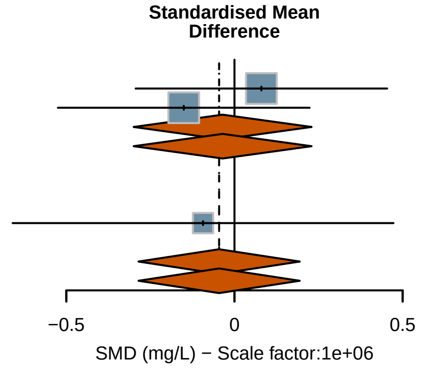

Heterogeneity: $I^2 = 0\%$, $\tau^2 = 0$, $p = 0.68$
Test for subgroup differences (common effect): $\chi^2_1 = 0.03$, df = 1 ($p = 0.86$)
Test for subgroup differences (random effects): $\chi^2_1 = 0.03$, df = 1 ($p = 0.86$)

SMD: −0.05; 95%C.I.[−0.29; 0.19] P value for common effect= 0.7083

SMD: −0.05; 95%C.I.[−0.29; 0.19] P value for random effect= 0.7083

Cytokine: IL−1beta − Treatment: Standard

Study	After TRT Total	After TRT Mean	After TRT SD	Before TRT Total	Before TRT Mean	Before TRT SD	SMD	95%−CI	Weight (common)	Weight (random)
**Smoke_binary:Sample without diabetic**										
Ide M 2003	24	0.89	0.5900	24	0.95	0.6700	−0.09	[−0.66; 0.47]	17.9%	17.9%
Yong−Wei Fu 2015	55	2.34	0.6500	55	2.29	0.5900	0.08	[−0.29; 0.45]	41.1%	41.1%
Yong−Wei Fu 2015	55	2.19	0.7200	55	2.29	0.5900	−0.15	[−0.53; 0.22]	41.0%	41.0%
**Common effect model**	**134**			**134**			**−0.05**	**[−0.29; 0.19]**	**100.0%**	**.**
**Random effects model**							**−0.05**	**[−0.29; 0.19]**	**.**	**100.0%**
Heterogeneity: $I^2 = 0\%$, $\tau^2 = 0$, $p = 0.68$										
**Common effect model**	**134**			**134**			**−0.05**	**[−0.29; 0.19]**	**100.0%**	**.**
**Random effects model**							**−0.05**	**[−0.29; 0.19]**	**.**	**100.0%**

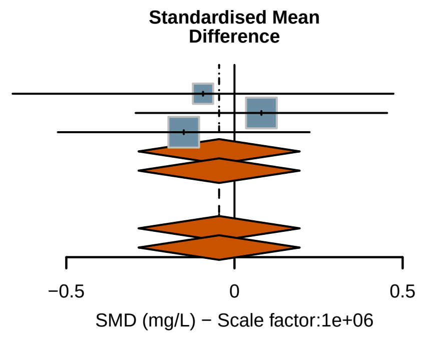

Heterogeneity: $I^2 = 0\%$, $\tau^2 = 0$, $p = 0.68$
Test for subgroup differences (common effect): $\chi^2_0 = 0.00$, df = 0 (p = NA)
Test for subgroup differences (random effects): $\chi^2_0 = 0.00$, df = 0 (p = NA)

SMD: −0.05; 95%C.I.[−0.29; 0.19] P value for common effect= 0.7083

SMD: −0.05; 95%C.I.[−0.29; 0.19] P value for random effect= 0.7083

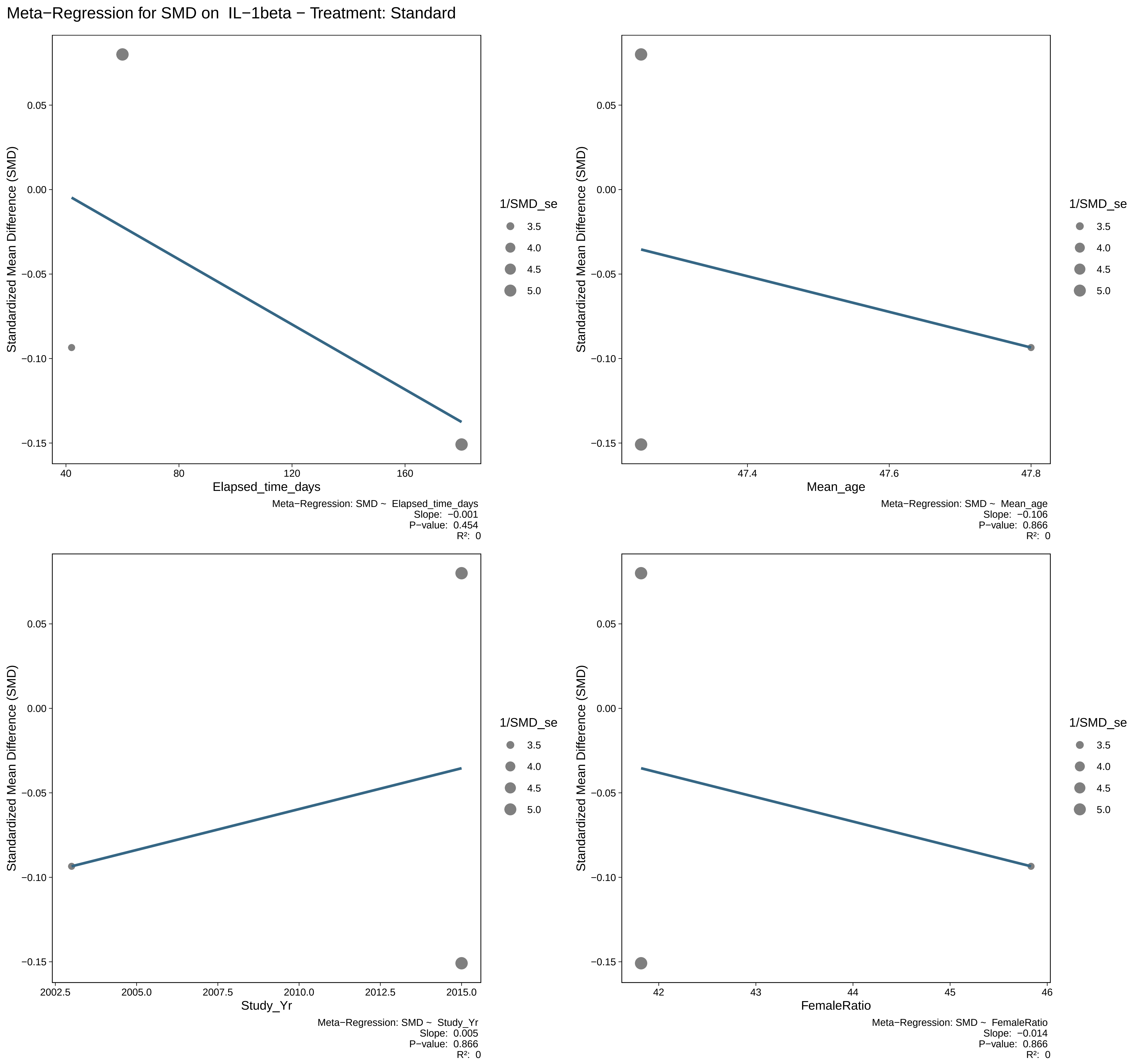
Meta−Regression for SMD on IL−1beta − Treatment: Standard
Standardized Mean Difference (SMD)
Elapsed_time_days
Meta−Regression: SMD ~ Elapsed_time_days
Slope: −0.001
P−value: 0.454
R²: 0
Mean_age
Meta−Regression: SMD ~ Mean_age
Slope: −0.106
P−value: 0.866
R²: 0
Study_Yr
Meta−Regression: SMD ~ Study_Yr
Slope: 0.005
P−value: 0.866
R²: 0
FemaleRatio
Meta−Regression: SMD ~ FemaleRatio
Slope: −0.014
P−value: 0.866
R²: 0
1/SMD_se
3.5
4.0
4.5
5.0
