# Supplementary material for: Temporal dynamics of early inflammatory markers after professional dental cleaning: a meta-analysis and spline-based meta-regression of TNF-α, IL-1β, IL-6, and (hs)CRP
Source: Front Immunol. 2025 Aug 28;16:1634622. doi: 10.3389/fimmu.2025.1634622 (PMC12423065; doi:10.3389/fimmu.2025.1634622)

Cytokine: IL−6 − Treatment: Intensive

Study	After TRT Total	After TRT Mean	After TRT SD	Before TRT Total	Before TRT Mean	Before TRT SD	SMD	95%−CI	Weight (common)	Weight (random)
**Days after treatment:21**										
Fiorini T 2012	27	5.50	2.3000	27	4.40	1.8000	0.52	[−0.02; 1.07]	2.1%	2.6%
**Days after treatment:30**										
Tonetti 2018	61	3.90	1.1000	61	2.40	5.4000	0.38	[0.02; 0.74]	4.9%	3.0%
de Sousa Rabelo 2020	15	7.83	17.3000	15	5.25	8.9900	0.18	[−0.54; 0.90]	1.2%	2.3%
de Sousa Rabelo 2020	15	3.62	8.1300	15	0.77	0.6220	0.48	[−0.25; 1.21]	1.2%	2.3%
de Sousa Rabelo 2020	15	2.87	5.7740	15	1.54	2.3900	0.29	[−0.43; 1.01]	1.2%	2.3%
**Common effect model**	**106**			**106**			**0.35**	**[0.08; 0.63]**	**8.4%**	**.**
**Random effects model**							**0.35**	**[0.08; 0.63]**	**.**	**9.8%**
Heterogeneity: $I^2 = 0\%$, $\tau^2 = 0$, $p = 0.94$										
**Days after treatment:35**										
Escobar Arregoces 2021	19	0.24	0.1300	19	0.31	0.1800	−0.44	[−1.08; 0.21]	1.5%	2.4%
**Days after treatment:42**										
Steven Offenbacher 2006	28	1.49	1.0500	28	0.47	0.2300	1.32	[0.74; 1.91]	1.8%	2.6%
Fang F 2015	48	4.72	2.1600	48	5.47	2.5700	−0.31	[−0.72; 0.09]	3.8%	2.9%
Nikolaos Markou 2023	20	6.40	8.2700	20	4.78	5.2360	0.23	[−0.39; 0.85]	1.6%	2.5%
Nikolaos Markou 2023	20	4.28	6.5670	20	4.99	7.9370	−0.10	[−0.72; 0.53]	1.6%	2.5%
Nikolaos Markou 2023	20	1.33	1.9540	20	1.54	2.2440	−0.10	[−0.72; 0.52]	1.6%	2.5%
**Common effect model**	**136**			**136**			**0.12**	**[−0.12; 0.37]**	**10.5%**	**.**
**Random effects model**							**0.20**	**[−0.38; 0.78]**	**.**	**12.9%**
Heterogeneity: $I^2 = 82\%$, $\tau^2 = 0.3485$, $p < 0.01$										
**Days after treatment:60**										
Tonetti 2018	61	2.50	0.7000	61	2.40	5.4000	0.03	[−0.33; 0.38]	4.9%	3.0%
Yong−Wei Fu 2015	54	1.18	0.4400	54	1.96	0.4900	−1.66	[−2.10; −1.22]	3.2%	2.8%
**Common effect model**	**115**			**115**			**−0.64**	**[−0.92; −0.36]**	**8.2%**	**.**
**Random effects model**							**−0.81**	**[−2.47; 0.84]**	**.**	**5.8%**
Heterogeneity: $I^2 = 97\%$, $\tau^2 = 1.3847$, $p < 0.01$										
**Days after treatment:90**										
Shih−Jung Lin 2011	14	1.53	0.7200	14	1.52	0.5100	0.02	[−0.72; 0.76]	1.1%	2.2%
Eduardo Machado Vilela 2011	36	4.08	3.0000	36	6.18	5.3000	−0.48	[−0.95; −0.01]	2.8%	2.8%
Eduardo Machado Vilela 2011	20	2.09	1.4000	20	2.95	2.2000	−0.46	[−1.09; 0.17]	1.6%	2.5%
Umut Altay 2013	22	0.77	0.8700	22	1.27	0.8700	−0.56	[−1.17; 0.04]	1.7%	2.5%
Umut Altay 2013	24	0.77	0.7900	24	1.10	0.3900	−0.52	[−1.10; 0.06]	1.9%	2.6%
Fang F 2015	48	4.42	1.9300	48	5.47	2.5700	−0.46	[−0.86; −0.05]	3.8%	2.9%
Nikolaos Markou 2023	20	4.16	5.2400	20	4.78	5.2360	−0.12	[−0.74; 0.50]	1.6%	2.5%
Nikolaos Markou 2023	20	6.32	9.2260	20	4.99	7.9370	0.15	[−0.47; 0.77]	1.6%	2.5%
Nikolaos Markou 2023	20	1.70	2.5760	20	1.54	2.2440	0.07	[−0.55; 0.69]	1.6%	2.5%
Filippo Graziani 2022	20	4.52	7.7500	20	3.60	3.2800	0.15	[−0.47; 0.77]	1.6%	2.5%
Filippo Graziani 2022	20	4.38	4.3000	20	5.21	4.7400	−0.18	[−0.80; 0.44]	1.6%	2.5%
Lobao 2018	33	3.94	3.3300	33	15.97	8.5800	−1.83	[−2.41; −1.25]	1.9%	2.6%
Wang 2017	19	13.35	8.4700	19	12828.40	13890.0000	−1.28	[−1.98; −0.57]	1.3%	2.3%
Kapellas 2016	24	3.70	2.1000	24	3.30	2.8900	0.16	[−0.41; 0.72]	1.9%	2.6%
**Common effect model**	**340**			**340**			**−0.40**	**[−0.55; −0.24]**	**26.1%**	**.**
**Random effects model**							**−0.38**	**[−0.68; −0.09]**	**.**	**35.3%**
Heterogeneity: $I^2 = 71\%$, $\tau^2 = 0.2271$, $p < 0.01$										
**Days after treatment:180**										
Tonetti 2018	61	2.50	0.8000	61	2.40	5.4000	0.03	[−0.33; 0.38]	4.9%	3.0%
Shih−Jung Lin 2011	14	1.74	0.5800	14	1.52	0.5100	0.39	[−0.36; 1.14]	1.1%	2.2%
Fang F 2015	48	4.30	1.8700	48	5.47	2.5700	−0.52	[−0.92; −0.11]	3.8%	2.9%
Hilana Paula Carillo ARTESE 2015	12	10900.00	2700.0000	12	13000.00	4400.0000	−0.56	[−1.37; 0.26]	0.9%	2.1%
Nikolaos Markou 2023	20	4.07	3.9100	20	4.78	5.2360	−0.15	[−0.77; 0.47]	1.6%	2.5%
Nikolaos Markou 2023	20	5.12	7.2700	20	4.99	7.9370	0.02	[−0.60; 0.64]	1.6%	2.5%
Nikolaos Markou 2023	20	1.37	1.6210	20	1.54	2.2440	−0.08	[−0.70; 0.54]	1.6%	2.5%
Geisinger 2016	240	2.64	2.3300	240	2.67	2.9300	−0.01	[−0.19; 0.17]	19.5%	3.2%
Yong−Wei Fu 2015	54	1.24	0.3900	54	1.96	0.4900	−1.61	[−2.05; −1.18]	3.3%	2.8%
**Common effect model**	**489**			**489**			**−0.20**	**[−0.33; −0.07]**	**38.3%**	**.**
**Random effects model**							**−0.29**	**[−0.68; 0.10]**	**.**	**23.7%**
Heterogeneity: $I^2 = 85\%$, $\tau^2 = 0.2831$, $p < 0.01$										
**Days after treatment:360**										
Nikolaos Markou 2023	20	3.08	3.0800	20	4.78	5.2360	−0.39	[−1.01; 0.24]	1.6%	2.5%
Nikolaos Markou 2023	20	5.78	9.3090	20	4.99	7.9370	0.09	[−0.53; 0.71]	1.6%	2.5%
Nikolaos Markou 2023	20	1.29	1.6210	20	1.54	2.2440	−0.13	[−0.75; 0.50]	1.6%	2.5%
**Common effect model**	**60**			**60**			**−0.14**	**[−0.50; 0.22]**	**4.8%**	**.**
**Random effects model**							**−0.14**	**[−0.50; 0.22]**	**.**	**7.4%**
Heterogeneity: $I^2 = 0\%$, $\tau^2 = 0$, $p = 0.57$										
**Common effect model**	**1292**			**1292**			**−0.19**	**[−0.27; −0.11]**	**100.0%**	**.**
**Random effects model**							**−0.20**	**[−0.39; −0.00]**	**.**	**100.0%**

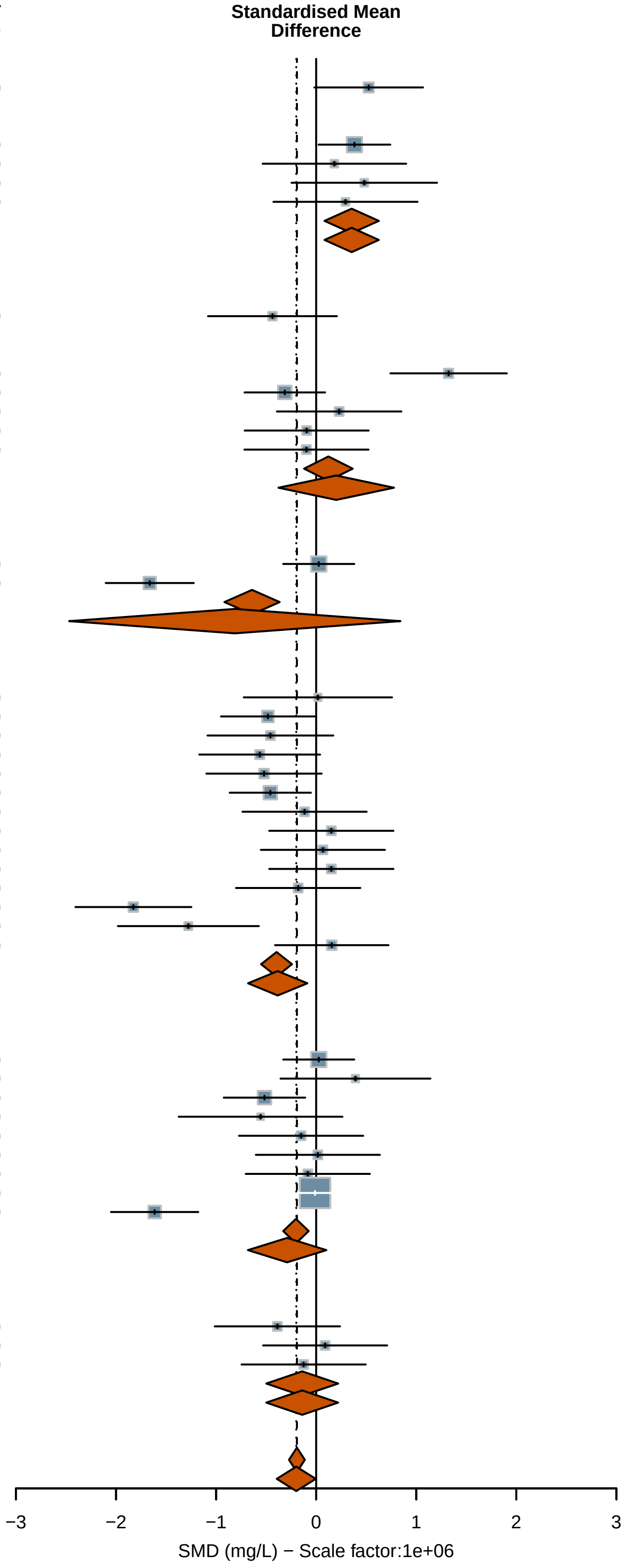

Heterogeneity: $I^2 = 81\%$, $\tau^2 = 0.2995$, $p < 0.01$
Test for subgroup differences (common effect): $\chi^2_7 = 46.15$, df = 7 ($p < 0.01$)
Test for subgroup differences (random effects): $\chi^2_7 = 22.26$, df = 7 ($p < 0.01$)

SMD: −0.19; 95%C.I.[−0.27; −0.11] P value for common effect= 0

SMD: −0.2; 95%C.I.[−0.39; 0] P value for random effect= 0.0456

Cytokine: IL−6 − Treatment: Intensive

Study	Total	Mean	After TRT SD	Total	Mean	Before TRT SD	SMD	95%−CI	Weight (common)	Weight (random)
**MonthsAfterTRT:4−6Wks**										
Steven Offenbacher 2006	28	1.49	1.0500	28	0.47	0.2300	1.32	[0.74; 1.91]	1.8%	2.6%
Tonetti 2018	61	3.90	1.1000	61	2.40	5.4000	0.38	[0.02; 0.74]	4.9%	3.0%
Fiorini T 2012	27	5.50	2.3000	27	4.40	1.8000	0.52	[−0.02; 1.07]	2.1%	2.6%
Fang F 2015	48	4.72	2.1600	48	5.47	2.5700	−0.31	[−0.72; 0.09]	3.8%	2.9%
Nikolaos Markou 2023	20	6.40	8.2700	20	4.78	5.2360	0.23	[−0.39; 0.85]	1.6%	2.5%
Nikolaos Markou 2023	20	4.28	6.5670	20	4.99	7.9370	−0.10	[−0.72; 0.53]	1.6%	2.5%
Nikolaos Markou 2023	20	1.33	1.9540	20	1.54	2.2440	−0.10	[−0.72; 0.52]	1.6%	2.5%
Escobar Arregoces 2021	19	0.24	0.1300	19	0.31	0.1800	−0.44	[−1.08; 0.21]	1.5%	2.4%
de Sousa Rabelo 2020	15	7.83	17.3000	15	5.25	8.9900	0.18	[−0.54; 0.90]	1.2%	2.3%
de Sousa Rabelo 2020	15	3.62	8.1300	15	0.77	0.6220	0.48	[−0.25; 1.21]	1.2%	2.3%
de Sousa Rabelo 2020	15	2.87	5.7740	15	1.54	2.3900	0.29	[−0.43; 1.01]	1.2%	2.3%
**Common effect model**	**288**			**288**			**0.21**	**[0.04; 0.38]**	**22.6%**	**.**
**Random effects model**							**0.22**	**[−0.07; 0.52]**	**.**	**27.8%**
Heterogeneity: $I^2 = 66\%$, $\tau^2 = 0.1551$, $p < 0.01$										
**MonthsAfterTRT:Within 3 Mo.**										
Tonetti 2018	61	2.50	0.7000	61	2.40	5.4000	0.03	[−0.33; 0.38]	4.9%	3.0%
Shih−Jung Lin 2011	14	1.53	0.7200	14	1.52	0.5100	0.02	[−0.72; 0.76]	1.1%	2.2%
Eduardo Machado Vilela 2011	36	4.08	3.0000	36	6.18	5.3000	−0.48	[−0.95; −0.01]	2.8%	2.8%
Eduardo Machado Vilela 2011	20	2.09	1.4000	20	2.95	2.2000	−0.46	[−1.09; 0.17]	1.6%	2.5%
Umut Altay 2013	22	0.77	0.8700	22	1.27	0.8700	−0.56	[−1.17; 0.04]	1.7%	2.5%
Umut Altay 2013	24	0.77	0.7900	24	1.10	0.3900	−0.52	[−1.10; 0.06]	1.9%	2.6%
Fang F 2015	48	4.42	1.9300	48	5.47	2.5700	−0.46	[−0.86; −0.05]	3.8%	2.9%
Nikolaos Markou 2023	20	4.16	5.2400	20	4.78	5.2360	−0.12	[−0.74; 0.50]	1.6%	2.5%
Nikolaos Markou 2023	20	6.32	9.2260	20	4.99	7.9370	0.15	[−0.47; 0.77]	1.6%	2.5%
Nikolaos Markou 2023	20	1.70	2.5760	20	1.54	2.2440	0.07	[−0.55; 0.69]	1.6%	2.5%
Filippo Graziani 2022	20	4.52	7.7500	20	3.60	3.2800	0.15	[−0.47; 0.77]	1.6%	2.5%
Filippo Graziani 2022	20	4.38	4.3000	20	5.21	4.7400	−0.18	[−0.80; 0.44]	1.6%	2.5%
Lobao 2018	33	3.94	3.3300	33	15.97	8.5800	−1.83	[−2.41; −1.25]	1.9%	2.6%
Wang 2017	19	13.35	8.4700	19	12828.40	13890.0000	−1.28	[−1.98; −0.57]	1.3%	2.3%
Kapellas 2016	24	3.70	2.1000	24	3.30	2.8900	0.16	[−0.41; 0.72]	1.9%	2.6%
Yong−Wei Fu 2015	54	1.18	0.4400	54	1.96	0.4900	−1.66	[−2.10; −1.22]	3.2%	2.8%
**Common effect model**	**455**			**455**			**−0.45**	**[−0.59; −0.32]**	**34.2%**	**.**
**Random effects model**							**−0.44**	**[−0.76; −0.13]**	**.**	**41.1%**
Heterogeneity: $I^2 = 81\%$, $\tau^2 = 0.3253$, $p < 0.01$										
**MonthsAfterTRT:Within 6 Mo.**										
Tonetti 2018	61	2.50	0.8000	61	2.40	5.4000	0.03	[−0.33; 0.38]	4.9%	3.0%
Shih−Jung Lin 2011	14	1.74	0.5800	14	1.52	0.5100	0.39	[−0.36; 1.14]	1.1%	2.2%
Fang F 2015	48	4.30	1.8700	48	5.47	2.5700	−0.52	[−0.92; −0.11]	3.8%	2.9%
Hilana Paula Carillo ARTESE 2015	12	10900.00	2700.0000	12	13000.00	4400.0000	−0.56	[−1.37; 0.26]	0.9%	2.1%
Nikolaos Markou 2023	20	4.07	3.9100	20	4.78	5.2360	−0.15	[−0.77; 0.47]	1.6%	2.5%
Nikolaos Markou 2023	20	5.12	7.2700	20	4.99	7.9370	0.02	[−0.60; 0.64]	1.6%	2.5%
Nikolaos Markou 2023	20	1.37	1.6210	20	1.54	2.2440	−0.08	[−0.70; 0.54]	1.6%	2.5%
Geisinger 2016	240	2.64	2.3300	240	2.67	2.9300	−0.01	[−0.19; 0.17]	19.5%	3.2%
Yong−Wei Fu 2015	54	1.24	0.3900	54	1.96	0.4900	−1.61	[−2.05; −1.18]	3.3%	2.8%
**Common effect model**	**489**			**489**			**−0.20**	**[−0.33; −0.07]**	**38.3%**	**.**
**Random effects model**							**−0.29**	**[−0.68; 0.10]**	**.**	**23.7%**
Heterogeneity: $I^2 = 85\%$, $\tau^2 = 0.2831$, $p < 0.01$										
**MonthsAfterTRT:6Mo. and more**										
Nikolaos Markou 2023	20	3.08	3.0800	20	4.78	5.2360	−0.39	[−1.01; 0.24]	1.6%	2.5%
Nikolaos Markou 2023	20	5.78	9.3090	20	4.99	7.9370	0.09	[−0.53; 0.71]	1.6%	2.5%
Nikolaos Markou 2023	20	1.29	1.6210	20	1.54	2.2440	−0.13	[−0.75; 0.50]	1.6%	2.5%
**Common effect model**	**60**			**60**			**−0.14**	**[−0.50; 0.22]**	**4.8%**	**.**
**Random effects model**							**−0.14**	**[−0.50; 0.22]**	**.**	**7.4%**
Heterogeneity: $I^2 = 0\%$, $\tau^2 = 0$, $p = 0.57$										
**Common effect model**	**1292**			**1292**			**−0.19**	**[−0.27; −0.11]**	**100.0%**	**.**
**Random effects model**							**−0.20**	**[−0.39; −0.00]**	**.**	**100.0%**

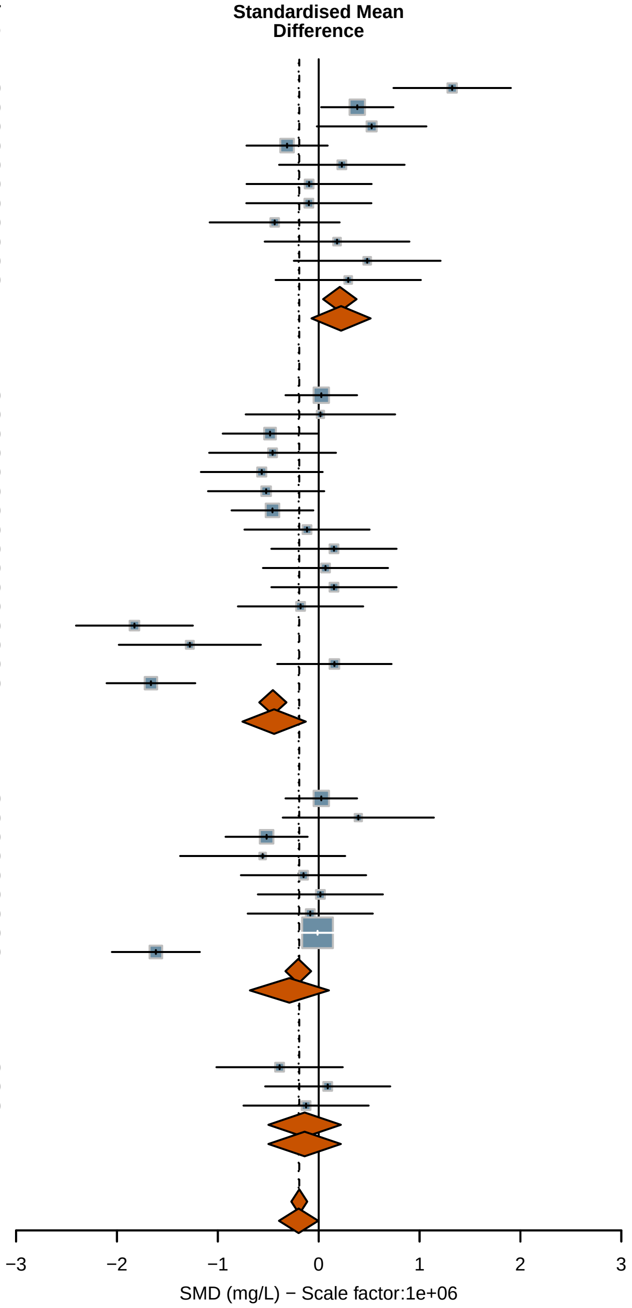

Heterogeneity: $I^2 = 81\%$, $\tau^2 = 0.2995$, $p < 0.01$
Test for subgroup differences (common effect): $\chi^2_3 = 37.10$, df = 3 ($p < 0.01$)
Test for subgroup differences (random effects): $\chi^2_3 = 9.95$, df = 3 ($p = 0.02$)

SMD: −0.19; 95%C.I.[−0.27; −0.11] P value for common effect= 0

SMD: −0.2; 95%C.I.[−0.39; 0] P value for random effect= 0.0456

Cytokine: IL−6 − Treatment: Intensive

Study	After TRT Total	After TRT Mean	After TRT SD	Before TRT Total	Before TRT Mean	Before TRT SD	SMD	95%−CI	Weight (common)	Weight (random)
**Assay type:ELISA**										
Steven Offenbacher 2006	28	1.49	1.0500	28	0.47	0.2300	1.32	[0.74; 1.91]	1.8%	2.6%
Tonetti 2018	61	3.90	1.1000	61	2.40	5.4000	0.38	[0.02; 0.74]	4.9%	3.0%
Tonetti 2018	61	2.50	0.7000	61	2.40	5.4000	0.03	[−0.33; 0.38]	4.9%	3.0%
Tonetti 2018	61	2.50	0.8000	61	2.40	5.4000	0.03	[−0.33; 0.38]	4.9%	3.0%
Shih−Jung Lin 2011	14	1.53	0.7200	14	1.52	0.5100	0.02	[−0.72; 0.76]	1.1%	2.2%
Shih−Jung Lin 2011	14	1.74	0.5800	14	1.52	0.5100	0.39	[−0.36; 1.14]	1.1%	2.2%
Umut Altay 2013	22	0.77	0.8700	22	1.27	0.8700	−0.56	[−1.17; 0.04]	1.7%	2.5%
Umut Altay 2013	24	0.77	0.7900	24	1.10	0.3900	−0.52	[−1.10; 0.06]	1.9%	2.6%
Hilana Paula Carillo ARTESE 2015	12	10900.00	2700.0000	12	13000.00	4400.0000	−0.56	[−1.37; 0.26]	0.9%	2.1%
Nikolaos Markou 2023	20	6.40	8.2700	20	4.78	5.2360	0.23	[−0.39; 0.85]	1.6%	2.5%
Nikolaos Markou 2023	20	4.16	5.2400	20	4.78	5.2360	−0.12	[−0.74; 0.50]	1.6%	2.5%
Nikolaos Markou 2023	20	4.07	3.9100	20	4.78	5.2360	−0.15	[−0.77; 0.47]	1.6%	2.5%
Nikolaos Markou 2023	20	3.08	3.0800	20	4.78	5.2360	−0.39	[−1.01; 0.24]	1.6%	2.5%
Nikolaos Markou 2023	20	4.28	6.5670	20	4.99	7.9370	−0.10	[−0.72; 0.53]	1.6%	2.5%
Nikolaos Markou 2023	20	6.32	9.2260	20	4.99	7.9370	0.15	[−0.47; 0.77]	1.6%	2.5%
Nikolaos Markou 2023	20	5.12	7.2700	20	4.99	7.9370	0.02	[−0.60; 0.64]	1.6%	2.5%
Nikolaos Markou 2023	20	5.78	9.3090	20	4.99	7.9370	0.09	[−0.53; 0.71]	1.6%	2.5%
Nikolaos Markou 2023	20	1.33	1.9540	20	1.54	2.2440	−0.10	[−0.72; 0.52]	1.6%	2.5%
Nikolaos Markou 2023	20	1.70	2.5760	20	1.54	2.2440	0.07	[−0.55; 0.69]	1.6%	2.5%
Nikolaos Markou 2023	20	1.37	1.6210	20	1.54	2.2440	−0.08	[−0.70; 0.54]	1.6%	2.5%
Nikolaos Markou 2023	20	1.29	1.6210	20	1.54	2.2440	−0.13	[−0.75; 0.50]	1.6%	2.5%
Filippo Graziani 2022	20	4.52	7.7500	20	3.60	3.2800	0.15	[−0.47; 0.77]	1.6%	2.5%
Filippo Graziani 2022	20	4.38	4.3000	20	5.21	4.7400	−0.18	[−0.80; 0.44]	1.6%	2.5%
Lobao 2018	33	3.94	3.3300	33	15.97	8.5800	−1.83	[−2.41; −1.25]	1.9%	2.6%
Wang 2017	19	13.35	8.4700	19	12828.40	13890.0000	−1.28	[−1.98; −0.57]	1.3%	2.3%
Kapellas 2016	24	3.70	2.1000	24	3.30	2.8900	0.16	[−0.41; 0.72]	1.9%	2.6%
Geisinger 2016	240	2.64	2.3300	240	2.67	2.9300	−0.01	[−0.19; 0.17]	19.5%	3.2%
Yong−Wei Fu 2015	54	1.18	0.4400	54	1.96	0.4900	−1.66	[−2.10; −1.22]	3.2%	2.8%
Yong−Wei Fu 2015	54	1.24	0.3900	54	1.96	0.4900	−1.61	[−2.05; −1.18]	3.3%	2.8%
**Common effect model**	**1001**			**1001**			**−0.18**	**[−0.27; −0.09]**	**77.0%**	**.**
**Random effects model**							**−0.22**	**[−0.47; 0.03]**	**.**	**74.2%**
Heterogeneity: $I^2 = 84\%$, $\tau^2 = 0.3742$, $p < 0.01$										
**Assay type:flow cytometry**										
Fiorini T 2012	27	5.50	2.3000	27	4.40	1.8000	0.52	[−0.02; 1.07]	2.1%	2.6%
**Assay type:immunosorbent assay**										
Fang F 2015	48	4.72	2.1600	48	5.47	2.5700	−0.31	[−0.72; 0.09]	3.8%	2.9%
Fang F 2015	48	4.42	1.9300	48	5.47	2.5700	−0.46	[−0.86; −0.05]	3.8%	2.9%
Fang F 2015	48	4.30	1.8700	48	5.47	2.5700	−0.52	[−0.92; −0.11]	3.8%	2.9%
**Common effect model**	**144**			**144**			**−0.43**	**[−0.66; −0.19]**	**11.4%**	**.**
**Random effects model**							**−0.43**	**[−0.66; −0.19]**	**.**	**8.7%**
Heterogeneity: $I^2 = 0\%$, $\tau^2 = 0$, $p = 0.77$										
**Assay type:latex−enhanced nephelometry**										
Eduardo Machado Vilela 2011	36	4.08	3.0000	36	6.18	5.3000	−0.48	[−0.95; −0.01]	2.8%	2.8%
Eduardo Machado Vilela 2011	20	2.09	1.4000	20	2.95	2.2000	−0.46	[−1.09; 0.17]	1.6%	2.5%
**Common effect model**	**56**			**56**			**−0.47**	**[−0.85; −0.10]**	**4.4%**	**.**
**Random effects model**							**−0.47**	**[−0.85; −0.10]**	**.**	**5.2%**
Heterogeneity: $I^2 = 0\%$, $\tau^2 = 0$, $p = 0.95$										
**Assay type:Milliplex Inflammation Human Cytokine kit**										
Escobar Arregoces 2021	19	0.24	0.1300	19	0.31	0.1800	−0.44	[−1.08; 0.21]	1.5%	2.4%
**Assay type:ultrasensitive multiplex assay**										
de Sousa Rabelo 2020	15	7.83	17.3000	15	5.25	8.9900	0.18	[−0.54; 0.90]	1.2%	2.3%
de Sousa Rabelo 2020	15	3.62	8.1300	15	0.77	0.6220	0.48	[−0.25; 1.21]	1.2%	2.3%
de Sousa Rabelo 2020	15	2.87	5.7740	15	1.54	2.3900	0.29	[−0.43; 1.01]	1.2%	2.3%
**Common effect model**	**45**			**45**			**0.32**	**[−0.10; 0.73]**	**3.6%**	**.**
**Random effects model**							**0.32**	**[−0.10; 0.73]**	**.**	**6.8%**
Heterogeneity: $I^2 = 0\%$, $\tau^2 = 0$, $p = 0.85$										
**Common effect model**	**1292**			**1292**			**−0.19**	**[−0.27; −0.11]**	**100.0%**	**.**
**Random effects model**							**−0.20**	**[−0.39; −0.00]**	**.**	**100.0%**

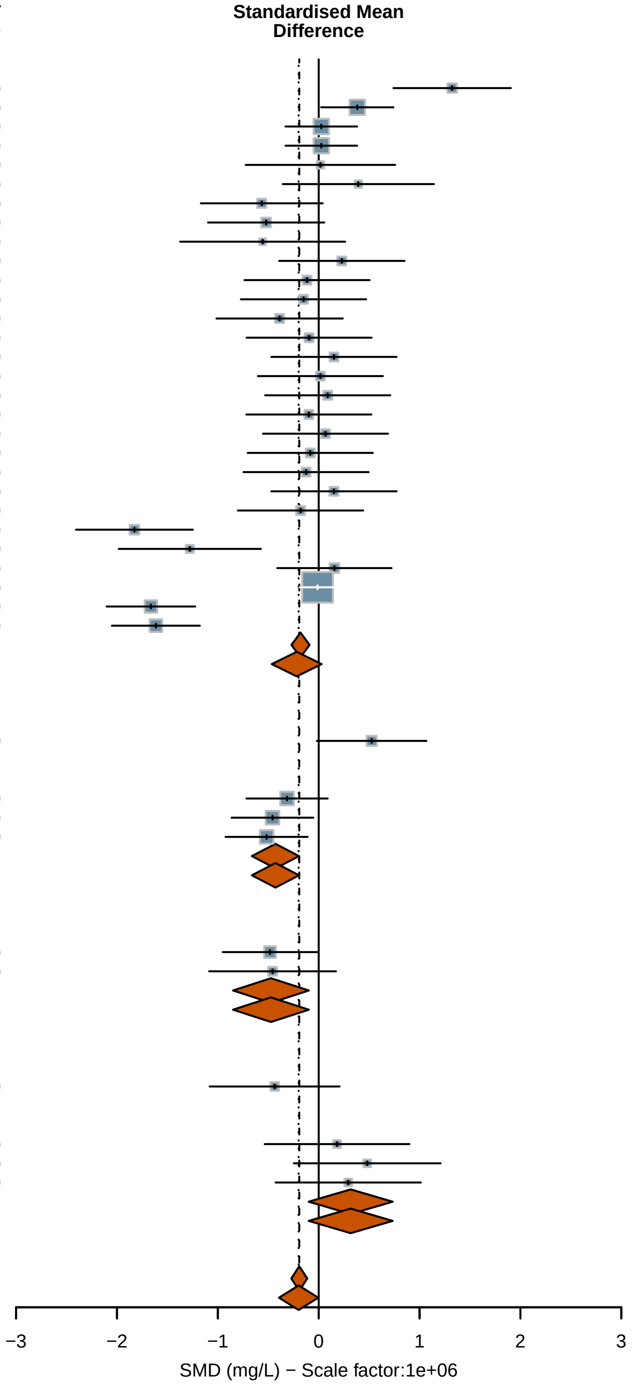

Heterogeneity: $I^2 = 81\%$, $\tau^2 = 0.2995$, $p < 0.01$
Test for subgroup differences (common effect): $\chi^2_5 = 19.14$, df = 5 ($p < 0.01$)
Test for subgroup differences (random effects): $\chi^2_5 = 18.83$, df = 5 ($p < 0.01$)

SMD: −0.19; 95%C.I.[−0.27; −0.11] P value for common effect= 0

SMD: −0.2; 95%C.I.[−0.39; 0] P value for random effect= 0.0456

Cytokine: IL−6 − Treatment: Intensive

Study	Total (After TRT)	Mean (After TRT)	SD (After TRT)	Total (Before TRT)	Mean (Before TRT)	SD (Before TRT)	SMD	95%−CI	Weight (common)	Weight (random)
**Bias judgment:High risk**										
Steven Offenbacher 2006	28	1.49	1.0500	28	0.47	0.2300	1.32	[0.74; 1.91]	1.8%	2.6%
Umut Altay 2013	22	0.77	0.8700	22	1.27	0.8700	−0.56	[−1.17; 0.04]	1.7%	2.5%
Umut Altay 2013	24	0.77	0.7900	24	1.10	0.3900	−0.52	[−1.10; 0.06]	1.9%	2.6%
Fiorini T 2012	27	5.50	2.3000	27	4.40	1.8000	0.52	[−0.02; 1.07]	2.1%	2.6%
Hilana Paula Carillo ARTESE 2015	12	10900.00	2700.0000	12	13000.00	4400.0000	−0.56	[−1.37; 0.26]	0.9%	2.1%
Nikolaos Markou 2023	20	6.40	8.2700	20	4.78	5.2360	0.23	[−0.39; 0.85]	1.6%	2.5%
Nikolaos Markou 2023	20	4.16	5.2400	20	4.78	5.2360	−0.12	[−0.74; 0.50]	1.6%	2.5%
Nikolaos Markou 2023	20	4.07	3.9100	20	4.78	5.2360	−0.15	[−0.77; 0.47]	1.6%	2.5%
Nikolaos Markou 2023	20	3.08	3.0800	20	4.78	5.2360	−0.39	[−1.01; 0.24]	1.6%	2.5%
Nikolaos Markou 2023	20	4.28	6.5670	20	4.99	7.9370	−0.10	[−0.72; 0.53]	1.6%	2.5%
Nikolaos Markou 2023	20	6.32	9.2260	20	4.99	7.9370	0.15	[−0.47; 0.77]	1.6%	2.5%
Nikolaos Markou 2023	20	5.12	7.2700	20	4.99	7.9370	0.02	[−0.60; 0.64]	1.6%	2.5%
Nikolaos Markou 2023	20	5.78	9.3090	20	4.99	7.9370	0.09	[−0.53; 0.71]	1.6%	2.5%
Nikolaos Markou 2023	20	1.33	1.9540	20	1.54	2.2440	−0.10	[−0.72; 0.52]	1.6%	2.5%
Nikolaos Markou 2023	20	1.70	2.5760	20	1.54	2.2440	0.07	[−0.55; 0.69]	1.6%	2.5%
Nikolaos Markou 2023	20	1.37	1.6210	20	1.54	2.2440	−0.08	[−0.70; 0.54]	1.6%	2.5%
Nikolaos Markou 2023	20	1.29	1.6210	20	1.54	2.2440	−0.13	[−0.75; 0.50]	1.6%	2.5%
Escobar Arregoces 2021	19	0.24	0.1300	19	0.31	0.1800	−0.44	[−1.08; 0.21]	1.5%	2.4%
de Sousa Rabelo 2020	15	7.83	17.3000	15	5.25	8.9900	0.18	[−0.54; 0.90]	1.2%	2.3%
de Sousa Rabelo 2020	15	3.62	8.1300	15	0.77	0.6220	0.48	[−0.25; 1.21]	1.2%	2.3%
de Sousa Rabelo 2020	15	2.87	5.7740	15	1.54	2.3900	0.29	[−0.43; 1.01]	1.2%	2.3%
Lobao 2018	33	3.94	3.3300	33	15.97	8.5800	−1.83	[−2.41; −1.25]	1.9%	2.6%
Kapellas 2016	24	3.70	2.1000	24	3.30	2.8900	0.16	[−0.41; 0.72]	1.9%	2.6%
**Common effect model**	**474**			**474**			**−0.06**	**[−0.19; 0.07]**	**36.7%**	**.**
**Random effects model**							**−0.06**	**[−0.30; 0.18]**	**.**	**56.5%**
Heterogeneity: $I^2 = 71\%$, $\tau^2 = 0.2376$, $p < 0.01$										
**Bias judgment:Low risk**										
Tonetti 2018	61	3.90	1.1000	61	2.40	5.4000	0.38	[0.02; 0.74]	4.9%	3.0%
Tonetti 2018	61	2.50	0.7000	61	2.40	5.4000	0.03	[−0.33; 0.38]	4.9%	3.0%
Tonetti 2018	61	2.50	0.8000	61	2.40	5.4000	0.03	[−0.33; 0.38]	4.9%	3.0%
Shih−Jung Lin 2011	14	1.53	0.7200	14	1.52	0.5100	0.02	[−0.72; 0.76]	1.1%	2.2%
Shih−Jung Lin 2011	14	1.74	0.5800	14	1.52	0.5100	0.39	[−0.36; 1.14]	1.1%	2.2%
Eduardo Machado Vilela 2011	36	4.08	3.0000	36	6.18	5.3000	−0.48	[−0.95; −0.01]	2.8%	2.8%
Eduardo Machado Vilela 2011	20	2.09	1.4000	20	2.95	2.2000	−0.46	[−1.09; 0.17]	1.6%	2.5%
Fang F 2015	48	4.72	2.1600	48	5.47	2.5700	−0.31	[−0.72; 0.09]	3.8%	2.9%
Fang F 2015	48	4.42	1.9300	48	5.47	2.5700	−0.46	[−0.86; −0.05]	3.8%	2.9%
Fang F 2015	48	4.30	1.8700	48	5.47	2.5700	−0.52	[−0.92; −0.11]	3.8%	2.9%
Yong−Wei Fu 2015	54	1.18	0.4400	54	1.96	0.4900	−1.66	[−2.10; −1.22]	3.2%	2.8%
Yong−Wei Fu 2015	54	1.24	0.3900	54	1.96	0.4900	−1.61	[−2.05; −1.18]	3.3%	2.8%
**Common effect model**	**519**			**519**			**−0.38**	**[−0.51; −0.26]**	**39.3%**	**.**
**Random effects model**							**−0.40**	**[−0.78; −0.02]**	**.**	**33.0%**
Heterogeneity: $I^2 = 89\%$, $\tau^2 = 0.3888$, $p < 0.01$										
**Bias judgment:Some concerns**										
Filippo Graziani 2022	20	4.52	7.7500	20	3.60	3.2800	0.15	[−0.47; 0.77]	1.6%	2.5%
Filippo Graziani 2022	20	4.38	4.3000	20	5.21	4.7400	−0.18	[−0.80; 0.44]	1.6%	2.5%
Wang 2017	19	13.35	8.4700	19	12828.40	13890.0000	−1.28	[−1.98; −0.57]	1.3%	2.3%
Geisinger 2016	240	2.64	2.3300	240	2.67	2.9300	−0.01	[−0.19; 0.17]	19.5%	3.2%
**Common effect model**	**299**			**299**			**−0.08**	**[−0.24; 0.08]**	**24.0%**	**.**
**Random effects model**							**−0.29**	**[−0.87; 0.29]**	**.**	**10.5%**
Heterogeneity: $I^2 = 76\%$, $\tau^2 = 0.2733$, $p < 0.01$										
**Common effect model**	**1292**			**1292**			**−0.19**	**[−0.27; −0.11]**	**100.0%**	**.**
**Random effects model**							**−0.20**	**[−0.39; −0.00]**	**.**	**100.0%**

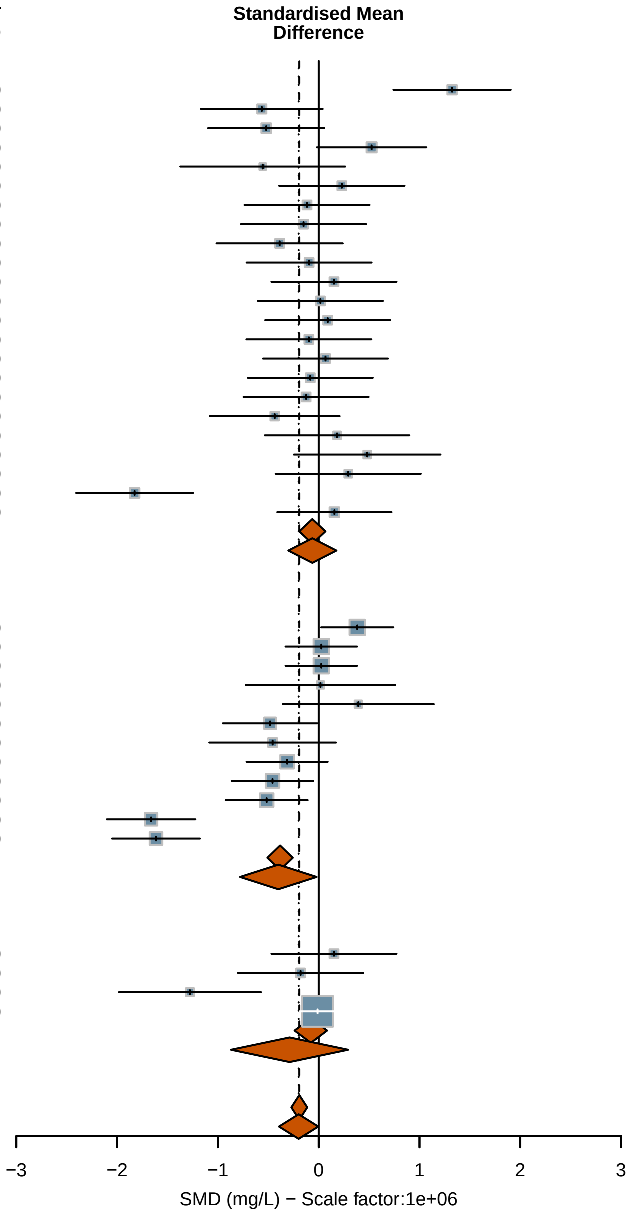

Heterogeneity: $I^2 = 81\%$, $\tau^2 = 0.2995$, $p < 0.01$
Test for subgroup differences (common effect): $\chi^2_2 = 14.51$, df = 2 ($p < 0.01$)
Test for subgroup differences (random effects): $\chi^2_2 = 2.35$, df = 2 ($p = 0.31$)

SMD: −0.19; 95%C.I.[−0.27; −0.11] P value for common effect= 0

SMD: −0.2; 95%C.I.[−0.39; 0] P value for random effect= 0.0456

Cytokine: IL−6 − Treatment: Intensive

Study	After TRT Total	After TRT Mean	After TRT SD	Before TRT Total	Before TRT Mean	Before TRT SD	SMD	95%−CI	Weight (common)	Weight (random)
**Study_design:Clinical trial**										
de Sousa Rabelo 2020	15	7.83	17.3000	15	5.25	8.9900	0.18	[−0.54; 0.90]	1.2%	2.3%
de Sousa Rabelo 2020	15	3.62	8.1300	15	0.77	0.6220	0.48	[−0.25; 1.21]	1.2%	2.3%
de Sousa Rabelo 2020	15	2.87	5.7740	15	1.54	2.3900	0.29	[−0.43; 1.01]	1.2%	2.3%
Lobao 2018	33	3.94	3.3300	33	15.97	8.5800	−1.83	[−2.41; −1.25]	1.9%	2.6%
**Common effect model**	**78**			**78**			**−0.41**	**[−0.75; −0.08]**	**5.4%**	**.**
**Random effects model**							**−0.23**	**[−1.31; 0.84]**	**.**	**9.4%**
Heterogeneity: $I^2 = 91\%$, $\tau^2 = 1.0886$, $p < 0.01$										
**Study_design:Comparative study**										
Umut Altay 2013	22	0.77	0.8700	22	1.27	0.8700	−0.56	[−1.17; 0.04]	1.7%	2.5%
Umut Altay 2013	24	0.77	0.7900	24	1.10	0.3900	−0.52	[−1.10; 0.06]	1.9%	2.6%
**Common effect model**	**46**			**46**			**−0.54**	**[−0.96; −0.12]**	**3.6%**	**.**
**Random effects model**							**−0.54**	**[−0.96; −0.12]**	**.**	**5.1%**
Heterogeneity: $I^2 = 0\%$, $\tau^2 = 0$, $p = 0.92$										
**Study_design:Interventional controlled non−RCT**										
Eduardo Machado Vilela 2011	36	4.08	3.0000	36	6.18	5.3000	−0.48	[−0.95; −0.01]	2.8%	2.8%
Eduardo Machado Vilela 2011	20	2.09	1.4000	20	2.95	2.2000	−0.46	[−1.09; 0.17]	1.6%	2.5%
**Common effect model**	**56**			**56**			**−0.47**	**[−0.85; −0.10]**	**4.4%**	**.**
**Random effects model**							**−0.47**	**[−0.85; −0.10]**	**.**	**5.2%**
Heterogeneity: $I^2 = 0\%$, $\tau^2 = 0$, $p = 0.95$										
**Study_design:Observational study**										
Escobar Arregoces 2021	19	0.24	0.1300	19	0.31	0.1800	−0.44	[−1.08; 0.21]	1.5%	2.4%
**Study_design:RCT**										
Steven Offenbacher 2006	28	1.49	1.0500	28	0.47	0.2300	1.32	[0.74; 1.91]	1.8%	2.6%
Tonetti 2018	61	3.90	1.1000	61	2.40	5.4000	0.38	[0.02; 0.74]	4.9%	3.0%
Tonetti 2018	61	2.50	0.7000	61	2.40	5.4000	0.03	[−0.33; 0.38]	4.9%	3.0%
Tonetti 2018	61	2.50	0.8000	61	2.40	5.4000	0.03	[−0.33; 0.38]	4.9%	3.0%
Shih−Jung Lin 2011	14	1.53	0.7200	14	1.52	0.5100	0.02	[−0.72; 0.76]	1.1%	2.2%
Shih−Jung Lin 2011	14	1.74	0.5800	14	1.52	0.5100	0.39	[−0.36; 1.14]	1.1%	2.2%
Fiorini T 2012	27	5.50	2.3000	27	4.40	1.8000	0.52	[−0.02; 1.07]	2.1%	2.6%
Fang F 2015	48	4.72	2.1600	48	5.47	2.5700	−0.31	[−0.72; 0.09]	3.8%	2.9%
Fang F 2015	48	4.42	1.9300	48	5.47	2.5700	−0.46	[−0.86; −0.05]	3.8%	2.9%
Fang F 2015	48	4.30	1.8700	48	5.47	2.5700	−0.52	[−0.92; −0.11]	3.8%	2.9%
Hilana Paula Carillo ARTESE 2015	12	10900.00	2700.0000	12	13000.00	4400.0000	−0.56	[−1.37; 0.26]	0.9%	2.1%
Nikolaos Markou 2023	20	6.40	8.2700	20	4.78	5.2360	0.23	[−0.39; 0.85]	1.6%	2.5%
Nikolaos Markou 2023	20	4.16	5.2400	20	4.78	5.2360	−0.12	[−0.74; 0.50]	1.6%	2.5%
Nikolaos Markou 2023	20	4.07	3.9100	20	4.78	5.2360	−0.15	[−0.77; 0.47]	1.6%	2.5%
Nikolaos Markou 2023	20	3.08	3.0800	20	4.78	5.2360	−0.39	[−1.01; 0.24]	1.6%	2.5%
Nikolaos Markou 2023	20	4.28	6.5670	20	4.99	7.9370	−0.10	[−0.72; 0.53]	1.6%	2.5%
Nikolaos Markou 2023	20	6.32	9.2260	20	4.99	7.9370	0.15	[−0.47; 0.77]	1.6%	2.5%
Nikolaos Markou 2023	20	5.12	7.2700	20	4.99	7.9370	0.02	[−0.60; 0.64]	1.6%	2.5%
Nikolaos Markou 2023	20	5.78	9.3090	20	4.99	7.9370	0.09	[−0.53; 0.71]	1.6%	2.5%
Nikolaos Markou 2023	20	1.33	1.9540	20	1.54	2.2440	−0.10	[−0.72; 0.52]	1.6%	2.5%
Nikolaos Markou 2023	20	1.70	2.5760	20	1.54	2.2440	0.07	[−0.55; 0.69]	1.6%	2.5%
Nikolaos Markou 2023	20	1.37	1.6210	20	1.54	2.2440	−0.08	[−0.70; 0.54]	1.6%	2.5%
Nikolaos Markou 2023	20	1.29	1.6210	20	1.54	2.2440	−0.13	[−0.75; 0.50]	1.6%	2.5%
Filippo Graziani 2022	20	4.52	7.7500	20	3.60	3.2800	0.15	[−0.47; 0.77]	1.6%	2.5%
Filippo Graziani 2022	20	4.38	4.3000	20	5.21	4.7400	−0.18	[−0.80; 0.44]	1.6%	2.5%
Wang 2017	19	13.35	8.4700	19	12828.40	13890.0000	−1.28	[−1.98; −0.57]	1.3%	2.3%
Kapellas 2016	24	3.70	2.1000	24	3.30	2.8900	0.16	[−0.41; 0.72]	1.9%	2.6%
Geisinger 2016	240	2.64	2.3300	240	2.67	2.9300	−0.01	[−0.19; 0.17]	19.5%	3.2%
Yong−Wei Fu 2015	54	1.18	0.4400	54	1.96	0.4900	−1.66	[−2.10; −1.22]	3.2%	2.8%
Yong−Wei Fu 2015	54	1.24	0.3900	54	1.96	0.4900	−1.61	[−2.05; −1.18]	3.3%	2.8%
**Common effect model**	**1093**			**1093**			**−0.14**	**[−0.23; −0.06]**	**85.1%**	**.**
**Random effects model**							**−0.14**	**[−0.36; 0.07]**	**.**	**77.9%**
Heterogeneity: $I^2 = 82\%$, $\tau^2 = 0.2843$, $p < 0.01$										
**Common effect model**	**1292**			**1292**			**−0.19**	**[−0.27; −0.11]**	**100.0%**	**.**
**Random effects model**							**−0.20**	**[−0.39; −0.00]**	**.**	**100.0%**

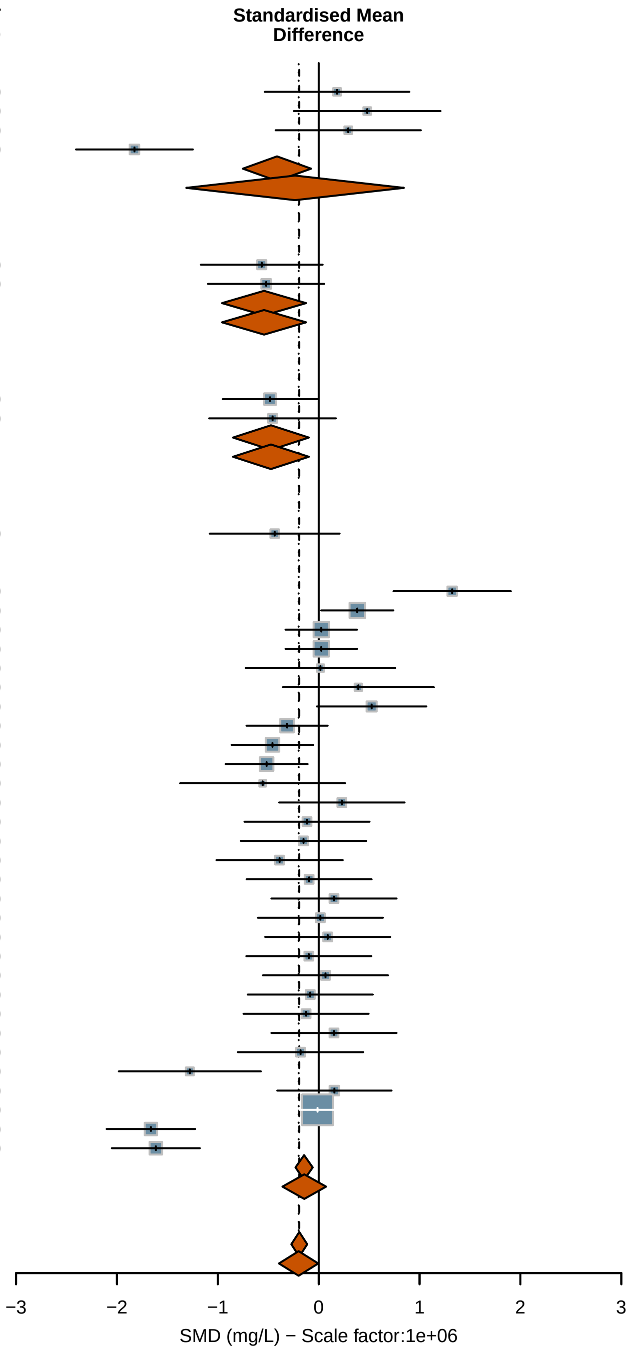

Heterogeneity: $I^2 = 81\%$, $\tau^2 = 0.2995$, $p < 0.01$
Test for subgroup differences (common effect): $\chi^2_4 = 8.23$, df = 4 ($p = 0.08$)
Test for subgroup differences (random effects): $\chi^2_4 = 4.29$, df = 4 ($p = 0.37$)

SMD: −0.19; 95%C.I.[−0.27; −0.11] P value for common effect= 0

SMD: −0.2; 95%C.I.[−0.39; 0] P value for random effect= 0.0456

Cytokine: IL−6 − Treatment: Intensive

Study	After TRT Total	After TRT Mean	After TRT SD	Before TRT Total	Before TRT Mean	Before TRT SD	SMD	95%−CI	Weight (common)	Weight (random)
**Additional_devices:FMD**										
Filippo Graziani 2022	20	4.52	7.7500	20	3.60	3.2800	0.15	[−0.47; 0.77]	7.1%	8.2%
**Additional_devices:laser 2 W, 200 mJ, 10Hz**										
Nikolaos Markou 2023	20	1.33	1.9540	20	1.54	2.2440	−0.10	[−0.72; 0.52]	7.1%	8.2%
Nikolaos Markou 2023	20	1.70	2.5760	20	1.54	2.2440	0.07	[−0.55; 0.69]	7.1%	8.2%
Nikolaos Markou 2023	20	1.37	1.6210	20	1.54	2.2440	−0.08	[−0.70; 0.54]	7.1%	8.2%
Nikolaos Markou 2023	20	1.29	1.6210	20	1.54	2.2440	−0.13	[−0.75; 0.50]	7.1%	8.2%
**Common effect model**	**80**			**80**			**−0.06**	**[−0.37; 0.25]**	**28.6%**	**.**
**Random effects model**							**−0.06**	**[−0.37; 0.25]**	**.**	**32.7%**
Heterogeneity: I^2 = 0%, τ^2 = 0, p = 0.97										
**Additional_devices:Laser 3 W, 150mJ, 20Hz**										
Nikolaos Markou 2023	20	4.28	6.5670	20	4.99	7.9370	−0.10	[−0.72; 0.53]	7.1%	8.2%
Nikolaos Markou 2023	20	6.32	9.2260	20	4.99	7.9370	0.15	[−0.47; 0.77]	7.1%	8.2%
Nikolaos Markou 2023	20	5.12	7.2700	20	4.99	7.9370	0.02	[−0.60; 0.64]	7.1%	8.2%
Nikolaos Markou 2023	20	5.78	9.3090	20	4.99	7.9370	0.09	[−0.53; 0.71]	7.1%	8.2%
**Common effect model**	**80**			**80**			**0.04**	**[−0.27; 0.35]**	**28.6%**	**.**
**Random effects model**							**0.04**	**[−0.27; 0.35]**	**.**	**32.7%**
Heterogeneity: I^2 = 0%, τ^2 = 0, p = 0.95										
**Additional_devices:Protocollo full mouth disinfection**										
Yong−Wei Fu 2015	54	1.18	0.4400	54	1.96	0.4900	−1.66	[−2.10; −1.22]	14.2%	9.1%
Yong−Wei Fu 2015	54	1.24	0.3900	54	1.96	0.4900	−1.61	[−2.05; −1.18]	14.4%	9.1%
**Common effect model**	**108**			**108**			**−1.64**	**[−1.95; −1.33]**	**28.6%**	**.**
**Random effects model**							**−1.64**	**[−1.95; −1.33]**	**.**	**18.2%**
Heterogeneity: I^2 = 0%, τ^2 = 0, p = 0.88										
**Additional_devices:QMD**										
Filippo Graziani 2022	20	4.38	4.3000	20	5.21	4.7400	−0.18	[−0.80; 0.44]	7.1%	8.2%
**Common effect model**	**308**			**308**			**−0.48**	**[−0.64; −0.31]**	**100.0%**	**.**
**Random effects model**							**−0.31**	**[−0.70; 0.08]**	**.**	**100.0%**

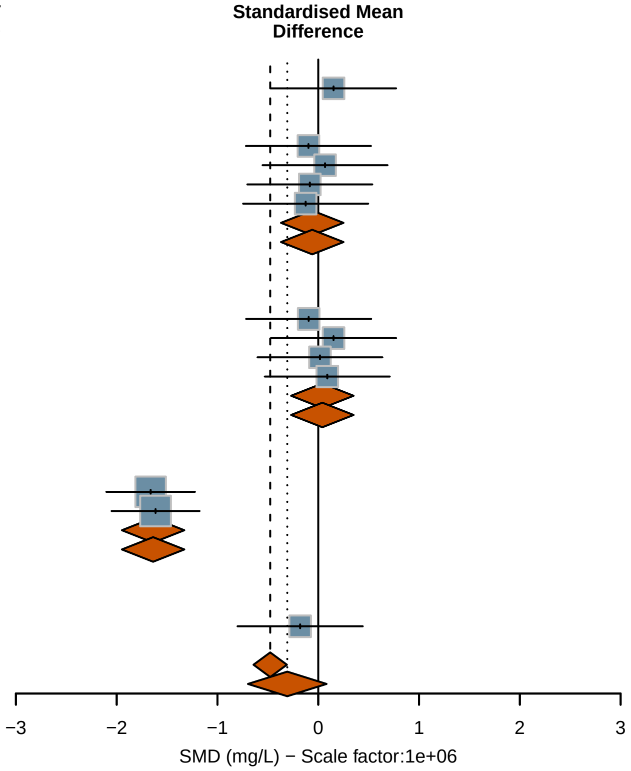

Heterogeneity: I^2 = 86%, τ^2 = 0.3840, p < 0.01
Test for subgroup differences (common effect): χ^2_4 = 76.53, df = 4 (p < 0.01)
Test for subgroup differences (random effects): χ^2_4 = 76.53, df = 4 (p < 0.01)

SMD: −0.48; 95%C.I.[−0.64; −0.31] P value for common effect= 0

SMD: −0.31; 95%C.I.[−0.7; 0.08] P value for random effect= 0.1222

Cytokine: IL−6 − Treatment: Intensive

Study	After TRT Total	After TRT Mean	After TRT SD	Before TRT Total	Before TRT Mean	Before TRT SD	SMD	95%−CI	Weight (common)	Weight (random)
**Female_binary:Only Female**										
Steven Offenbacher 2006	28	1.49	1.0500	28	0.47	0.2300	1.32	[0.74; 1.91]	1.8%	2.6%
Fiorini T 2012	27	5.50	2.3000	27	4.40	1.8000	0.52	[−0.02; 1.07]	2.1%	2.6%
**Common effect model**	**55**			**55**			**0.90**	**[0.50; 1.29]**	**4.0%**	**.**
**Random effects model**							**0.92**	**[0.13; 1.70]**	**.**	**5.2%**
Heterogeneity: $I^2 = 74\%$, $\tau^2 = 0.2362$, $p = 0.05$										
**Female_binary:Sex mixed**										
Tonetti 2018	61	3.90	1.1000	61	2.40	5.4000	0.38	[0.02; 0.74]	4.9%	3.0%
Tonetti 2018	61	2.50	0.7000	61	2.40	5.4000	0.03	[−0.33; 0.38]	4.9%	3.0%
Tonetti 2018	61	2.50	0.8000	61	2.40	5.4000	0.03	[−0.33; 0.38]	4.9%	3.0%
Shih−Jung Lin 2011	14	1.53	0.7200	14	1.52	0.5100	0.02	[−0.72; 0.76]	1.1%	2.2%
Shih−Jung Lin 2011	14	1.74	0.5800	14	1.52	0.5100	0.39	[−0.36; 1.14]	1.1%	2.2%
Eduardo Machado Vilela 2011	36	4.08	3.0000	36	6.18	5.3000	−0.48	[−0.95; −0.01]	2.8%	2.8%
Eduardo Machado Vilela 2011	20	2.09	1.4000	20	2.95	2.2000	−0.46	[−1.09; 0.17]	1.6%	2.5%
Umut Altay 2013	22	0.77	0.8700	22	1.27	0.8700	−0.56	[−1.17; 0.04]	1.7%	2.5%
Umut Altay 2013	24	0.77	0.7900	24	1.10	0.3900	−0.52	[−1.10; 0.06]	1.9%	2.6%
Fang F 2015	48	4.72	2.1600	48	5.47	2.5700	−0.31	[−0.72; 0.09]	3.8%	2.9%
Fang F 2015	48	4.42	1.9300	48	5.47	2.5700	−0.46	[−0.86; −0.05]	3.8%	2.9%
Fang F 2015	48	4.30	1.8700	48	5.47	2.5700	−0.52	[−0.92; −0.11]	3.8%	2.9%
Hilana Paula Carillo ARTESE 2015	12	10900.00	2700.0000	12	13000.00	4400.0000	−0.56	[−1.37; 0.26]	0.9%	2.1%
Nikolaos Markou 2023	20	6.40	8.2700	20	4.78	5.2360	0.23	[−0.39; 0.85]	1.6%	2.5%
Nikolaos Markou 2023	20	4.16	5.2400	20	4.78	5.2360	−0.12	[−0.74; 0.50]	1.6%	2.5%
Nikolaos Markou 2023	20	4.07	3.9100	20	4.78	5.2360	−0.15	[−0.77; 0.47]	1.6%	2.5%
Nikolaos Markou 2023	20	3.08	3.0800	20	4.78	5.2360	−0.39	[−1.01; 0.24]	1.6%	2.5%
Nikolaos Markou 2023	20	4.28	6.5670	20	4.99	7.9370	−0.10	[−0.72; 0.53]	1.6%	2.5%
Nikolaos Markou 2023	20	6.32	9.2260	20	4.99	7.9370	0.15	[−0.47; 0.77]	1.6%	2.5%
Nikolaos Markou 2023	20	5.12	7.2700	20	4.99	7.9370	0.02	[−0.60; 0.64]	1.6%	2.5%
Nikolaos Markou 2023	20	5.78	9.3090	20	4.99	7.9370	0.09	[−0.53; 0.71]	1.6%	2.5%
Nikolaos Markou 2023	20	1.33	1.9540	20	1.54	2.2440	−0.10	[−0.72; 0.52]	1.6%	2.5%
Nikolaos Markou 2023	20	1.70	2.5760	20	1.54	2.2440	0.07	[−0.55; 0.69]	1.6%	2.5%
Nikolaos Markou 2023	20	1.37	1.6210	20	1.54	2.2440	−0.08	[−0.70; 0.54]	1.6%	2.5%
Nikolaos Markou 2023	20	1.29	1.6210	20	1.54	2.2440	−0.13	[−0.75; 0.50]	1.6%	2.5%
Filippo Graziani 2022	20	4.52	7.7500	20	3.60	3.2800	0.15	[−0.47; 0.77]	1.6%	2.5%
Filippo Graziani 2022	20	4.38	4.3000	20	5.21	4.7400	−0.18	[−0.80; 0.44]	1.6%	2.5%
Escobar Arregoces 2021	19	0.24	0.1300	19	0.31	0.1800	−0.44	[−1.08; 0.21]	1.5%	2.4%
de Sousa Rabelo 2020	15	7.83	17.3000	15	5.25	8.9900	0.18	[−0.54; 0.90]	1.2%	2.3%
de Sousa Rabelo 2020	15	3.62	8.1300	15	0.77	0.6220	0.48	[−0.25; 1.21]	1.2%	2.3%
de Sousa Rabelo 2020	15	2.87	5.7740	15	1.54	2.3900	0.29	[−0.43; 1.01]	1.2%	2.3%
Lobao 2018	33	3.94	3.3300	33	15.97	8.5800	−1.83	[−2.41; −1.25]	1.9%	2.6%
Wang 2017	19	13.35	8.4700	19	12828.40	13890.0000	−1.28	[−1.98; −0.57]	1.3%	2.3%
Kapellas 2016	24	3.70	2.1000	24	3.30	2.8900	0.16	[−0.41; 0.72]	1.9%	2.6%
Geisinger 2016	240	2.64	2.3300	240	2.67	2.9300	−0.01	[−0.19; 0.17]	19.5%	3.2%
Yong−Wei Fu 2015	54	1.18	0.4400	54	1.96	0.4900	−1.66	[−2.10; −1.22]	3.2%	2.8%
Yong−Wei Fu 2015	54	1.24	0.3900	54	1.96	0.4900	−1.61	[−2.05; −1.18]	3.3%	2.8%
**Common effect model**	**1237**			**1237**			**−0.24**	**[−0.32; −0.16]**	**96.0%**	**.**
**Random effects model**							**−0.26**	**[−0.45; −0.08]**	**.**	**94.8%**
Heterogeneity: $I^2 = 78\%$, $\tau^2 = 0.2404$, $p < 0.01$										
**Common effect model**	**1292**			**1292**			**−0.19**	**[−0.27; −0.11]**	**100.0%**	**.**
**Random effects model**							**−0.20**	**[−0.39; −0.00]**	**.**	**100.0%**

Standardised Mean Difference

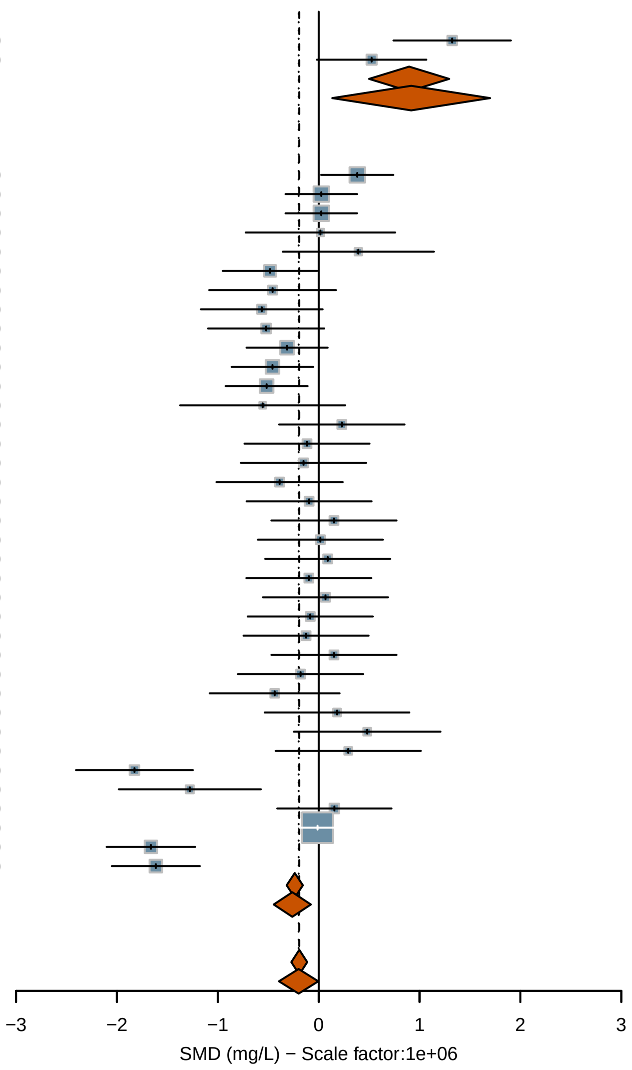

Heterogeneity: $I^2 = 81\%$, $\tau^2 = 0.2995$, $p < 0.01$
Test for subgroup differences (common effect): $\chi^2_1 = 30.08$, df = 1 ($p < 0.01$)
Test for subgroup differences (random effects): $\chi^2_1 = 8.26$, df = 1 ($p < 0.01$)

SMD: −0.19; 95%C.I.[−0.27; −0.11] P value for common effect= 0

SMD: −0.2; 95%C.I.[−0.39; 0] P value for random effect= 0.0456

Cytokine: IL−6 − Treatment: Intensive

Study	After TRT Total	After TRT Mean	After TRT SD	Before TRT Total	Before TRT Mean	Before TRT SD	SMD	95%−CI	Weight (common)	Weight (random)
**Smoke_binary:Sample with smokers**										
Tonetti 2018	61	3.90	1.1000	61	2.40	5.4000	0.38	[0.02; 0.74]	5.3%	3.3%
Tonetti 2018	61	2.50	0.7000	61	2.40	5.4000	0.03	[−0.33; 0.38]	5.4%	3.3%
Tonetti 2018	61	2.50	0.8000	61	2.40	5.4000	0.03	[−0.33; 0.38]	5.4%	3.3%
Umut Altay 2013	22	0.77	0.8700	22	1.27	0.8700	−0.56	[−1.17; 0.04]	1.9%	2.9%
Umut Altay 2013	24	0.77	0.7900	24	1.10	0.3900	−0.52	[−1.10; 0.06]	2.0%	2.9%
Fiorini T 2012	27	5.50	2.3000	27	4.40	1.8000	0.52	[−0.02; 1.07]	2.3%	3.0%
Fang F 2015	48	4.72	2.1600	48	5.47	2.5700	−0.31	[−0.72; 0.09]	4.2%	3.3%
Fang F 2015	48	4.42	1.9300	48	5.47	2.5700	−0.46	[−0.86; −0.05]	4.1%	3.3%
Fang F 2015	48	4.30	1.8700	48	5.47	2.5700	−0.52	[−0.92; −0.11]	4.1%	3.2%
Nikolaos Markou 2023	20	6.40	8.2700	20	4.78	5.2360	0.23	[−0.39; 0.85]	1.7%	2.8%
Nikolaos Markou 2023	20	4.16	5.2400	20	4.78	5.2360	−0.12	[−0.74; 0.50]	1.8%	2.8%
Nikolaos Markou 2023	20	4.07	3.9100	20	4.78	5.2360	−0.15	[−0.77; 0.47]	1.7%	2.8%
Nikolaos Markou 2023	20	3.08	3.0800	20	4.78	5.2360	−0.39	[−1.01; 0.24]	1.7%	2.8%
Nikolaos Markou 2023	20	4.28	6.5670	20	4.99	7.9370	−0.10	[−0.72; 0.53]	1.8%	2.8%
Nikolaos Markou 2023	20	6.32	9.2260	20	4.99	7.9370	0.15	[−0.47; 0.77]	1.7%	2.8%
Nikolaos Markou 2023	20	5.12	7.2700	20	4.99	7.9370	0.02	[−0.60; 0.64]	1.8%	2.8%
Nikolaos Markou 2023	20	5.78	9.3090	20	4.99	7.9370	0.09	[−0.53; 0.71]	1.8%	2.8%
Nikolaos Markou 2023	20	1.33	1.9540	20	1.54	2.2440	−0.10	[−0.72; 0.52]	1.8%	2.8%
Nikolaos Markou 2023	20	1.70	2.5760	20	1.54	2.2440	0.07	[−0.55; 0.69]	1.8%	2.8%
Nikolaos Markou 2023	20	1.37	1.6210	20	1.54	2.2440	−0.08	[−0.70; 0.54]	1.8%	2.8%
Nikolaos Markou 2023	20	1.29	1.6210	20	1.54	2.2440	−0.13	[−0.75; 0.50]	1.8%	2.8%
Filippo Graziani 2022	20	4.52	7.7500	20	3.60	3.2800	0.15	[−0.47; 0.77]	1.7%	2.8%
Filippo Graziani 2022	20	4.38	4.3000	20	5.21	4.7400	−0.18	[−0.80; 0.44]	1.7%	2.8%
Wang 2017	19	13.35	8.4700	19	12828.40	13890.0000	−1.28	[−1.98; −0.57]	1.4%	2.6%
Kapellas 2016	24	3.70	2.1000	24	3.30	2.8900	0.16	[−0.41; 0.72]	2.1%	2.9%
Geisinger 2016	240	2.64	2.3300	240	2.67	2.9300	−0.01	[−0.19; 0.17]	21.1%	3.6%
Yong−Wei Fu 2015	54	1.18	0.4400	54	1.96	0.4900	−1.66	[−2.10; −1.22]	3.5%	3.2%
Yong−Wei Fu 2015	54	1.24	0.3900	54	1.96	0.4900	−1.61	[−2.05; −1.18]	3.5%	3.2%
**Common effect model**	**1071**			**1071**			**−0.20**	**[−0.29; −0.11]**	**90.5%**	**.**
**Random effects model**							**−0.23**	**[−0.43; −0.03]**	**.**	**83.6%**
Heterogeneity: $I^2 = 80\%$, $\tau^2 = 0.2248$, $p < 0.01$										
**Smoke_binary:Sample without smokers**										
Steven Offenbacher 2006	28	1.49	1.0500	28	0.47	0.2300	1.32	[0.74; 1.91]	2.0%	2.9%
Escobar Arregoces 2021	19	0.24	0.1300	19	0.31	0.1800	−0.44	[−1.08; 0.21]	1.6%	2.8%
de Sousa Rabelo 2020	15	7.83	17.3000	15	5.25	8.9900	0.18	[−0.54; 0.90]	1.3%	2.6%
de Sousa Rabelo 2020	15	3.62	8.1300	15	0.77	0.6220	0.48	[−0.25; 1.21]	1.3%	2.6%
de Sousa Rabelo 2020	15	2.87	5.7740	15	1.54	2.3900	0.29	[−0.43; 1.01]	1.3%	2.6%
Lobao 2018	33	3.94	3.3300	33	15.97	8.5800	−1.83	[−2.41; −1.25]	2.0%	2.9%
**Common effect model**	**125**			**125**			**−0.05**	**[−0.32; 0.21]**	**9.5%**	**.**
**Random effects model**							**−0.00**	**[−0.87; 0.86]**	**.**	**16.4%**
Heterogeneity: $I^2 = 92\%$, $\tau^2 = 1.0575$, $p < 0.01$										
**Common effect model**	**1196**			**1196**			**−0.19**	**[−0.27; −0.10]**	**100.0%**	**.**
**Random effects model**							**−0.19**	**[−0.41; 0.02]**	**.**	**100.0%**

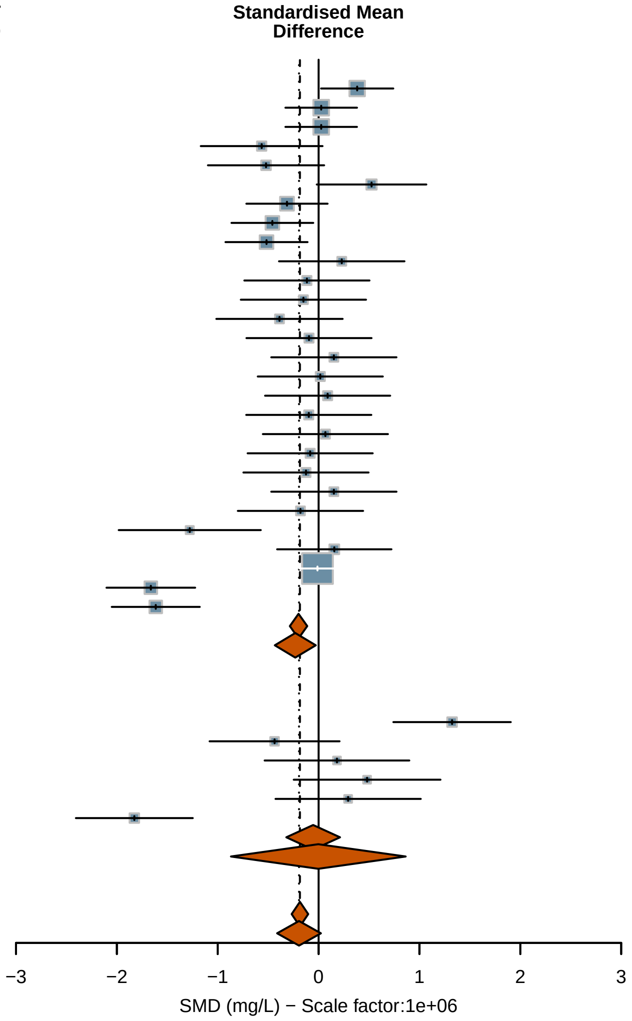

Heterogeneity: $I^2 = 83\%$, $\tau^2 = 0.3350$, $p < 0.01$
Test for subgroup differences (common effect): $\chi^2_1 = 1.03$, df = 1 ($p = 0.31$)
Test for subgroup differences (random effects): $\chi^2_1 = 0.25$, df = 1 ($p = 0.62$)

SMD: −0.19; 95%C.I.[−0.27; −0.1] P value for common effect= 0

SMD: −0.19; 95%C.I.[−0.41; 0.02] P value for random effect= 0.0799

Cytokine: IL−6 − Treatment: Intensive

Study	After TRT Total	After TRT Mean	After TRT SD	Before TRT Total	Before TRT Mean	Before TRT SD	SMD	95%−CI	Weight (common)	Weight (random)
**Diabetes_binary:Sample with diabetic**										
Shih−Jung Lin 2011	14	1.53	0.7200	14	1.52	0.5100	0.02	[−0.72; 0.76]	1.7%	4.0%
Shih−Jung Lin 2011	14	1.74	0.5800	14	1.52	0.5100	0.39	[−0.36; 1.14]	1.6%	4.0%
Eduardo Machado Vilela 2011	36	4.08	3.0000	36	6.18	5.3000	−0.48	[−0.95; −0.01]	4.2%	4.6%
Hilana Paula Carillo ARTESE 2015	12	10900.00	2700.0000	12	13000.00	4400.0000	−0.56	[−1.37; 0.26]	1.4%	3.8%
Filippo Graziani 2022	20	4.52	7.7500	20	3.60	3.2800	0.15	[−0.47; 0.77]	2.4%	4.2%
Filippo Graziani 2022	20	4.38	4.3000	20	5.21	4.7400	−0.18	[−0.80; 0.44]	2.4%	4.2%
de Sousa Rabelo 2020	15	2.87	5.7740	15	1.54	2.3900	0.29	[−0.43; 1.01]	1.8%	4.0%
Wang 2017	19	13.35	8.4700	19	12828.40	13890.0000	−1.28	[−1.98; −0.57]	1.8%	4.1%
Kapellas 2016	24	3.70	2.1000	24	3.30	2.8900	0.16	[−0.41; 0.72]	2.9%	4.4%
Geisinger 2016	240	2.64	2.3300	240	2.67	2.9300	−0.01	[−0.19; 0.17]	28.6%	5.0%
**Common effect model**	**414**			**414**			**−0.08**	**[−0.22; 0.06]**	**48.7%**	**.**
**Random effects model**							**−0.14**	**[−0.41; 0.14]**	**.**	**42.2%**
Heterogeneity: $I^2 = 54\%$, $\tau^2 = 0.1027$, $p = 0.02$										
**Diabetes_binary:Sample without diabetic**										
Steven Offenbacher 2006	28	1.49	1.0500	28	0.47	0.2300	1.32	[0.74; 1.91]	2.7%	4.3%
Tonetti 2018	61	3.90	1.1000	61	2.40	5.4000	0.38	[0.02; 0.74]	7.1%	4.8%
Tonetti 2018	61	2.50	0.7000	61	2.40	5.4000	0.03	[−0.33; 0.38]	7.3%	4.8%
Tonetti 2018	61	2.50	0.8000	61	2.40	5.4000	0.03	[−0.33; 0.38]	7.3%	4.8%
Eduardo Machado Vilela 2011	20	2.09	1.4000	20	2.95	2.2000	−0.46	[−1.09; 0.17]	2.3%	4.2%
Umut Altay 2013	22	0.77	0.8700	22	1.27	0.8700	−0.56	[−1.17; 0.04]	2.5%	4.3%
Umut Altay 2013	24	0.77	0.7900	24	1.10	0.3900	−0.52	[−1.10; 0.06]	2.8%	4.3%
Fiorini T 2012	27	5.50	2.3000	27	4.40	1.8000	0.52	[−0.02; 1.07]	3.1%	4.4%
Escobar Arregoces 2021	19	0.24	0.1300	19	0.31	0.1800	−0.44	[−1.08; 0.21]	2.2%	4.2%
de Sousa Rabelo 2020	15	7.83	17.3000	15	5.25	8.9900	0.18	[−0.54; 0.90]	1.8%	4.0%
Lobao 2018	33	3.94	3.3300	33	15.97	8.5800	−1.83	[−2.41; −1.25]	2.7%	4.3%
Yong−Wei Fu 2015	54	1.18	0.4400	54	1.96	0.4900	−1.66	[−2.10; −1.22]	4.7%	4.6%
Yong−Wei Fu 2015	54	1.24	0.3900	54	1.96	0.4900	−1.61	[−2.05; −1.18]	4.8%	4.6%
**Common effect model**	**479**			**479**			**−0.33**	**[−0.46; −0.20]**	**51.3%**	**.**
**Random effects model**							**−0.36**	**[−0.86; 0.15]**	**.**	**57.8%**
Heterogeneity: $I^2 = 93\%$, $\tau^2 = 0.7878$, $p < 0.01$										
**Common effect model**	**893**			**893**			**−0.21**	**[−0.30; −0.11]**	**100.0%**	**.**
**Random effects model**							**−0.27**	**[−0.58; 0.05]**	**.**	**100.0%**

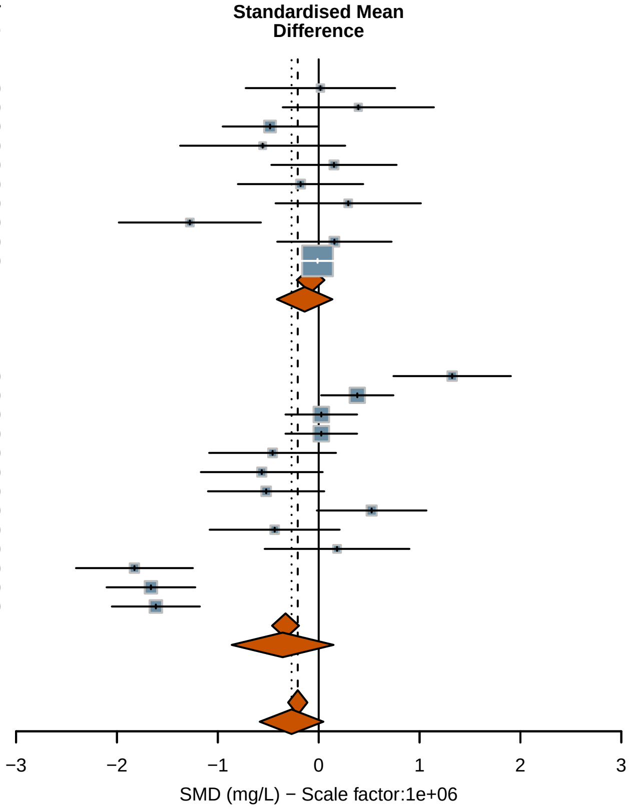

Heterogeneity: $I^2 = 88\%$, $\tau^2 = 0.5091$, $p < 0.01$
Test for subgroup differences (common effect): $\chi^2_1 = 6.49$, df = 1 ($p = 0.01$)
Test for subgroup differences (random effects): $\chi^2_1 = 0.55$, df = 1 ($p = 0.46$)

SMD: −0.21; 95%C.I.[−0.3; −0.11] P value for common effect= 0

SMD: −0.27; 95%C.I.[−0.58; 0.05] P value for random effect= 0.0947

Meta−Regression for SMD on IL−6 − Treatment: Intensive

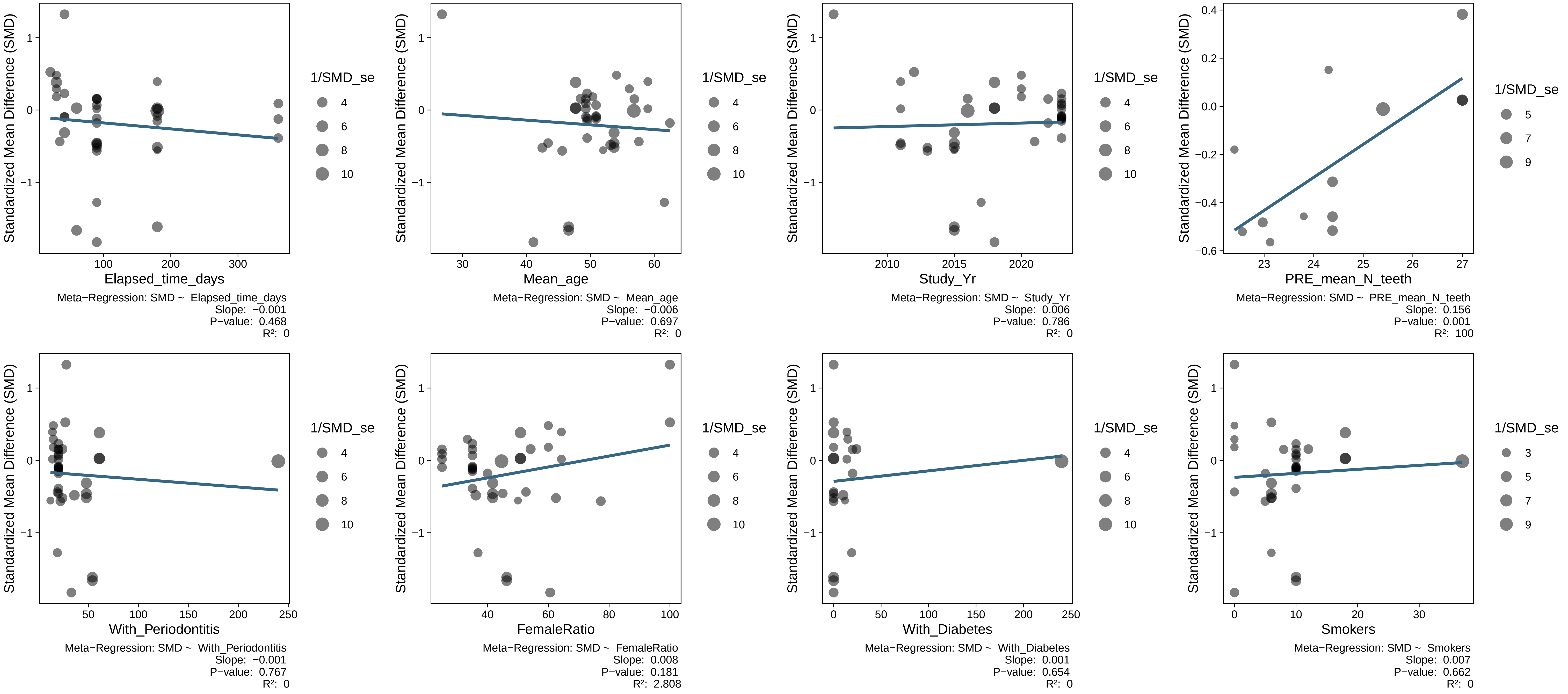

Supplement: Supplementary file 1 [file DataSheet1.zip › Supplementary materials/PDF/IL-6_Intensive_results.pdf]
