# Supplementary material for: Temporal dynamics of early inflammatory markers after professional dental cleaning: a meta-analysis and spline-based meta-regression of TNF-α, IL-1β, IL-6, and (hs)CRP
Source: Front Immunol. 2025 Aug 28;16:1634622. doi: 10.3389/fimmu.2025.1634622 (PMC12423065; doi:10.3389/fimmu.2025.1634622)
Supplement: Supplementary file 1 [file DataSheet1.zip › Supplementary materials/PDF/IL-6_Standard_results.pdf]

Cytokine: IL−6 − Treatment: Standard

Study	After TRT Total	After TRT Mean	After TRT SD	Before TRT Total	Before TRT Mean	Before TRT SD	SMD	95%−CI	Weight (common)	Weight (random)
**Days after treatment:30**										
Tonetti 2018	59	1.59	0.4700	59	2.10	3.9000	−0.18	[−0.54; 0.18]	12.2%	9.1%
Ushida 2008	12	0.52	0.3100	12	0.51	0.2800	0.03	[−0.77; 0.83]	2.5%	3.7%
Ushida 2008	12	0.56	0.2700	12	0.86	0.3700	−0.89	[−1.74; −0.05]	2.2%	3.4%
Levent Kardesxler 2010	13	2.70	1.3000	13	6.10	3.6000	−1.22	[−2.06; −0.37]	2.2%	3.4%
Levent Kardesxler 2010	12	2.50	1.9000	12	3.60	2.7000	−0.45	[−1.27; 0.36]	2.4%	3.6%
Levent Kardesxler 2010	15	2.00	1.7000	15	3.50	2.6000	−0.66	[−1.40; 0.07]	2.9%	4.2%
Yasuko Shimada 2010	33	1.40	0.7000	33	1.70	1.0000	−0.34	[−0.83; 0.14]	6.8%	7.0%
**Common effect model**	**156**			**156**			**−0.39**	**[−0.62; −0.16]**	**31.3%**	**.**
**Random effects model**							**−0.43**	**[−0.70; −0.16]**	**.**	**34.5%**
Heterogeneity: $I^2 = 24\%$, $\tau^2 = 0.0268$, $p = 0.24$										
**Days after treatment:42**										
Ide M 2003	24	2.02	0.7000	24	1.58	0.3800	0.77	[0.18; 1.36]	4.6%	5.7%
**Days after treatment:60**										
F. D'Aiuto 2005	21	1900.00	710.0000	21	1930.00	870.0000	−0.04	[−0.64; 0.57]	4.4%	5.5%
Tonetti 2018	59	1.78	0.5100	59	2.10	3.9000	−0.11	[−0.48; 0.25]	12.3%	9.1%
Yong−Wei Fu 2015	55	1.83	0.4500	55	1.92	0.4700	−0.19	[−0.57; 0.18]	11.4%	8.9%
**Common effect model**	**135**			**135**			**−0.13**	**[−0.37; 0.10]**	**28.0%**	**.**
**Random effects model**							**−0.13**	**[−0.37; 0.10]**	**.**	**23.5%**
Heterogeneity: $I^2 = 0\%$, $\tau^2 = 0$, $p = 0.90$										
**Days after treatment:90**										
Vidal 2009	11	10.00	25.8000	11	24.40	69.5000	−0.26	[−1.10; 0.58]	2.3%	3.4%
Levent Kardesxler 2010	13	2.90	2.0000	13	6.10	3.6000	−1.06	[−1.89; −0.23]	2.3%	3.5%
Levent Kardesxler 2010	12	2.80	0.5000	12	3.60	2.7000	−0.40	[−1.21; 0.41]	2.4%	3.6%
Levent Kardesxler 2010	15	1.50	1.4000	15	3.50	2.6000	−0.93	[−1.69; −0.17]	2.8%	4.0%
**Common effect model**	**51**			**51**			**−0.68**	**[−1.08; −0.27]**	**9.8%**	**.**
**Random effects model**							**−0.68**	**[−1.08; −0.27]**	**.**	**14.6%**
Heterogeneity: $I^2 = 0\%$, $\tau^2 = 0$, $p = 0.45$										
**Days after treatment:180**										
Tonetti 2018	59	1.69	0.4800	59	2.10	3.9000	−0.15	[−0.51; 0.21]	12.3%	9.1%
Hilana Paula Carillo ARTESE 2015	12	13700.00	4000.0000	12	14100.00	5400.0000	−0.08	[−0.88; 0.72]	2.5%	3.7%
Yong−Wei Fu 2015	55	1.87	0.5100	55	1.92	0.4700	−0.10	[−0.48; 0.27]	11.4%	8.9%
**Common effect model**	**126**			**126**			**−0.12**	**[−0.37; 0.13]**	**26.2%**	**.**
**Random effects model**							**−0.12**	**[−0.37; 0.13]**	**.**	**21.8%**
Heterogeneity: $I^2 = 0\%$, $\tau^2 = 0$, $p = 0.98$										
**Common effect model**	**492**			**492**			**−0.22**	**[−0.35; −0.10]**	**100.0%**	**.**
**Random effects model**							**−0.27**	**[−0.44; −0.09]**	**.**	**100.0%**

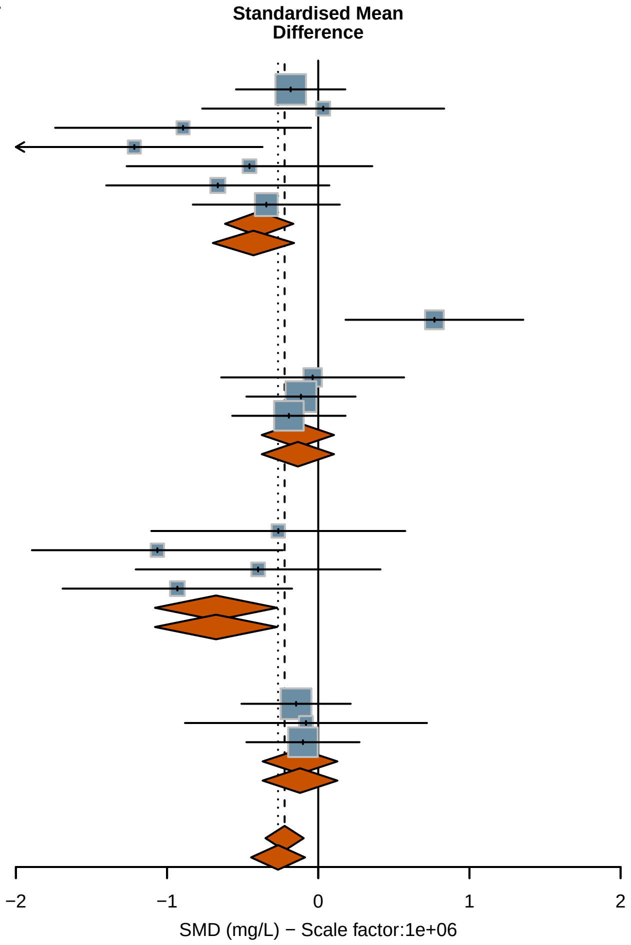

Heterogeneity: $I^2 = 43\%$, $\tau^2 = 0.0567$, $p = 0.03$
Test for subgroup differences (common effect): $\chi^2_4 = 19.05$, df = 4 ($p < 0.01$)
Test for subgroup differences (random effects): $\chi^2_4 = 19.18$, df = 4 ($p < 0.01$)

SMD: −0.22; 95%C.I.[−0.35; −0.1] P value for common effect= 6e−04

SMD: −0.27; 95%C.I.[−0.44; −0.09] P value for random effect= 0.0035

Cytokine: IL−6 − Treatment: Standard

Study	Total	Mean	After TRT SD	Total	Mean	Before TRT SD	SMD	95%−CI	Weight (common)	Weight (random)
**MonthsAfterTRT:4−6Wks**										
Ide M 2003	24	2.02	0.7000	24	1.58	0.3800	0.77	[0.18; 1.36]	4.6%	5.7%
Tonetti 2018	59	1.59	0.4700	59	2.10	3.9000	−0.18	[−0.54; 0.18]	12.2%	9.1%
Ushida 2008	12	0.52	0.3100	12	0.51	0.2800	0.03	[−0.77; 0.83]	2.5%	3.7%
Ushida 2008	12	0.56	0.2700	12	0.86	0.3700	−0.89	[−1.74; −0.05]	2.2%	3.4%
Levent Kardesxler 2010	13	2.70	1.3000	13	6.10	3.6000	−1.22	[−2.06; −0.37]	2.2%	3.4%
Levent Kardesxler 2010	12	2.50	1.9000	12	3.60	2.7000	−0.45	[−1.27; 0.36]	2.4%	3.6%
Levent Kardesxler 2010	15	2.00	1.7000	15	3.50	2.6000	−0.66	[−1.40; 0.07]	2.9%	4.2%
Yasuko Shimada 2010	33	1.40	0.7000	33	1.70	1.0000	−0.34	[−0.83; 0.14]	6.8%	7.0%
**Common effect model**	**180**			**180**			**−0.24**	**[−0.45; −0.03]**	**36.0%**	**.**
**Random effects model**							**−0.32**	**[−0.74; 0.09]**	**.**	**40.1%**
Heterogeneity: $I^2 = 67\%$, $\tau^2 = 0.2345$, $p < 0.01$										
**MonthsAfterTRT:Within 3 Mo.**										
F. D'Aiuto 2005	21	1900.00	710.0000	21	1930.00	870.0000	−0.04	[−0.64; 0.57]	4.4%	5.5%
Tonetti 2018	59	1.78	0.5100	59	2.10	3.9000	−0.11	[−0.48; 0.25]	12.3%	9.1%
Vidal 2009	11	10.00	25.8000	11	24.40	69.5000	−0.26	[−1.10; 0.58]	2.3%	3.4%
Levent Kardesxler 2010	13	2.90	2.0000	13	6.10	3.6000	−1.06	[−1.89; −0.23]	2.3%	3.5%
Levent Kardesxler 2010	12	2.80	0.5000	12	3.60	2.7000	−0.40	[−1.21; 0.41]	2.4%	3.6%
Levent Kardesxler 2010	15	1.50	1.4000	15	3.50	2.6000	−0.93	[−1.69; −0.17]	2.8%	4.0%
Yong−Wei Fu 2015	55	1.83	0.4500	55	1.92	0.4700	−0.19	[−0.57; 0.18]	11.4%	8.9%
**Common effect model**	**186**			**186**			**−0.28**	**[−0.48; −0.07]**	**37.9%**	**.**
**Random effects model**							**−0.29**	**[−0.51; −0.07]**	**.**	**38.1%**
Heterogeneity: $I^2 = 25\%$, $\tau^2 = 0.0074$, $p = 0.24$										
**MonthsAfterTRT:Within 6 Mo.**										
Tonetti 2018	59	1.69	0.4800	59	2.10	3.9000	−0.15	[−0.51; 0.21]	12.3%	9.1%
Hilana Paula Carillo ARTESE 2015	12	13700.00	4000.0000	12	14100.00	5400.0000	−0.08	[−0.88; 0.72]	2.5%	3.7%
Yong−Wei Fu 2015	55	1.87	0.5100	55	1.92	0.4700	−0.10	[−0.48; 0.27]	11.4%	8.9%
**Common effect model**	**126**			**126**			**−0.12**	**[−0.37; 0.13]**	**26.2%**	**.**
**Random effects model**							**−0.12**	**[−0.37; 0.13]**	**.**	**21.8%**
Heterogeneity: $I^2 = 0\%$, $\tau^2 = 0$, $p = 0.98$										
**Common effect model**	**492**			**492**			**−0.22**	**[−0.35; −0.10]**	**100.0%**	**.**
**Random effects model**							**−0.27**	**[−0.44; −0.09]**	**.**	**100.0%**

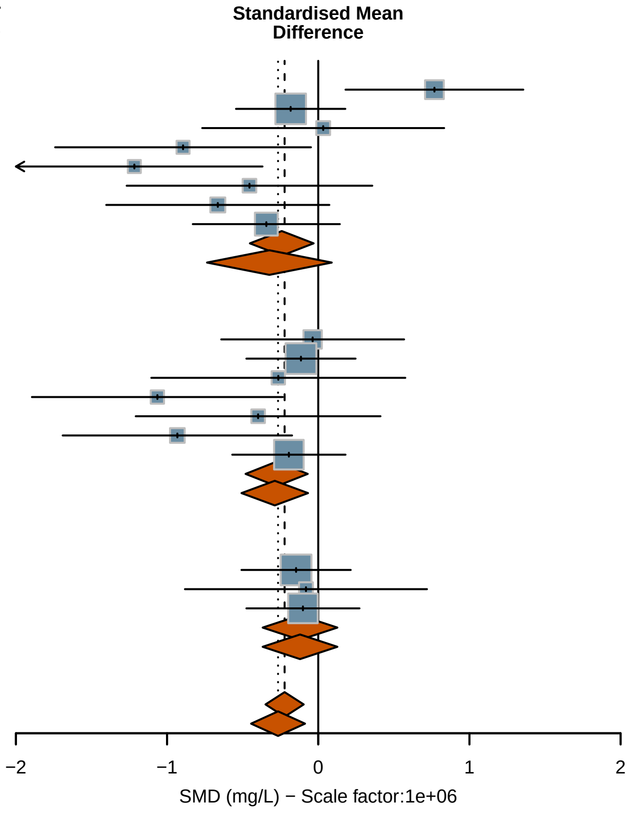

Heterogeneity: $I^2 = 43\%$, $\tau^2 = 0.0567$, $p = 0.03$
Test for subgroup differences (common effect): $\chi^2_2 = 0.94$, df = 2 ($p = 0.63$)
Test for subgroup differences (random effects): $\chi^2_2 = 1.21$, df = 2 ($p = 0.55$)

SMD: −0.22; 95%C.I.[−0.35; −0.1] P value for common effect= 6e−04

SMD: −0.27; 95%C.I.[−0.44; −0.09] P value for random effect= 0.0035

Cytokine: IL−6 − Treatment: Standard

Study	After TRT Total	After TRT Mean	After TRT SD	Before TRT Total	Before TRT Mean	Before TRT SD	SMD	95%−CI	Weight (common)	Weight (random)
**Assay type:ELISA**										
Ide M 2003	24	2.02	0.7000	24	1.58	0.3800	0.77	[0.18; 1.36]	4.6%	5.7%
F. D'Aiuto 2005	21	1900.00	710.0000	21	1930.00	870.0000	−0.04	[−0.64; 0.57]	4.4%	5.5%
Tonetti 2018	59	1.59	0.4700	59	2.10	3.9000	−0.18	[−0.54; 0.18]	12.2%	9.1%
Tonetti 2018	59	1.78	0.5100	59	2.10	3.9000	−0.11	[−0.48; 0.25]	12.3%	9.1%
Tonetti 2018	59	1.69	0.4800	59	2.10	3.9000	−0.15	[−0.51; 0.21]	12.3%	9.1%
Ushida 2008	12	0.52	0.3100	12	0.51	0.2800	0.03	[−0.77; 0.83]	2.5%	3.7%
Ushida 2008	12	0.56	0.2700	12	0.86	0.3700	−0.89	[−1.74; −0.05]	2.2%	3.4%
Vidal 2009	11	10.00	25.8000	11	24.40	69.5000	−0.26	[−1.10; 0.58]	2.3%	3.4%
Levent Kardesxler 2010	13	2.70	1.3000	13	6.10	3.6000	−1.22	[−2.06; −0.37]	2.2%	3.4%
Levent Kardesxler 2010	13	2.90	2.0000	13	6.10	3.6000	−1.06	[−1.89; −0.23]	2.3%	3.5%
Levent Kardesxler 2010	12	2.50	1.9000	12	3.60	2.7000	−0.45	[−1.27; 0.36]	2.4%	3.6%
Levent Kardesxler 2010	12	2.80	0.5000	12	3.60	2.7000	−0.40	[−1.21; 0.41]	2.4%	3.6%
Levent Kardesxler 2010	15	2.00	1.7000	15	3.50	2.6000	−0.66	[−1.40; 0.07]	2.9%	4.2%
Levent Kardesxler 2010	15	1.50	1.4000	15	3.50	2.6000	−0.93	[−1.69; −0.17]	2.8%	4.0%
Yasuko Shimada 2010	33	1.40	0.7000	33	1.70	1.0000	−0.34	[−0.83; 0.14]	6.8%	7.0%
Hilana Paula Carillo ARTESE 2015	12	13700.00	4000.0000	12	14100.00	5400.0000	−0.08	[−0.88; 0.72]	2.5%	3.7%
Yong−Wei Fu 2015	55	1.83	0.4500	55	1.92	0.4700	−0.19	[−0.57; 0.18]	11.4%	8.9%
Yong−Wei Fu 2015	55	1.87	0.5100	55	1.92	0.4700	−0.10	[−0.48; 0.27]	11.4%	8.9%
**Common effect model**	**492**			**492**			**−0.22**	**[−0.35; −0.10]**	**100.0%**	**.**
**Random effects model**							**−0.27**	**[−0.44; −0.09]**	**.**	**100.0%**
Heterogeneity: $I^2 = 43\%$, $\tau^2 = 0.0567$, $p = 0.03$										
**Common effect model**	**492**			**492**			**−0.22**	**[−0.35; −0.10]**	**100.0%**	**.**
**Random effects model**							**−0.27**	**[−0.44; −0.09]**	**.**	**100.0%**

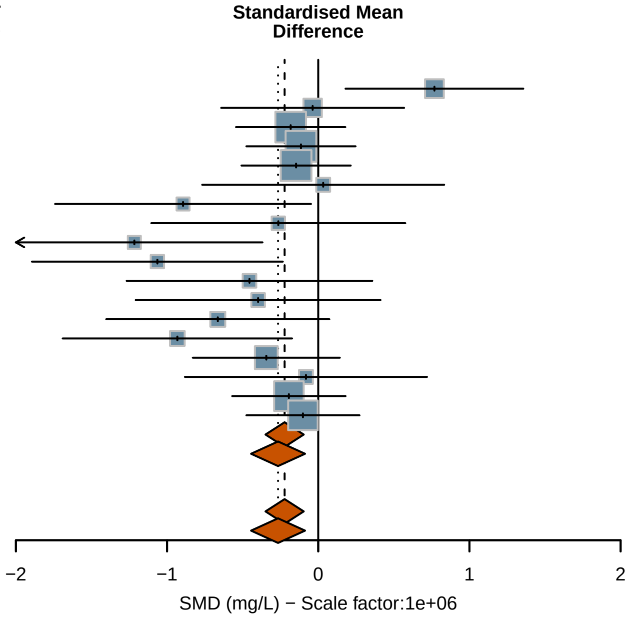

Heterogeneity: $I^2 = 43\%$, $\tau^2 = 0.0567$, $p = 0.03$
Test for subgroup differences (common effect): $\chi^2_0 = 0.00$, df = 0 (p = NA)
Test for subgroup differences (random effects): $\chi^2_0 = 0.00$, df = 0 (p = NA)

SMD: −0.22; 95%C.I.[−0.35; −0.1] P value for common effect= 6e−04

SMD: −0.27; 95%C.I.[−0.44; −0.09] P value for random effect= 0.0035

Cytokine: IL−6 − Treatment: Standard

Study	After TRT Total	After TRT Mean	After TRT SD	Before TRT Total	Before TRT Mean	Before TRT SD	SMD	95%−CI	Weight (common)	Weight (random)
**Bias judgment:High risk**										
Ushida 2008	12	0.52	0.3100	12	0.51	0.2800	0.03	[−0.77; 0.83]	2.5%	3.7%
Ushida 2008	12	0.56	0.2700	12	0.86	0.3700	−0.89	[−1.74; −0.05]	2.2%	3.4%
Vidal 2009	11	10.00	25.8000	11	24.40	69.5000	−0.26	[−1.10; 0.58]	2.3%	3.4%
Yasuko Shimada 2010	33	1.40	0.7000	33	1.70	1.0000	−0.34	[−0.83; 0.14]	6.8%	7.0%
Hilana Paula Carillo ARTESE 2015	12	13700.00	4000.0000	12	14100.00	5400.0000	−0.08	[−0.88; 0.72]	2.5%	3.7%
**Common effect model**	**80**			**80**			**−0.31**	**[−0.62; 0.00]**	**16.3%**	**.**
**Random effects model**							**−0.31**	**[−0.62; 0.00]**	**.**	**21.3%**
Heterogeneity: $I^2 = 0\%$, $\tau^2 = 0$, $p = 0.58$										
**Bias judgment:Low risk**										
Ide M 2003	24	2.02	0.7000	24	1.58	0.3800	0.77	[0.18; 1.36]	4.6%	5.7%
Tonetti 2018	59	1.59	0.4700	59	2.10	3.9000	−0.18	[−0.54; 0.18]	12.2%	9.1%
Tonetti 2018	59	1.78	0.5100	59	2.10	3.9000	−0.11	[−0.48; 0.25]	12.3%	9.1%
Tonetti 2018	59	1.69	0.4800	59	2.10	3.9000	−0.15	[−0.51; 0.21]	12.3%	9.1%
Levent Kardesxler 2010	13	2.70	1.3000	13	6.10	3.6000	−1.22	[−2.06; −0.37]	2.2%	3.4%
Levent Kardesxler 2010	13	2.90	2.0000	13	6.10	3.6000	−1.06	[−1.89; −0.23]	2.3%	3.5%
Levent Kardesxler 2010	12	2.50	1.9000	12	3.60	2.7000	−0.45	[−1.27; 0.36]	2.4%	3.6%
Levent Kardesxler 2010	12	2.80	0.5000	12	3.60	2.7000	−0.40	[−1.21; 0.41]	2.4%	3.6%
Levent Kardesxler 2010	15	2.00	1.7000	15	3.50	2.6000	−0.66	[−1.40; 0.07]	2.9%	4.2%
Levent Kardesxler 2010	15	1.50	1.4000	15	3.50	2.6000	−0.93	[−1.69; −0.17]	2.8%	4.0%
Yong−Wei Fu 2015	55	1.83	0.4500	55	1.92	0.4700	−0.19	[−0.57; 0.18]	11.4%	8.9%
Yong−Wei Fu 2015	55	1.87	0.5100	55	1.92	0.4700	−0.10	[−0.48; 0.27]	11.4%	8.9%
**Common effect model**	**391**			**391**			**−0.21**	**[−0.36; −0.07]**	**79.4%**	**.**
**Random effects model**							**−0.31**	**[−0.57; −0.04]**	**.**	**73.3%**
Heterogeneity: $I^2 = 58\%$, $\tau^2 = 0.1290$, $p < 0.01$										
**Bias judgment:Some concerns**										
F. D'Aiuto 2005	21	1900.00	710.0000	21	1930.00	870.0000	−0.04	[−0.64; 0.57]	4.4%	5.5%
**Common effect model**	**492**			**492**			**−0.22**	**[−0.35; −0.10]**	**100.0%**	**.**
**Random effects model**							**−0.27**	**[−0.44; −0.09]**	**.**	**100.0%**

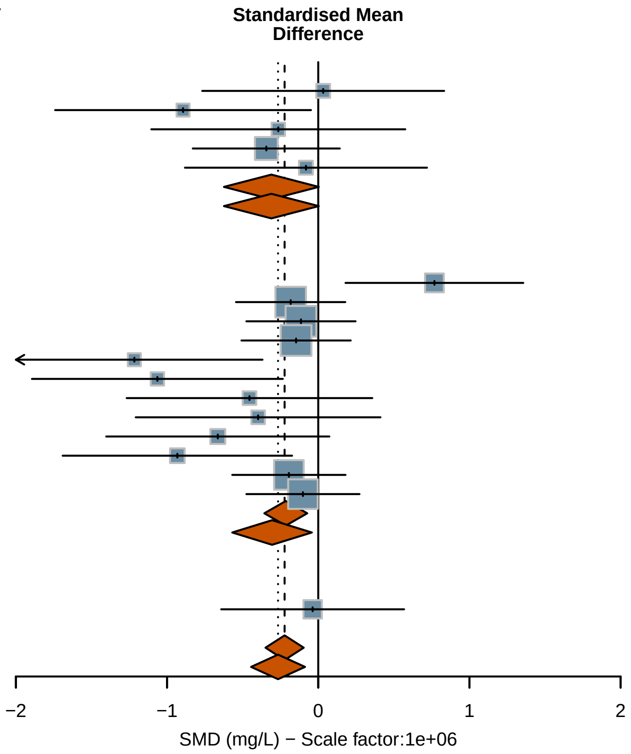

Heterogeneity: $I^2 = 43\%$, $\tau^2 = 0.0567$, $p = 0.03$
Test for subgroup differences (common effect): $\chi^2_2 = 0.67$, df = 2 ($p = 0.71$)
Test for subgroup differences (random effects): $\chi^2_2 = 0.69$, df = 2 ($p = 0.71$)

SMD: −0.22; 95%C.I.[−0.35; −0.1] P value for common effect= 6e−04

SMD: −0.27; 95%C.I.[−0.44; −0.09] P value for random effect= 0.0035

Cytokine: IL−6 − Treatment: Standard

Study	After TRT Total	After TRT Mean	After TRT SD	Before TRT Total	Before TRT Mean	Before TRT SD	SMD	95%−CI	Weight (common)	Weight (random)
**Study design:Comparative study**										
Levent Kardesxler 2010	13	2.70	1.3000	13	6.10	3.6000	−1.22	[−2.06; −0.37]	2.2%	3.4%
Levent Kardesxler 2010	13	2.90	2.0000	13	6.10	3.6000	−1.06	[−1.89; −0.23]	2.3%	3.5%
Levent Kardesxler 2010	12	2.50	1.9000	12	3.60	2.7000	−0.45	[−1.27; 0.36]	2.4%	3.6%
Levent Kardesxler 2010	12	2.80	0.5000	12	3.60	2.7000	−0.40	[−1.21; 0.41]	2.4%	3.6%
Levent Kardesxler 2010	15	2.00	1.7000	15	3.50	2.6000	−0.66	[−1.40; 0.07]	2.9%	4.2%
Levent Kardesxler 2010	15	1.50	1.4000	15	3.50	2.6000	−0.93	[−1.69; −0.17]	2.8%	4.0%
Yasuko Shimada 2010	33	1.40	0.7000	33	1.70	1.0000	−0.34	[−0.83; 0.14]	6.8%	7.0%
**Common effect model**	**113**			**113**			**−0.64**	**[−0.92; −0.37]**	**21.9%**	**.**
**Random effects model**							**−0.65**	**[−0.93; −0.37]**	**.**	**29.4%**
Heterogeneity: $I^2 = 0\%$, $\tau^2 = 0.0047$, $p = 0.50$										
**Study design:Prospective Interventional cohort study**										
Ide M 2003	24	2.02	0.7000	24	1.58	0.3800	0.77	[0.18; 1.36]	4.6%	5.7%
**Study design:RCT**										
F. D'Aiuto 2005	21	1900.00	710.0000	21	1930.00	870.0000	−0.04	[−0.64; 0.57]	4.4%	5.5%
Tonetti 2018	59	1.59	0.4700	59	2.10	3.9000	−0.18	[−0.54; 0.18]	12.2%	9.1%
Tonetti 2018	59	1.78	0.5100	59	2.10	3.9000	−0.11	[−0.48; 0.25]	12.3%	9.1%
Tonetti 2018	59	1.69	0.4800	59	2.10	3.9000	−0.15	[−0.51; 0.21]	12.3%	9.1%
Ushida 2008	12	0.52	0.3100	12	0.51	0.2800	0.03	[−0.77; 0.83]	2.5%	3.7%
Ushida 2008	12	0.56	0.2700	12	0.86	0.3700	−0.89	[−1.74; −0.05]	2.2%	3.4%
Vidal 2009	11	10.00	25.8000	11	24.40	69.5000	−0.26	[−1.10; 0.58]	2.3%	3.4%
Hilana Paula Carillo ARTESE 2015	12	13700.00	4000.0000	12	14100.00	5400.0000	−0.08	[−0.88; 0.72]	2.5%	3.7%
Yong−Wei Fu 2015	55	1.83	0.4500	55	1.92	0.4700	−0.19	[−0.57; 0.18]	11.4%	8.9%
Yong−Wei Fu 2015	55	1.87	0.5100	55	1.92	0.4700	−0.10	[−0.48; 0.27]	11.4%	8.9%
**Common effect model**	**355**			**355**			**−0.16**	**[−0.31; −0.01]**	**73.5%**	**.**
**Random effects model**							**−0.16**	**[−0.31; −0.01]**	**.**	**65.0%**
Heterogeneity: $I^2 = 0\%$, $\tau^2 = 0$, $p = 0.94$										
**Common effect model**	**492**			**492**			**−0.22**	**[−0.35; −0.10]**	**100.0%**	**.**
**Random effects model**							**−0.27**	**[−0.44; −0.09]**	**.**	**100.0%**

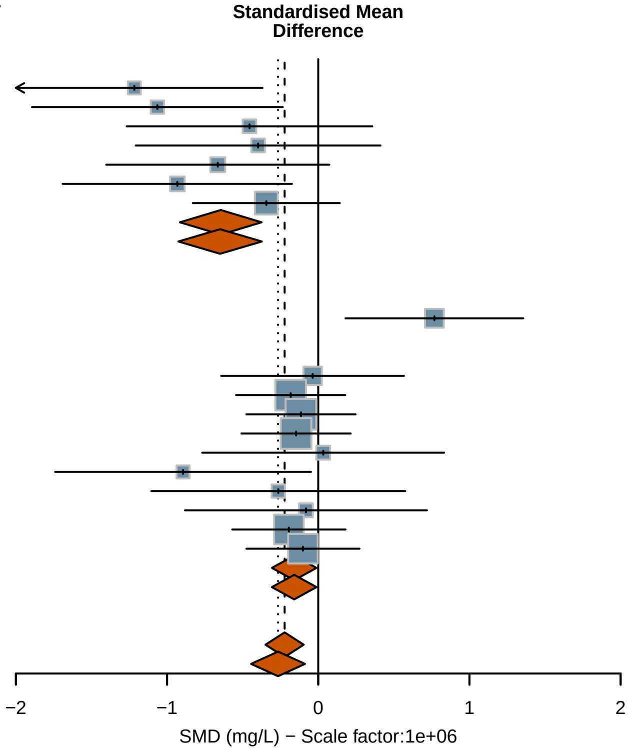

Heterogeneity: $I^2 = 43\%$, $\tau^2 = 0.0567$, $p = 0.03$
Test for subgroup differences (common effect): $\chi^2_2 = 21.00$, df = 2 ($p < 0.01$)
Test for subgroup differences (random effects): $\chi^2_2 = 20.79$, df = 2 ($p < 0.01$)

SMD: −0.22; 95%C.I.[−0.35; −0.1] P value for common effect= 6e−04

SMD: −0.27; 95%C.I.[−0.44; −0.09] P value for random effect= 0.0035

Cytokine: IL−6 − Treatment: Standard

Study	After TRT Total	After TRT Mean	After TRT SD	Before TRT Total	Before TRT Mean	Before TRT SD	SMD	95%−CI	Weight (common)	Weight (random)
**Additional_devices:FMD and water**										
Ushida 2008	12	0.52	0.3100	12	0.51	0.2800	0.03	[−0.77; 0.83]	52.8%	51.1%
**Additional_devices:QMD**										
Ushida 2008	12	0.56	0.2700	12	0.86	0.3700	−0.89	[−1.74; −0.05]	47.2%	48.9%
**Common effect model**	**24**			**24**			**−0.40**	**[−0.99; 0.18]**	**100.0%**	**.**
**Random effects model**							**−0.42**	**[−1.33; 0.49]**	**.**	**100.0%**

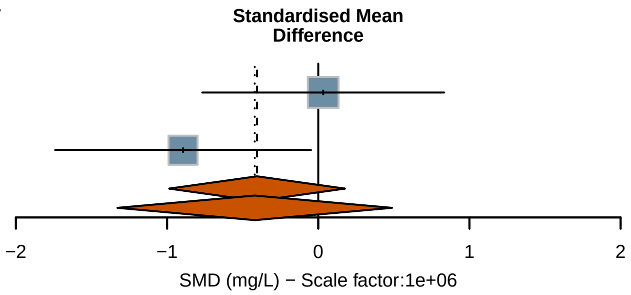

Heterogeneity: $I^2 = 59\%$, $\tau^2 = 0.2531$, $p = 0.12$
Test for subgroup differences (common effect): $\chi^2_1 = 2.43$, df = 1 ($p = 0.12$)
Test for subgroup differences (random effects): $\chi^2_1 = 2.43$, df = 1 ($p = 0.12$)

SMD: −0.4; 95%C.I.[−0.99; 0.18] P value for common effect= 0.1723

SMD: −0.42; 95%C.I.[−1.33; 0.49] P value for random effect= 0.3645

Cytokine: IL−6 − Treatment: Standard

Study	After TRT Total	After TRT Mean	After TRT SD	Before TRT Total	Before TRT Mean	Before TRT SD	SMD	95%−CI	Weight (common)	Weight (random)
**Female_binary:Sex mixed**										
Ide M 2003	24	2.02	0.7000	24	1.58	0.3800	0.77	[0.18; 1.36]	4.6%	5.7%
F. D'Aiuto 2005	21	1900.00	710.0000	21	1930.00	870.0000	−0.04	[−0.64; 0.57]	4.4%	5.5%
Tonetti 2018	59	1.59	0.4700	59	2.10	3.9000	−0.18	[−0.54; 0.18]	12.2%	9.1%
Tonetti 2018	59	1.78	0.5100	59	2.10	3.9000	−0.11	[−0.48; 0.25]	12.3%	9.1%
Tonetti 2018	59	1.69	0.4800	59	2.10	3.9000	−0.15	[−0.51; 0.21]	12.3%	9.1%
Ushida 2008	12	0.52	0.3100	12	0.51	0.2800	0.03	[−0.77; 0.83]	2.5%	3.7%
Ushida 2008	12	0.56	0.2700	12	0.86	0.3700	−0.89	[−1.74; −0.05]	2.2%	3.4%
Vidal 2009	11	10.00	25.8000	11	24.40	69.5000	−0.26	[−1.10; 0.58]	2.3%	3.4%
Levent Kardesxler 2010	13	2.70	1.3000	13	6.10	3.6000	−1.22	[−2.06; −0.37]	2.2%	3.4%
Levent Kardesxler 2010	13	2.90	2.0000	13	6.10	3.6000	−1.06	[−1.89; −0.23]	2.3%	3.5%
Levent Kardesxler 2010	12	2.50	1.9000	12	3.60	2.7000	−0.45	[−1.27; 0.36]	2.4%	3.6%
Levent Kardesxler 2010	12	2.80	0.5000	12	3.60	2.7000	−0.40	[−1.21; 0.41]	2.4%	3.6%
Levent Kardesxler 2010	15	2.00	1.7000	15	3.50	2.6000	−0.66	[−1.40; 0.07]	2.9%	4.2%
Levent Kardesxler 2010	15	1.50	1.4000	15	3.50	2.6000	−0.93	[−1.69; −0.17]	2.8%	4.0%
Yasuko Shimada 2010	33	1.40	0.7000	33	1.70	1.0000	−0.34	[−0.83; 0.14]	6.8%	7.0%
Hilana Paula Carillo ARTESE 2015	12	13700.00	4000.0000	12	14100.00	5400.0000	−0.08	[−0.88; 0.72]	2.5%	3.7%
Yong−Wei Fu 2015	55	1.83	0.4500	55	1.92	0.4700	−0.19	[−0.57; 0.18]	11.4%	8.9%
Yong−Wei Fu 2015	55	1.87	0.5100	55	1.92	0.4700	−0.10	[−0.48; 0.27]	11.4%	8.9%
**Common effect model**	**492**			**492**			**−0.22**	**[−0.35; −0.10]**	**100.0%**	**.**
**Random effects model**							**−0.27**	**[−0.44; −0.09]**	**.**	**100.0%**
Heterogeneity: I^2 = 43%, τ^2 = 0.0567, p = 0.03										
**Common effect model**	**492**			**492**			**−0.22**	**[−0.35; −0.10]**	**100.0%**	**.**
**Random effects model**							**−0.27**	**[−0.44; −0.09]**	**.**	**100.0%**

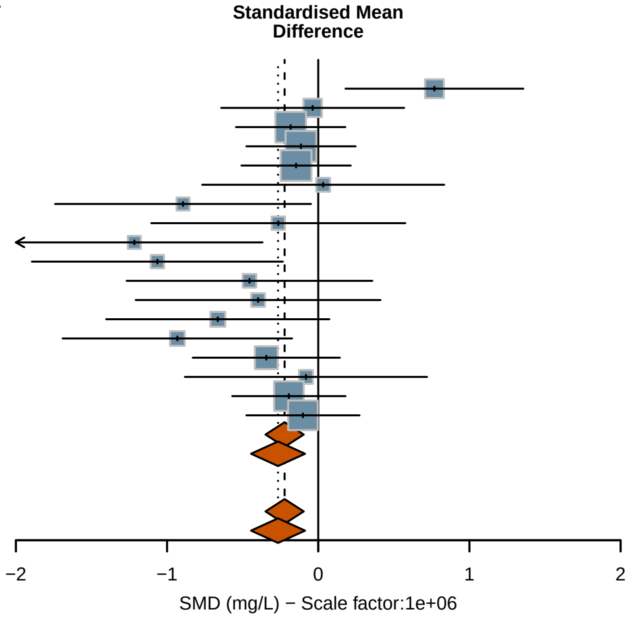

Heterogeneity: I^2 = 43%, τ^2 = 0.0567, p = 0.03
Test for subgroup differences (common effect): χ^2_0 = 0.00, df = 0 (p = NA)
Test for subgroup differences (random effects): χ^2_0 = 0.00, df = 0 (p = NA)

SMD: −0.22; 95%C.I.[−0.35; −0.1] P value for common effect= 6e−04

SMD: −0.27; 95%C.I.[−0.44; −0.09] P value for random effect= 0.0035

Cytokine: IL−6 − Treatment: Standard

Study	Total	After TRT Mean	After TRT SD	Total	Before TRT Mean	Before TRT SD	SMD	95%−CI	Weight (common)	Weight (random)
**Smoke_binary:Sample with smokers**										
F. D'Aiuto 2005	21	1900.00	710.0000	21	1930.00	870.0000	−0.04	[−0.64; 0.57]	4.5%	5.7%
Tonetti 2018	59	1.59	0.4700	59	2.10	3.9000	−0.18	[−0.54; 0.18]	12.5%	9.2%
Tonetti 2018	59	1.78	0.5100	59	2.10	3.9000	−0.11	[−0.48; 0.25]	12.6%	9.2%
Tonetti 2018	59	1.69	0.4800	59	2.10	3.9000	−0.15	[−0.51; 0.21]	12.6%	9.2%
Vidal 2009	11	10.00	25.8000	11	24.40	69.5000	−0.26	[−1.10; 0.58]	2.3%	3.7%
Levent Kardesxler 2010	13	2.70	1.3000	13	6.10	3.6000	−1.22	[−2.06; −0.37]	2.3%	3.7%
Levent Kardesxler 2010	13	2.90	2.0000	13	6.10	3.6000	−1.06	[−1.89; −0.23]	2.4%	3.8%
Levent Kardesxler 2010	12	2.50	1.9000	12	3.60	2.7000	−0.45	[−1.27; 0.36]	2.5%	3.9%
Levent Kardesxler 2010	12	2.80	0.5000	12	3.60	2.7000	−0.40	[−1.21; 0.41]	2.5%	3.9%
Levent Kardesxler 2010	15	2.00	1.7000	15	3.50	2.6000	−0.66	[−1.40; 0.07]	3.0%	4.5%
Levent Kardesxler 2010	15	1.50	1.4000	15	3.50	2.6000	−0.93	[−1.69; −0.17]	2.9%	4.3%
Yasuko Shimada 2010	33	1.40	0.7000	33	1.70	1.0000	−0.34	[−0.83; 0.14]	6.9%	7.2%
Yong−Wei Fu 2015	55	1.83	0.4500	55	1.92	0.4700	−0.19	[−0.57; 0.18]	11.7%	9.0%
Yong−Wei Fu 2015	55	1.87	0.5100	55	1.92	0.4700	−0.10	[−0.48; 0.27]	11.7%	9.0%
**Common effect model**	**432**			**432**			**−0.27**	**[−0.40; −0.13]**	**90.4%**	**.**
**Random effects model**							**−0.27**	**[−0.40; −0.13]**	**.**	**86.4%**
Heterogeneity: $I^2 = 17\%$, $\tau^2 = < 0.0001$, $p = 0.27$										
**Smoke_binary:Sample without smokers**										
Ide M 2003	24	2.02	0.7000	24	1.58	0.3800	0.77	[0.18; 1.36]	4.7%	5.9%
Ushida 2008	12	0.52	0.3100	12	0.51	0.2800	0.03	[−0.77; 0.83]	2.6%	4.0%
Ushida 2008	12	0.56	0.2700	12	0.86	0.3700	−0.89	[−1.74; −0.05]	2.3%	3.7%
**Common effect model**	**48**			**48**			**0.18**	**[−0.24; 0.59]**	**9.6%**	**.**
**Random effects model**							**0.01**	**[−0.94; 0.96]**	**.**	**13.6%**
Heterogeneity: $I^2 = 80\%$, $\tau^2 = 0.5601$, $p < 0.01$										
**Common effect model**	**480**			**480**			**−0.23**	**[−0.35; −0.10]**	**100.0%**	**.**
**Random effects model**							**−0.28**	**[−0.47; −0.09]**	**.**	**100.0%**

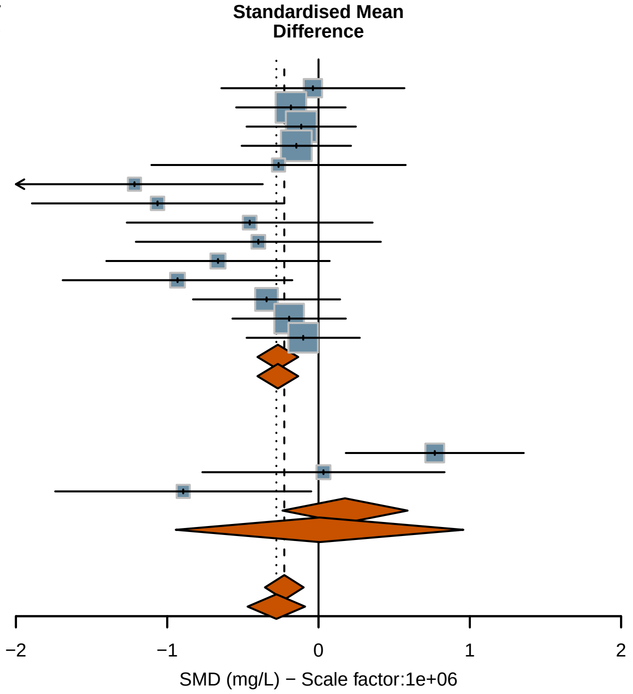

Heterogeneity: $I^2 = 46\%$, $\tau^2 = 0.0682$, $p = 0.02$
Test for subgroup differences (common effect): $\chi^2_1 = 4.00$, df = 1 ($p = 0.05$)
Test for subgroup differences (random effects): $\chi^2_1 = 0.32$, df = 1 ($p = 0.57$)

SMD: −0.23; 95%C.I.[−0.35; −0.1] P value for common effect= 5e−04

SMD: −0.28; 95%C.I.[−0.47; −0.09] P value for random effect= 0.004

Cytokine: IL−6 − Treatment: Standard

Study	After TRT Total	After TRT Mean	After TRT SD	Before TRT Total	Before TRT Mean	Before TRT SD	SMD	95%−CI	Weight (common)	Weight (random)
**Diabetes_binary:Sample with diabetic**										
Levent Kardesxler 2010	13	2.70	1.3000	13	6.10	3.6000	−1.22	[−2.06; −0.37]	2.2%	3.4%
Levent Kardesxler 2010	13	2.90	2.0000	13	6.10	3.6000	−1.06	[−1.89; −0.23]	2.3%	3.5%
Levent Kardesxler 2010	12	2.50	1.9000	12	3.60	2.7000	−0.45	[−1.27; 0.36]	2.4%	3.6%
Levent Kardesxler 2010	12	2.80	0.5000	12	3.60	2.7000	−0.40	[−1.21; 0.41]	2.4%	3.6%
Hilana Paula Carillo ARTESE 2015	12	13700.00	4000.0000	12	14100.00	5400.0000	−0.08	[−0.88; 0.72]	2.5%	3.7%
**Common effect model**	**62**			**62**			**−0.63**	**[−0.99; −0.26]**	**11.9%**	**.**
**Random effects model**							**−0.63**	**[−1.05; −0.21]**	**.**	**17.9%**
Heterogeneity: I^2 = 23%, τ^2 = 0.0507, p = 0.27										
**Diabetes_binary:Sample without diabetic**										
Ide M 2003	24	2.02	0.7000	24	1.58	0.3800	0.77	[0.18; 1.36]	4.6%	5.7%
F. D'Aiuto 2005	21	1900.00	710.0000	21	1930.00	870.0000	−0.04	[−0.64; 0.57]	4.4%	5.5%
Tonetti 2018	59	1.59	0.4700	59	2.10	3.9000	−0.18	[−0.54; 0.18]	12.2%	9.1%
Tonetti 2018	59	1.78	0.5100	59	2.10	3.9000	−0.11	[−0.48; 0.25]	12.3%	9.1%
Tonetti 2018	59	1.69	0.4800	59	2.10	3.9000	−0.15	[−0.51; 0.21]	12.3%	9.1%
Ushida 2008	12	0.52	0.3100	12	0.51	0.2800	0.03	[−0.77; 0.83]	2.5%	3.7%
Ushida 2008	12	0.56	0.2700	12	0.86	0.3700	−0.89	[−1.74; −0.05]	2.2%	3.4%
Vidal 2009	11	10.00	25.8000	11	24.40	69.5000	−0.26	[−1.10; 0.58]	2.3%	3.4%
Levent Kardesxler 2010	15	2.00	1.7000	15	3.50	2.6000	−0.66	[−1.40; 0.07]	2.9%	4.2%
Levent Kardesxler 2010	15	1.50	1.4000	15	3.50	2.6000	−0.93	[−1.69; −0.17]	2.8%	4.0%
Yasuko Shimada 2010	33	1.40	0.7000	33	1.70	1.0000	−0.34	[−0.83; 0.14]	6.8%	7.0%
Yong−Wei Fu 2015	55	1.83	0.4500	55	1.92	0.4700	−0.19	[−0.57; 0.18]	11.4%	8.9%
Yong−Wei Fu 2015	55	1.87	0.5100	55	1.92	0.4700	−0.10	[−0.48; 0.27]	11.4%	8.9%
**Common effect model**	**430**			**430**			**−0.17**	**[−0.30; −0.03]**	**88.1%**	**.**
**Random effects model**							**−0.17**	**[−0.30; −0.03]**	**.**	**82.1%**
Heterogeneity: I^2 = 38%, τ^2 = < 0.0001, p = 0.08										
**Common effect model**	**492**			**492**			**−0.22**	**[−0.35; −0.10]**	**100.0%**	**.**
**Random effects model**							**−0.27**	**[−0.44; −0.09]**	**.**	**100.0%**

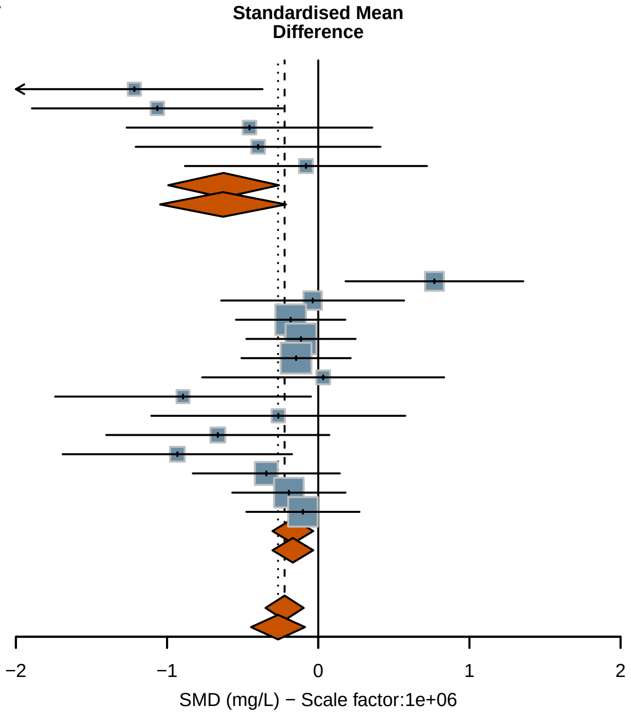

Heterogeneity: I^2 = 43%, τ^2 = 0.0567, p = 0.03
Test for subgroup differences (common effect): χ^2_1 = 5.29, df = 1 (p = 0.02)
Test for subgroup differences (random effects): χ^2_1 = 4.28, df = 1 (p = 0.04)

SMD: −0.22; 95%C.I.[−0.35; −0.1] P value for common effect= 6e−04

SMD: −0.27; 95%C.I.[−0.44; −0.09] P value for random effect= 0.0035

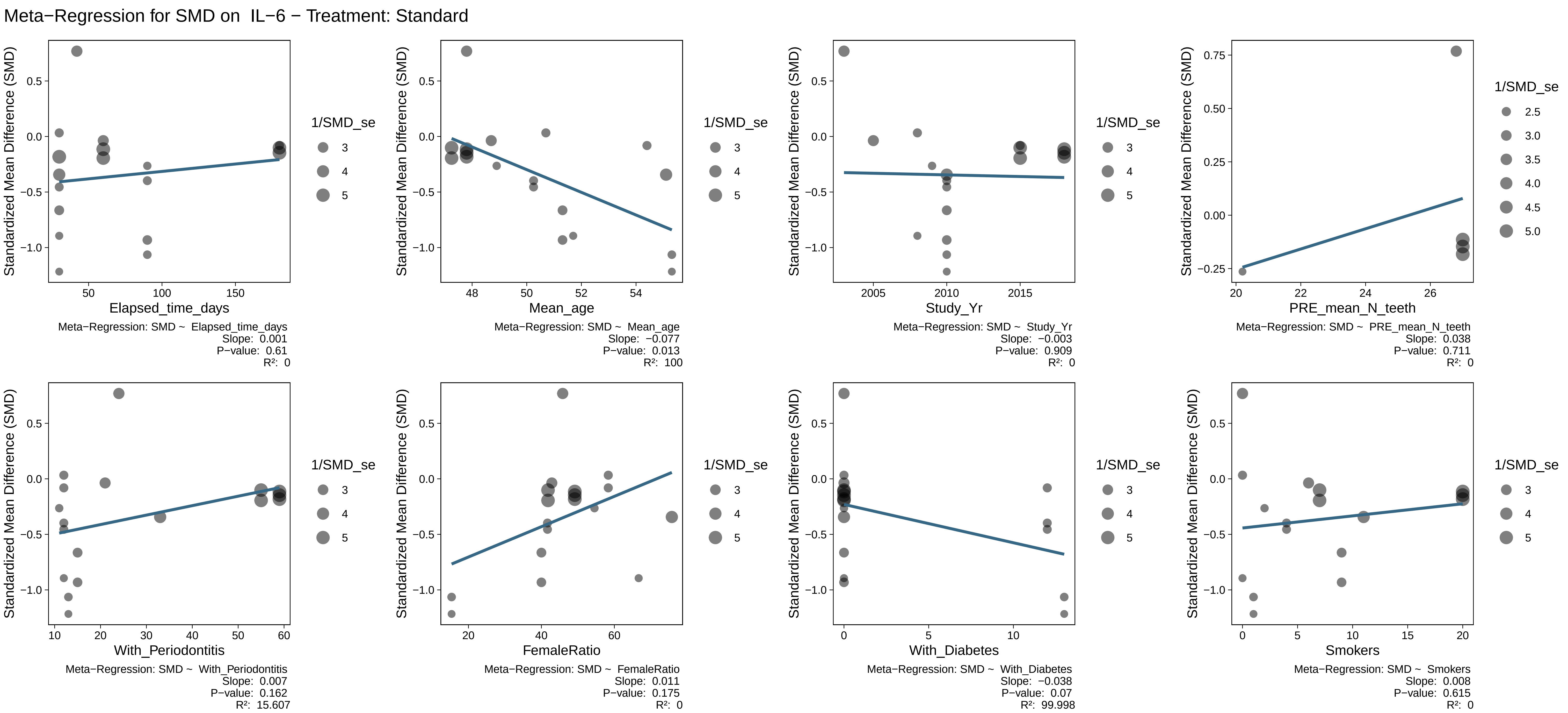
Meta−Regression for SMD on IL−6 − Treatment: Standard
Standardized Mean Difference (SMD)
1/SMD_se
Elapsed_time_days
Meta−Regression: SMD ~ Elapsed_time_days
Slope: 0.001
P−value: 0.61
R²: 0
Mean_age
Meta−Regression: SMD ~ Mean_age
Slope: −0.077
P−value: 0.013
R²: 100
Study_Yr
Meta−Regression: SMD ~ Study_Yr
Slope: −0.003
P−value: 0.909
R²: 0
PRE_mean_N_teeth
Meta−Regression: SMD ~ PRE_mean_N_teeth
Slope: 0.038
P−value: 0.711
R²: 0
With_Periodontitis
Meta−Regression: SMD ~ With_Periodontitis
Slope: 0.007
P−value: 0.162
R²: 15.607
FemaleRatio
Meta−Regression: SMD ~ FemaleRatio
Slope: 0.011
P−value: 0.175
R²: 0
With_Diabetes
Meta−Regression: SMD ~ With_Diabetes
Slope: −0.038
P−value: 0.07
R²: 99.998
Smokers
Meta−Regression: SMD ~ Smokers
Slope: 0.008
P−value: 0.615
R²: 0
