# Supplementary material for: Temporal dynamics of early inflammatory markers after professional dental cleaning: a meta-analysis and spline-based meta-regression of TNF-α, IL-1β, IL-6, and (hs)CRP
Source: Front Immunol. 2025 Aug 28;16:1634622. doi: 10.3389/fimmu.2025.1634622 (PMC12423065; doi:10.3389/fimmu.2025.1634622)
Supplement: Supplementary file 1 [file DataSheet1.zip › Supplementary materials/PDF/TNF-a_Intensive_results.pdf]

Cytokine: TNF−a − Treatment: Intensive

Study	After TRT Total	After TRT Mean	After TRT SD	Before TRT Total	Before TRT Mean	Before TRT SD	SMD	95%−CI	Weight (common)	Weight (random)
**Days after treatment:21**										
Fiorini T 2012	27	1.00	0.0700	27	1.00	0.1000	0.00	[−0.53; 0.53]	3.2%	4.4%
**Days after treatment:30**										
de Sousa Rabelo 2020	15	1.79	6.4900	15	2.24	3.3400	−0.08	[−0.80; 0.63]	1.8%	4.2%
de Sousa Rabelo 2020	15	0.81	1.8700	15	0.40	0.8380	0.28	[−0.44; 1.00]	1.8%	4.2%
de Sousa Rabelo 2020	15	0.57	1.5295	15	0.44	0.8180	0.10	[−0.61; 0.82]	1.8%	4.2%
Anwar Mohammad 2020	30	7.37	0.6520	30	10.56	0.7130	−4.60	[−5.59; −3.61]	0.9%	3.8%
Anwar Mohammad 2020	30	8.48	0.4530	30	10.52	0.7170	−3.35	[−4.15; −2.55]	1.4%	4.1%
**Common effect model**	**105**			**105**			**−1.11**	**[−1.46; −0.77]**	**7.7%**	**.**
**Random effects model**							**−1.52**	**[−3.50; 0.47]**	**.**	**20.5%**
Heterogeneity: $I^2 = 96\%$, $\tau^2 = 4.9739$, $p < 0.01$										
**Days after treatment:35**										
Escobar Arregoces 2021	19	14.29	1.0500	19	15.84	1.0600	−1.44	[−2.16; −0.72]	1.8%	4.2%
**Days after treatment:42**										
Fang F 2015	48	3.27	1.4700	48	3.71	1.7700	−0.27	[−0.67; 0.13]	5.7%	4.6%
Balci Yuce 2016	18	4.48	0.8900	18	7.92	11.6700	−0.41	[−1.07; 0.25]	2.1%	4.3%
**Common effect model**	**66**			**66**			**−0.31**	**[−0.65; 0.04]**	**7.8%**	**.**
**Random effects model**							**−0.31**	**[−0.65; 0.04]**	**.**	**8.8%**
Heterogeneity: $I^2 = 0\%$, $\tau^2 = 0$, $p = 0.73$										
**Days after treatment:45**										
Lei Chen 2012	42	10.12	3.3000	42	11.19	3.9700	−0.29	[−0.72; 0.14]	4.9%	4.5%
**Days after treatment:56**										
Pradeep Koppolu 2013	20	0.20	0.0161	20	0.22	0.0146	−1.21	[−1.89; −0.53]	2.0%	4.2%
**Days after treatment:60**										
Yong−Wei Fu 2015	54	14.55	5.0700	54	16.94	4.0300	−0.52	[−0.90; −0.13]	6.2%	4.6%
**Days after treatment:90**										
Lei Chen 2012	42	11.37	5.1800	42	11.19	3.9700	0.04	[−0.39; 0.47]	5.0%	4.5%
Umut Altay 2013	22	3860000.00	2140000.0000	22	5830000.00	4830000.0000	−0.52	[−1.12; 0.08]	2.5%	4.3%
Umut Altay 2013	24	3270000.00	870000.0000	24	3870000.00	1500000.0000	−0.48	[−1.06; 0.09]	2.8%	4.4%
Fang F 2015	48	3.67	1.7000	48	3.71	1.7700	−0.02	[−0.42; 0.38]	5.7%	4.6%
Wang 2017	19	17.94	7.9410	19	25.59	14.2650	−0.65	[−1.30; 0.01]	2.1%	4.3%
**Common effect model**	**155**			**155**			**−0.22**	**[−0.44; 0.01]**	**18.1%**	**.**
**Random effects model**							**−0.25**	**[−0.53; 0.03]**	**.**	**22.1%**
Heterogeneity: $I^2 = 30\%$, $\tau^2 = 0.0321$, $p = 0.22$										
**Days after treatment:180**										
Poliana Mendes Duarte 2010	14	1.16	0.7100	14	1.15	0.4500	0.02	[−0.72; 0.76]	1.7%	4.2%
Lei Chen 2012	42	10.78	2.9700	42	11.19	3.9700	−0.12	[−0.54; 0.31]	5.0%	4.5%
Fang F 2015	48	3.53	1.3100	48	3.71	1.7700	−0.11	[−0.52; 0.29]	5.7%	4.6%
Hilana Paula Carillo ARTESE 2015	12	6200.00	2000.0000	12	6000.00	1800.0000	0.10	[−0.70; 0.90]	1.4%	4.1%
Geisinger 2016	240	1.91	1.2900	240	1.91	2.4500	0.00	[−0.18; 0.18]	28.6%	4.7%
Yong−Wei Fu 2015	54	13.65	4.6300	54	16.94	4.0300	−0.75	[−1.14; −0.36]	6.0%	4.6%
**Common effect model**	**410**			**410**			**−0.12**	**[−0.25; 0.02]**	**48.3%**	**.**
**Random effects model**							**−0.17**	**[−0.44; 0.10]**	**.**	**26.6%**
Heterogeneity: $I^2 = 59\%$, $\tau^2 = 0.0604$, $p = 0.03$										
**Common effect model**	**898**			**898**			**−0.30**	**[−0.40; −0.20]**	**100.0%**	**.**
**Random effects model**							**−0.59**	**[−1.02; −0.16]**	**.**	**100.0%**

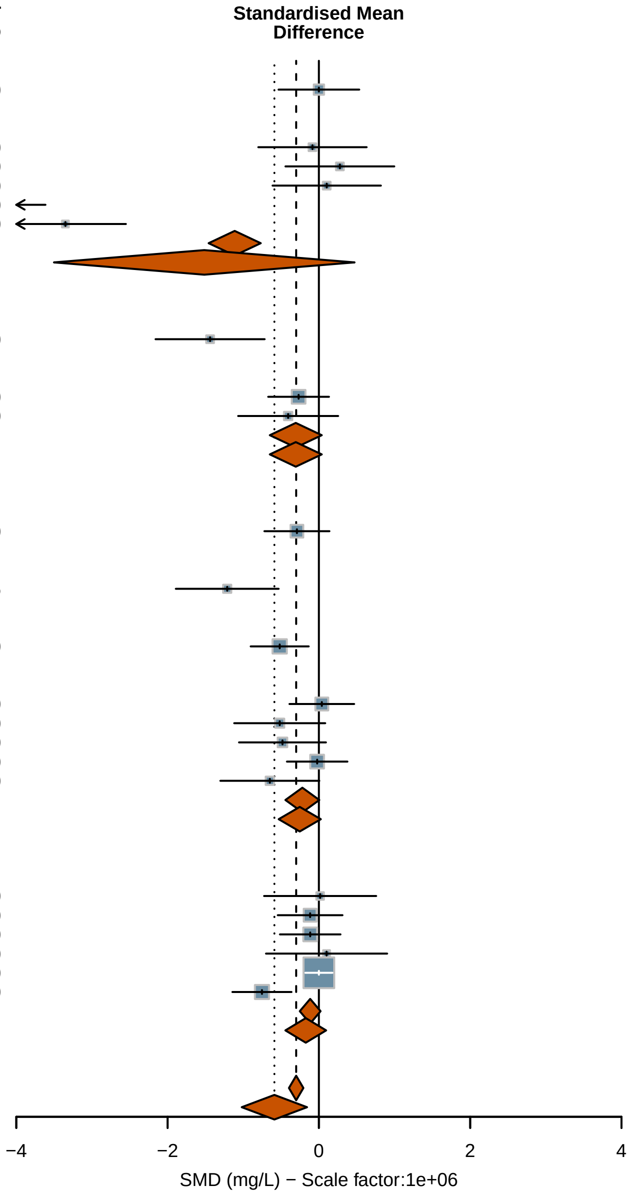

Heterogeneity: $I^2 = 88\%$, $\tau^2 = 1.0221$, $p < 0.01$
Test for subgroup differences (common effect): $\chi^2_8 = 47.76$, df = 8 ($p < 0.01$)
Test for subgroup differences (random effects): $\chi^2_8 = 20.88$, df = 8 ($p < 0.01$)

SMD: −0.3; 95%C.I.[−0.4; −0.2] P value for common effect= 0

SMD: −0.59; 95%C.I.[−1.02; −0.16] P value for random effect= 0.0076

Cytokine: TNF−a − Treatment: Intensive

Study	After TRT Total	After TRT Mean	After TRT SD	Before TRT Total	Before TRT Mean	Before TRT SD	SMD	95%−CI	Weight (common)	Weight (random)
**MonthsAfterTRT:4−6Wks**										
Fiorini T 2012	27	1.00	0.0700	27	1.00	0.1000	0.00	[−0.53; 0.53]	3.2%	4.4%
Fang F 2015	48	3.27	1.4700	48	3.71	1.7700	−0.27	[−0.67; 0.13]	5.7%	4.6%
Escobar Arregoces 2021	19	14.29	1.0500	19	15.84	1.0600	−1.44	[−2.16; −0.72]	1.8%	4.2%
de Sousa Rabelo 2020	15	1.79	6.4900	15	2.24	3.3400	−0.08	[−0.80; 0.63]	1.8%	4.2%
de Sousa Rabelo 2020	15	0.81	1.8700	15	0.40	0.8380	0.28	[−0.44; 1.00]	1.8%	4.2%
de Sousa Rabelo 2020	15	0.57	1.5295	15	0.44	0.8180	0.10	[−0.61; 0.82]	1.8%	4.2%
Anwar Mohammad 2020	30	7.37	0.6520	30	10.56	0.7130	−4.60	[−5.59; −3.61]	0.9%	3.8%
Anwar Mohammad 2020	30	8.48	0.4530	30	10.52	0.7170	−3.35	[−4.15; −2.55]	1.4%	4.1%
Balci Yuce 2016	18	4.48	0.8900	18	7.92	11.6700	−0.41	[−1.07; 0.25]	2.1%	4.3%
**Common effect model**	**217**			**217**			**−0.66**	**[−0.87; −0.45]**	**20.4%**	**.**
**Random effects model**							**−1.06**	**[−2.17; 0.05]**	**.**	**37.9%**
Heterogeneity: $I^2 = 94\%$, $\tau^2 = 2.7652$, $p < 0.01$										
**MonthsAfterTRT:Within 3 Mo.**										
Lei Chen 2012	42	10.12	3.3000	42	11.19	3.9700	−0.29	[−0.72; 0.14]	4.9%	4.5%
Lei Chen 2012	42	11.37	5.1800	42	11.19	3.9700	0.04	[−0.39; 0.47]	5.0%	4.5%
Umut Altay 2013	22	3860000.00	2140000.0000	22	5830000.00	4830000.0000	−0.52	[−1.12; 0.08]	2.5%	4.3%
Umut Altay 2013	24	3270000.00	870000.0000	24	3870000.00	1500000.0000	−0.48	[−1.06; 0.09]	2.8%	4.4%
Pradeep Koppolu 2013	20	0.20	0.0161	20	0.22	0.0146	−1.21	[−1.89; −0.53]	2.0%	4.2%
Fang F 2015	48	3.67	1.7000	48	3.71	1.7700	−0.02	[−0.42; 0.38]	5.7%	4.6%
Wang 2017	19	17.94	7.9410	19	25.59	14.2650	−0.65	[−1.30; 0.01]	2.1%	4.3%
Yong−Wei Fu 2015	54	14.55	5.0700	54	16.94	4.0300	−0.52	[−0.90; −0.13]	6.2%	4.6%
**Common effect model**	**271**			**271**			**−0.35**	**[−0.52; −0.18]**	**31.3%**	**.**
**Random effects model**							**−0.40**	**[−0.64; −0.15]**	**.**	**35.5%**
Heterogeneity: $I^2 = 50\%$, $\tau^2 = 0.0622$, $p = 0.05$										
**MonthsAfterTRT:Within 6 Mo.**										
Poliana Mendes Duarte 2010	14	1.16	0.7100	14	1.15	0.4500	0.02	[−0.72; 0.76]	1.7%	4.2%
Lei Chen 2012	42	10.78	2.9700	42	11.19	3.9700	−0.12	[−0.54; 0.31]	5.0%	4.5%
Fang F 2015	48	3.53	1.3100	48	3.71	1.7700	−0.11	[−0.52; 0.29]	5.7%	4.6%
Hilana Paula Carillo ARTESE 2015	12	6200.00	2000.0000	12	6000.00	1800.0000	0.10	[−0.70; 0.90]	1.4%	4.1%
Geisinger 2016	240	1.91	1.2900	240	1.91	2.4500	0.00	[−0.18; 0.18]	28.6%	4.7%
Yong−Wei Fu 2015	54	13.65	4.6300	54	16.94	4.0300	−0.75	[−1.14; −0.36]	6.0%	4.6%
**Common effect model**	**410**			**410**			**−0.12**	**[−0.25; 0.02]**	**48.3%**	**.**
**Random effects model**							**−0.17**	**[−0.44; 0.10]**	**.**	**26.6%**
Heterogeneity: $I^2 = 59\%$, $\tau^2 = 0.0604$, $p = 0.03$										
**Common effect model**	**898**			**898**			**−0.30**	**[−0.40; −0.20]**	**100.0%**	**.**
**Random effects model**							**−0.59**	**[−1.02; −0.16]**	**.**	**100.0%**

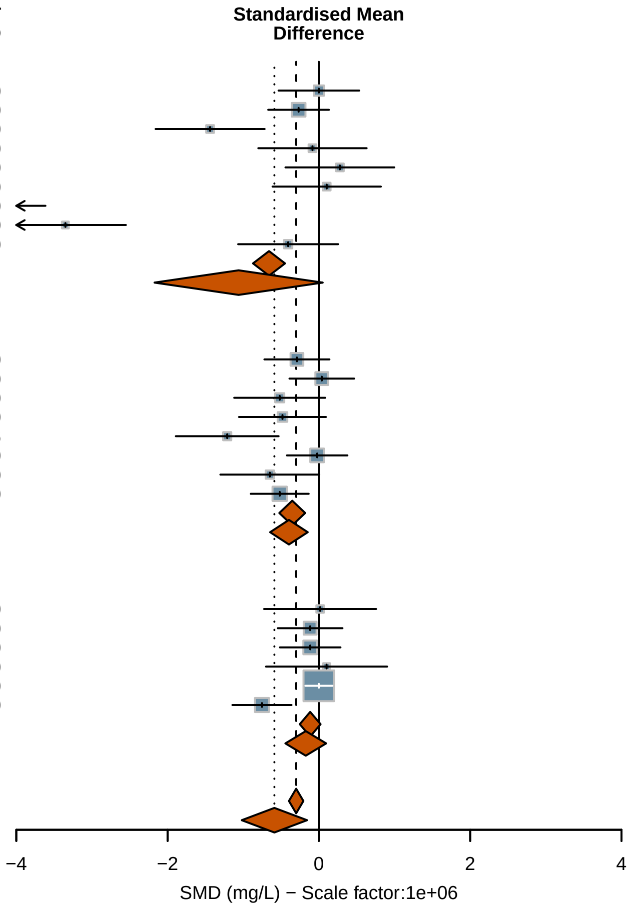

Heterogeneity: $I^2 = 88\%$, $\tau^2 = 1.0221$, $p < 0.01$
Test for subgroup differences (common effect): $\chi^2_2 = 18.37$, df = 2 ($p < 0.01$)
Test for subgroup differences (random effects): $\chi^2_2 = 3.19$, df = 2 ($p = 0.20$)

SMD: −0.3; 95%C.I.[−0.4; −0.2] P value for common effect= 0

SMD: −0.59; 95%C.I.[−1.02; −0.16] P value for random effect= 0.0076

Cytokine: TNF−a − Treatment: Intensive

Study	After TRT Total	After TRT Mean	After TRT SD	Before TRT Total	Before TRT Mean	Before TRT SD	SMD	95%−CI	Weight (common)	Weight (random)
**Assay type:ELISA**										
Poliana Mendes Duarte 2010	14	1.16	0.7100	14	1.15	0.4500	0.02	[−0.72; 0.76]	1.7%	4.2%
Lei Chen 2012	42	10.12	3.3000	42	11.19	3.9700	−0.29	[−0.72; 0.14]	4.9%	4.5%
Lei Chen 2012	42	11.37	5.1800	42	11.19	3.9700	0.04	[−0.39; 0.47]	5.0%	4.5%
Lei Chen 2012	42	10.78	2.9700	42	11.19	3.9700	−0.12	[−0.54; 0.31]	5.0%	4.5%
Umut Altay 2013	22	3860000.00	2140000.0000	22	5830000.00	4830000.0000	−0.52	[−1.12; 0.08]	2.5%	4.3%
Umut Altay 2013	24	3270000.00	870000.0000	24	3870000.00	1500000.0000	−0.48	[−1.06; 0.09]	2.8%	4.4%
Pradeep Koppolu 2013	20	0.20	0.0161	20	0.22	0.0146	−1.21	[−1.89; −0.53]	2.0%	4.2%
Hilana Paula Carillo ARTESE 2015	12	6200.00	2000.0000	12	6000.00	1800.0000	0.10	[−0.70; 0.90]	1.4%	4.1%
Anwar Mohammad 2020	30	7.37	0.6520	30	10.56	0.7130	−4.60	[−5.59; −3.61]	0.9%	3.8%
Anwar Mohammad 2020	30	8.48	0.4530	30	10.52	0.7170	−3.35	[−4.15; −2.55]	1.4%	4.1%
Wang 2017	19	17.94	7.9410	19	25.59	14.2650	−0.65	[−1.30; 0.01]	2.1%	4.3%
Balci Yuce 2016	18	4.48	0.8900	18	7.92	11.6700	−0.41	[−1.07; 0.25]	2.1%	4.3%
Geisinger 2016	240	1.91	1.2900	240	1.91	2.4500	0.00	[−0.18; 0.18]	28.6%	4.7%
Yong−Wei Fu 2015	54	14.55	5.0700	54	16.94	4.0300	−0.52	[−0.90; −0.13]	6.2%	4.6%
Yong−Wei Fu 2015	54	13.65	4.6300	54	16.94	4.0300	−0.75	[−1.14; −0.36]	6.0%	4.6%
**Common effect model**	**663**			**663**			**−0.35**	**[−0.47; −0.24]**	**72.6%**	**.**
**Random effects model**							**−0.81**	**[−1.45; −0.18]**	**.**	**65.1%**
Heterogeneity: $I^2 = 91\%$, $\tau^2 = 1.4868$, $p < 0.01$										
**Assay type:flow cytometry**										
Fiorini T 2012	27	1.00	0.0700	27	1.00	0.1000	0.00	[−0.53; 0.53]	3.2%	4.4%
**Assay type:immunosorbent assay**										
Fang F 2015	48	3.27	1.4700	48	3.71	1.7700	−0.27	[−0.67; 0.13]	5.7%	4.6%
Fang F 2015	48	3.67	1.7000	48	3.71	1.7700	−0.02	[−0.42; 0.38]	5.7%	4.6%
Fang F 2015	48	3.53	1.3100	48	3.71	1.7700	−0.11	[−0.52; 0.29]	5.7%	4.6%
**Common effect model**	**144**			**144**			**−0.13**	**[−0.37; 0.10]**	**17.1%**	**.**
**Random effects model**							**−0.13**	**[−0.37; 0.10]**	**.**	**13.7%**
Heterogeneity: $I^2 = 0\%$, $\tau^2 = 0$, $p = 0.69$										
**Assay type:Milliplex Inflammation Human Cytokine kit**										
Escobar Arregoces 2021	19	14.29	1.0500	19	15.84	1.0600	−1.44	[−2.16; −0.72]	1.8%	4.2%
**Assay type:ultrasensitive multiplex assay**										
de Sousa Rabelo 2020	15	1.79	6.4900	15	2.24	3.3400	−0.08	[−0.80; 0.63]	1.8%	4.2%
de Sousa Rabelo 2020	15	0.81	1.8700	15	0.40	0.8380	0.28	[−0.44; 1.00]	1.8%	4.2%
de Sousa Rabelo 2020	15	0.57	1.5295	15	0.44	0.8180	0.10	[−0.61; 0.82]	1.8%	4.2%
**Common effect model**	**45**			**45**			**0.10**	**[−0.32; 0.51]**	**5.3%**	**.**
**Random effects model**							**0.10**	**[−0.32; 0.51]**	**.**	**12.6%**
Heterogeneity: $I^2 = 0\%$, $\tau^2 = 0$, $p = 0.78$										
**Common effect model**	**898**			**898**			**−0.30**	**[−0.40; −0.20]**	**100.0%**	**.**
**Random effects model**							**−0.59**	**[−1.02; −0.16]**	**.**	**100.0%**

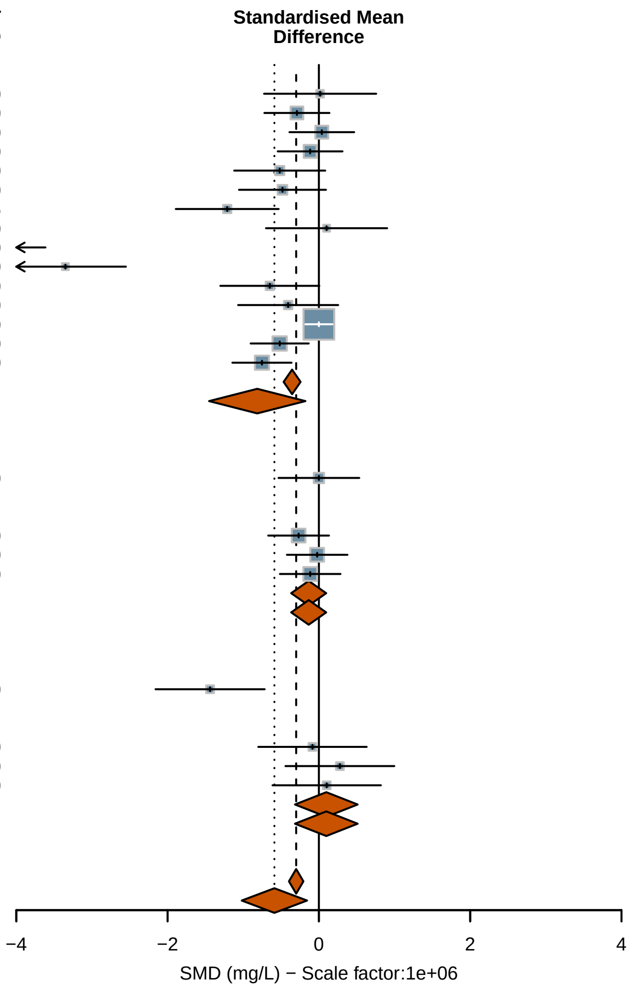

Heterogeneity: $I^2 = 88\%$, $\tau^2 = 1.0221$, $p < 0.01$
Test for subgroup differences (common effect): $\chi^2_4 = 17.18$, df = 4 ($p < 0.01$)
Test for subgroup differences (random effects): $\chi^2_4 = 17.73$, df = 4 ($p < 0.01$)

SMD: −0.3; 95%C.I.[−0.4; −0.2] P value for common effect= 0

SMD: −0.59; 95%C.I.[−1.02; −0.16] P value for random effect= 0.0076

Cytokine: TNF−a − Treatment: Intensive

Study	After TRT Total	After TRT Mean	After TRT SD	Before TRT Total	Before TRT Mean	Before TRT SD	SMD	95%−CI	Weight (common)	Weight (random)
**Bias judgment:High risk**										
Umut Altay 2013	22	3860000.00	2140000.0000	22	5830000.00	4830000.0000	−0.52	[−1.12; 0.08]	2.5%	4.3%
Umut Altay 2013	24	3270000.00	870000.0000	24	3870000.00	1500000.0000	−0.48	[−1.06; 0.09]	2.8%	4.4%
Fiorini T 2012	27	1.00	0.0700	27	1.00	0.1000	0.00	[−0.53; 0.53]	3.2%	4.4%
Pradeep Koppolu 2013	20	0.20	0.0161	20	0.22	0.0146	−1.21	[−1.89; −0.53]	2.0%	4.2%
Hilana Paula Carillo ARTESE 2015	12	6200.00	2000.0000	12	6000.00	1800.0000	0.10	[−0.70; 0.90]	1.4%	4.1%
Escobar Arregoces 2021	19	14.29	1.0500	19	15.84	1.0600	−1.44	[−2.16; −0.72]	1.8%	4.2%
de Sousa Rabelo 2020	15	1.79	6.4900	15	2.24	3.3400	−0.08	[−0.80; 0.63]	1.8%	4.2%
de Sousa Rabelo 2020	15	0.81	1.8700	15	0.40	0.8380	0.28	[−0.44; 1.00]	1.8%	4.2%
de Sousa Rabelo 2020	15	0.57	1.5295	15	0.44	0.8180	0.10	[−0.61; 0.82]	1.8%	4.2%
Balci Yuce 2016	18	4.48	0.8900	18	7.92	11.6700	−0.41	[−1.07; 0.25]	2.1%	4.3%
**Common effect model**	**187**			**187**			**−0.37**	**[−0.58; −0.16]**	**21.1%**	**.**
**Random effects model**							**−0.37**	**[−0.71; −0.03]**	**.**	**42.5%**
Heterogeneity: $I^2 = 61\%$, $\tau^2 = 0.1887$, $p < 0.01$										
**Bias judgment:Low risk**										
Poliana Mendes Duarte 2010	14	1.16	0.7100	14	1.15	0.4500	0.02	[−0.72; 0.76]	1.7%	4.2%
Fang F 2015	48	3.27	1.4700	48	3.71	1.7700	−0.27	[−0.67; 0.13]	5.7%	4.6%
Fang F 2015	48	3.67	1.7000	48	3.71	1.7700	−0.02	[−0.42; 0.38]	5.7%	4.6%
Fang F 2015	48	3.53	1.3100	48	3.71	1.7700	−0.11	[−0.52; 0.29]	5.7%	4.6%
Anwar Mohammad 2020	30	7.37	0.6520	30	10.56	0.7130	−4.60	[−5.59; −3.61]	0.9%	3.8%
Anwar Mohammad 2020	30	8.48	0.4530	30	10.52	0.7170	−3.35	[−4.15; −2.55]	1.4%	4.1%
Yong−Wei Fu 2015	54	14.55	5.0700	54	16.94	4.0300	−0.52	[−0.90; −0.13]	6.2%	4.6%
Yong−Wei Fu 2015	54	13.65	4.6300	54	16.94	4.0300	−0.75	[−1.14; −0.36]	6.0%	4.6%
**Common effect model**	**326**			**326**			**−0.57**	**[−0.74; −0.41]**	**33.3%**	**.**
**Random effects model**							**−1.17**	**[−2.35; 0.02]**	**.**	**34.9%**
Heterogeneity: $I^2 = 95\%$, $\tau^2 = 2.8301$, $p < 0.01$										
**Bias judgment:Some concerns**										
Lei Chen 2012	42	10.12	3.3000	42	11.19	3.9700	−0.29	[−0.72; 0.14]	4.9%	4.5%
Lei Chen 2012	42	11.37	5.1800	42	11.19	3.9700	0.04	[−0.39; 0.47]	5.0%	4.5%
Lei Chen 2012	42	10.78	2.9700	42	11.19	3.9700	−0.12	[−0.54; 0.31]	5.0%	4.5%
Wang 2017	19	17.94	7.9410	19	25.59	14.2650	−0.65	[−1.30; 0.01]	2.1%	4.3%
Geisinger 2016	240	1.91	1.2900	240	1.91	2.4500	0.00	[−0.18; 0.18]	28.6%	4.7%
**Common effect model**	**385**			**385**			**−0.07**	**[−0.21; 0.07]**	**45.6%**	**.**
**Random effects model**							**−0.08**	**[−0.24; 0.08]**	**.**	**22.6%**
Heterogeneity: $I^2 = 18\%$, $\tau^2 = 0.0036$, $p = 0.30$										
**Common effect model**	**898**			**898**			**−0.30**	**[−0.40; −0.20]**	**100.0%**	**.**
**Random effects model**							**−0.59**	**[−1.02; −0.16]**	**.**	**100.0%**

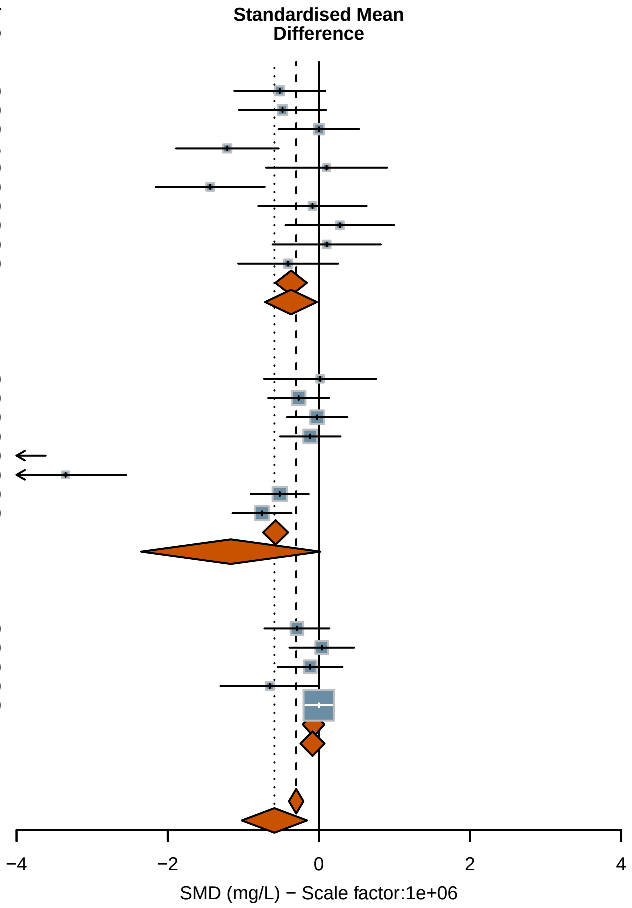

Heterogeneity: $I^2 = 88\%$, $\tau^2 = 1.0221$, $p < 0.01$
Test for subgroup differences (common effect): $\chi^2_2 = 20.96$, df = 2 ($p < 0.01$)
Test for subgroup differences (random effects): $\chi^2_2 = 5.04$, df = 2 ($p = 0.08$)

SMD: −0.3; 95%C.I.[−0.4; −0.2] P value for common effect= 0

SMD: −0.59; 95%C.I.[−1.02; −0.16] P value for random effect= 0.0076

Cytokine: TNF−a − Treatment: Intensive

Study	After TRT Total	After TRT Mean	After TRT SD	Before TRT Total	Before TRT Mean	Before TRT SD	SMD	95%−CI	Weight (common)	Weight (random)
**Study design:Clinical trial**										
de Sousa Rabelo 2020	15	1.79	6.4900	15	2.24	3.3400	−0.08	[−0.80; 0.63]	1.8%	4.2%
de Sousa Rabelo 2020	15	0.81	1.8700	15	0.40	0.8380	0.28	[−0.44; 1.00]	1.8%	4.2%
de Sousa Rabelo 2020	15	0.57	1.5295	15	0.44	0.8180	0.10	[−0.61; 0.82]	1.8%	4.2%
**Common effect model**	**45**			**45**			**0.10**	**[−0.32; 0.51]**	**5.3%**	**.**
**Random effects model**							**0.10**	**[−0.32; 0.51]**	**.**	**12.6%**
Heterogeneity: $I^2 = 0\%$, $\tau^2 = 0$, $p = 0.78$										
**Study design:Comparative study**										
Poliana Mendes Duarte 2010	14	1.16	0.7100	14	1.15	0.4500	0.02	[−0.72; 0.76]	1.7%	4.2%
Umut Altay 2013	22	3860000.00	2140000.0000	22	5830000.00	4830000.0000	−0.52	[−1.12; 0.08]	2.5%	4.3%
Umut Altay 2013	24	3270000.00	870000.0000	24	3870000.00	1500000.0000	−0.48	[−1.06; 0.09]	2.8%	4.4%
Anwar Mohammad 2020	30	7.37	0.6520	30	10.56	0.7130	−4.60	[−5.59; −3.61]	0.9%	3.8%
Anwar Mohammad 2020	30	8.48	0.4530	30	10.52	0.7170	−3.35	[−4.15; −2.55]	1.4%	4.1%
**Common effect model**	**120**			**120**			**−1.25**	**[−1.57; −0.94]**	**9.3%**	**.**
**Random effects model**							**−1.76**	**[−3.55; 0.02]**	**.**	**20.8%**
Heterogeneity: $I^2 = 96\%$, $\tau^2 = 4.0098$, $p < 0.01$										
**Study design:Observational study**										
Escobar Arregoces 2021	19	14.29	1.0500	19	15.84	1.0600	−1.44	[−2.16; −0.72]	1.8%	4.2%
**Study design:RCT**										
Lei Chen 2012	42	10.12	3.3000	42	11.19	3.9700	−0.29	[−0.72; 0.14]	4.9%	4.5%
Lei Chen 2012	42	11.37	5.1800	42	11.19	3.9700	0.04	[−0.39; 0.47]	5.0%	4.5%
Lei Chen 2012	42	10.78	2.9700	42	11.19	3.9700	−0.12	[−0.54; 0.31]	5.0%	4.5%
Fiorini T 2012	27	1.00	0.0700	27	1.00	0.1000	0.00	[−0.53; 0.53]	3.2%	4.4%
Pradeep Koppolu 2013	20	0.20	0.0161	20	0.22	0.0146	−1.21	[−1.89; −0.53]	2.0%	4.2%
Fang F 2015	48	3.27	1.4700	48	3.71	1.7700	−0.27	[−0.67; 0.13]	5.7%	4.6%
Fang F 2015	48	3.67	1.7000	48	3.71	1.7700	−0.02	[−0.42; 0.38]	5.7%	4.6%
Fang F 2015	48	3.53	1.3100	48	3.71	1.7700	−0.11	[−0.52; 0.29]	5.7%	4.6%
Hilana Paula Carillo ARTESE 2015	12	6200.00	2000.0000	12	6000.00	1800.0000	0.10	[−0.70; 0.90]	1.4%	4.1%
Wang 2017	19	17.94	7.9410	19	25.59	14.2650	−0.65	[−1.30; 0.01]	2.1%	4.3%
Balci Yuce 2016	18	4.48	0.8900	18	7.92	11.6700	−0.41	[−1.07; 0.25]	2.1%	4.3%
Geisinger 2016	240	1.91	1.2900	240	1.91	2.4500	0.00	[−0.18; 0.18]	28.6%	4.7%
Yong−Wei Fu 2015	54	14.55	5.0700	54	16.94	4.0300	−0.52	[−0.90; −0.13]	6.2%	4.6%
Yong−Wei Fu 2015	54	13.65	4.6300	54	16.94	4.0300	−0.75	[−1.14; −0.36]	6.0%	4.6%
**Common effect model**	**714**			**714**			**−0.20**	**[−0.30; −0.09]**	**83.6%**	**.**
**Random effects model**							**−0.27**	**[−0.44; −0.10]**	**.**	**62.4%**
Heterogeneity: $I^2 = 56\%$, $\tau^2 = 0.0514$, $p < 0.01$										
**Common effect model**	**898**			**898**			**−0.30**	**[−0.40; −0.20]**	**100.0%**	**.**
**Random effects model**							**−0.59**	**[−1.02; −0.16]**	**.**	**100.0%**

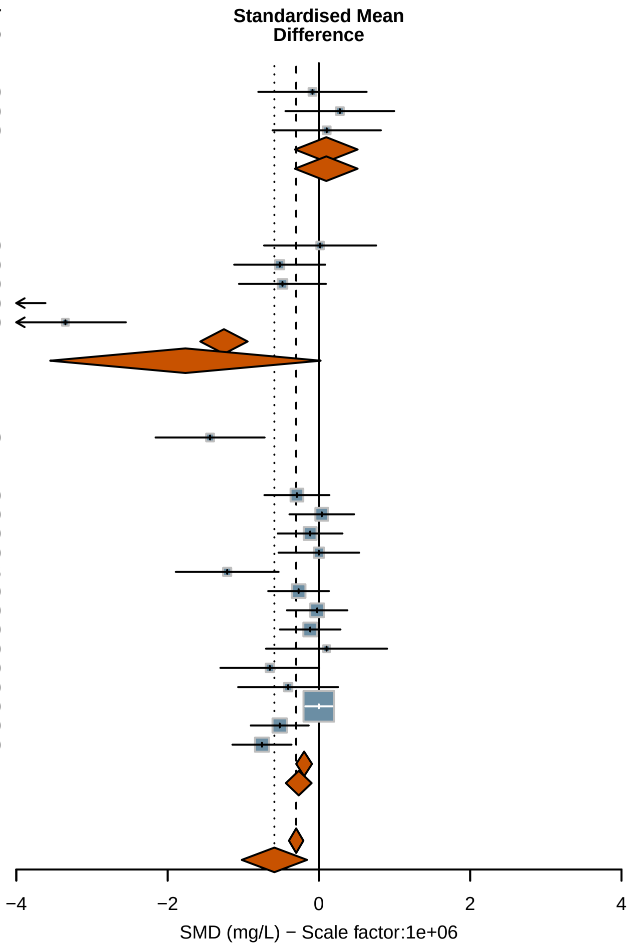

Heterogeneity: $I^2 = 88\%$, $\tau^2 = 1.0221$, $p < 0.01$
Test for subgroup differences (common effect): $\chi^2_3 = 52.68$, df = 3 ($p < 0.01$)
Test for subgroup differences (random effects): $\chi^2_3 = 15.78$, df = 3 ($p < 0.01$)

SMD: −0.3; 95%C.I.[−0.4; −0.2] P value for common effect= 0

SMD: −0.59; 95%C.I.[−1.02; −0.16] P value for random effect= 0.0076

Cytokine: TNF−a − Treatment: Intensive

Study	After TRT Total	After TRT Mean	After TRT SD	Before TRT Total	Before TRT Mean	Before TRT SD	SMD	95%−CI	Weight (common)	Weight (random)
**Additional_devices:curcumin gel**										
Anwar Mohammad 2020	30	7.37	0.6520	30	10.56	0.7130	−4.60	[−5.59; −3.61]	7.1%	32.4%
**Additional_devices:Protocollo full mouth disinfection**										
Yong−Wei Fu 2015	54	14.55	5.0700	54	16.94	4.0300	−0.52	[−0.90; −0.13]	47.3%	33.8%
Yong−Wei Fu 2015	54	13.65	4.6300	54	16.94	4.0300	−0.75	[−1.14; −0.36]	45.6%	33.8%
**Common effect model**	**108**			**108**			**−0.63**	**[−0.91; −0.36]**	**92.9%**	**.**
**Random effects model**							**−0.63**	**[−0.91; −0.36]**	**.**	**67.6%**
Heterogeneity: I^2 = 0%, τ^2 = 0, p = 0.40										
**Common effect model**	**138**			**138**			**−0.92**	**[−1.18; −0.65]**	**100.0%**	**.**
**Random effects model**							**−1.92**	**[−4.48; 0.64]**	**.**	**100.0%**

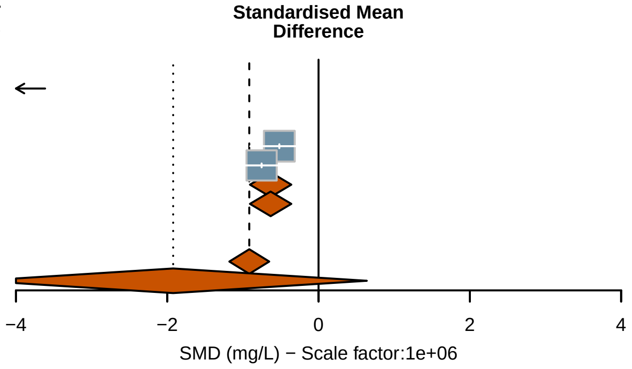

Heterogeneity: I^2 = 97%, τ^2 = 5.0082, p < 0.01
Test for subgroup differences (common effect): χ^2_1 = 57.42, df = 1 (p < 0.01)
Test for subgroup differences (random effects): χ^2_1 = 57.42, df = 1 (p < 0.01)

SMD: −0.92; 95%C.I.[−1.18; −0.65] P value for common effect= 0

SMD: −1.92; 95%C.I.[−4.48; 0.64] P value for random effect= 0.1412

Cytokine: TNF−a − Treatment: Intensive

Study	After TRT Total	After TRT Mean	After TRT SD	Before TRT Total	Before TRT Mean	Before TRT SD	SMD	95%−CI	Weight (common)	Weight (random)
**Female_binary:Only Female**										
Fiorini T 2012	27	1.00	0.0700	27	1.00	0.1000	0.00	[−0.53; 0.53]	3.2%	4.4%
**Female_binary:Sex mixed**										
Poliana Mendes Duarte 2010	14	1.16	0.7100	14	1.15	0.4500	0.02	[−0.72; 0.76]	1.7%	4.2%
Lei Chen 2012	42	10.12	3.3000	42	11.19	3.9700	−0.29	[−0.72; 0.14]	4.9%	4.5%
Lei Chen 2012	42	11.37	5.1800	42	11.19	3.9700	0.04	[−0.39; 0.47]	5.0%	4.5%
Lei Chen 2012	42	10.78	2.9700	42	11.19	3.9700	−0.12	[−0.54; 0.31]	5.0%	4.5%
Umut Altay 2013	22	3860000.00	2140000.0000	22	5830000.00	4830000.0000	−0.52	[−1.12; 0.08]	2.5%	4.3%
Umut Altay 2013	24	3270000.00	870000.0000	24	3870000.00	1500000.0000	−0.48	[−1.06; 0.09]	2.8%	4.4%
Pradeep Koppolu 2013	20	0.20	0.0161	20	0.22	0.0146	−1.21	[−1.89; −0.53]	2.0%	4.2%
Fang F 2015	48	3.27	1.4700	48	3.71	1.7700	−0.27	[−0.67; 0.13]	5.7%	4.6%
Fang F 2015	48	3.67	1.7000	48	3.71	1.7700	−0.02	[−0.42; 0.38]	5.7%	4.6%
Fang F 2015	48	3.53	1.3100	48	3.71	1.7700	−0.11	[−0.52; 0.29]	5.7%	4.6%
Hilana Paula Carillo ARTESE 2015	12	6200.00	2000.0000	12	6000.00	1800.0000	0.10	[−0.70; 0.90]	1.4%	4.1%
Escobar Arregoces 2021	19	14.29	1.0500	19	15.84	1.0600	−1.44	[−2.16; −0.72]	1.8%	4.2%
de Sousa Rabelo 2020	15	1.79	6.4900	15	2.24	3.3400	−0.08	[−0.80; 0.63]	1.8%	4.2%
de Sousa Rabelo 2020	15	0.81	1.8700	15	0.40	0.8380	0.28	[−0.44; 1.00]	1.8%	4.2%
de Sousa Rabelo 2020	15	0.57	1.5295	15	0.44	0.8180	0.10	[−0.61; 0.82]	1.8%	4.2%
Anwar Mohammad 2020	30	7.37	0.6520	30	10.56	0.7130	−4.60	[−5.59; −3.61]	0.9%	3.8%
Anwar Mohammad 2020	30	8.48	0.4530	30	10.52	0.7170	−3.35	[−4.15; −2.55]	1.4%	4.1%
Wang 2017	19	17.94	7.9410	19	25.59	14.2650	−0.65	[−1.30; 0.01]	2.1%	4.3%
Balci Yuce 2016	18	4.48	0.8900	18	7.92	11.6700	−0.41	[−1.07; 0.25]	2.1%	4.3%
Geisinger 2016	240	1.91	1.2900	240	1.91	2.4500	0.00	[−0.18; 0.18]	28.6%	4.7%
Yong−Wei Fu 2015	54	14.55	5.0700	54	16.94	4.0300	−0.52	[−0.90; −0.13]	6.2%	4.6%
Yong−Wei Fu 2015	54	13.65	4.6300	54	16.94	4.0300	−0.75	[−1.14; −0.36]	6.0%	4.6%
**Common effect model**	**871**			**871**			**−0.31**	**[−0.41; −0.21]**	**96.8%**	**.**
**Random effects model**							**−0.62**	**[−1.07; −0.17]**	**.**	**95.6%**
Heterogeneity: $I^2 = 88\%$, $\tau^2 = 1.0676$, $p < 0.01$										
**Common effect model**	**898**			**898**			**−0.30**	**[−0.40; −0.20]**	**100.0%**	**.**
**Random effects model**							**−0.59**	**[−1.02; −0.16]**	**.**	**100.0%**

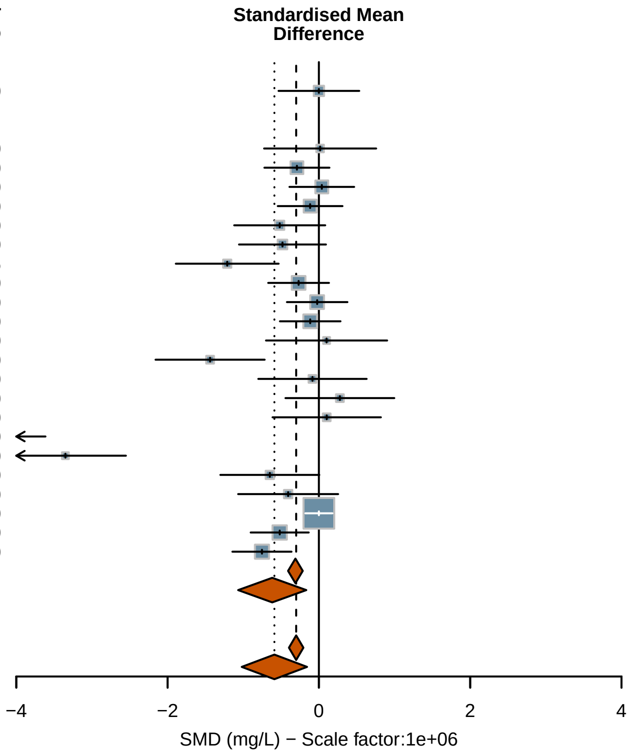

Heterogeneity: $I^2 = 88\%$, $\tau^2 = 1.0221$, $p < 0.01$
Test for subgroup differences (common effect): $\chi^2_1 = 1.26$, df = 1 ($p = 0.26$)
Test for subgroup differences (random effects): $\chi^2_1 = 3.00$, df = 1 ($p = 0.08$)

SMD: −0.3; 95%C.I.[−0.4; −0.2] P value for common effect= 0

SMD: −0.59; 95%C.I.[−1.02; −0.16] P value for random effect= 0.0076

Cytokine: TNF−a − Treatment: Intensive

Study	Total	Mean	After TRT SD	Total	Mean	Before TRT SD	SMD	95%−CI	Weight (common)	Weight (random)
**Smoke_binary:Sample with smokers**										
Lei Chen 2012	42	10.12	3.3000	42	11.19	3.9700	−0.29	[−0.72; 0.14]	5.0%	4.7%
Lei Chen 2012	42	11.37	5.1800	42	11.19	3.9700	0.04	[−0.39; 0.47]	5.1%	4.7%
Lei Chen 2012	42	10.78	2.9700	42	11.19	3.9700	−0.12	[−0.54; 0.31]	5.1%	4.7%
Umut Altay 2013	22	3860000.00	2140000.0000	22	5830000.00	4830000.0000	−0.52	[−1.12; 0.08]	2.6%	4.5%
Umut Altay 2013	24	3270000.00	870000.0000	24	3870000.00	1500000.0000	−0.48	[−1.06; 0.09]	2.8%	4.6%
Fiorini T 2012	27	1.00	0.0700	27	1.00	0.1000	0.00	[−0.53; 0.53]	3.3%	4.6%
Fang F 2015	48	3.27	1.4700	48	3.71	1.7700	−0.27	[−0.67; 0.13]	5.7%	4.7%
Fang F 2015	48	3.67	1.7000	48	3.71	1.7700	−0.02	[−0.42; 0.38]	5.8%	4.7%
Fang F 2015	48	3.53	1.3100	48	3.71	1.7700	−0.11	[−0.52; 0.29]	5.8%	4.7%
Wang 2017	19	17.94	7.9410	19	25.59	14.2650	−0.65	[−1.30; 0.01]	2.2%	4.5%
Balci Yuce 2016	18	4.48	0.8900	18	7.92	11.6700	−0.41	[−1.07; 0.25]	2.1%	4.5%
Geisinger 2016	240	1.91	1.2900	240	1.91	2.4500	0.00	[−0.18; 0.18]	29.0%	4.9%
Yong−Wei Fu 2015	54	14.55	5.0700	54	16.94	4.0300	−0.52	[−0.90; −0.13]	6.3%	4.8%
Yong−Wei Fu 2015	54	13.65	4.6300	54	16.94	4.0300	−0.75	[−1.14; −0.36]	6.1%	4.8%
**Common effect model**	**728**			**728**			**−0.20**	**[−0.30; −0.09]**	**86.7%**	**.**
**Random effects model**							**−0.25**	**[−0.40; −0.10]**	**.**	**65.5%**
Heterogeneity: I^2 = 42%, τ^2 = 0.0311, p = 0.05										
**Smoke_binary:Sample without smokers**										
Poliana Mendes Duarte 2010	14	1.16	0.7100	14	1.15	0.4500	0.02	[−0.72; 0.76]	1.7%	4.3%
Pradeep Koppolu 2013	20	0.20	0.0161	20	0.22	0.0146	−1.21	[−1.89; −0.53]	2.0%	4.4%
Escobar Arregoces 2021	19	14.29	1.0500	19	15.84	1.0600	−1.44	[−2.16; −0.72]	1.8%	4.4%
de Sousa Rabelo 2020	15	1.79	6.4900	15	2.24	3.3400	−0.08	[−0.80; 0.63]	1.8%	4.4%
de Sousa Rabelo 2020	15	0.81	1.8700	15	0.40	0.8380	0.28	[−0.44; 1.00]	1.8%	4.4%
de Sousa Rabelo 2020	15	0.57	1.5295	15	0.44	0.8180	0.10	[−0.61; 0.82]	1.8%	4.4%
Anwar Mohammad 2020	30	7.37	0.6520	30	10.56	0.7130	−4.60	[−5.59; −3.61]	0.9%	4.0%
Anwar Mohammad 2020	30	8.48	0.4530	30	10.52	0.7170	−3.35	[−4.15; −2.55]	1.5%	4.3%
**Common effect model**	**158**			**158**			**−1.03**	**[−1.29; −0.76]**	**13.3%**	**.**
**Random effects model**							**−1.27**	**[−2.50; −0.04]**	**.**	**34.5%**
Heterogeneity: I^2 = 94%, τ^2 = 3.0145, p < 0.01										
**Common effect model**	**886**			**886**			**−0.31**	**[−0.40; −0.21]**	**100.0%**	**.**
**Random effects model**							**−0.62**	**[−1.07; −0.17]**	**.**	**100.0%**

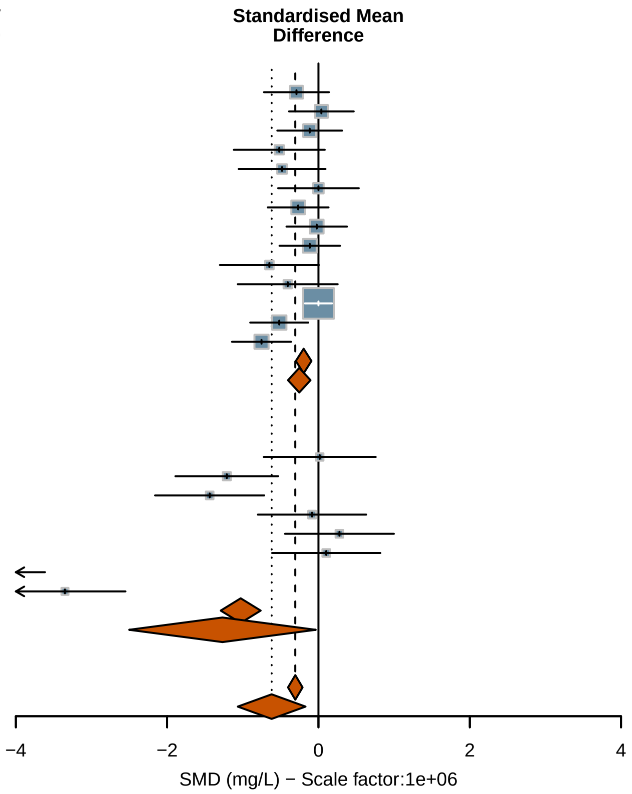

Heterogeneity: I^2 = 88%, τ^2 = 1.0594, p < 0.01
Test for subgroup differences (common effect): χ^2_1 = 33.06, df = 1 (p < 0.01)
Test for subgroup differences (random effects): χ^2_1 = 2.57, df = 1 (p = 0.11)

SMD: −0.31; 95%C.I.[−0.4; −0.21] P value for common effect= 0

SMD: −0.62; 95%C.I.[−1.07; −0.17] P value for random effect= 0.0068

Cytokine: TNF−a − Treatment: Intensive

Study	After TRT Total	After TRT Mean	After TRT SD	Before TRT Total	Before TRT Mean	Before TRT SD	SMD	95%−CI	Weight (common)	Weight (random)
**Diabetes_binary:Sample with diabetic**										
Lei Chen 2012	42	10.12	3.3000	42	11.19	3.9700	−0.29	[−0.72; 0.14]	6.3%	7.2%
Lei Chen 2012	42	11.37	5.1800	42	11.19	3.9700	0.04	[−0.39; 0.47]	6.3%	7.2%
Lei Chen 2012	42	10.78	2.9700	42	11.19	3.9700	−0.12	[−0.54; 0.31]	6.3%	7.2%
Hilana Paula Carillo ARTESE 2015	12	6200.00	2000.0000	12	6000.00	1800.0000	0.10	[−0.70; 0.90]	1.8%	3.9%
de Sousa Rabelo 2020	15	0.57	1.5295	15	0.44	0.8180	0.10	[−0.61; 0.82]	2.3%	4.4%
Wang 2017	19	17.94	7.9410	19	25.59	14.2650	−0.65	[−1.30; 0.01]	2.7%	4.9%
Geisinger 2016	240	1.91	1.2900	240	1.91	2.4500	0.00	[−0.18; 0.18]	36.2%	10.1%
**Common effect model**	**412**			**412**			**−0.06**	**[−0.20; 0.08]**	**62.0%**	**.**
**Random effects model**							**−0.06**	**[−0.20; 0.08]**	**.**	**44.9%**
Heterogeneity: $I^2 = 0\%$, $\tau^2 = 0$, $p = 0.51$										
**Diabetes_binary:Sample without diabetic**										
Poliana Mendes Duarte 2010	14	1.16	0.7100	14	1.15	0.4500	0.02	[−0.72; 0.76]	2.1%	4.3%
Umut Altay 2013	22	3860000.00	2140000.0000	22	5830000.00	4830000.0000	−0.52	[−1.12; 0.08]	3.2%	5.4%
Umut Altay 2013	24	3270000.00	870000.0000	24	3870000.00	1500000.0000	−0.48	[−1.06; 0.09]	3.5%	5.6%
Fiorini T 2012	27	1.00	0.0700	27	1.00	0.1000	0.00	[−0.53; 0.53]	4.1%	6.0%
Pradeep Koppolu 2013	20	0.20	0.0161	20	0.22	0.0146	−1.21	[−1.89; −0.53]	2.5%	4.7%
Escobar Arregoces 2021	19	14.29	1.0500	19	15.84	1.0600	−1.44	[−2.16; −0.72]	2.2%	4.4%
de Sousa Rabelo 2020	15	1.79	6.4900	15	2.24	3.3400	−0.08	[−0.80; 0.63]	2.3%	4.4%
Balci Yuce 2016	18	4.48	0.8900	18	7.92	11.6700	−0.41	[−1.07; 0.25]	2.7%	4.9%
Yong−Wei Fu 2015	54	14.55	5.0700	54	16.94	4.0300	−0.52	[−0.90; −0.13]	7.9%	7.7%
Yong−Wei Fu 2015	54	13.65	4.6300	54	16.94	4.0300	−0.75	[−1.14; −0.36]	7.6%	7.6%
**Common effect model**	**267**			**267**			**−0.54**	**[−0.72; −0.37]**	**38.0%**	**.**
**Random effects model**							**−0.54**	**[−0.80; −0.27]**	**.**	**55.1%**
Heterogeneity: $I^2 = 52\%$, $\tau^2 = 0.0914$, $p = 0.03$										
**Common effect model**	**679**			**679**			**−0.24**	**[−0.35; −0.14]**	**100.0%**	**.**
**Random effects model**							**−0.35**	**[−0.54; −0.15]**	**.**	**100.0%**

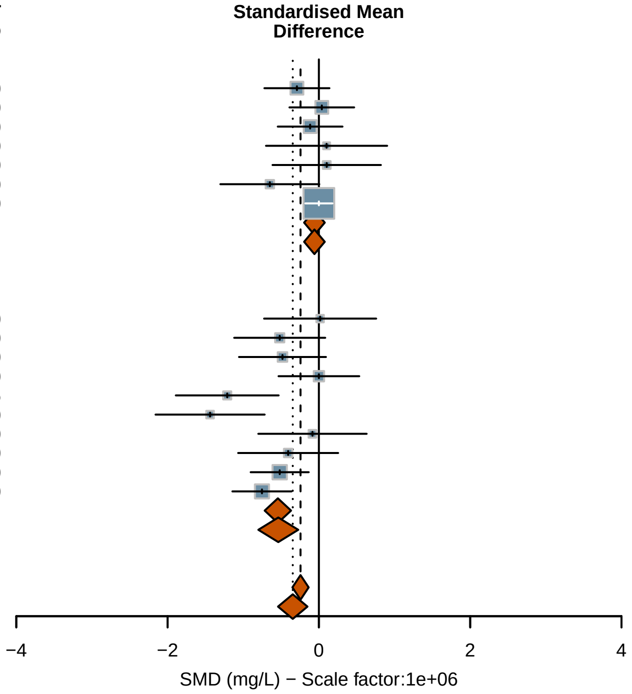

Heterogeneity: $I^2 = 62\%$, $\tau^2 = 0.0897$, $p < 0.01$
Test for subgroup differences (common effect): $\chi^2_1 = 18.25$, df = 1 ($p < 0.01$)
Test for subgroup differences (random effects): $\chi^2_1 = 9.92$, df = 1 ($p < 0.01$)

SMD: −0.24; 95%C.I.[−0.35; −0.14] P value for common effect= 0

SMD: −0.35; 95%C.I.[−0.54; −0.15] P value for random effect= 5e−04

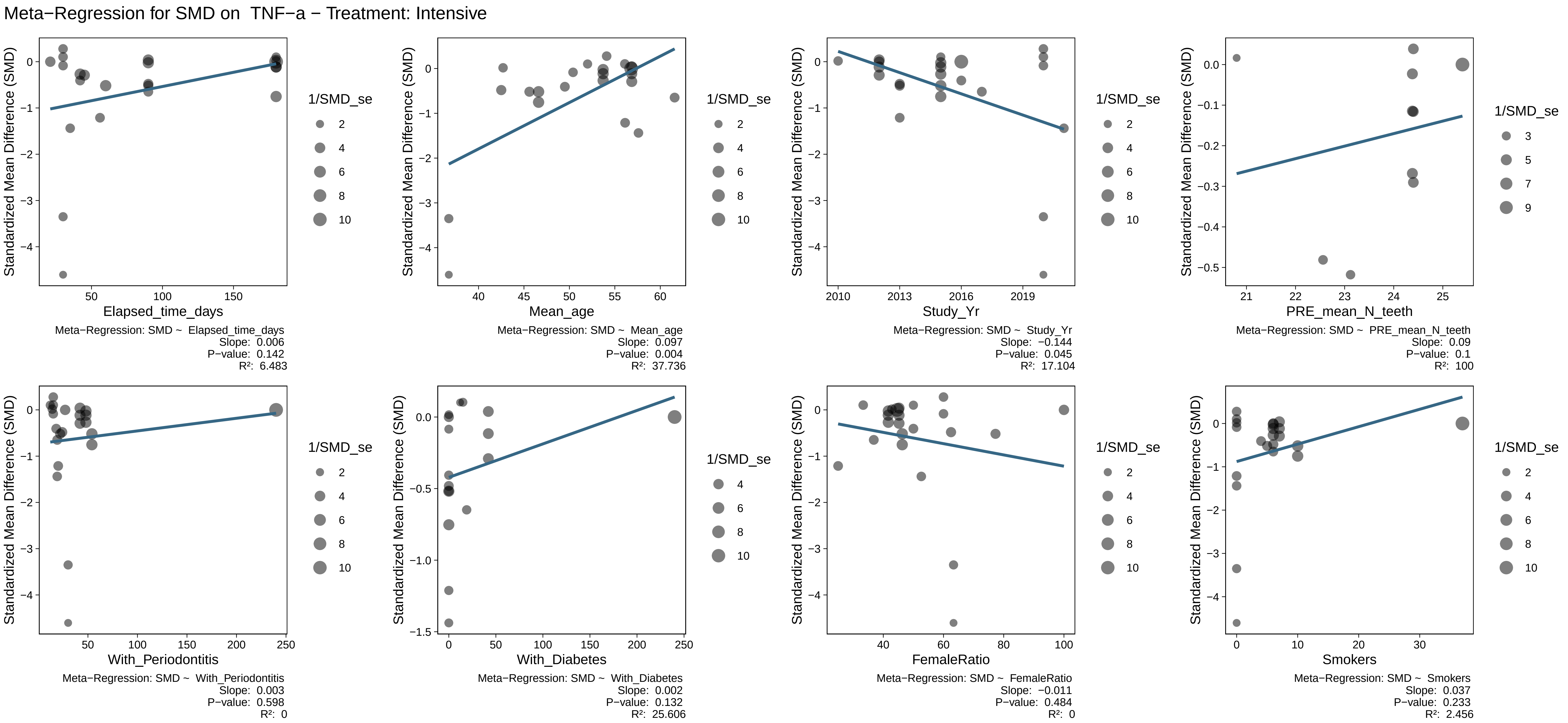
Meta−Regression for SMD on TNF−a − Treatment: Intensive
Standardized Mean Difference (SMD)
1/SMD_se
Elapsed_time_days
Meta−Regression: SMD ~ Elapsed_time_days
Slope: 0.006
P−value: 0.142
R²: 6.483
Mean_age
Meta−Regression: SMD ~ Mean_age
Slope: 0.097
P−value: 0.004
R²: 37.736
Study_Yr
Meta−Regression: SMD ~ Study_Yr
Slope: −0.144
P−value: 0.045
R²: 17.104
PRE_mean_N_teeth
Meta−Regression: SMD ~ PRE_mean_N_teeth
Slope: 0.09
P−value: 0.1
R²: 100
With_Periodontitis
Meta−Regression: SMD ~ With_Periodontitis
Slope: 0.003
P−value: 0.598
R²: 0
With_Diabetes
Meta−Regression: SMD ~ With_Diabetes
Slope: 0.002
P−value: 0.132
R²: 25.606
FemaleRatio
Meta−Regression: SMD ~ FemaleRatio
Slope: −0.011
P−value: 0.484
R²: 0
Smokers
Meta−Regression: SMD ~ Smokers
Slope: 0.037
P−value: 0.233
R²: 2.456
