# Supplementary material for: Temporal dynamics of early inflammatory markers after professional dental cleaning: a meta-analysis and spline-based meta-regression of TNF-α, IL-1β, IL-6, and (hs)CRP
Source: Front Immunol. 2025 Aug 28;16:1634622. doi: 10.3389/fimmu.2025.1634622 (PMC12423065; doi:10.3389/fimmu.2025.1634622)
Supplement: Supplementary file 1 [file DataSheet1.zip › Supplementary materials/PDF/TNF-a_Standard_results.pdf]

Cytokine: TNF−a − Treatment: Standard

Study	After TRT Total	After TRT Mean	After TRT SD	Before TRT Total	Before TRT Mean	Before TRT SD	SMD	95%−CI	Weight (common)	Weight (random)
**Days after treatment:30**										
Levent Kardesxler 2010	13	4.90	8.9000	13	5.00	8.0000	−0.01	[−0.78; 0.76]	3.5%	3.5%
Levent Kardesxler 2010	12	2.50	2.5000	12	3.50	2.2000	−0.41	[−1.22; 0.40]	3.1%	3.1%
Levent Kardesxler 2010	15	3.50	3.8000	15	5.00	5.0000	−0.33	[−1.05; 0.39]	3.9%	3.9%
Yasuko Shimada 2010	33	1.90	0.6000	33	1.80	0.7000	0.15	[−0.33; 0.63]	8.8%	8.8%
**Common effect model**	**73**			**73**			**−0.07**	**[−0.39; 0.26]**	**19.3%**	**.**
**Random effects model**							**−0.07**	**[−0.39; 0.26]**	**.**	**19.3%**
Heterogeneity: $I^2 = 0\%$, $\tau^2 = 0$, $p = 0.57$										
**Days after treatment:42**										
Ide M 2003	24	2.20	0.4700	24	2.21	0.5300	−0.02	[−0.59; 0.55]	6.4%	6.4%
**Days after treatment:45**										
Lei Chen 2012	43	10.68	36.3000	43	12.43	15.5500	−0.06	[−0.48; 0.36]	11.5%	11.5%
**Days after treatment:60**										
Yong−Wei Fu 2015	55	17.83	3.7400	55	17.70	3.8500	0.03	[−0.34; 0.41]	14.7%	14.7%
**Days after treatment:90**										
Levent Kardesxler 2010	13	2.90	1.9000	13	5.00	8.0000	−0.35	[−1.13; 0.43]	3.4%	3.4%
Levent Kardesxler 2010	12	30.00	1.8000	12	3.50	2.2000	> 12.73	[8.73; 16.73]	0.1%	0.1%
Levent Kardesxler 2010	15	3.30	2.4000	15	5.00	5.0000	−0.42	[−1.15; 0.30]	3.9%	3.9%
Lei Chen 2012	43	10.37	43.9000	43	12.43	15.5500	−0.06	[−0.48; 0.36]	11.5%	11.5%
**Common effect model**	**83**			**83**			**−0.10**	**[−0.43; 0.23]**	**18.9%**	**.**
**Random effects model**							**2.73**	**[−3.32; 8.77]**	**.**	**18.9%**
Heterogeneity: $I^2 = 93\%$, $\tau^2 = 36.9741$, $p < 0.01$										
**Days after treatment:180**										
Lei Chen 2012	43	11.03	37.7000	43	12.43	15.5500	−0.05	[−0.47; 0.37]	11.5%	11.5%
Hilana Paula Carillo ARTESE 2015	12	6000.00	1400.0000	12	5700.00	1600.0000	0.19	[−0.61; 1.00]	3.2%	3.2%
Yong−Wei Fu 2015	55	17.49	4.9000	55	17.70	3.8500	−0.05	[−0.42; 0.33]	14.7%	14.7%
**Common effect model**	**110**			**110**			**−0.02**	**[−0.29; 0.24]**	**29.3%**	**.**
**Random effects model**							**−0.02**	**[−0.29; 0.24]**	**.**	**29.3%**
Heterogeneity: $I^2 = 0\%$, $\tau^2 = 0$, $p = 0.86$										
**Common effect model**	**388**			**388**			**−0.04**	**[−0.18; 0.10]**	**100.0%**	**.**
**Random effects model**							**−0.04**	**[−0.18; 0.10]**	**.**	**100.0%**

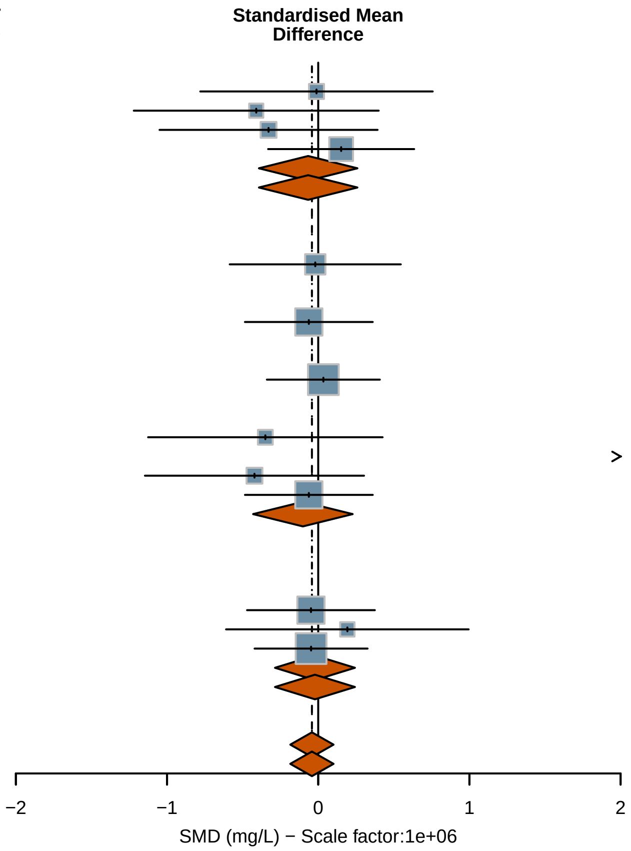

Heterogeneity: $I^2 = 70\%$, $\tau^2 < 0.0001$, $p < 0.01$
Test for subgroup differences (common effect): $\chi^2_5 = 0.34$, df = 5 ($p = 1.00$)
Test for subgroup differences (random effects): $\chi^2_5 = 0.99$, df = 5 ($p = 0.96$)

SMD: −0.04; 95%C.I.[−0.18; 0.1] P value for common effect= 0.5678

SMD: −0.04; 95%C.I.[−0.18; 0.1] P value for random effect= 0.5678

Cytokine: TNF−a − Treatment: Standard

Study	After TRT Total	After TRT Mean	After TRT SD	Before TRT Total	Before TRT Mean	Before TRT SD	SMD	95%−CI	Weight (common)	Weight (random)
**MonthsAfterTRT:4−6Wks**										
Ide M 2003	24	2.20	0.4700	24	2.21	0.5300	−0.02	[−0.59; 0.55]	6.4%	6.4%
Levent Kardesxler 2010	13	4.90	8.9000	13	5.00	8.0000	−0.01	[−0.78; 0.76]	3.5%	3.5%
Levent Kardesxler 2010	12	2.50	2.5000	12	3.50	2.2000	−0.41	[−1.22; 0.40]	3.1%	3.1%
Levent Kardesxler 2010	15	3.50	3.8000	15	5.00	5.0000	−0.33	[−1.05; 0.39]	3.9%	3.9%
Yasuko Shimada 2010	33	1.90	0.6000	33	1.80	0.7000	0.15	[−0.33; 0.63]	8.8%	8.8%
**Common effect model**	**97**			**97**			**−0.05**	**[−0.34; 0.23]**	**25.7%**	**.**
**Random effects model**							**−0.05**	**[−0.34; 0.23]**	**.**	**25.7%**
Heterogeneity: $I^2 = 0\%$, $\tau^2 = 0$, $p = 0.73$										
**MonthsAfterTRT:Within 3 Mo.**										
Levent Kardesxler 2010	13	2.90	1.9000	13	5.00	8.0000	−0.35	[−1.13; 0.43]	3.4%	3.4%
Levent Kardesxler 2010	12	30.00	1.8000	12	3.50	2.2000	> 12.73	[8.73; 16.73]	0.1%	0.1%
Levent Kardesxler 2010	15	3.30	2.4000	15	5.00	5.0000	−0.42	[−1.15; 0.30]	3.9%	3.9%
Lei Chen 2012	43	10.68	36.3000	43	12.43	15.5500	−0.06	[−0.48; 0.36]	11.5%	11.5%
Lei Chen 2012	43	10.37	43.9000	43	12.43	15.5500	−0.06	[−0.48; 0.36]	11.5%	11.5%
Yong−Wei Fu 2015	55	17.83	3.7400	55	17.70	3.8500	0.03	[−0.34; 0.41]	14.7%	14.7%
**Common effect model**	**181**			**181**			**−0.05**	**[−0.26; 0.17]**	**45.0%**	**.**
**Random effects model**							**1.68**	**[−2.06; 5.42]**	**.**	**45.0%**
Heterogeneity: $I^2 = 88\%$, $\tau^2 = 21.1371$, $p < 0.01$										
**MonthsAfterTRT:Within 6 Mo.**										
Lei Chen 2012	43	11.03	37.7000	43	12.43	15.5500	−0.05	[−0.47; 0.37]	11.5%	11.5%
Hilana Paula Carillo ARTESE 2015	12	6000.00	1400.0000	12	5700.00	1600.0000	0.19	[−0.61; 1.00]	3.2%	3.2%
Yong−Wei Fu 2015	55	17.49	4.9000	55	17.70	3.8500	−0.05	[−0.42; 0.33]	14.7%	14.7%
**Common effect model**	**110**			**110**			**−0.02**	**[−0.29; 0.24]**	**29.3%**	**.**
**Random effects model**							**−0.02**	**[−0.29; 0.24]**	**.**	**29.3%**
Heterogeneity: $I^2 = 0\%$, $\tau^2 = 0$, $p = 0.86$										
**Common effect model**	**388**			**388**			**−0.04**	**[−0.18; 0.10]**	**100.0%**	**.**
**Random effects model**							**−0.04**	**[−0.18; 0.10]**	**.**	**100.0%**

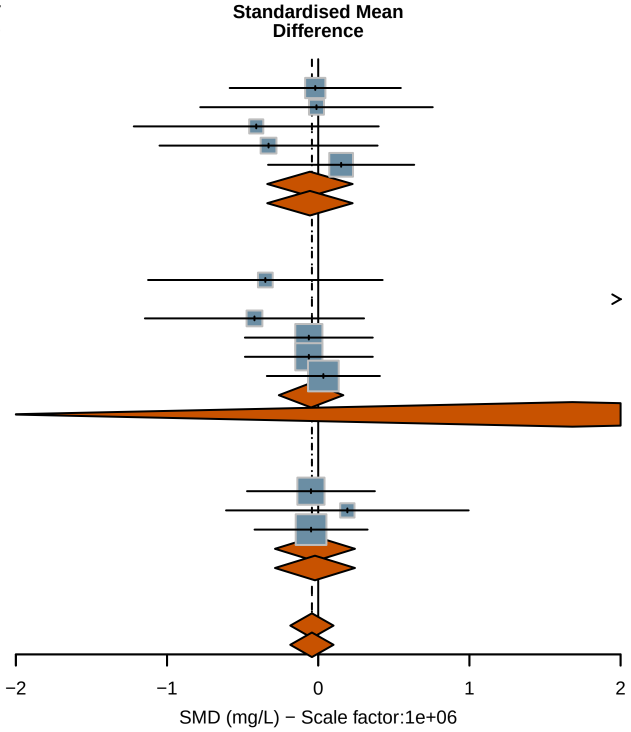

Heterogeneity: $I^2 = 70\%$, $\tau^2 < 0.0001$, $p < 0.01$
Test for subgroup differences (common effect): $\chi^2_2 = 0.03$, df = 2 ($p = 0.98$)
Test for subgroup differences (random effects): $\chi^2_2 = 0.84$, df = 2 ($p = 0.66$)

SMD: −0.04; 95%C.I.[−0.18; 0.1] P value for common effect= 0.5678

SMD: −0.04; 95%C.I.[−0.18; 0.1] P value for random effect= 0.5678

Cytokine: TNF−a − Treatment: Standard

Study	After TRT Total	After TRT Mean	After TRT SD	Before TRT Total	Before TRT Mean	Before TRT SD	SMD	95%−CI	Weight (common)	Weight (random)
**Assay type:ELISA**										
Ide M 2003	24	2.20	0.4700	24	2.21	0.5300	−0.02	[−0.59; 0.55]	6.4%	6.4%
Levent Kardesxler 2010	13	4.90	8.9000	13	5.00	8.0000	−0.01	[−0.78; 0.76]	3.5%	3.5%
Levent Kardesxler 2010	13	2.90	1.9000	13	5.00	8.0000	−0.35	[−1.13; 0.43]	3.4%	3.4%
Levent Kardesxler 2010	12	2.50	2.5000	12	3.50	2.2000	−0.41	[−1.22; 0.40]	3.1%	3.1%
Levent Kardesxler 2010	12	30.00	1.8000	12	3.50	2.2000	> 12.73	[8.73; 16.73]	0.1%	0.1%
Levent Kardesxler 2010	15	3.50	3.8000	15	5.00	5.0000	−0.33	[−1.05; 0.39]	3.9%	3.9%
Levent Kardesxler 2010	15	3.30	2.4000	15	5.00	5.0000	−0.42	[−1.15; 0.30]	3.9%	3.9%
Yasuko Shimada 2010	33	1.90	0.6000	33	1.80	0.7000	0.15	[−0.33; 0.63]	8.8%	8.8%
Lei Chen 2012	43	10.68	36.3000	43	12.43	15.5500	−0.06	[−0.48; 0.36]	11.5%	11.5%
Lei Chen 2012	43	10.37	43.9000	43	12.43	15.5500	−0.06	[−0.48; 0.36]	11.5%	11.5%
Lei Chen 2012	43	11.03	37.7000	43	12.43	15.5500	−0.05	[−0.47; 0.37]	11.5%	11.5%
Hilana Paula Carillo ARTESE 2015	12	6000.00	1400.0000	12	5700.00	1600.0000	0.19	[−0.61; 1.00]	3.2%	3.2%
Yong−Wei Fu 2015	55	17.83	3.7400	55	17.70	3.8500	0.03	[−0.34; 0.41]	14.7%	14.7%
Yong−Wei Fu 2015	55	17.49	4.9000	55	17.70	3.8500	−0.05	[−0.42; 0.33]	14.7%	14.7%
**Common effect model**	**388**			**388**			**−0.04**	**[−0.18; 0.10]**	**100.0%**	**.**
**Random effects model**							**−0.04**	**[−0.18; 0.10]**	**.**	**100.0%**
Heterogeneity: $I^2 = 70\%$, $\tau^2 = < 0.0001$, $p < 0.01$										
**Common effect model**	**388**			**388**			**−0.04**	**[−0.18; 0.10]**	**100.0%**	**.**
**Random effects model**							**−0.04**	**[−0.18; 0.10]**	**.**	**100.0%**

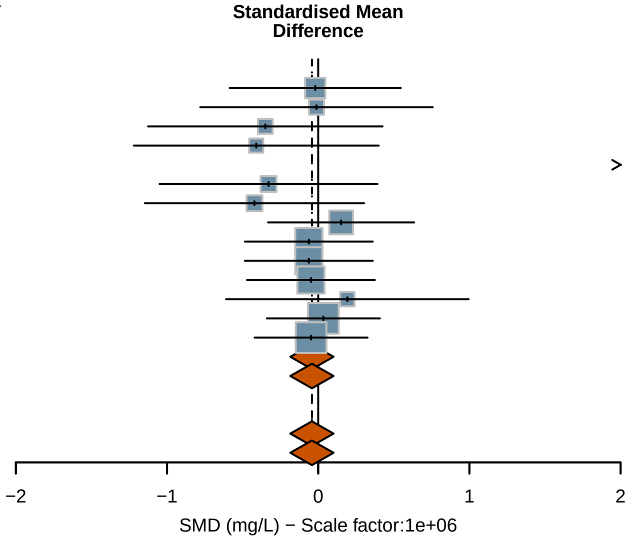

Heterogeneity: $I^2 = 70\%$, $\tau^2 < 0.0001$, $p < 0.01$
Test for subgroup differences (common effect): $\chi^2_0 = 0.00$, df = 0 (p = NA)
Test for subgroup differences (random effects): $\chi^2_0 = 0.00$, df = 0 (p = NA)

SMD: −0.04; 95%C.I.[−0.18; 0.1] P value for common effect= 0.5678

SMD: −0.04; 95%C.I.[−0.18; 0.1] P value for random effect= 0.5678

Cytokine: TNF−a − Treatment: Standard

Study	After TRT Total	After TRT Mean	After TRT SD	Before TRT Total	Before TRT Mean	Before TRT SD	SMD	95%−CI	Weight (common)	Weight (random)
**Bias judgment:High risk**										
Yasuko Shimada 2010	33	1.90	0.6000	33	1.80	0.7000	0.15	[−0.33; 0.63]	8.8%	8.8%
Hilana Paula Carillo ARTESE 2015	12	6000.00	1400.0000	12	5700.00	1600.0000	0.19	[−0.61; 1.00]	3.2%	3.2%
**Common effect model**	**45**			**45**			**0.16**	**[−0.25; 0.58]**	**12.0%**	**.**
**Random effects model**							**0.16**	**[−0.25; 0.58]**	**.**	**12.0%**
Heterogeneity: $I^2 = 0\%$, $\tau^2 = 0$, $p = 0.93$										
**Bias judgment:Low risk**										
Ide M 2003	24	2.20	0.4700	24	2.21	0.5300	−0.02	[−0.59; 0.55]	6.4%	6.4%
Levent Kardesxler 2010	13	4.90	8.9000	13	5.00	8.0000	−0.01	[−0.78; 0.76]	3.5%	3.5%
Levent Kardesxler 2010	13	2.90	1.9000	13	5.00	8.0000	−0.35	[−1.13; 0.43]	3.4%	3.4%
Levent Kardesxler 2010	12	2.50	2.5000	12	3.50	2.2000	−0.41	[−1.22; 0.40]	3.1%	3.1%
Levent Kardesxler 2010	12	30.00	1.8000	12	3.50	2.2000	> 12.73	[8.73; 16.73]	0.1%	0.1%
Levent Kardesxler 2010	15	3.50	3.8000	15	5.00	5.0000	−0.33	[−1.05; 0.39]	3.9%	3.9%
Levent Kardesxler 2010	15	3.30	2.4000	15	5.00	5.0000	−0.42	[−1.15; 0.30]	3.9%	3.9%
Yong−Wei Fu 2015	55	17.83	3.7400	55	17.70	3.8500	0.03	[−0.34; 0.41]	14.7%	14.7%
Yong−Wei Fu 2015	55	17.49	4.9000	55	17.70	3.8500	−0.05	[−0.42; 0.33]	14.7%	14.7%
**Common effect model**	**214**			**214**			**−0.08**	**[−0.27; 0.12]**	**53.7%**	**.**
**Random effects model**							**−0.08**	**[−0.27; 0.12]**	**.**	**53.7%**
Heterogeneity: $I^2 = 81\%$, $\tau^2 = 0$, $p < 0.01$										
**Bias judgment:Some concerns**										
Lei Chen 2012	43	10.68	36.3000	43	12.43	15.5500	−0.06	[−0.48; 0.36]	11.5%	11.5%
Lei Chen 2012	43	10.37	43.9000	43	12.43	15.5500	−0.06	[−0.48; 0.36]	11.5%	11.5%
Lei Chen 2012	43	11.03	37.7000	43	12.43	15.5500	−0.05	[−0.47; 0.37]	11.5%	11.5%
**Common effect model**	**129**			**129**			**−0.06**	**[−0.30; 0.19]**	**34.4%**	**.**
**Random effects model**							**−0.06**	**[−0.30; 0.19]**	**.**	**34.4%**
Heterogeneity: $I^2 = 0\%$, $\tau^2 = 0$, $p = 1.00$										
**Common effect model**	**388**			**388**			**−0.04**	**[−0.18; 0.10]**	**100.0%**	**.**
**Random effects model**							**−0.04**	**[−0.18; 0.10]**	**.**	**100.0%**

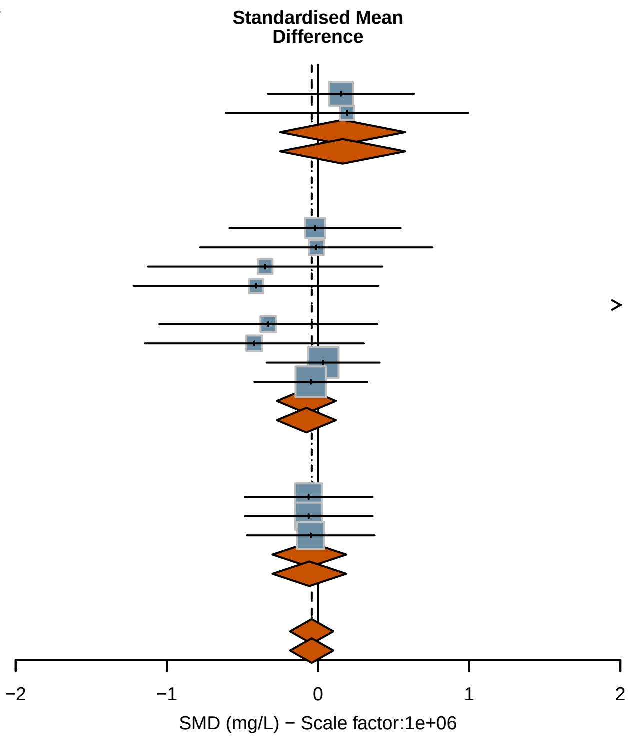

Heterogeneity: $I^2 = 70\%$, $\tau^2 < 0.0001$, $p < 0.01$
Test for subgroup differences (common effect): $\chi^2_2 = 1.08$, df = 2 ($p = 0.58$)
Test for subgroup differences (random effects): $\chi^2_2 = 1.08$, df = 2 ($p = 0.58$)

SMD: −0.04; 95%C.I.[−0.18; 0.1] P value for common effect= 0.5678

SMD: −0.04; 95%C.I.[−0.18; 0.1] P value for random effect= 0.5678

Cytokine: TNF−a − Treatment: Standard

Study	After TRT Total	After TRT Mean	After TRT SD	Before TRT Total	Before TRT Mean	Before TRT SD	SMD	95%−CI	Weight (common)	Weight (random)
**Study design:Comparative study**										
Levent Kardesxler 2010	13	4.90	8.9000	13	5.00	8.0000	−0.01	[−0.78; 0.76]	3.5%	3.5%
Levent Kardesxler 2010	13	2.90	1.9000	13	5.00	8.0000	−0.35	[−1.13; 0.43]	3.4%	3.4%
Levent Kardesxler 2010	12	2.50	2.5000	12	3.50	2.2000	−0.41	[−1.22; 0.40]	3.1%	3.1%
Levent Kardesxler 2010	12	30.00	1.8000	12	3.50	2.2000	12.73	[8.73; 16.73]	0.1%	0.1%
Levent Kardesxler 2010	15	3.50	3.8000	15	5.00	5.0000	−0.33	[−1.05; 0.39]	3.9%	3.9%
Levent Kardesxler 2010	15	3.30	2.4000	15	5.00	5.0000	−0.42	[−1.15; 0.30]	3.9%	3.9%
Yasuko Shimada 2010	33	1.90	0.6000	33	1.80	0.7000	0.15	[−0.33; 0.63]	8.8%	8.8%
**Common effect model**	**113**			**113**			**−0.09**	**[−0.37; 0.18]**	**26.7%**	**.**
**Random effects model**							**1.31**	**[−1.80; 4.42]**	**.**	**26.7%**
Heterogeneity: $I^2 = 86\%$, $\tau^2 = 17.0021$, $p < 0.01$										
**Study design:Prospective Interventional cohort study**										
Ide M 2003	24	2.20	0.4700	24	2.21	0.5300	−0.02	[−0.59; 0.55]	6.4%	6.4%
**Study design:RCT**										
Lei Chen 2012	43	10.68	36.3000	43	12.43	15.5500	−0.06	[−0.48; 0.36]	11.5%	11.5%
Lei Chen 2012	43	10.37	43.9000	43	12.43	15.5500	−0.06	[−0.48; 0.36]	11.5%	11.5%
Lei Chen 2012	43	11.03	37.7000	43	12.43	15.5500	−0.05	[−0.47; 0.37]	11.5%	11.5%
Hilana Paula Carillo ARTESE 2015	12	6000.00	1400.0000	12	5700.00	1600.0000	0.19	[−0.61; 1.00]	3.2%	3.2%
Yong−Wei Fu 2015	55	17.83	3.7400	55	17.70	3.8500	0.03	[−0.34; 0.41]	14.7%	14.7%
Yong−Wei Fu 2015	55	17.49	4.9000	55	17.70	3.8500	−0.05	[−0.42; 0.33]	14.7%	14.7%
**Common effect model**	**251**			**251**			**−0.02**	**[−0.20; 0.15]**	**66.9%**	**.**
**Random effects model**							**−0.02**	**[−0.20; 0.15]**	**.**	**66.9%**
Heterogeneity: $I^2 = 0\%$, $\tau^2 = 0$, $p = 0.99$										
**Common effect model**	**388**			**388**			**−0.04**	**[−0.18; 0.10]**	**100.0%**	**.**
**Random effects model**							**−0.04**	**[−0.18; 0.10]**	**.**	**100.0%**

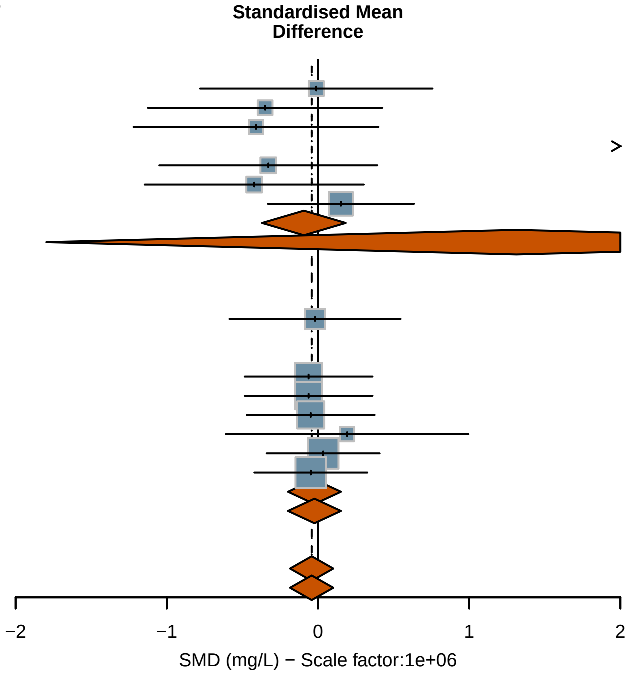

Heterogeneity: $I^2 = 70\%$, $\tau^2 < 0.0001$, $p < 0.01$
Test for subgroup differences (common effect): $\chi^2_2 = 0.18$, df = 2 ($p = 0.91$)
Test for subgroup differences (random effects): $\chi^2_2 = 0.71$, df = 2 ($p = 0.70$)

SMD: −0.04; 95%C.I.[−0.18; 0.1] P value for common effect= 0.5678

SMD: −0.04; 95%C.I.[−0.18; 0.1] P value for random effect= 0.5678

Cytokine: TNF−a − Treatment: Standard

Study	Total	Mean	After TRT SD	Total	Mean	Before TRT SD	SMD	95%−CI	Weight (common)	Weight (random)
**Additional_devices:Sex mixed**										
Ide M 2003	24	2.20	0.4700	24	2.21	0.5300	−0.02	[−0.59; 0.55]	6.4%	6.4%
Levent Kardesxler 2010	13	4.90	8.9000	13	5.00	8.0000	−0.01	[−0.78; 0.76]	3.5%	3.5%
Levent Kardesxler 2010	13	2.90	1.9000	13	5.00	8.0000	−0.35	[−1.13; 0.43]	3.4%	3.4%
Levent Kardesxler 2010	12	2.50	2.5000	12	3.50	2.2000	−0.41	[−1.22; 0.40]	3.1%	3.1%
Levent Kardesxler 2010	12	30.00	1.8000	12	3.50	2.2000	12.73	[8.73; 16.73]	0.1%	0.1%
Levent Kardesxler 2010	15	3.50	3.8000	15	5.00	5.0000	−0.33	[−1.05; 0.39]	3.9%	3.9%
Levent Kardesxler 2010	15	3.30	2.4000	15	5.00	5.0000	−0.42	[−1.15; 0.30]	3.9%	3.9%
Yasuko Shimada 2010	33	1.90	0.6000	33	1.80	0.7000	0.15	[−0.33; 0.63]	8.8%	8.8%
Lei Chen 2012	43	10.68	36.3000	43	12.43	15.5500	−0.06	[−0.48; 0.36]	11.5%	11.5%
Lei Chen 2012	43	10.37	43.9000	43	12.43	15.5500	−0.06	[−0.48; 0.36]	11.5%	11.5%
Lei Chen 2012	43	11.03	37.7000	43	12.43	15.5500	−0.05	[−0.47; 0.37]	11.5%	11.5%
Hilana Paula Carillo ARTESE 2015	12	6000.00	1400.0000	12	5700.00	1600.0000	0.19	[−0.61; 1.00]	3.2%	3.2%
Yong−Wei Fu 2015	55	17.83	3.7400	55	17.70	3.8500	0.03	[−0.34; 0.41]	14.7%	14.7%
Yong−Wei Fu 2015	55	17.49	4.9000	55	17.70	3.8500	−0.05	[−0.42; 0.33]	14.7%	14.7%
**Common effect model**	**388**			**388**			**−0.04**	**[−0.18; 0.10]**	**100.0%**	**.**
**Random effects model**							**−0.04**	**[−0.18; 0.10]**	**.**	**100.0%**
Heterogeneity: I^2 = 70%, τ^2 = < 0.0001, p < 0.01										
**Common effect model**	**388**			**388**			**−0.04**	**[−0.18; 0.10]**	**100.0%**	**.**
**Random effects model**							**−0.04**	**[−0.18; 0.10]**	**.**	**100.0%**

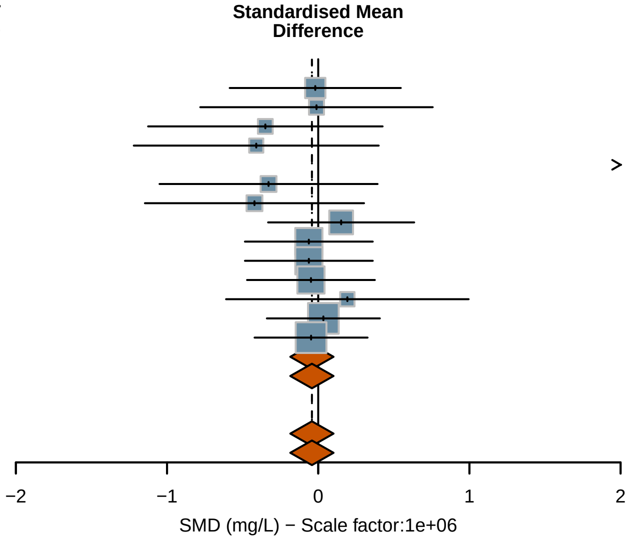

Heterogeneity: I^2 = 70%, τ^2 < 0.0001, p < 0.01
Test for subgroup differences (common effect): χ^2_0 = 0.00, df = 0 (p = NA)
Test for subgroup differences (random effects): χ^2_0 = 0.00, df = 0 (p = NA)

SMD: −0.04; 95%C.I.[−0.18; 0.1] P value for common effect= 0.5678

SMD: −0.04; 95%C.I.[−0.18; 0.1] P value for random effect= 0.5678

Cytokine: TNF−a − Treatment: Standard

Study	After TRT Total	After TRT Mean	After TRT SD	Before TRT Total	Before TRT Mean	Before TRT SD	SMD	95%−CI	Weight (common)	Weight (random)
**Female_binary:Sample with smokers**										
Levent Kardesxler 2010	13	4.90	8.9000	13	5.00	8.0000	−0.01	[−0.78; 0.76]	3.6%	3.6%
Levent Kardesxler 2010	13	2.90	1.9000	13	5.00	8.0000	−0.35	[−1.13; 0.43]	3.5%	3.5%
Levent Kardesxler 2010	12	2.50	2.5000	12	3.50	2.2000	−0.41	[−1.22; 0.40]	3.2%	3.2%
Levent Kardesxler 2010	12	30.00	1.8000	12	3.50	2.2000	> 12.73	[8.73; 16.73]	0.1%	0.1%
Levent Kardesxler 2010	15	3.50	3.8000	15	5.00	5.0000	−0.33	[−1.05; 0.39]	4.1%	4.1%
Levent Kardesxler 2010	15	3.30	2.4000	15	5.00	5.0000	−0.42	[−1.15; 0.30]	4.0%	4.0%
Yasuko Shimada 2010	33	1.90	0.6000	33	1.80	0.7000	0.15	[−0.33; 0.63]	9.1%	9.1%
Lei Chen 2012	43	10.68	36.3000	43	12.43	15.5500	−0.06	[−0.48; 0.36]	11.8%	11.8%
Lei Chen 2012	43	10.37	43.9000	43	12.43	15.5500	−0.06	[−0.48; 0.36]	11.8%	11.8%
Lei Chen 2012	43	11.03	37.7000	43	12.43	15.5500	−0.05	[−0.47; 0.37]	11.8%	11.8%
Yong−Wei Fu 2015	55	17.83	3.7400	55	17.70	3.8500	0.03	[−0.34; 0.41]	15.1%	15.1%
Yong−Wei Fu 2015	55	17.49	4.9000	55	17.70	3.8500	−0.05	[−0.42; 0.33]	15.1%	15.1%
**Common effect model**	**352**			**352**			**−0.05**	**[−0.20; 0.10]**	**93.4%**	**.**
**Random effects model**							**−0.05**	**[−0.20; 0.10]**	**.**	**93.4%**
Heterogeneity: $I^2 = 74\%$, $\tau^2 = < 0.0001$, $p < 0.01$										
**Female_binary:Sample without smokers**										
Ide M 2003	24	2.20	0.4700	24	2.21	0.5300	−0.02	[−0.59; 0.55]	6.6%	6.6%
**Common effect model**	**376**			**376**			**−0.05**	**[−0.19; 0.10]**	**100.0%**	**.**
**Random effects model**							**−0.05**	**[−0.19; 0.10]**	**.**	**100.0%**

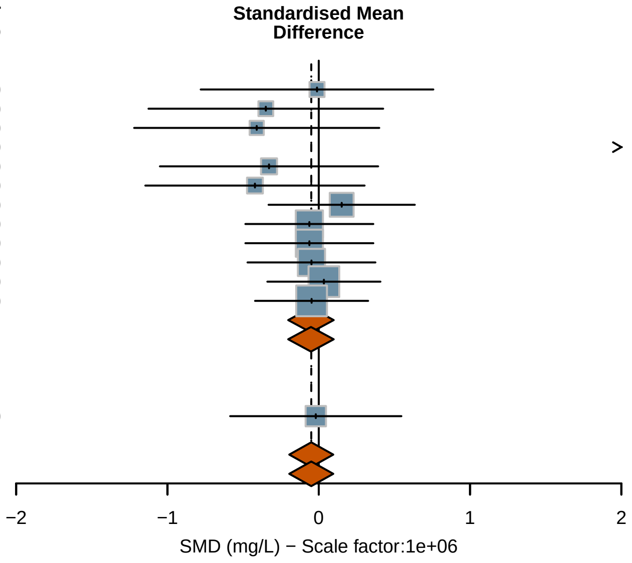

Heterogeneity: $I^2 = 72\%$, $\tau^2 < 0.0001$, $p < 0.01$
Test for subgroup differences (common effect): $\chi^2_1 = 0.01$, df = 1 ($p = 0.91$)
Test for subgroup differences (random effects): $\chi^2_1 = 0.01$, df = 1 ($p = 0.91$)

SMD: −0.05; 95%C.I.[−0.19; 0.1] P value for common effect= 0.5055

SMD: −0.05; 95%C.I.[−0.19; 0.1] P value for random effect= 0.5055

Cytokine: TNF−a − Treatment: Standard

Study	After TRT			Before TRT					Weight	Weight
	Total	Mean	SD	Total	Mean	SD	SMD	95%−CI	(common)	(random)
**Smoke_binary:Sample with diabetic**										
Levent Kardesxler 2010	13	4.90	8.9000	13	5.00	8.0000	−0.01	[−0.78; 0.76]	3.5%	3.5%
Levent Kardesxler 2010	13	2.90	1.9000	13	5.00	8.0000	−0.35	[−1.13; 0.43]	3.4%	3.4%
Levent Kardesxler 2010	12	2.50	2.5000	12	3.50	2.2000	−0.41	[−1.22; 0.40]	3.1%	3.1%
Levent Kardesxler 2010	12	30.00	1.8000	12	3.50	2.2000	> 12.73	[8.73; 16.73]	0.1%	0.1%
Lei Chen 2012	43	10.68	36.3000	43	12.43	15.5500	−0.06	[−0.48; 0.36]	11.5%	11.5%
Lei Chen 2012	43	10.37	43.9000	43	12.43	15.5500	−0.06	[−0.48; 0.36]	11.5%	11.5%
Lei Chen 2012	43	11.03	37.7000	43	12.43	15.5500	−0.05	[−0.47; 0.37]	11.5%	11.5%
Hilana Paula Carillo ARTESE 2015	12	6000.00	1400.0000	12	5700.00	1600.0000	0.19	[−0.61; 1.00]	3.2%	3.2%
**Common effect model**	**191**			**191**			**−0.05**	**[−0.25; 0.16]**	**47.7%**	**.**
**Random effects model**							**1.16**	**[−1.40; 3.72]**	**.**	**47.7%**
Heterogeneity: $I^2 = 83\%$, $\tau^2 = 13.1408$, $p < 0.01$										
**Smoke_binary:Sample without diabetic**										
Ide M 2003	24	2.20	0.4700	24	2.21	0.5300	−0.02	[−0.59; 0.55]	6.4%	6.4%
Levent Kardesxler 2010	15	3.50	3.8000	15	5.00	5.0000	−0.33	[−1.05; 0.39]	3.9%	3.9%
Levent Kardesxler 2010	15	3.30	2.4000	15	5.00	5.0000	−0.42	[−1.15; 0.30]	3.9%	3.9%
Yasuko Shimada 2010	33	1.90	0.6000	33	1.80	0.7000	0.15	[−0.33; 0.63]	8.8%	8.8%
Yong−Wei Fu 2015	55	17.83	3.7400	55	17.70	3.8500	0.03	[−0.34; 0.41]	14.7%	14.7%
Yong−Wei Fu 2015	55	17.49	4.9000	55	17.70	3.8500	−0.05	[−0.42; 0.33]	14.7%	14.7%
**Common effect model**	**197**			**197**			**−0.04**	**[−0.23; 0.16]**	**52.3%**	**.**
**Random effects model**							**−0.04**	**[−0.23; 0.16]**	**.**	**52.3%**
Heterogeneity: $I^2 = 0\%$, $\tau^2 = 0$, $p = 0.79$										
**Common effect model**	**388**			**388**			**−0.04**	**[−0.18; 0.10]**	**100.0%**	**.**
**Random effects model**							**−0.04**	**[−0.18; 0.10]**	**.**	**100.0%**

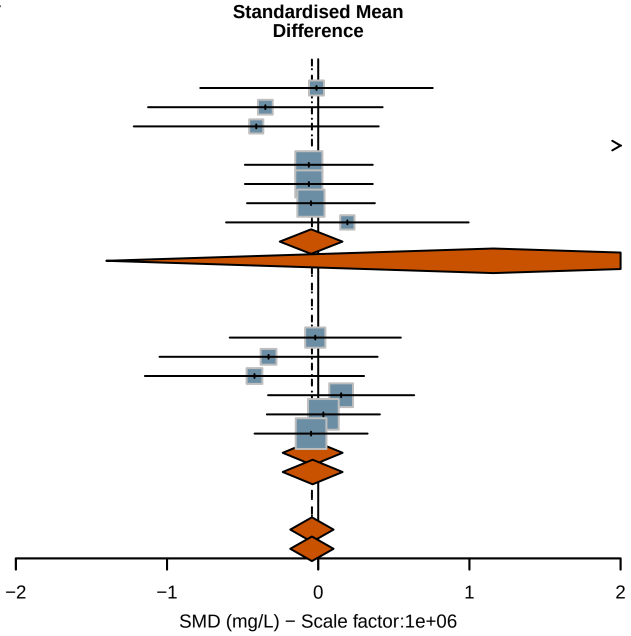

Heterogeneity: $I^2 = 70\%$, $\tau^2 < 0.0001$, $p < 0.01$
Test for subgroup differences (common effect): $\chi^2_1 = 0.00$, df = 1 ($p = 0.94$)
Test for subgroup differences (random effects): $\chi^2_1 = 0.83$, df = 1 ($p = 0.36$)

SMD: −0.04; 95%C.I.[−0.18; 0.1] P value for common effect= 0.5678

SMD: −0.04; 95%C.I.[−0.18; 0.1] P value for random effect= 0.5678

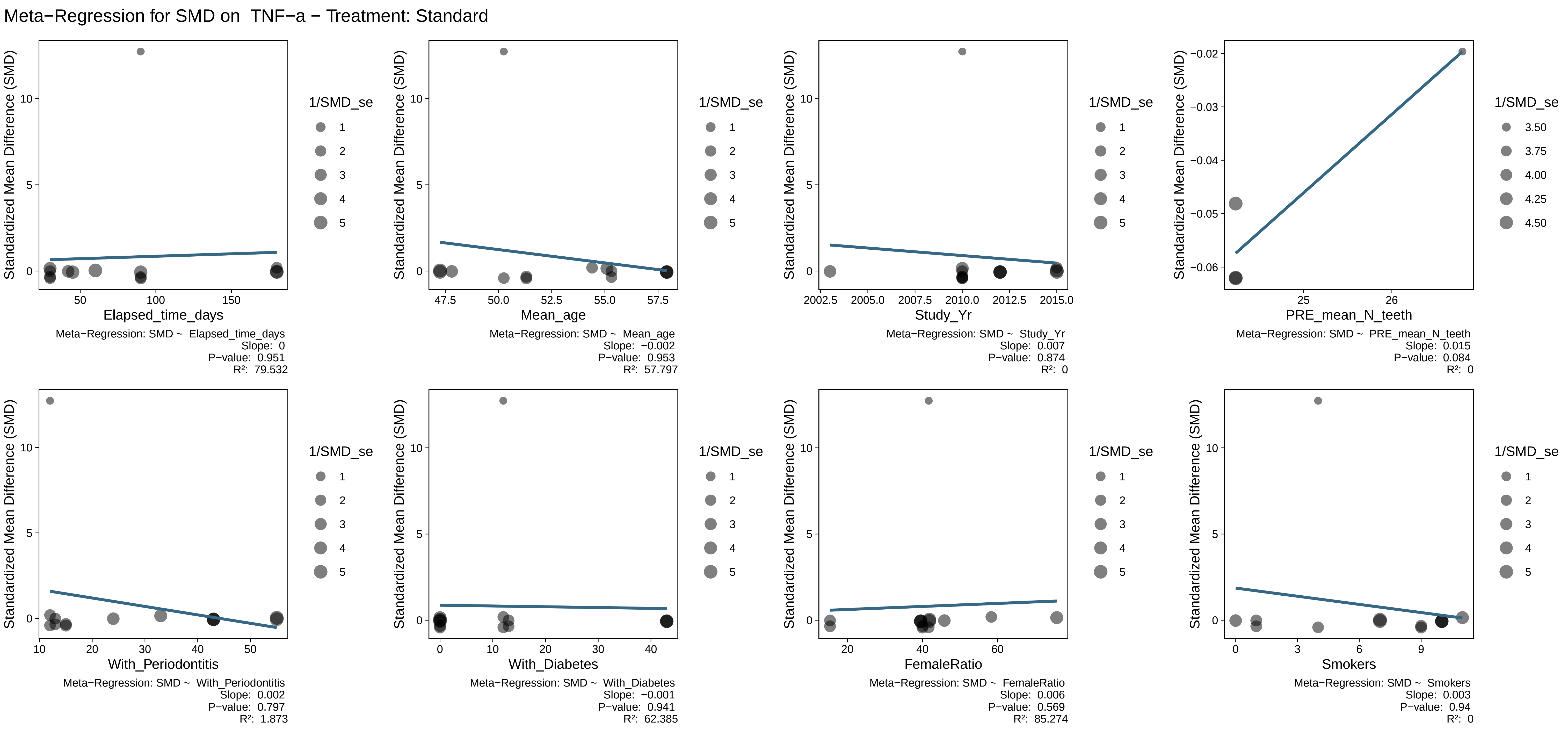
Meta−Regression for SMD on TNF−a − Treatment: Standard
Standardized Mean Difference (SMD)
1/SMD_se
Elapsed_time_days
Meta−Regression: SMD ~ Elapsed_time_days
Slope: 0
P−value: 0.951
R²: 79.532
Mean_age
Meta−Regression: SMD ~ Mean_age
Slope: −0.002
P−value: 0.953
R²: 57.797
Study_Yr
Meta−Regression: SMD ~ Study_Yr
Slope: 0.007
P−value: 0.874
R²: 0
PRE_mean_N_teeth
Meta−Regression: SMD ~ PRE_mean_N_teeth
Slope: 0.015
P−value: 0.084
R²: 0
With_Periodontitis
Meta−Regression: SMD ~ With_Periodontitis
Slope: 0.002
P−value: 0.797
R²: 1.873
With_Diabetes
Meta−Regression: SMD ~ With_Diabetes
Slope: −0.001
P−value: 0.941
R²: 62.385
FemaleRatio
Meta−Regression: SMD ~ FemaleRatio
Slope: 0.006
P−value: 0.569
R²: 85.274
Smokers
Meta−Regression: SMD ~ Smokers
Slope: 0.003
P−value: 0.94
R²: 0
