# Supplementary material for: Temporal dynamics of early inflammatory markers after professional dental cleaning: a meta-analysis and spline-based meta-regression of TNF-α, IL-1β, IL-6, and (hs)CRP
Source: Front Immunol. 2025 Aug 28;16:1634622. doi: 10.3389/fimmu.2025.1634622 (PMC12423065; doi:10.3389/fimmu.2025.1634622)
Supplement: Supplementary file 1 [file DataSheet1.zip › Supplementary materials/Supplementary Figure 1.docx]

**SUPPLEMENTARY FIGURE 1**


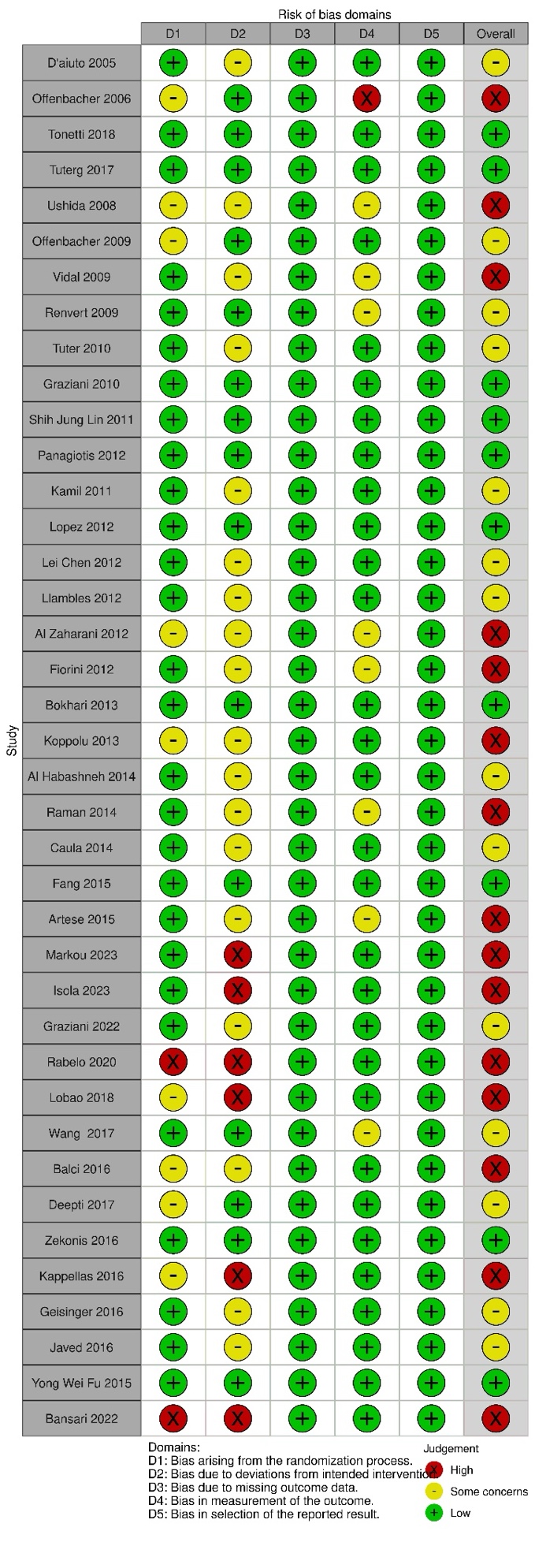


**Figure 1:** Cochrane Risk of Bias template (RoB 2.0) for randomized trials. [1]

[1] McGuinness, LA, Higgins, JPT. Risk-of-bias VISualization (robvis): An R package and Shiny web app for visualizing risk-of-bias assessments. Res Syn Meth. 2020; 1- 7. <https://doi.org/10.1002/jrsm.1411>
