# Supplementary material for: Temporal dynamics of early inflammatory markers after professional dental cleaning: a meta-analysis and spline-based meta-regression of TNF-α, IL-1β, IL-6, and (hs)CRP
Source: Front Immunol. 2025 Aug 28;16:1634622. doi: 10.3389/fimmu.2025.1634622 (PMC12423065; doi:10.3389/fimmu.2025.1634622)
Supplement: Supplementary file 1 [file DataSheet1.zip › Supplementary materials/Supplementary File 1.html]

Supplementary Results – Cardisciani et al 2025


# Supplementary Results

Full extended results of the meta-analysis titled:  
**Temporal dynamics of early inflammatory markers after professional dental cleaning: a meta-analysis and spline-based meta-regression of TNF-α, IL-1β, IL-6, and (hs)CRP**  
Authors: Martina Cardisciani, Sara Di Nicolantonio, Serena Altamura, Eleonora Ortu, Rita Del Pinto, Davide Pietropaoli

## Supplementary Files

- CRP – Intensive Treatment Results (PDF)
- CRP – Standard Treatment Results (PDF)
- IL-1β – Intensive Treatment Results (PDF)
- IL-1β – Standard Treatment Results (PDF)
- IL-6 – Intensive Treatment Results (PDF)
- IL-6 – Standard Treatment Results (PDF)
- TNF-α – Intensive Treatment Results (PDF)
- TNF-α – Standard Treatment Results (PDF)
- hs-CRP – Intensive Treatment Results (PDF)
- hs-CRP – Standard Treatment Results (PDF)

If any file does not open directly in your browser, please ensure your PDF viewer is enabled or download the file manually.
