# Supplementary material for: Temporal dynamics of early inflammatory markers after professional dental cleaning: a meta-analysis and spline-based meta-regression of TNF-α, IL-1β, IL-6, and (hs)CRP
Source: Front Immunol. 2025 Aug 28;16:1634622. doi: 10.3389/fimmu.2025.1634622 (PMC12423065; doi:10.3389/fimmu.2025.1634622)
Supplement: Supplementary file 1 [file DataSheet1.zip › Supplementary materials/Supplementary Methods 1.docx]

**SUPPLEMENTARY METHODS 1**

**Methods 1.** Search Strategy

**Date of final search:** January 2024

**Primary Search Strategy for Pubmed:** ("periodontal diseases"[MeSH Terms] AND ("cytokines"[MeSH Terms] OR "c reactive protein"[MeSH Terms])) AND ((fha[Filter]) AND (clinicalstudy[Filter] OR clinicaltrial[Filter] OR clinicaltrialprotocol[Filter] OR clinicaltrialphasei[Filter] OR clinicaltrialphaseii[Filter] OR clinicaltrialphaseiii[Filter] OR clinicaltrialphaseiv[Filter] OR comparativestudy[Filter] OR controlledclinicaltrial[Filter] OR dataset[Filter] OR meta-analysis[Filter] OR observationalstudy[Filter] OR pragmaticclinicaltrial[Filter] OR randomizedcontrolledtrial[Filter]))
