# Supplementary material for: Temporal dynamics of early inflammatory markers after professional dental cleaning: a meta-analysis and spline-based meta-regression of TNF-α, IL-1β, IL-6, and (hs)CRP
Source: Front Immunol. 2025 Aug 28;16:1634622. doi: 10.3389/fimmu.2025.1634622 (PMC12423065; doi:10.3389/fimmu.2025.1634622)
Supplement: Supplementary file 1 [file DataSheet1.zip › Supplementary materials/Supplementary Methods 2.docx]

**SUPPLEMENTARY METHODS 2**

**Methods 2.** Data Collection Form

**Study Identification**

DOI

Year of pubblication

Authors’ name

**Methods**

Assay type

Sampling Site

Study design

Quality tool of bias

Bias judgment

**Population**

Mean age

Sex ( n. male, n. female)

Smokers

Participants with periodontitis

Participants with other conditions or systemic diseases (diabetes, hypertension, cardiovascular dideases, hyperlipidemia, chronic kidney disease, metabolic syndrome, obesity and pregnancy)

**Interventions**

Type of treatment (Standard or Intensive non-surgical periodontal treatment)

Additional devices used

**Outcome**

Cytokine (type of cytokine, units, PRE-mean, PRE-SD, POST-mean and POST-SD)
