# Supplementary material for: Temporal dynamics of early inflammatory markers after professional dental cleaning: a meta-analysis and spline-based meta-regression of TNF-α, IL-1β, IL-6, and (hs)CRP
Source: Front Immunol. 2025 Aug 28;16:1634622. doi: 10.3389/fimmu.2025.1634622 (PMC12423065; doi:10.3389/fimmu.2025.1634622)
Supplement: Supplementary file 1 [file DataSheet1.zip › Supplementary materials/Supplementary Table 2.docx]

**SUPPLEMENTARY TABLE 2**

| **NON RCT studies** |  | **Selection** | | | **Comparability** | | | **Exposure** | | | **Total quality score** |  |
| --- | --- | --- | --- | --- | --- | --- | --- | --- | --- | --- | --- | --- |
| **First Author** | **Year** | **The case defination is adequate with independent validation** | **Consecutive or obviously representative series of cases** | **Community controls** | **Controls with no hystory of disease (endpoint)** | **Cases and controls with comparable ages** | **Cases and controls with comparability on any other factors** | **Ascertainment of exposure using secure records (eg surgical records) or structured interviews with blinding to case/control statuses** | **Ascertainment of exposure using the same method for cases and controls** | **Ascertainment of exposure with non-response rate for both groups** |  |  |
|  |  |  |  |  |  |  |  |  |  |  |  |  |
|  |  |  |  |  |  |  |  |  |  |  |  |  |
|  |  |  |  |  |  |  |  |  |  |  |  |  |
|  |  |  |  |  |  |  |  |  |  |  |  |  |
|  |  |  |  |  |  |  |  |  |  |  |  |  |
|  |  |  |  |  |  |  |  |  |  |  |  |  |
|  |  |  |  |  |  |  |  |  |  |  |  |  |
|  |  |  |  |  |  |  |  |  |  |  |  |  |
| Kardesxler | 2010 |  | * | * |  | * | * | * | * | * | low |  |
| Mendes Duarte | 2010 |  |  | * | * | * | * | * |  | * | some concerns |  |
| Shimada | 2010 |  |  |  | * | * | * | * |  |  | high |  |
| Fentog | 2010 | * | * |  | * | * | * | * |  |  | some concerns |  |
| Vilela | 2011 |  |  | * |  | * | * | * | * | * | some concerns |  |
| Umut | 2013 |  |  | * |  | * | * | * | * |  | high |  |
| Patil | 2013 | * | * | * |  | * | * | * | * | * | low |  |
| Munenaga | 2013 | * | * | * |  | * | * | * |  |  | some concerns |  |
| Arregocés | 2021 | * | * |  |  |  |  | * |  |  | high |  |
| Anwar | 2020 | * | * | * | * | * | * | * |  |  | low |  |
| Ide | 2003 |  |  | * | * | * | * | * | * | * | low |  |

**Table 2.** New Ottawa Scale (NOS) for assessing Risk of Bias (RoB) of Cohort and Case-control studies.
