# Supplementary material for: Temporal dynamics of early inflammatory markers after professional dental cleaning: a meta-analysis and spline-based meta-regression of TNF-α, IL-1β, IL-6, and (hs)CRP
Source: Front Immunol. 2025 Aug 28;16:1634622. doi: 10.3389/fimmu.2025.1634622 (PMC12423065; doi:10.3389/fimmu.2025.1634622)
Supplement: Supplementary file 1 [file DataSheet1.zip › Supplementary materials/Supplementary Table 3.docx]

**SUPPLEMENTARY TABLE 3**

| **Excluded studies** | **Justification** |
| --- | --- |
| Mohamed Farouk Elsadek 2023 | Patients with perimplantitis |
| Manpreet Kaur et al 2023 | No serum cytokines assessment |
| Shalinie King et al 2021 | Pilot study |
| Ahmet Cemil Talmac 2022 | No serum cytokines assessment |
| Giovana Lecio et al 2020 | No serum cytokines assessment |
| Małgorzata Woźniewicz  2018 | Patients with gingivitis |
| Hadi Bazyar 2019 | Wrong treatment: no NSPT treatment performed |
| Ruchir Trivedi 2018 | No results available: Study protocol |
| Raluca Cosgarea 2019 | No serum cytokines assessment |
| Jin Zhang 2017 | Wrong treatment: surgical treatment (Periodontal flap surgery for deep pockets) |
| Abdullah Seckin Ertugrul 2017 | No serum cytokines assessment |
| Kenneth E 2016 | Wrong therapy: antibiotics administration |
| Zaira F. Kharaeva 2015 | Wrong treatment: Surgical tartar removal |
| Holger F R Jentsch 2016 | No serum cytokines assessment |
| C Giannopoulou 2016 | Lack of accuracy in dosing |
| Thuy Anh Vu Pham  2022 | Lack of information regarding assay type |
| Fernanda C. Milanesi  2022 | Lack of information regarding assay type |
| Abdullah A. Alyousef  2017 | Lack of information regarding cytokines measurement |
| Alihan Bozoglan 2017 | Lack of information regarding assay type |
| [Maninder Kaur](https://pubmed.ncbi.nlm.nih.gov/?term=Kaur+M&cauthor_id=25079400) 2014 | Data not available and not extractable |
| [Anne Carolina Eleutério Leite](https://pubmed.ncbi.nlm.nih.gov/?term=Leite+AC&cauthor_id=24896165) 2014 | Data not available |
| [Manar Aljateeli](https://pubmed.ncbi.nlm.nih.gov/?term=Aljateeli+M&cauthor_id=24730621) 2014 | No serum cytokines assessment |
| [Maria F Kolbe](https://pubmed.ncbi.nlm.nih.gov/?term=Kolbe+MF&cauthor_id=24555751) 2014 | No serum cytokines assessment |
| [Kemal Üstün](https://pubmed.ncbi.nlm.nih.gov/?term=%C3%9Cst%C3%BCn+K&cauthor_id=24444428) 2014 | No serum cytokines assessment |
| [Hong Jiang](https://pubmed.ncbi.nlm.nih.gov/?term=Jiang+H&cauthor_id=24321402) 2013 | No results available (study protocol) |
| [Marco Aurélio Lumertz Saffi](https://pubmed.ncbi.nlm.nih.gov/?term=Saffi+MA&cauthor_id=24010954) 2013 | No results available (study protocol) |
| [L M Shaddox](https://pubmed.ncbi.nlm.nih.gov/?term=Shaddox+LM&cauthor_id=23788609) 2013 | Wrong therapy: antibiotics administration |
| [Vanessa H Luchesi](https://pubmed.ncbi.nlm.nih.gov/?term=Luchesi+VH&cauthor_id=23731242) 2013 | No serum cytokines assessment |
| [Véronique S Müller Campanile](https://pubmed.ncbi.nlm.nih.gov/?term=M%C3%BCller+Campanile+VS&cauthor_id=23660738) 2015 | No serum cytokines assessment |
| [Nupur Arora](https://pubmed.ncbi.nlm.nih.gov/?term=Arora+N&cauthor_id=23479592) 2013 | No serum cytokines assessment |
| [Peter Eickholz](https://pubmed.ncbi.nlm.nih.gov/?term=Eickholz+P&cauthor_id=23432024) 2013 | Wrong therapy: antibiotics administration |
| [Meggan M H Wehmeyer](https://pubmed.ncbi.nlm.nih.gov/?term=Wehmeyer+MM&cauthor_id=23261122) 2013 | Wrong therapy: antibiotics administration |
| [Martina Pirie](https://pubmed.ncbi.nlm.nih.gov/?term=Pirie+M&cauthor_id=23237583) 2013 | Serum cytokines assessed on cord serum of pregnant women |
| [L Nibali](https://pubmed.ncbi.nlm.nih.gov/?term=Nibali+L&cauthor_id=22918663) 2013 | No serum cytokines assessment |
| [V R Santos](https://pubmed.ncbi.nlm.nih.gov/?term=Santos+VR&cauthor_id=21806617) 2012 | No serum cytokines assessment |
| [Wei-Lian Sun](https://pubmed.ncbi.nlm.nih.gov/?term=Sun+WL&cauthor_id=21804283) 2011 | Wrong treatment: surgical treatment and antibiotics administration |
| [Jose R Gonzales](https://pubmed.ncbi.nlm.nih.gov/?term=Gonzales+JR&cauthor_id=21491990) 2011 | No data available |
| [Jeffrey B Payne](https://pubmed.ncbi.nlm.nih.gov/?term=Payne+JB&cauthor_id=21357860) 2011 | No data available |
| [Jorge H Ramírez](https://pubmed.ncbi.nlm.nih.gov/?term=Ram%C3%ADrez+JH&cauthor_id=21324167)  2011 | No results available (study protocol) |
| [Atsushi Ishikado](https://pubmed.ncbi.nlm.nih.gov/?term=Ishikado+A&cauthor_id=20930389) 2010 | No serum cytokines assessment |
| [Wei-Lian Sun](https://pubmed.ncbi.nlm.nih.gov/?term=Sun+WL&cauthor_id=20889139) 2010 | Wrong treatment: surgical treatment |
| [Rosamma Joseph](https://pubmed.ncbi.nlm.nih.gov/?term=Joseph+R&cauthor_id=20681817) 2011 | Wrong treatment: antibiotics administration |
| [Bryan S Michalowicz](https://pubmed.ncbi.nlm.nih.gov/?term=Michalowicz+BS&cauthor_id=19905943) 2009 | No data available |
| [Patricia A A O'Connell](https://pubmed.ncbi.nlm.nih.gov/?term=O%27Connell+PA&cauthor_id=18454655) 2008 | Wrong treatment: antibiotics administration |
| [Monica K Lee](https://pubmed.ncbi.nlm.nih.gov/?term=Lee+MK&cauthor_id=18341600) 2008 | No serum cytokines assessment |
| [Minoru Yamaoka](https://pubmed.ncbi.nlm.nih.gov/?term=Yamaoka+M&cauthor_id=18194335) 2008 | No serum cytokines assessment |
| [Francesco D'Aiuto](https://pubmed.ncbi.nlm.nih.gov/?term=D%27Aiuto+F&cauthor_id=17214734) 2007 | No data available |
| [Francesco D'Aiuto](https://pubmed.ncbi.nlm.nih.gov/?term=D%27Aiuto+F&cauthor_id=15086624) 2004 | No data available |
| [Vasiliki Avradopoulos](https://pubmed.ncbi.nlm.nih.gov/?term=Avradopoulos+V&cauthor_id=15079952) 2004 | No serum cytokines assessment |
| [Sultan Al-Mubarak](https://pubmed.ncbi.nlm.nih.gov/?term=Al-Mubarak+S&cauthor_id=11966926) 2002 | No data available |

Not listed are articles with inaccessible full texts and no author information provided as well as most excluded studies from registries and manual search

**Table 3:** Excluded studies and justifications
